# Identification of promoter elements in the *Dolichospermum circinale* AWQC131C saxitoxin gene cluster and the experimental analysis of their use for heterologous expression

**DOI:** 10.1186/s12866-020-1720-3

**Published:** 2020-02-17

**Authors:** Paul M. D’Agostino, Bakir Al-Sinawi, Rabia Mazmouz, Julia Muenchhoff, Brett A. Neilan, Michelle C. Moffitt

**Affiliations:** 1 School of Science, Western Sydney University, Sydney, NSW Australia; 2 School of Biotechnology and Biomolecular Sciences, University of New South Wales, Sydney, NSW Australia; 3 Biosystems Chemistry, Department of Chemistry, Technische Universität München, Garching, Germany; 4 Technical Biochemistry, Faculty of Chemistry and Food Chemistry, Technische Universität Dresden, Dresden, Germany; 5 Centre for Healthy Brain Ageing, School of Psychiatry, University of New South Wales, Sydney, Australia; 6 School of Environmental and Life Sciences, University of Newcastle, Callaghan, Australia

**Keywords:** Cyanobacteria, *Dolichospermum circinale*, *Anabaena circinalis*, Saxitoxin, Paralytic shellfish toxins, Regulation, Promoter, Transcription, Heterologous expression

## Abstract

**Background:**

*Dolichospermum circinale* is a filamentous bloom-forming cyanobacterium responsible for biosynthesis of the paralytic shellfish toxins (PST), including saxitoxin. PSTs are neurotoxins and in their purified form are important analytical standards for monitoring the quality of water and seafood and biomedical research tools for studying neuronal sodium channels. More recently, PSTs have been recognised for their utility as local anaesthetics. Characterisation of the transcriptional elements within the saxitoxin (*sxt*) biosynthetic gene cluster (BGC) is a first step towards accessing these molecules for biotechnology.

**Results:**

In *D. circinale* AWQC131C the *sxt* BGC is transcribed from two bidirectional promoter regions encoding five individual promoters. These promoters were identified experimentally using 5′ RACE and their activity assessed via coupling to a *lux* reporter system in *E. coli* and *Synechocystis* sp. PCC 6803. Transcription of the predicted drug/metabolite transporter (DMT) encoded by *sxtPER* was found to initiate from two promoters, P*sxtPER1* and P*sxtPER2*. In *E. coli*, strong expression of *lux* from P*sxtP*, P*sxtD* and P*sxtPER1* was observed while expression from P*orf24* and P*sxtPER2* was remarkably weaker. In contrast, heterologous expression in *Synechocystis* sp. PCC 6803 showed that expression of *lux* from P*sxtP*, P*sxtPER1*, and P*orf24* promoters was statistically higher compared to the non-promoter control, while P*sxtD* showed poor activity under the described conditions.

**Conclusions:**

Both of the heterologous hosts investigated in this study exhibited high expression levels from three of the five *sxt* promoters. These results indicate that the majority of the native *sxt* promoters appear active in different heterologous hosts, simplifying initial cloning efforts. Therefore, heterologous expression of the *sxt* BGC in either *E. coli* or *Synechocystis* could be a viable first option for producing PSTs for industrial or biomedical purposes.

## Background

Saxitoxin (STX) is a neurotoxin produced by cyanobacteria and dinoflagellates and is a member of the broader group of alkaloids known as the paralytic shellfish toxins (PSTs) [1]. When high concentrations of PSTs are consumed by humans, acute poisoning can lead to death due to respiratory paralysis [2–4]. Therefore, PSTs are needed as analytical standards for the monitoring and protection of commercial seafood and freshwater reservoirs, as well as for use in biomedical research. While the PSTs pose a significant public health risk and economic burden on society during algal bloom events, their scientific and pharmaceutical potential is well known [3, 4]. Purified PSTs have been a critical tool for researchers investigating neuronal sodium channels, where the toxins specifically block site 1 of voltage-gated sodium channels [5]. Under controlled administration, PSTs are potent anaesthetics, particularly in combination with other local anaesthetics [6, 7]. Further attempts to utilise STX in clinical trials are hindered by its toxicity, but more recent approaches, such as generating liposomal formulations of STX, resulted in blockage of the sciatic nerves in rats with no myotoxic, cytotoxic or neurotoxic effects [8]. It has been postulated that the same delivery could provide effective localised treatment for severe joint pain [9]. Other PSTs, such as the gonyautoxins (GTXs), also have clinical potential and have been utilised for the treatment of anal fissures and chronic tension type headaches [10–12].

Obtaining significant quantities of purified PSTs for clinical research or water quality analysis is difficult. Chemical synthesis and biocatalytic synthesis of PSTs is complex, difficult to scale up and may not produce all relevant naturally occurring isoforms [13, 14], The most common form of obtaining purified compounds involves extraction and isolation from dinoflagellate blooms, cyanobacterial cultures or contaminated shellfish coupled with synthetic conversion to additional PSTs [15–17]. Analytical calibration standards are commercially available from the National Research Council Canada (NRC), which are obtained from extractions of PST producing dinoflagellates or contaminated shellfish, and in some instances, semi-synthetic conversions of PSTs are required to obtain or broad array of analogues. Thus, the process is difficult, inefficient and costly [18]. These issues clearly highlight the need for an alternative and reliable method for the production and purification of commercial quantities of PSTs.

Heterologous expression of cyanobacterial biosynthetic gene clusters (BGCs) is not well established in comparison to heterologous expression of Actinomycete BGCs. *Streptomyces* expression hosts successfully produced more than 90 Actinomycete NPs, most of which are heterologously expressed using native promoters [19]. On the other hand, only 10 cyanobacterial NPs have been successfully produced from heterologous systems using both native and heterologous promoters [20], highlighting the need to better understand the function of native promoters in heterologous hosts. *E. coli* is a suitable host for the heterologous expression of cyanobacterial pathways based on its fast growth rate as previously demonstrated [21, 22]. Initial studies used native promoters to produce the ribosomal peptides patellamide A and C, and the microviridins [23, 24]. Recently, there has been a focus on the heterologous expression of cyanobacterial natural product BGCs including the lyngbyatoxin (*ltx*) BGC in *E. coli* using the tetracycline-inducible P*tet*_*O*_ promoter [21, 25]. The *ltx* BGC has been the focus of multiple heterologous expression studies due to its relatively small size. It has also been expressed in the cyanobacterium *Anabaena* sp. PCC 7120, and *E. coli* GB05-MtaA [26, 27]. While the native *ltx* promoters were active in *Anabaena* sp. PCC 7120 and drive the production of lyngbyatoxin A, the native promoters were not active in *E. coli.* The addition of *Anabaena* sp. PCC 7120 sigma factors to the *E. coli* host also failed to induce expression of lyngbyatoxin A, suggesting that the heterologous host was unable to recognise the cyanobacterial ribosome binding sites [28]. Subsequently, titres of lyngbyatoxin A close to the native producer were achieved when using the cyanobacterium *Anabaena* sp. PCC 7120 as a heterologous host [26]. This highlights that discrepancies between cyanobacterial promoter efficiencies in different host organisms remain poorly understood. Therefore, it is beneficial to test the activity of promoters in heterologous hosts and expression vectors using reporter systems prior to cloning complex biosynthetic pathways for biotechnological applications [29].

For the most part, cyanobacterial transcription machinery is similar to that found in *E. coli*, with the main difference being the widespread absence of the − 35 hexamer in cyanobacteria, which is believed to be replaced by a transcription factor binding site to initiate transcription [30]. In *E. coli*, σ^70^ is able to recognise the majority of promoters while in cyanobacteria, a range of different sigma factors have been identified [31, 32].

The saxitoxin (*sxt*) BGC has been characterised in six cyanobacterial species from the order Nostocales and one from the order Oscillatoriales [33–37]. Each *sxt* BGC encodes a ‘core’ set of enzymes putatively responsible for STX biosynthesis, supplemented with ‘tailoring’ and ‘auxiliary’ genes that give rise to PST analogues or perform functions after PST biosynthesis. Information regarding the regulation of transcriptional elements of cyanobacterial secondary metabolite biosynthesis remains limited to the microcystin (*mcy*) and jamaicamide (*jam*) BGCs [38–43]. However, the regulation of PSTs at the molecular level, including the transcriptional elements of the *sxt* BGC remains largely unknown [44, 45].

Here, we identify the transcription units of the *sxt* BGC within the cyanobacterium *Dolichospermum circinale* AWQC131C which enabled the experimental isolation of five promoter regions. We then assessed the reliability of a luciferase reporter (*lux*) system to assay the activity of uncharacterised cyanobacterial promoters in the heterologous hosts *E. coli* and *Synechocystis* sp. PCC 6803 for the first time. Characterisation of these cyanobacterial promoters and determination of their activity in *E. coli* and *Synechocystis*, highlights the unpredictability of cyanobacterial promoters of natural product BGCs in heterologous hosts. This study is the first essential phase of understanding the cloning strategy expression of PST biosynthesis, identifying the need for promoter engineering or exchange in future experiments.

## Results

### Identification of transcriptional units within the *sxt* biosynthetic gene cluster

**Fig. 1:**
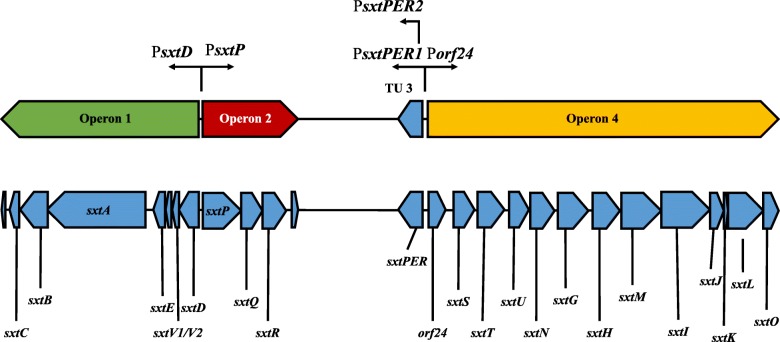
Transcriptional analysis of the *D. circinale* AWQC131C *sxt* cluster. Genes required for PST biosynthesis are transcribed by a minimum of four mRNA transcripts from two bi-directional promoter regions encoding five promoters; P*sxtD* (operon 1), P*sxtP* (operon 2), P*sxtPER1* (TU 3), P*sxtPER2* (TU 3) and P*orf24* (operon 4). Direction of transcription is indicated by black arrows

Reverse-transcriptase PCR revealed that the *sxt* BGC in *D. circinale* AWQC131C is transcribed as five transcriptional units from two bidirectional promoter regions (Fig. 1; Additional file 1: Figure S1). All five transcripts appear to be constitutively expressed under standard laboratory conditions, since *sxt* mRNA was detected across all time points. Operon 1, sxtDV*EABC (* indicates disrupted ORF of *sxtV* [34]), spans 7.3 kb, is transcribed in the reverse direction and encodes several proteins predicted to be involved early in PST biosynthesis. Operon 2, *sxtPQR*, spans 3.5 kb and is transcribed in the forward direction. The catalytic functions of SxtP, SxtQ and SxtR are unknown, but they are likely to be essential for PST biosynthesis as their presence and organisation is conserved amongst all reported *sxt* clusters. The third transcriptional unit is monocistronic and encodes SxtPER, a putative permease of the drug/metabolite transporter family of proteins and is transcribed from two promoters, as discussed further below. Operon 4, is transcribed in the forward direction and spans 12.8 kb. Operon 4 encodes a protein of unknown function, Orf24 that is conserved in most *sxt* clusters, followed by genes encoding 12 enzymes involved in PST biosynthesis, resulting in the polycistron *orf24sxtSTUNGHMIJKLO*.

The 3′ ends of operons 1–4 were bioinformatically screened for putative Rho-dependent and Rho-independent transcriptional termination sites using the programs TransTerm and TranstermHP, respectively [46, 47]. Rho-independent transcription termination sites were identified in the non-coding regions of three out of four *sxt* mRNA transcripts (Additional file 1: Table S1). Rho-dependent or Rho-independent termination sites were not identified in the sequence of mRNA encoding operon 1.

### Transcription start sites and promoter regions of the *sxt* operons

**Fig. 2:**
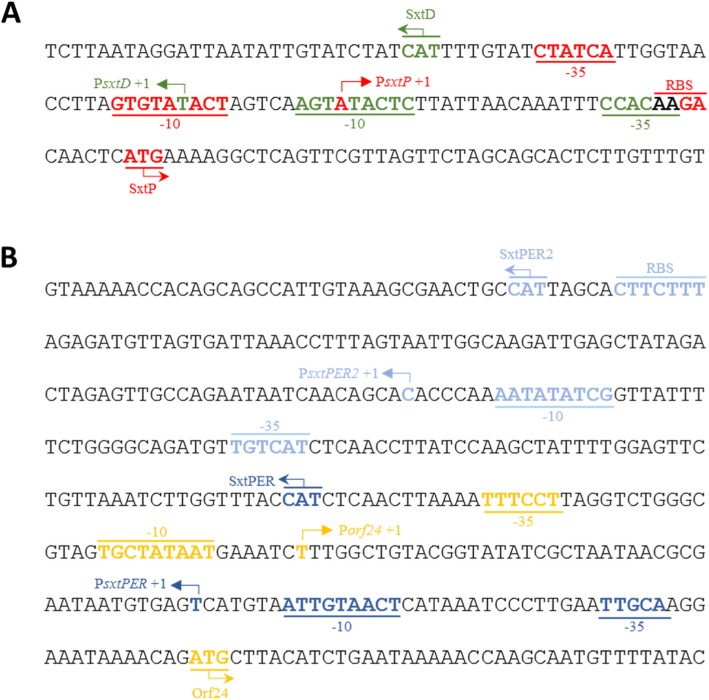
Sequence analysis of the five promoters present within the *D. circinale* AWQC131C *sxt* biosynthetic gene cluster. **a** Intergenic between *sxtD *and *sxtP *showing the bidirectional promoter region of operon 1 and 2. **b** Intergenic between *sxtPER *and *orf24 *showing the bidirectional promoter region of operon 3 and 4. Individual promoters include, P*sxtD* (green), P*sxtP* (red), P*sxtPER2* (light blue), P*sxtPER1* (dark blue) and P*orf24* (yellow). Transcriptional elements including promoters, TSS, translation start site, and RBS are shown for the five *sxt* promoters

The transcriptional start sites (TSSs) of each operon were experimentally identified via 5′ rapid amplification of cDNA ends (5′ RACE) (Table 1; Additional file 1: Figure S2). The upstream region of each TSS was screened for a promoter sequence consistent with conserved binding sequences of group 1, 2 and 3 sigma factors [31]. All promoters identified in this study displayed sequence similarity to the consensus − 10 hexamer (Pribnow box) of the prokaryotic RNA polymerase binding site, while there was sporadic presence of the − 35 hexamer binding site (Table 1). These results suggest that the *sxt* promoters of *D. circinale* AWQC131C are activated by an RNA polymerase core enzyme in conjunction with a group 1 or group 2 sigma factor [31, 48]. For the − 10 promoter sequences identified, a search was conducted for an extended − 10 binding site and upstream (UP) element. The 5′ untranslated region (UTR) of each operon was also bioinformatically screened for the presence of consensus ribosomal binding site (RBS) sequences, although previously reported bioinformatics surveys of cyanobacterial genomes were not able to identify the consensus RBS sequence in all genes [49, 50]. Based on the 5′ RACE and bioinformatics data, the *D. circinale* AWQC131C *sxt* BGC includes of a total of five TSSs under standard culture conditions (Fig. 2).

**Table 1:** Characteristics of promoter regions in the *sxt* biosynthetic gene cluster of *D. circinale* AWQC131C

Promoter	Promoter sequence 5′ ➔ 3′	Position^Δ^
P*sxtD*	tgtc**TTG**TGG....(14 bp).....GAg**TATA**C**T**tgactagt**A**	−32
P*sxtP*	gtatC**T**AT**CA**....(12 bp).....GTg**TATA**C**T**agtcaagt**A**	−34
P*sxtPER1*	ttcc**TTG**CA**A**....(15 bp).....A**G**t**TA**C**AAT**tacatg**A**	−91
P*sxtPER2*	tgagA**TGACA**....(21 bp).....C**G**a**TATA**T**T**ttgggt**G**	+ 94
P*orf24*	aaaa**TT**TC**C**T....(15 bp).....**TG**c**TATAAT**gaaatc**T**	−160
*E. coli* σ^70^ consensus	....**TTGACA**....(14 bp).....**TGnTATAAT......N**	

The −10 and − 35 hexamers are capitalised and conserved nucleotides are in bold print. N indicates transcriptional start site (TSS). ^Δ^ Position of promoter relative to TSS

Operon 1 (sxtDV*EABC) contains a short 5′ UTR of − 32 bp upstream of the translation start site and a promoter (P*sxtD*) with high sequence similarity to the *E. coli* σ^70^–10 and − 35 hexamers. P*sxtP* initiates the transcription of operon 2, possesses a short 5′ UTR spanning 34 bp and contains both − 10 and − 35 regions. The transcript initiated by P*sxtP* also displayed a likely RBS (AAGA) 6 nucleotides upstream of the *sxtP* translation start site. A conserved − 35 sequence was also identified 21 bp upstream of the extended − 10 sequence, resulting in an unusually long distance between the two hexamers. P*orf24* has a perfectly conserved − 10 consensus sequence, including the extended − 10 TGn motif (Table 1). The 5′ UTR for *orf24* is 160 bp in length.

Unusually, transcription of the putative transporter, *sxtPER,* was initiated from two promoters, P*sxtPER1* and P*sxtPER2*. P*sxtPER1* is located 91 bp upstream of the annotated TSS of *sxtPER* (Fig. 2) and contains a highly conserved − 10 and − 35 RNA polymerase binding site. P*sxtPER2* is located 94 bp downstream of the translational start site and contains a highly conserved − 10 sequence, including the single nucleotide seen in extended − 10 promoters as well as an RBS (AAAGAAG).

### The activity of *sxt* promoters in *E. coli*

The five promoters identified in the *D. circinale sxt* cluster using 5′ RACE, P*sxtP*, P*sxtD*, P*sxtPER1*, P*sxtPER2* and P*orf24*, were amplified by PCR and cloned into the *E. coli* expression vector, pET28b (Novagen), directly in front of a *lux* operon (Additional file 1: Figure S4). Expression of luciferase from each of these promoters was measured and compared with negative controls; *pET28-lux* harbouring a non-promoter region from within the *sxtO* gene and the pET28-*lux* plasmid with no added promoter. Unpaired t-tests showed that all promoters exhibited significant levels of expression (Additional file 1: Table S2) when compared to the pET28-*lux* negative control. Under the described culture conditions, the heterologous P*sxtD,* P*sxtP*, and P*sxtPER1* promoters mediated the highest levels of luciferase expression in *E. coli* (Additional file 1: Table S3). There was a statistically significant difference (*p* < 0.0001) between the highest performing promoter P*sxtD* and all the other promoters, as well as the controls (*sxtO* and pET28-*lux*) (Additional file 1: Table S4).

**Fig. 3:**
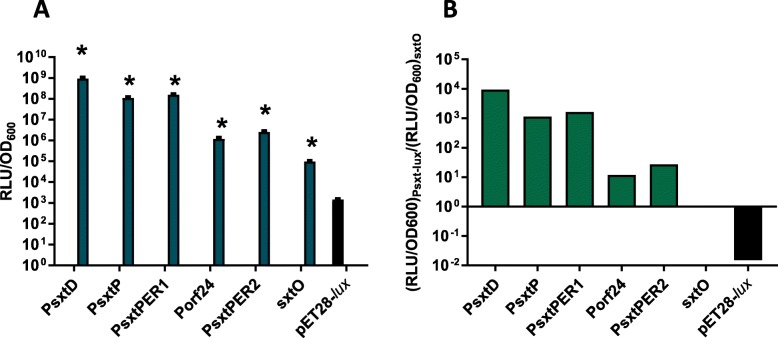
Heterologous expression from of luciferase from *sxt* promoters in *E. coli* DH5α. **a** Luciferase expression was normalised to the optical density, OD_600_. The activity of five promoters was tested: P*sxtD*, P*sxtP*, P*sxtPER1*, P*orf24*, and P*sxtPER2*. An intergenic *sxtO* sequence was used as the non-promoter control. The highest RLU/OD for the *E. coli* DH5α strains expressing *luxCDABE*. (*) Denotes statistically significant differences (*p* < 0.0001) between the *E. coli* DH5α strains and the pET28-*lux* control in unpaired t-tests. **b** Expression relative to the *sxtO*-*lux* control. The mean normalised luciferase expression for each promoter was divided by the mean normalised expression of *sxtO*. The three strongest promoters (P*sxtD*, P*sxtP*, P*sxtPER*) exhibit between 1000 and 9500 fold higher levels of luciferase expression over the *sxtO* control. The P*orf24* and P*sxtPER2* promoters had only a 12 and 27-fold increase in expression, respectively

The promoter responsible for the transcription of *orf24* and the second promoter of *sxtPER, PsxtPER2* were weaker than the other promoters, but still significantly stronger than the controls (Fig. 3a). The incorporation of both promoters into the *lux* expression constructs resulted in a 12–27-fold increase in luciferase expression over *sxtO*-*lux* (Fig. 3b), and 810–1770-fold increase in luciferase expression over the pET28-*lux* control. These results indicate that the promoters are active, albeit weaker than the other three promoters.

### The activity of *sxt* promoters in *Synechocystis* sp. PCC 6803

**Fig. 4:**
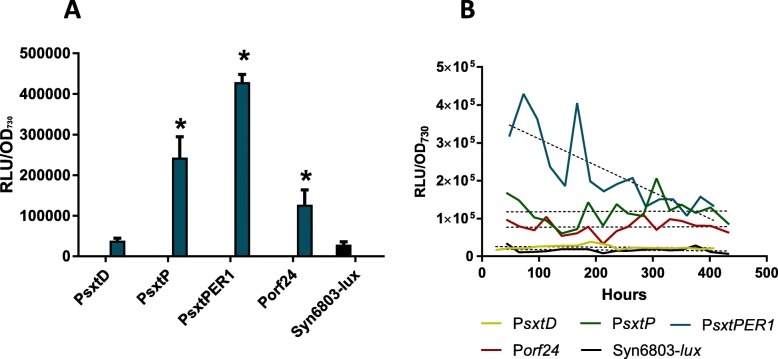
Heterologous expression of luciferase from *sxt* promoters in *Synechocystis* sp. PCC 6803. **a** Luciferase expression from the four main *sxt* promoters in *Synechocystis* sp. PCC 6803. The highest RLU/OD for the *Synechocystis* sp.PCC 6803 strains expressing *luxCDABE*. (*) Denotes statistically significant differences (*p* < 0.05) between the *Synechocystis* sp. PCC 6803 strains and the *Synechcocystis* sp. PCC 6803-*lx* control in unpaired t-tests. **b** Normalised luciferase expression over time in *Synechocystis* PCC6803. RLU/OD_730_ over 400 h of growth. P*sxtD* normalised expression was low. P*sxtP* and P*orf24* had consistent levels of luciferase expression. P*sxtPER* showed high initial expression that declined over growth to expression reach levels similar to P*sxtP* and P*orf24*

Four *sxt* promoters were active in *Synechocystis* sp. PCC 6803 (Fig. 4). Unpaired t-tests showed that expression of luciferase from the P*sxtP*, P*sxtPER1*, and P*orf24* were significantly different to expression in the control strain, while expression from P*sxtD* was not statistically different to the control strain (*P* < 0.05; Additional file 1: Table S5).

The *sxtD* promoter regulates the transcription of operon 1 of the *D. circinale sxt* cluster, which carries the core biosynthetic genes, including the polyketide synthase-like enzyme, *sxtA*. Strains harbouring P*sxtD* had very low luciferase expression levels that were only 1.3-fold higher than expression levels in the promoter-less control strain (Additional file 1: Table S6), and were statistically lower than the other 3 promoters (Additional file 1: Table S7). The lack of statistically significant expression from P*sxtD* indicates the promoter as the only candidate for exchange for heterologous expression of PSTs in *Synechocystis* sp. PCC 6803. P*sxtP* and P*orf24* mediated consistent levels of luciferase expression per OD_730_ throughout the experiment (Fig. 4b) P*sxtPER1* mediated expression levels that were initially up to three-fold higher than P*sxtP*, however, the rate of expression decreased over the course of growth.

## Discussion

Information regarding the transcriptional elements of cyanobacterial secondary metabolite biosynthesis is an essential first step in both understanding how these metabolites may be regulated within the native producers as well as harnessing these genes for future heterologous expression experiments. Here, we targeted the transcriptional units of the *D. circinale* AWQC131C *sxt* BGC and measured promoter activity within two possible future heterologous hosts, *E. coli* and *Synechocystis*.

The entire *D. circinale* AWQC131C *sxt* cluster was found to be transcribed on a total of five transcriptional units from two bidirectional promoter sites. The isolated promoters all contained a − 10 Pribnow box, as expected, but sporadically contained other transcriptional elements including the − 35 hexamer, UP element and RBS, which is commonly observed in cyanobacteria [30, 49, 50]. A further feature was the identification of a second promoter region initiating transcription of the proposed permease SxtPER. One of these included an intragenic promoter region, which results in the expression of a 257 aa truncated isoform of SxtPER. While uncommon, the use of a second TSS to produce two protein isoforms has previously been reported [51]. For example, the bacterocin colicin V is secreted by a membrane transporter, where both the full length CvaA and shorter CvaA* proteins, translated from the same *cvaA* gene, are both required for optimal excretion [52]. Interestingly, BLASTp analysis revealed that a complete RhaT super domain of the drug/metabolite transporter family [53] is present within both isoforms of the protein. The larger encoded protein contains additional sequence at the N-terminus. While our previous proteomics experiments in *D. circinale* were unable to detect both isoforms of SxtPER protein under standard conditions, further experiments are required to determine if the two isoforms of the proteins are required for excretion of PST in *D. circinale*.

The expression of P*sxtP* is an interesting example of the promoter elements required for heterologous expression of cyanobacterial promoters in *E. coli*. P*sxtP* does not seem to have a discernible − 35 binding region yet does have a RBS and promoted high expression levels in *E. coli*. Previous studies have shown that while the distance between the − 10 and − 35 sequences can effect transcription in cyanobacteria, the − 35 hexamer is not always required [54–56]. Thus, the competing preferences between the TSS sequence and position, taken together with other elements of the promoter such as − 10, and − 35 sequences, transcription factors, the sequence length between the − 10 and − 35 regions, and the RBS, highlights the complexity of transcriptional regulation and shows the importance of experimental validation of promoter activation data to further improve bioinformatics databases.

It was found that he expression levels of PsxtPER1 initiated at a high level but decreased over the course of growth. This indicates the majority of toxins could be exported from the cell early in culture and retained in the cell as the culture progresses. This would allow future research to optimise PST extraction at different culture stages, either from the cell free component or the cell mass. Alternatively, since P*sxtPER1* is active at the early growth stages within the heterologous host, it could be a target for repression to limit toxin export and therefore retaining toxin within the cell. This will increase the efficiency of toxin isolation from the cell biomass.

It is known that gene expression levels will have significant impact on the amount of PST molecule produced. Here, the promoters that regulate PST biosynthesis mediated lowered expression of luciferase in *Synechocystis* sp. PCC 6803 compared to *E. coli*. The significant decrease in luciferase expression by the cyanobacterial promoters in *Synechocystis* sp. PCC 6803 has previously been observed in studies of the zinc inducible promoter, P*smt,* from *Synechococcus* sp. PCC7002. P*smt* mediated higher levels of protein synthesis and therefore higher levels of ethylene production in *E. coli,* compared to *Synechocystis* sp. PCC 6803, which only produced residual levels [57]. Conversely, high expression levels impacted lynbyatoxin biosynthesis in *E. coli*. Heterologous expression of lyngbyatoxin (*ltxA-D*) in *E. coli* was only successful when the strong T7 phage promoter was replaced with the weaker P*tet*_*O*_ promoter [27]. P*tet*_*O*_ has since been exploited for the heterologous expression of multiple cyanobacterial BGCs in *E. coli* [21, 22, 25]. Subsequent heterologous expression of lyngbyatoxin in *Anabaena* using non-native promoters was more successful than the *E. coli* system while expression from the native promoter did not occur in either case [23]. Together, these results suggest that native promoters are recognised differently in heterologous hosts and that while successful transcription of cyanobacterial BGCs in heterologous hosts is important, other factors play a role in the efficiency of the host production of secondary metabolites. This study has identified each of the five native *sxt* promoters and established through the use of the *lux* reporter, which of those were recognised in both *E. coli* and *Synechocystis*.

## Conclusion

PSTs have a range of biomedical applications and thus heterologous expression of the *sxt* BGC should be explored as a potential tool for the characterisation, manipulation and sustainable production of these compounds. Heterologous expression of cyanobacterial natural product BGCs has had mixed success in the past and further characterisation of cyanobacterial promoters is required for successful expression of complex biosynthetic pathways such as the PST biosynthetic pathway. This study identified five putative *sxt* promoters in *D. circinale* AWQC131C and tested their activity in *E. coli* and *Synechocystis* sp. PCC 6803. In *E. coli*, P*sxtD*, P*sxtP* and P*sxtPER1* promoted luciferase expression while P*orf24* was significantly weaker. Further, if *Synechocystis* sp. PCC6803 is to be used as a host, the inactive P*sxtD* should be replaced by host-compatible promoters. Successfully manipulating the *sxt* BGC within a heterologous host at the transcription level is the first step to efficiently access the PSTs for a range of biotechnological applications.

## Methods

### Strains and culture conditions

*D. circinale* AWQC131C was maintained in Jaworski’s medium (JM) [58] at 24 °C ± 1 °C and illuminated with 11 μmol m^− 2^ s^− 1^ of photons on a 12:12 h light/dark cycle. *Synechocystis* sp. PCC 6803 was maintained in BG11 medium supplemented with 100 μg mL^− 1^ spectinomycin when required, at 30 °C under constant illumination. Unless otherwise specified, *E. coli* strains (Table 2) were maintained in Luria broth or on agar plates supplemented with 100 μg mL^− 1^ ampicillin or 50 μg mL^− 1^ kanamycin and grown at 37 °C.

**Table 2:** Strains and plasmids

Organism	Purpose	Ref.
DH5α	Expression host of *lux* reporter assay	Promega
TOP 10	Transformation and propagation of plasmids	Invitrogen
*D. circinale* AWQC131C	Organism harbouring native *sxt* cluster	[34]
*Synechocystis* sp. PCC6803	Expression host of *lux* reporter assay	
*Synechocystis* SXL7	Δ*phbC*:: P*sxtD*-*luxCDABE* - Spec^R^	This study
*Synechocystis* SXL8	Δ*phbC*:: P*sxtP*-*luxCDABE* - Spec^R^	This study
*Synechocystis* SXL9	Δ*phbC*:: P*sxtPER1*-*luxCDABE* - Spec^R^	This study
*Synechocystis* SXL10	Δ*phbC*:: P*orf24*-*luxCDABE* - Spec^R^	This study
*Synechocystis* SXL11	Δ*phbC*:: *luxCDABE* - Spec^R^	This study
Plasmids	Purpose	Ref.
pCR™2.1 TOPO	Cloning and sequencing of 5′ RACE TSS products	Invitrogen
pGEM T-Easy	Cloning and sequencing of 5′ RACE TSS products	Promega
pET28b	Cloning and expression of *sxt* promoters	Novagen
pSYN_6.3	Cloning and transformation of *sxt* promoters in *Synehcocystis* sp. PCC 6803	Unpublished
pLUX NS II	*luxCDABE*	[59]
pSXL1	pET28b::P*sxtD-luxCDABE*	This study
pSXL2	pET28b::P*sxtP-luxCDABE*	This study
pSXL3	pET28b::P*sxtPER1-luxCDABE*	This study
pSXL4	pET28b::P*orf24*-*luxCDABE*	This study
pSXL5	pET28b::P*sxtPER2*-*luxCDABE*	This study
pSXL6	pET28-*sxtO-luxCDABE*	This study
pET28-*lux*	pET28-*luxCDABE*	This study
pSXL7	pSYN_6.3::PsxtD- *luxCDABE*	This study
pSXL8	pSYN_6.3::PsxtP- *luxCDABE*	This study
pSXL9	pSYN_6.3::PsxtPER1- *luxCDABE*	This study
pSXL10	pSYN_6.3::Porf24- *luxCDABE*	This study
pSXL11	pSYN_6.3::*luxCDABE*	This study

### Total RNA extraction, cDNA synthesis and transcriptional analysis

To extract high quality total RNA, cell pellets were snap-frozen in liquid nitrogen and ground into a fine powder with a mortar and pestle prior to extraction with the RNeasy Plant Mini kit (QIAGEN). Residual genomic (g) DNA was removed from total RNA samples using TURBO DNA-*free*™ DNase as described by the manufacturer (Ambion). Removal of contaminating gDNA was confirmed via PCR with the 27F/809R PCR primer set targeting the cyanobacterial 16S rRNA gene [60]. RNA quality was also checked via formaldehyde gel electrophoresis, while gDNA was checked by agarose gel electrophoresis.

The Superscript® III First Strand synthesis system (Invitrogen) was used to reverse transcribe 1 μg of total RNA primed with an antisense gene-specific primer (GSP). Transcriptional units were determined by PCR amplification in a 20 μL reaction mixture containing 2.5 mM MgCl_2_, 1 × PCR buffer (Fisher Biotec, Geneworks), 10 pmol dNTPs (Austral Scientific), 10 pmol of GSP, 1 U of *Taq* polymerase (Fisher Biotec, Geneworks) and sterile Milli-Q water. Thermal cycling was performed in a Bio- Rad 96-well iCycler (Bio-Rad) and began with an initial denaturation cycle of 95 °C for 4 min, followed by 35 cycles of DNA denaturation at 95 °C for 20 s and primer annealing at 55 °C for 20 s. DNA strand extension was altered to 1 min for every 1 kb of amplified product. A final extension at 72 °C for 7 min and a final holding temperature of 4 °C completed the thermal cycling. Each reaction contained cDNA as the template and two primers (Additional file 1: Table S8), which were designed to target an adjacent gene. Amplification was observed if the two adjacent genes were located on the same mRNA transcript. The positive control for each PCR contained gDNA. Two negative control reactions were performed by adding template from a cDNA synthesis reaction, the first omitting reverse transcriptase and the second reaction omitting a nucleic acid template.

### Isolation of *D. circinale* AWQC131C *sxt* biosynthetic gene cluster transcription start sites (TSS) and promoters using 5′ rapid amplification of cDNA ends (5′RACE)

To isolate the promoter of each transcriptional unit, TSSs were localised with the FirstChoice® RLM-RACE kit for 5′ RACE (Ambion) with 10 μg total RNA as starting material. The 5′ RACE adapter was ligated directly onto RNA followed by reverse transcription cDNA synthesis. First round PCR reactions were performed using a 5′ outer adapter primer in conjunction with four reverse GSPs at approximately 50–100 bp intervals (Additional file 1: Figure S3, Table S9). Reactions containing amplified products from the first round PCR became the template for second round nested PCR containing a 5′ adapter inner primer in conjunction with the same four reverse primers. Amplicons of interest were analysed on a 2% (w/v) agarose gel and purified using a QIAquick spin gel extraction kit (QIAGEN). Purified PCR products were then cloned into the pGEM-T Easy vector (Promega) and sequenced using an ABI 3730 capillary sequencer at the Ramaciotti Centre for Genomics, UNSW.

### Cloning and transformation

The TOPO TA cloning® kit (Invitrogen) and the pGEM®-T Easy Vector kit (Promega) were used for the cloning and transformation of *E. coli* (Table 2). Cloning with the TOPO TA cloning® kit involved setting up a ligation reaction containing 4 μL of PCR product, 1 μL of Invitrogen salt solution (1.2 M NaCl, 0.06 M MgCl_2_) and 10 ng pCR®2.1-TOPO® plasmid DNA (Invitrogen). The ligation reaction was incubated for 20 min at room temperature and was then ready for transformation. The pGEM**®**-T Easy vector ligation reaction contained 1 × rapid ligation buffer (Promega), 50 ng of pGEM**®**-T Easy vector DNA (Promega), 3 Weiss U of T4 DNA ligase (Promega) and 3 μL of PCR product. The ligation reaction was left to incubate overnight at 4 °C and was then ready for transformation. Positive transformants were selected by blue and white colony screening and the presence of a cloned insert was confirmed by colony PCR using either the primer sets M13F and M13R (pCR®2.1-TOPO) or T7F and M13R (pGEM-T Easy). Plasmids containing an insert were then sequenced.

### Engineering the *sxt* promoter-luciferase reporter constructs for expression in *E. coli* DH5α

Five promoters (P*sxtD*, P*sxtP*, P*sxtPER1*, P*orf24*, P*sxtPER2*) and a non-promoter region within the *sxtO* open reading frame were cloned into the pET28b expression vector along with the luciferase reporter (*luxCDABE*) operon from *Photorhabdus luminescens* (Additional file 1: Figure S4A). The luciferase operon (*luxCDABE*) was amplified from the pLuxNSII plasmid [59] via PCR (denaturation at 98 °C for 3 min, followed by 30 cycles of denaturation at 98 °C for 15 s, annealing step at 60 °C for 20s, extension at 72 °C for 30s/kb, and a final extension at 72 °C for 10 min), and the pET28b backbone was also PCR amplified to remove the T7 promoter region. All primers were designed using the NEBuilder assembly tool (Additional file 1: Table S10). Double-stranded PCR fragments were amplified using the KAPA HiFi Hotstart DNA polymerase (KAPA biosystems). The pET28b backbone, *sxt* promoter (*Psxt)*, and *lux* operon were assembled using the Gibson assembly master mix (NEB) [61], and incubated at 50 °C, for 1 h. The reaction was transformed into chemically competent *E. coli* DH5α and positive colonies selected as above.

### Engineering the *sxt* promoter-luciferase reporter constructs for expression in *Synechocystis* sp. PCC 6803

The *Psxt-lux* integration vector was engineered through classical restriction/ligation cloning using the restriction enzymes *Not*I and *Kpn*I (NEB). The P*sxt*-*lux* fragments were amplified from the pET28b-P-*lux* vectors using the lacI-P-lux_NotI_F, and lacI-P-lux_KpnI_R primers (Additional file 1: Table S10). Linear DNA fragments were digested, purified and ligated into the pSYN_6.3 vector (Additional file 1: Figure S4B) using T4 DNA ligase at 22 °C for 1 h, followed by transformation into *E. coli* DH5α and colony screening. Plasmid constructs were confirmed by terminal-end sequencing.

The integration of the P*sxt*-*lux* fragments into the *Synechocystis* sp. PCC 6803 genome (Additional file 1: Figure S4C) was achieved via the natural competence of the host [62]. *Synechocystis* sp. PCC 6803 was grown at 30 °C, with shaking at 100 rpm, under constant light until exponential phase, and used to inoculate a 50 mL BG11 medium to an initial OD_730_ of 0.05. After ~ 4 days of photoautotrophic growth, the cells reached an OD_730_ of 0.5, and were harvested by centrifugation at 2750 *g* for 5 min. Cells were resuspended in 2 mL of fresh BG11 medium, divided into 0.5 mL aliquots (OD_730_ of 2.5), combined with 10 μg of DNA and incubated at 30 °C for 6 h. A sterile Immobilon Transfer membrane (Merk Millipore) was placed on each BG11 agar plate, overlaid with 200 μL of the transformation mixture and incubated for 12 h under constant illumination at 30 °C. Membranes were transferred to BG11 agar plates containing 25 μg mL^− 1^ spectinomycin. The plates were incubated for a further 2 days at 30 °C under constant illumination, before the membrane was transferred to plates containing 50 μg mL^− 1^ spectinomycin and incubated for a further 7–10 days until colonies become visible. Recombinant *Synechocystis* sp. PCC 6803 colonies were picked and streaked onto BG11 agar plates supplemented with 100 μg mL^− 1^ spectinomycin and subcultured a further three times to achieve integration of the cloned reporter fragment and full chromosomal segregation. Transformants were confirmed using the PhaCaF and PhaCbR PCR primers (Additional file 1: Table S10).

### Activity of *sxt* promoters in *E. coli* DH5α

Promoter-luciferase reporter constructs were transformed into *E. coli* DH5α and grown on M9 minimal medium supplemented with 50 μg mL^− 1^ kanamycin at 37 °C for 24 h. Bioluminescence (RLU) and optical density measurements were measured at one-hour intervals until the OD_600_ reached 0.8. A final measurement was taken at 24 h. The strength of each promoter was measured as the highest bioluminescence, normalised to OD_600_. One-way ANOVA (Graphpad Prism 7) was used to calculate any statistical differences between the promoters. Unpaired t-tests were also used to determine the statistical differences between the strains and the control.

### Activity of *sxt* promoters in *Synechocystis* sp. PCC 6803

Promoter-luciferase reporter constructs were transformed into *Synechocystis* sp. PCC 6803 strains, which were inoculated into BG11 medium supplemented with 100 μg mL^− 1^ of spectinomycin and grown at 30 °C with shaking under constant illumination. Optical density and relative light units (RLU) were measured every 24 h for 400 h. Promoter strength was measured by determining the highest RLU per OD_730._ One-way ANOVA (Graphpad Prism 7) was used to calculate any statistical differences between the promoters. Unpaired t-tests were also used to determine the statistical differences between the strains and the control.

## Supplementary information

**Additional file 1.** Contains supplimentary data including results for transcript analysis and TSS sequencing, nested PCR to determine TSS, cloning strategy, transcriptional terminator prediction, statistical analysis, primer sequences, as well as all vector sequences with corresponding vector maps.

## Data Availability

All data generated or analysed during this study are included in this published article and its supplementary information files. Sequences of plasmids used in this study are available at the end of the supplementary information file.

## Abbreviations

5′ RACE: Rapid amplification of cDNA ends
BGC: Biosynthetic gene cluster
GSP: Gene specific primer
PST: Paralytic shellfish toxins
RBS: Ribosomal binding site
TSS: Transcriptional start site
UP: Upstream promoter element
UTR: Untranslated region
