# Supplementary material for: Identification of promoter elements in the Dolichospermum circinale AWQC131C saxitoxin gene cluster and the experimental analysis of their use for heterologous expression
Source: BMC Microbiol. 2020 Feb 17;20:35. doi: 10.1186/s12866-020-1720-3 (PMC7027233; doi:10.1186/s12866-020-1720-3)
Supplement: Supplementary file 1 — Additional file 1. Contains supplimentary data including results for transcript analysis and TSS sequencing, nested PCR to determine TSS, cloning strategy, transcriptional terminator prediction, statistical analysis, primer sequences, as well as all vector sequences with corresponding vector maps. [file 12866_2020_1720_MOESM1_ESM.docx]

**Identification of promoter elements in the *Dolichospermum circinale* AWQC131C saxitoxin gene cluster and the experimental analysis of their use for heterologous expression**

*Paul M. D’Agostino^1,2,3, a^, Bakir Al-Sinawi^2, a^, Rabia Mazmouz^2^, Julia Muenchhoff^2,4^, Brett A. Neilan^2,5*^, Michelle C. Moffitt ^1^**

^1^School of Science and Health, Western Sydney University, Sydney, NSW, Australia

^2^School of Biotechnology and Biomolecular Sciences, University of New South Wales, Sydney, NSW, Australia

^3^Biosystems Chemistry, Department of Chemistry, Technische Universität München, Garching, Germany

^4^Centre for Healthy Brain Ageing, School of Psychiatry, University of New South Wales, Sydney, Australia

^5^School of Environmental and Life Sciences, University of Newcastle, Callaghan, Australia

^a^Authors contributed equally

**SUPPORTING INFORMATION**


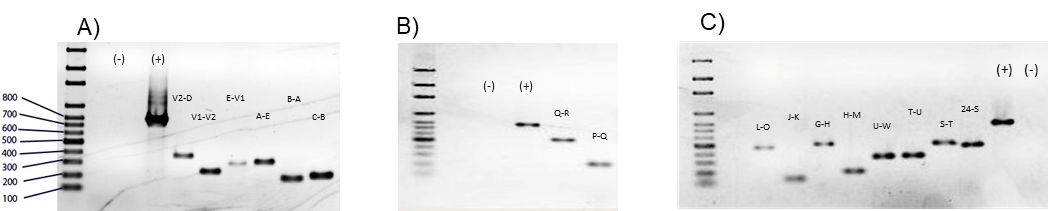


**Figure S1. Agarose gel electrophoresis of transcript analysis PCR products.** A negative control (-) included a PCR reaction devoid of template and a positive control (+) used template obtained from cDNA synthesis primed with random hexamide primers and amplification of the cyanobacterial 16S. Each cDNA was generated with a gene specific primer targeting the last gene (3ʹ-end) of each transcript. A) Amplicons obtained from transcript 1. B) Amplicons obtained from transcript 2. C) Amplicons obtained from transcript 4.


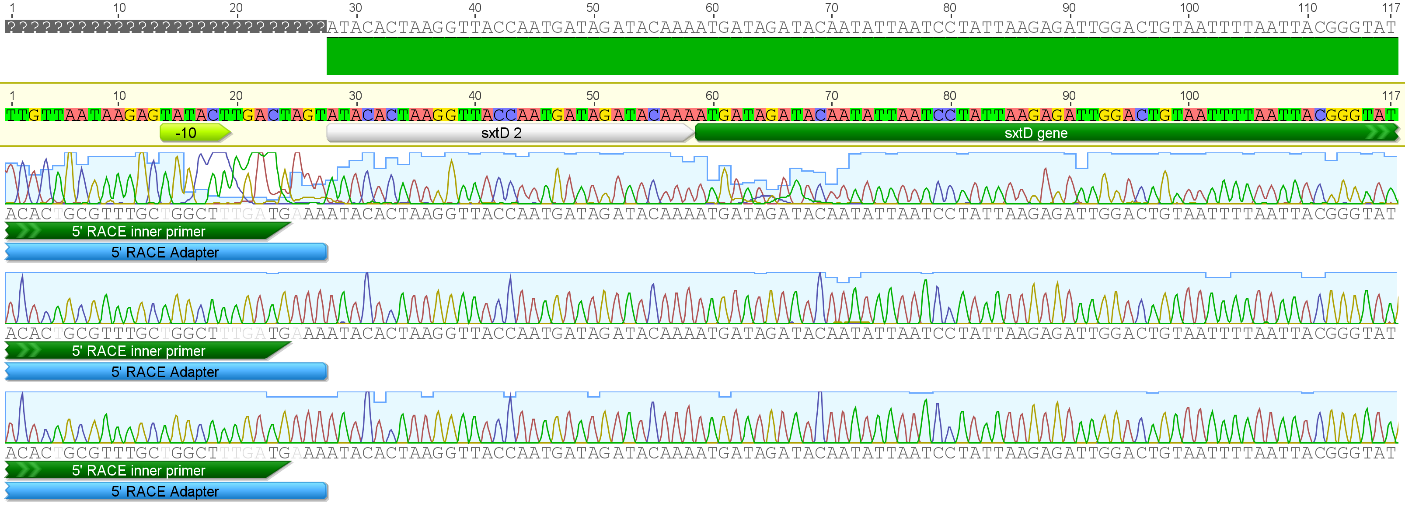
**Transcription start point of P*sxtD***

**
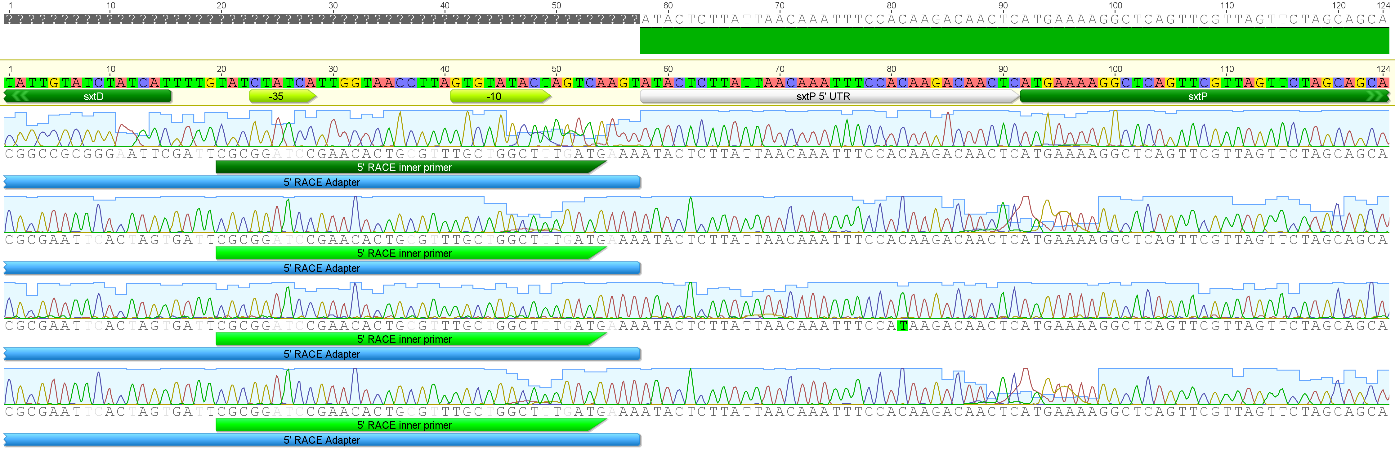
Transcription start point of P*sxtP***

**Transcription start point of P*sxtPER2***


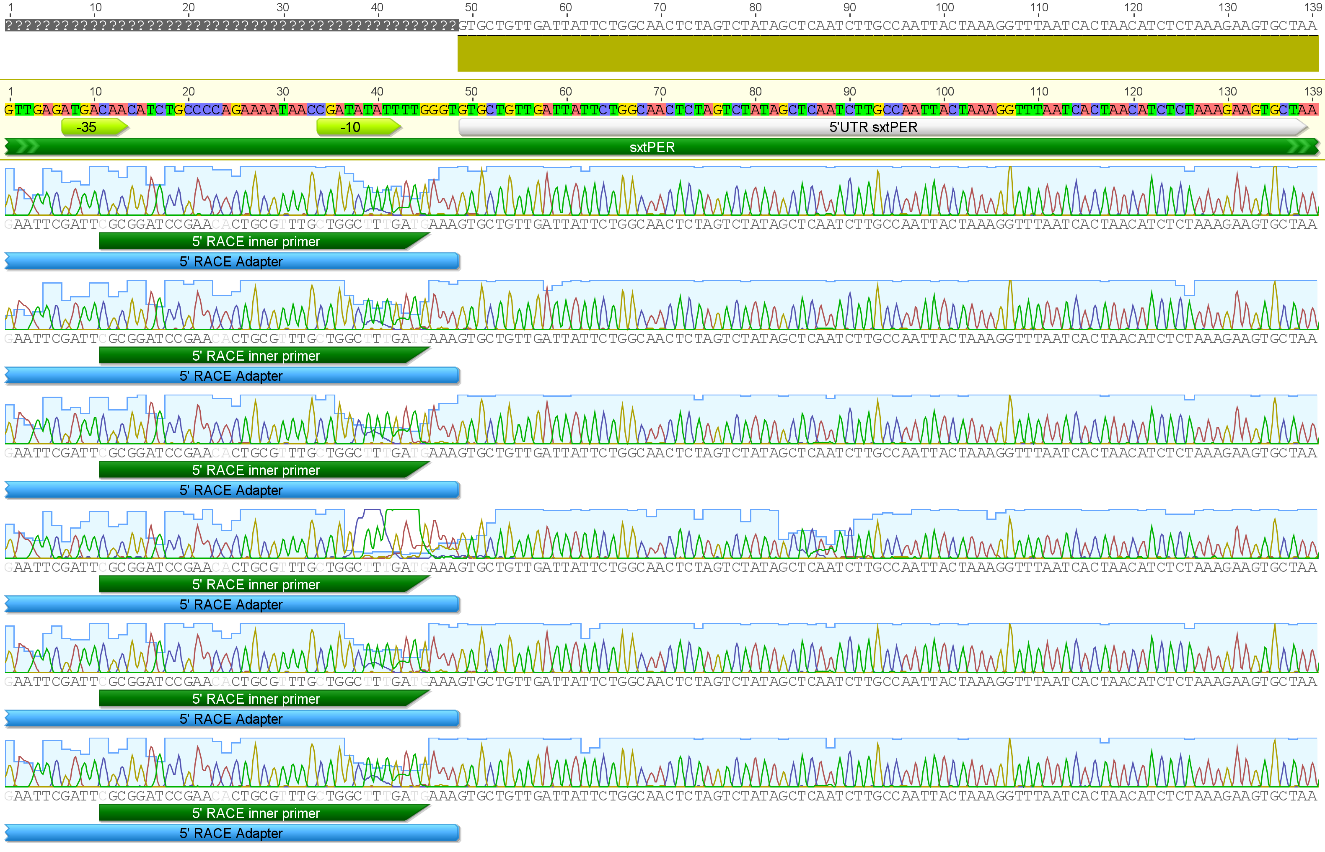


**Transcription start point of *sxtPER***


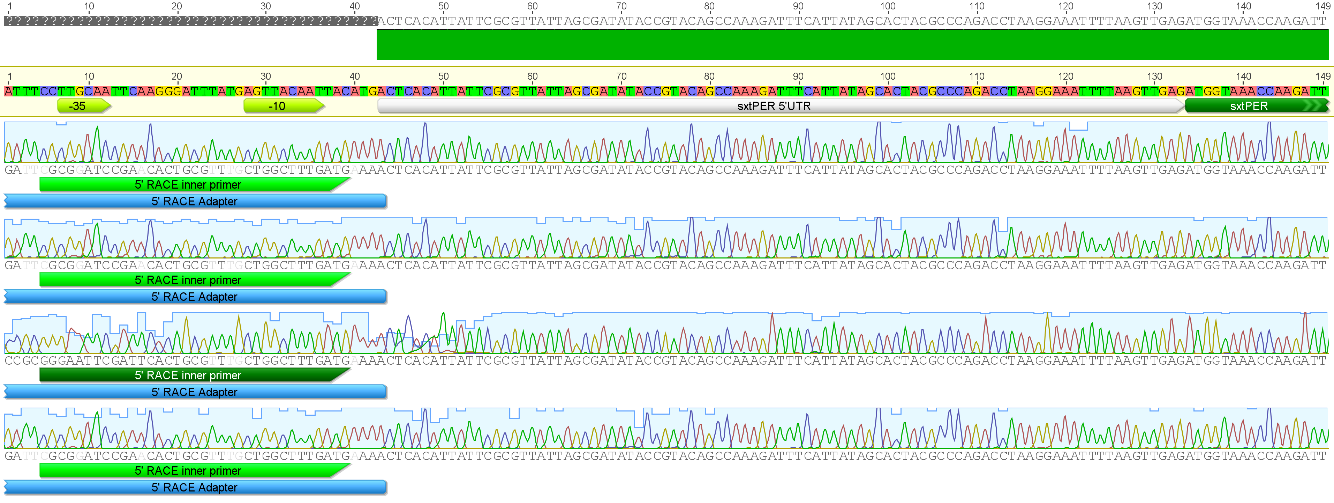


**Transcription start point of *orf24***


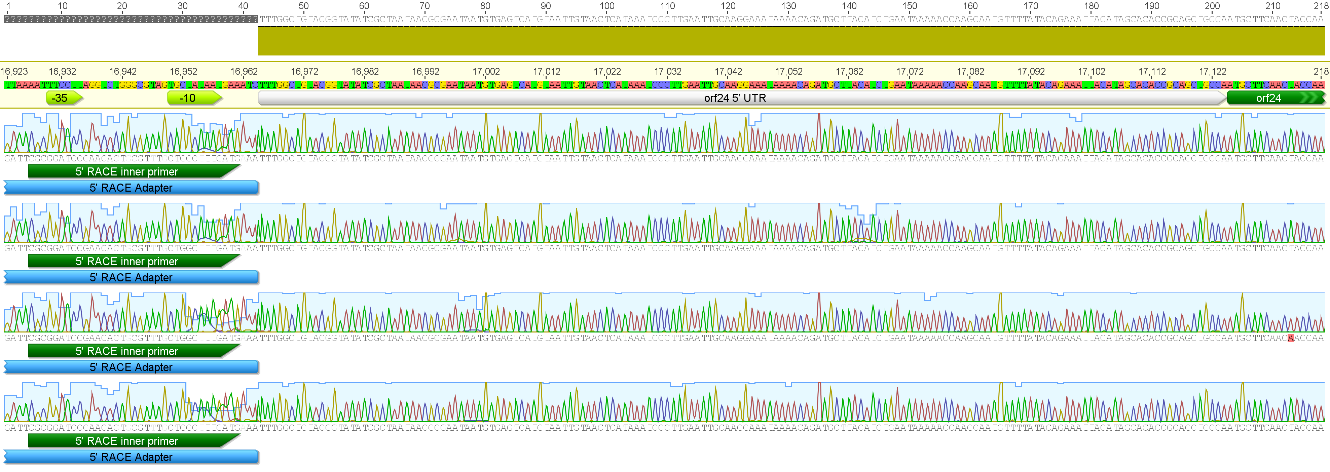


**Figure S2: Experimental determination of transcription start point *via* 5' RACE**

The TSP of each transcript was determined using 5′ RACE. The first nucleotide located downstream of the 5′ RACE adapter (blue) is the 5′ terminal bp for each transcript and thus, the first bp of the 5′ UTR (grey) followed by the encoding gene (green). The 5′ RACE inner primer is show and was used for amplification in conjunction with four reverse facing GSPs. Dark green indicates sequencing in the forward direction whilst light green indicates sequencing in the reverse direction. The nucleotide sequencing within the yellow highlighted region is the reference sequence of the 131C *sxt* cluster.


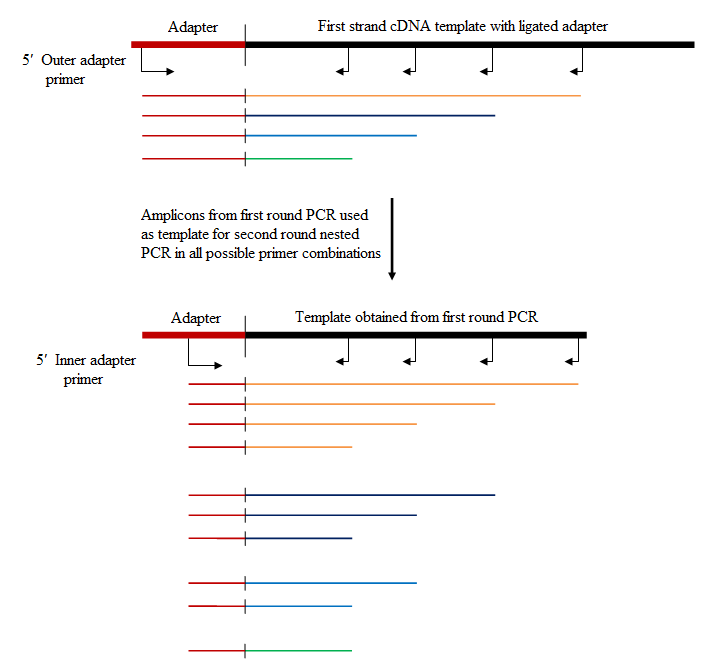


**Figure S3: Nested PCR for the determination of TSPs**

As a template for 5′ RACE, cDNA was synthesised from RNA containing a small adapter ligated to the 5′ end of mRNA transcripts. First round PCR to determine TSSs of the *sxt* gene cluster was achieved with a 5′ outer adapter primer in conjunction with 4 reverse facing GSPs Amplicons generated from first round PCR was used as a template for second round PCR. Amplification was performed in combination with all possible reverse facing primers in conjunction with a 5′ inner adapter primer. Knowledge of the distance between primers allowed for distinction between correct bands and background amplification.


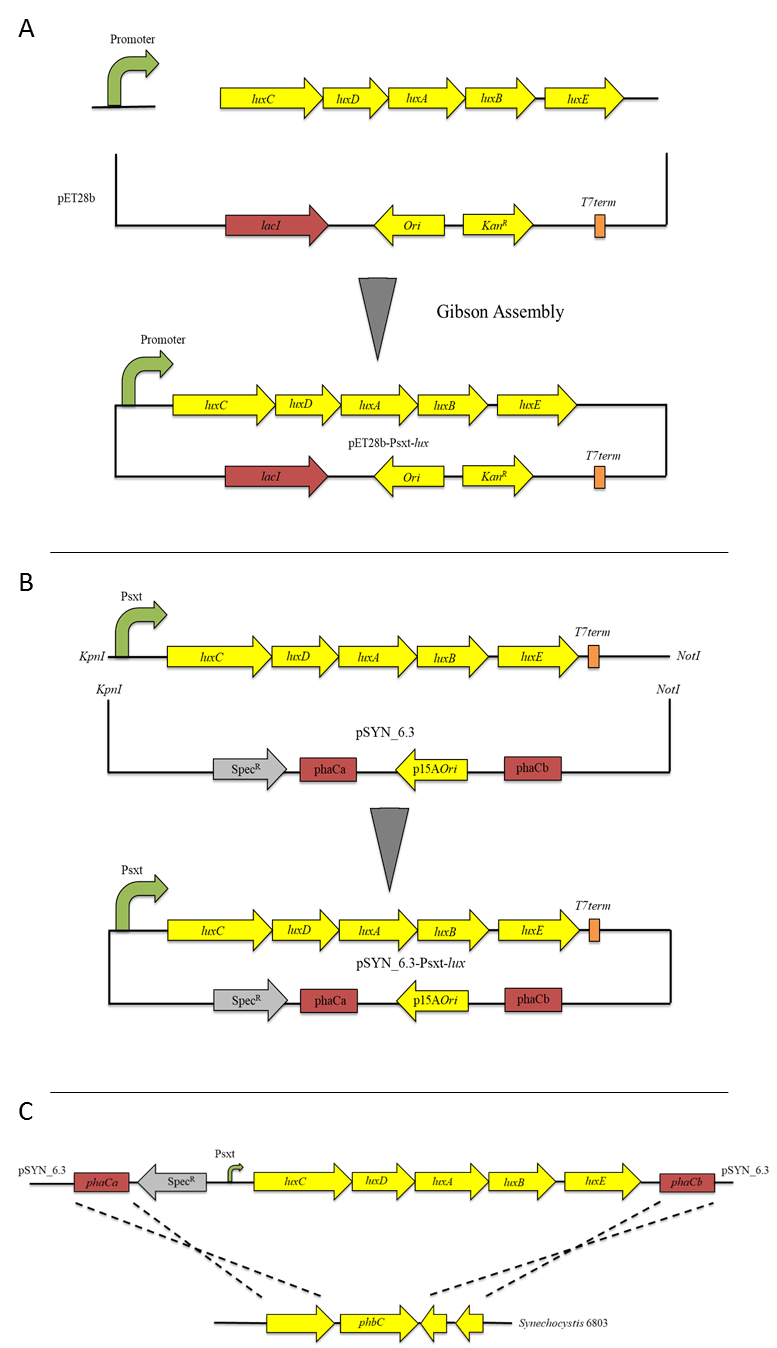


**Figure S4. Cloning of P*sxt*-*lux* *E. coli* expression vectors and *Synechocystis* integration vectors:** (A) Linear *luxCDABE* and promoter fragments were cloned into the pET28b vector through Gibson assembly. (B) P-*lux* fragments were digested with *Not*I/*Kpn*I and ligated into the pSYN_6.3 vector. (C) Transformation of P*sxt*-*lux* into the *Synechocystis* PCC6803 genome: The pSYN_6.3 vector was mobilised into *Synechocystis* PCC6803 through natural transformation. The P*sxt*-*lux* fragment on the pSYN_6.3 vector was integrated into the *Synechocystis* PCC6803 genome by replacing the *phbC* gene through homologous recombination.

| **Table S1: Transcript termination sites determined by TranstermHP** | | | | | |
| --- | --- | --- | --- | --- | --- |
| **Operon** | **dG** | **Score** | **5′ Tail** | **Stem-loop** | **3′ tail** |
| **1** | - | - | - | - | - |
| **2** | -7.7 | -3.95 | TCCTTTTTACAAAAA | TGGGA-TGC-TCCCA | TTTAATTGCGCTTTT |
| **3** | -2.5 | -2.95 | TAAACTACGGCTATT | TGAATAATG-GGAGAG-CATTATCCA | TAATTATTACCTCGT |
| **4** | -4 | -4.58 | CTTA-ACA-TAAG | CTTA-ACA-TAAG | TTTTTCCCACAATTC |

| **Table S2: unpaired t-tests *E. coli*** | | | | | | |
| --- | --- | --- | --- | --- | --- | --- |
| Table Analyzed | RLU/OD | RLU/OD | RLU/OD | RLU/OD | RLU/OD | RLU/OD |
| Column F | **P*sxtP*** | **Control** | **Control** | **Control** | **P*orf24*** | **Control** |
| vs. | vs. | vs. | vs. | vs. | vs. | vs. |
| Column E | **Control** | **P*sxtPER*** | **P*sxtD*** | **P*sxtPER2*** | **Control** | **P*sxtO*** |
| Unpaired t test |  |  |  |  |  |  |
| **P value** | **<0.0001** | **<0.0001** | **<0.0001** | **<0.0001** | **<0.0001** | **<0.0001** |
| P value summary | **** | **** | **** | **** | **** | **** |
| Significantly different (P < 0.05)? | Yes | Yes | Yes | Yes | Yes | Yes |
| One- or two-tailed P value? | Two-tailed | Two-tailed | Two-tailed | Two-tailed | Two-tailed | Two-tailed |
| t, df | t=9.711 df=13 | t=13.52 df=13 | t=7.296 df=13 | t=10.77 df=13 | t=11.42 df=10 | t=9.649 df=13 |
| How big is the difference? |  |  |  |  |  |  |
| Mean ± SEM of column E | 1467 ± 136.5, n=9 | 157740426 ± 14574405, n=6 | 926543945 ± 158604243, n=6 | 2600089 ± 301443, n=6 | 1467 ± 136.5, n=9 | 97263 ± 12398, n=6 |
| Mean ± SEM of column F | 111526337 ± 14343689, n=6 | 1467 ± 136.5, n=9 | 1467 ± 136.5, n=9 | 1467 ± 136.5, n=9 | 1188002 ± 201199, n=3 | 1467 ± 136.5, n=9 |
| Difference between means | 111524871 ± 11484142 | -157738959 ± 11668863 | -926542478 ± 126985023 | -2598622 ± 241348 | 1186535 ± 103899 | -95796 ± 9928 |
| 95% confidence interval | 86714890 to 136334851 | -182948005 to -132529913 | -1200876941 to -652208015 | -3120022 to -2077222 | 955034 to 1418037 | -117244 to -74348 |
| R squared (eta squared) | 0.8789 | 0.9336 | 0.8037 | 0.8992 | 0.9288 | 0.8775 |
| F test to compare variances |  |  |  |  |  |  |
| F, DFn, Dfd | 7366117336, 5, 8 | 7604988994, 5, 8 | 900630216132, 5, 8 | 3253323, 5, 8 | 724666, 2, 8 | 5503, 5, 8 |
| P value | <0.0001 | <0.0001 | <0.0001 | <0.0001 | <0.0001 | <0.0001 |
| P value summary | **** | **** | **** | **** | **** | **** |
| Significantly different (P < 0.05)? | Yes | Yes | Yes | Yes | Yes | Yes |

| **Table S3: Relative expression of promoters vs control in *E. coli*** | | | | | |
| --- | --- | --- | --- | --- | --- |
| **pSXL1** | **pSXL2** | **pSXL3** | **pSXL4** | **pSXL5** | **pSXL6** |
| 631591 | 76023 | 107526 | 810 | 1772 | 66 |

| **Table S4: *P* values from one-way ANOVA in *E. coli*** | | | | | | |
| --- | --- | --- | --- | --- | --- | --- |
|  | PsxtD | PsxtP | PsxtPER | Porf24 | PsxtPER2 | PsxtO |
| PsxtD |  | <0.0001 | <0.0001 | <0.0001 | <0.0001 | <0.0001 |
| PsxtP | <0.0001 |  | >0.9999 | >0.9999 | >0.9999 | >0.9999 |
| PsxtPER | <0.0001 | >0.9999 |  | >0.9999 | >0.9999 | >0.9999 |
| Porf24 | <0.0001 | >0.9999 | >0.9999 |  | >0.9999 | >0.9999 |
| PsxtPER2 | <0.0001 | >0.9999 | >0.9999 | >0.9999 |  | >0.9999 |
| PsxtO | <0.0001 | >0.9999 | >0.9999 | >0.9999 | >0.9999 |  |

| **Table S5: Unpaired t-tests *Synechocystis*** | | | | |
| --- | --- | --- | --- | --- |
| Table Analyzed | RLU/OD | RLU/OD | RLU/OD | RLU/OD |
| Column E | **Control** | **Control** | **Control** | **Control** |
| vs. | vs. | vs. | vs. | vs. |
| Column B | **PsxtP** | **PsxtPER** | **Porf24** | **PsxtD** |
| Unpaired t test |  |  |  |  |
| P value | 0.002 | <0.0001 | 0.0105 | 0.1347 |
| P value summary | ** | **** | * | ns |
| Significantly different (P < 0.05)? | Yes | Yes | Yes | No |
| One- or two-tailed P value? | Two-tailed | Two-tailed | Two-tailed | Two-tailed |
| t, df | t=7.19 df=4 | t=34.09 df=4 | t=4.536 df=4 | t=1.871 df=4 |
| How big is the difference? |  |  |  |  |
| Mean ± SEM of column B | 243162 ± 29557, n=3 | 428831 ± 11066, n=3 | 126747 ± 21240, n=3 | 38636 ± 3532, n=3 |
| Mean ± SEM of column E | 28791 ± 3902, n=3 | 28791 ± 3902, n=3 | 28791 ± 3902, n=3 | 28791 ± 3902, n=3 |
| Difference between means | -214372 ± 29814 | -400040 ± 11734 | -97957 ± 21595 | -9846 ± 5263 |
| 95% confidence interval | -297148 to -131595 | -432618 to -367462 | -157915 to -37998 | -24458 to 4767 |
| R squared (eta squared) | 0.9282 | 0.9966 | 0.8372 | 0.4666 |
| F test to compare variances |  |  |  |  |
| F, DFn, Dfd | 57.39, 2, 2 | 8.044, 2, 2 | 29.64, 2, 2 | 1.22, 2, 2 |
| P value | 0.0343 | 0.2211 | 0.0653 | 0.9008 |
| P value summary | * | ns | ns | ns |
| Significantly different (P < 0.05)? | Yes | No | No | No |

| **Table S6: Relative expression of promoters vs control in *Synechocystis*** | | | |
| --- | --- | --- | --- |
| **PsxtD** | **PsxtP** | **PsxtPER** | **Porf24** |
| 1.34 | 8.45 | 14.89 | 4.40 |

| **Table S7: *P* values from one-way ANOVA in *Synechocystis*** | | | | |
| --- | --- | --- | --- | --- |
|  | PsxtD | PsxtP | PsxtPER | Porf24 |
| PsxtD |  | 0.0004 | <0.0001 | 0.069 |
| PsxtP | 0.0004 |  | 0.0008 | 0.0155 |
| PsxtPER | <0.0001 | 0.0008 |  | <0.0001 |
| Porf24 | 0.069 | 0.0155 | <0.0001 |  |

| Table S8: Oligonucleotides used for transcriptional analysis | | | |
| --- | --- | --- | --- |
| **Gene** | **Primer** | **Sequence 5′ → 3′** | **Ref.** |
| *sxtC* | TAcF | AGAGGAGGAAAGTGTGCGAATC | This Study |
|  | TAcR | CAATTAATGCCCGTTGCTG | This Study |
| *sxtB* | TAbR | ACGCCCCTGCAACTCTCTCC | This Study |
|  | TAbF | AAAGTGGAACCTGAGCAATCG | This Study |
| *sxtA* | TAaR | ATTTTGATTACTCCGGCTATTTTC | This Study |
|  | TAaF3 | CAATCAAAATACCCATGCC | This Study |
|  | TAaR3 | GCTTCTAGATCCGATTGTGG | This Study |
|  | TAaF2 | AATCGCAACAAATCCGTGGG | This Study |
| *sxtE* | TAeR | AGCAATGGTGGCCAAGTAAC | This Study |
|  | TAeF | AAATGCGAAGACGAATGCTAGTG | This Study |
| *sxtV* | TAv1F | CTTTCTGAGCCGCCTGTGG | This Study |
|  | TAv1R | CACAGGCGGCTCAGAAAGGC | This Study |
|  | TAv2F | TCGCTGCGTGTTTCTGTGC | This Study |
|  | TAv2R | GCGGCTGCTCTTGTAGCTG | This Study |
| *sxtD* | TAdR | GCCCCGTGTTCATGCTTTGC | This Study |
|  | TAdF | ACGAGTGATATTCCTGCCC | This Study |
| *sxtP* | TApR | TGCTGCTAGAACTAACGAACTG | This Study |
|  | TApF | TCAACGGCGACAGCAAGCTC | This Study |
| *sxtQ* | TAqR | GCGTACCCAGGCAGGAAGACAC | This Study |
|  | TAqF | CCACCTCGGATCGGGAATG | This Study |
| *sxtR* | TArR | GCCAAAGCCCAGTCACAACC | This Study |
|  | TArF | CGAGCTAATGTCAAACAGGGGG | This Study |
|  | TAis41R | GAACTTGGCAAAACTGACAACGG | This Study |
|  | TAn2F | ATAGGGGAGAAATGTTTGGGACG | This Study |
|  | TAn2R | TGGGATAAAAGGATGCTGACATGG | This Study |
|  | TAorf25F | TCATCGCCTTAAAAGCCGC | This Study |
|  | TAorf26R | GCAAAGTAGGGCGGTCTG | This Study |
|  | TAn3F | GTTTGGGGTGCGGAATGTGG | This Study |
|  | TAn3R | AGCGGAGAAAGAGACAGGTATCACAG | This Study |
|  | TAorf10F | TCGGGGTTTATCTGGCTGGG | This Study |
|  | TAorf10R | TACGATGACGAGAATGAACAACTTGG | This Study |
|  | TAn4F | GAACGACTTCTAGGACAAGCCCAC | This Study |
|  | TAorf11R | GCTGGCATGGTGTTAGGGG | This Study |
|  | TAorf11F | ACAACAGTCCCATGATTGCCCC | This Study |
|  | TAorf12R | TCCTCCATCTCTTGGCGAAATTGTTC | This Study |
|  | TAorf12F | CTGGAATGAAGGCGCGGAG | This Study |
| *sxtS* | TAsR | ACCCGGCAATACTCCTCCAC | This Study |
|  | TAsF | GCTGTTGGCGGATGCTTTG | This Study |
| *sxtT* | TAtR | CCGTAGCGTTCCTGGCAATG | This Study |
|  | TAtF | ACAGCCAGCGCGTTTACCAC | This Study |
| *sxtU* | TAuR | ATTGCGACTTTTGCCCCCTC | This Study |
|  | TAuF | TGGGGCTGGAGTTTATAGTGC | This Study |
| *sxtN* | TAnR | CGAACGACTTCTAGGACAAGCCC | This Study |
|  | TAnF | GATCGTCCCAAACATTTCTCCC | This Study |
| *sxtG* | TAgR | GGCTGCTTGGCGATTGGTG | This Study |
|  | TAgF | CGACGCGGAACTCTTCAATC | This Study |
| *sxtH* | TAhR | GCACTTGCTGGGGGTATCATGTC | This Study |
|  | TAhF | CAACAACCAGCGCGTTTACC | This Study |
| *sxtM* | TAmR | CCAATTGTAGGCTGACTCGTGC | This Study |
|  | TAmF | AGTAGGCTGGGGTCTTGGGTTG | This Study |
| *sxtI* | TAiR | AACGTTCTTCCTGGGCTGCG | This Study |
|  | TAiF | TCCTCGTTCCCAAATTCCTGC | This Study |
| *sxtJ* | TAjR | CCGATCAGTCCAAATTCACGC | This Study |
|  | TAjF | TCCAGATTTGCCCACTTATCGC | This Study |
| *sxtK* | TAkR | CGAAGACAATTAGCGCCCCC | This Study |
|  | TAkF | GGGGGCGCTAATTGTCTTCG | This Study |
| *sxtL* | TAlR | AACCGGAAACATGAGGTCGCC | This Study |
|  | TAlF | AAGAAGGGCAGAAAGCAGC | This Study |
| *sxtO* | TAoR | ACGGGATTCGCAAACTTCTAAGGG | This Study |
| 16S | 27F | AGAGTTTGATCCTGGCTCAG | (1) |
|  | 809R | AGAGTTTGATCCTGGCTCAG | (1) |

| **Table S9: Oligonucleotide list for the identification of TSS** | | |
| --- | --- | --- |
| **Primer** | **Gene** | **Sequence 5′ → 3′** |
| TAdF | *sxtD* | ACGAGTGATATTCCTGCCC |
| TSPdF2 |  | CAGAGAGGCACATACATTAGTCC |
| TSPdF3 |  | ATTGCTTTGTTGAATGATGC |
| TSPdF4 |  | TATCCAATTATTATGAATCACTTGATG |
| TApR | *sxtP* | TGCTGCTAGAACTAACGAACTG |
| TSPpR2 |  | CACTAAATTGCCCAGAAAGC |
| TSPpR3 |  | GAAAAGTAATTGTGTTTGTCTCTGTAG |
| TSPpR4 |  | AGTTAGTAGCAGTGGCATAAGACC |
| TAorf11F | *sxtPER* | ACAACAGTCCCATGATTGCCCC |
| TSPperF2 |  | ACAGCATCACAAAGATCACG |
| TSPperF3 |  | TGTACGAACTGCTGAATTGC |
| TSPperF4 |  | TACCAAGATTGACATTGAGACTG |
| TAorf12R | *orf24* | TCCTCCATCTCTTGGCGAAATTGTTC |
| TSPorf24R2 |  | ATGAAACCAACGAAGACTAACAC |
| TSPorf24R3 |  | TCCAACGTCTGTGCATCTAG |
| TSPorf24R4 |  | TTAATGACTGTACTGATGCTAAGACG |
| 5′ RACE Adapter | GCUGAUGGCGAUGAAUGAACACUGCGUUUGCUGGCUUUGAUGAAA | |
| 5′ Inner Adapter | CGCGGATCCGAACACTGCGTTTGCTGGCTTTGATG | |
| 5′ Outer Adapter | GCTGATGGCGATGAATGAACACTG | |
| T7 | TAATACGACTCACTATAGGG | |
| M13R | CAGGAAACAGCTATGAC | |

| Table S10: Oligos used for Gibson assembly of the *E. coli* based pET28b::P*sxt* and *Synechocystis* based pSYN_6.3::P*sxt* *lux* reporter vectors | | |
| --- | --- | --- |
| Primer | **Sequence 5′ → 3′** | **Ref**. |
| lux-PsxtP_R | CACAAGACAACTCATGACTAAAAAAATTTCATTCATTATTAAC | This Study |
| lux-Porf24_R | ACCGCAGCTGCCAATGACTAAAAAAATTTCATTCATTATTAAC | This Study |
| lux-PsxtPER_R | ATTTTAAGTTGAGATGACTAAAAAAATTTCATTCATTATTAAC | This Study |
| Porf24-lux_F | TTTTTTTAGTCATTGGCAGCTGCGGTGTGCT | This Study |
| PsxtP-lux_F | TTTTTTTAGTCATGAGTTGTCTTGTGGAAATTTG | This Study |
| PsxtPER-lux_F | TTTTTTTAGTCATCTCAACTTAAAATTTCCTTAGGTC | This Study |
| Porf24-pET28_R | GATCGAGATCTCGATCCCGCGAAATGTTAGTGATTAAACCTTTAGTAATTGGCAAGATTGAGC | This Study |
| PsxtP-pET28_R | GATCGAGATCTCGATCCCGCGAAATCATAGATAATTCTAAAGACGAGTG | This Study |
| PsxtPER-pET28_R | GATCGAGATCTCGATCCCGCGAAATGCTGACAACATTGATAATCC | This Study |
| pET28_R | CTCGAGCACCACCACCAC | This Study |
| lux-pET28_F | agtggtggtggtggtggtgctcgagTCAACTATCAAACGCTTCG | This Study |
| lux-PsxtD_R | tgatagatacaaaATGACTAAAAAAATTTCATTCATTATTAAC | This Study |
| PsxtD-lux_F | tttttttagtcatTTTGTATCTATCATTGGTAACCTTAG | This Study |
| PsxtD-pET28_R | gatcgagatctcgatcccgcgaaatCAGAAAGCTTTTCATTCTTCAG | This Study |
| pET28_F | ATTTCGCGGGATCGAGATC | This Study |
| Screening primer_Fwd | TGCGTCAGCAACCAGTTATC | This Study |
| Screening primer_Rev | CGATCGCGTATTTCGTCTCGC | This Study |
| lacI-P-lux_NotI_F | *GCGCGCGGCCGC*CCAATCCGGATATAGTTC | This Study |
| LacI-P-lux-KpnI_R | *GCGCGGTACC*CGGTGCCTAATGAGTGAG | This Study |
| pSyn_6.3_NotI-R | *GCGCGCGGCCGC*GGAGATTAACTGTCAAAAAGGTG | This Study |
| pSyn_6.3-KpnI_F | *GCGCGGTACC*ATGCATCGAGTGCCTGGC | This Study |
| PhaCa_F | ATACACTCCGCTAAGAGTTACTTAAGCTTTCTTTTG | This Study |
| PacCb_R | AGCGTCAGACCCCTACCATTACCACCCTGGTAC | This Study |


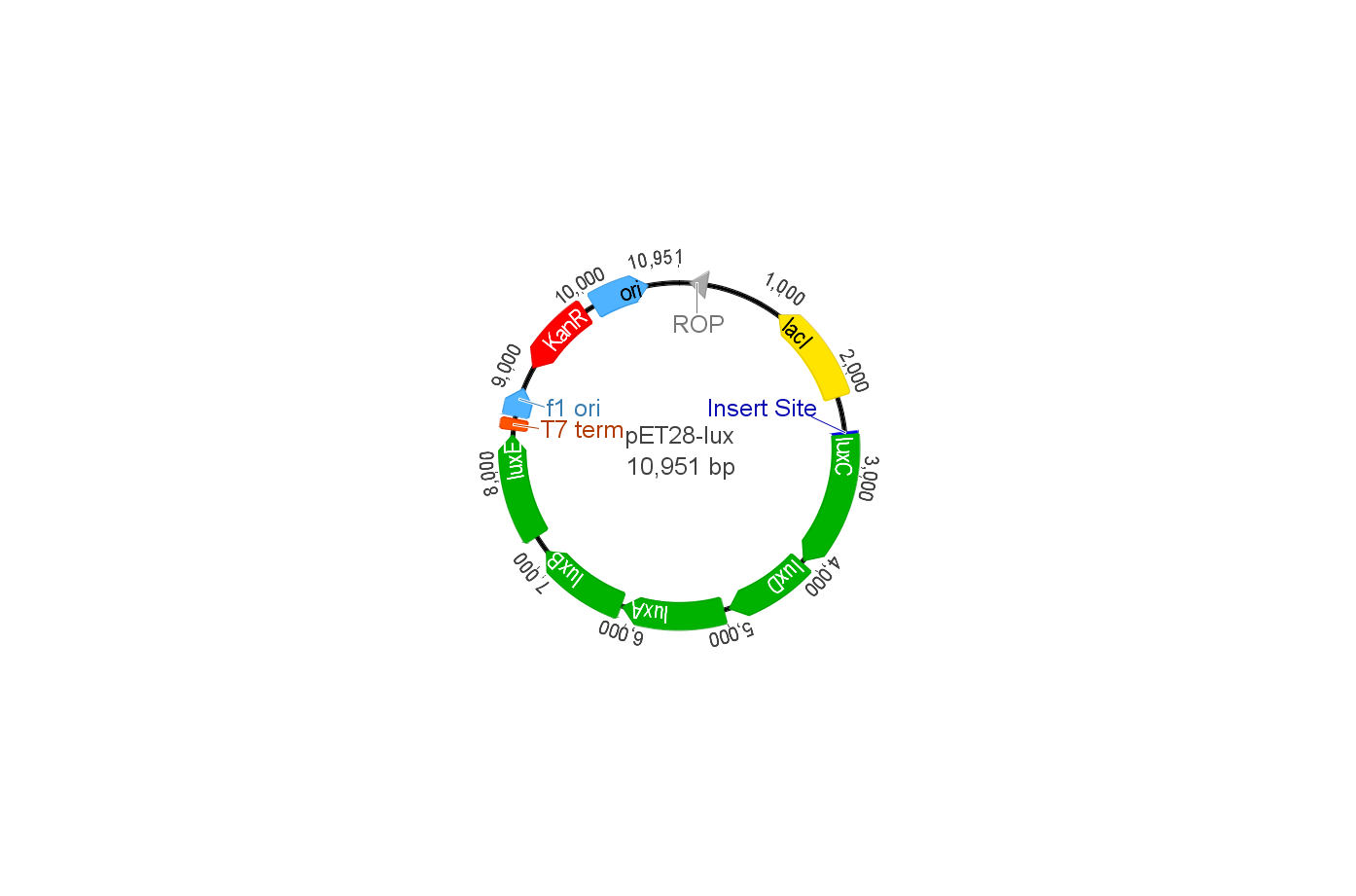


>pET28-lux_nucl

GCTGCGCCCCGACACCCGCCAACACCCGCTGACGCGCCCTGACGGGCTTGTCTGCTCCCGGCATCCGCTTACAGACAAGCTGTGACCGTCTCCGGGAGCTGCATGTGTCAGAGGTTTTCACCGTCATCACCGAAACGCGCGAGGCAGCTGCGGTAAAGCTCATCAGCGTGGTCGTGAAGCGATTCACAGATGTCTGCCTGTTCATCCGCGTCCAGCTCGTTGAGTTTCTCCAGAAGCGTTAATGTCTGGCTTCTGATAAAGCGGGCCATGTTAAGGGCGGTTTTTTCCTGTTTGGTCACTGATGCCTCCGTGTAAGGGGGATTTCTGTTCATGGGGGTAATGATACCGATGAAACGAGAGAGGATGCTCACGATACGGGTTACTGATGATGAACATGCCCGGTTACTGGAACGTTGTGAGGGTAAACAACTGGCGGTATGGATGCGGCGGGACCAGAGAAAAATCACTCAGGGTCAATGCCAGCGCTTCGTTAATACAGATGTAGGTGTTCCACAGGGTAGCCAGCAGCATCCTGCGATGCAGATCCGGAACATAATGGTGCAGGGCGCTGACTTCCGCGTTTCCAGACTTTACGAAACACGGAAACCGAAGACCATTCATGTTGTTGCTCAGGTCGCAGACGTTTTGCAGCAGCAGTCGCTTCACGTTCGCTCGCGTATCGGTGATTCATTCTGCTAACCAGTAAGGCAACCCCGCCAGCCTAGCCGGGTCCTCAACGACAGGAGCACGATCATGCGCACCCGTGGGGCCGCCATGCCGGCGATAATGGCCTGCTTCTCGCCGAAACGTTTGGTGGCGGGACCAGTGACGAAGGCTTGAGCGAGGGCGTGCAAGATTCCGAATACCGCAAGCGACAGGCCGATCATCGTCGCGCTCCAGCGAAAGCGGTCCTCGCCGAAAATGACCCAGAGCGCTGCCGGCACCTGTCCTACGAGTTGCATGATAAAGAAGACAGTCATAAGTGCGGCGACGATAGTCATGCCCCGCGCCCACCGGAAGGAGCTGACTGGGTTGAAGGCTCTCAAGGGCATCGGTCGAGATCCCGGTGCCTAATGAGTGAGCTAACTTACATTAATTGCGTTGCGCTCACTGCCCGCTTTCCAGTCGGGAAACCTGTCGTGCCAGCTGCATTAATGAATCGGCCAACGCGCGGGGAGAGGCGGTTTGCGTATTGGGCGCCAGGGTGGTTTTTCTTTTCACCAGTGAGACGGGCAACAGCTGATTGCCCTTCACCGCCTGGCCCTGAGAGAGTTGCAGCAAGCGGTCCACGCTGGTTTGCCCCAGCAGGCGAAAATCCTGTTTGATGGTGGTTAACGGCGGGATATAACATGAGCTGTCTTCGGTATCGTCGTATCCCACTACCGAGATATCCGCACCAACGCGCAGCCCGGACTCGGTAATGGCGCGCATTGCGCCCAGCGCCATCTGATCGTTGGCAACCAGCATCGCAGTGGGAACGATGCCCTCATTCAGCATTTGCATGGTTTGTTGAAAACCGGACATGGCACTCCAGTCGCCTTCCCGTTCCGCTATCGGCTGAATTTGATTGCGAGTGAGATATTTATGCCAGCCAGCCAGACGCAGACGCGCCGAGACAGAACTTAATGGGCCCGCTAACAGCGCGATTTGCTGGTGACCCAATGCGACCAGATGCTCCACGCCCAGTCGCGTACCGTCTTCATGGGAGAAAATAATACTGTTGATGGGTGTCTGGTCAGAGACATCAAGAAATAACGCCGGAACATTAGTGCAGGCAGCTTCCACAGCAATGGCATCCTGGTCATCCAGCGGATAGTTAATGATCAGCCCACTGACGCGTTGCGCGAGAAGATTGTGCACCGCCGCTTTACAGGCTTCGACGCCGCTTCGTTCTACCATCGACACCACCACGCTGGCACCCAGTTGATCGGCGCGAGATTTAATCGCCGCGACAATTTGCGACGGCGCGTGCAGGGCCAGACTGGAGGTGGCAACGCCAATCAGCAACGACTGTTTGCCCGCCAGTTGTTGTGCCACGCGGTTGGGAATGTAATTCAGCTCCGCCATCGCCGCTTCCACTTTTTCCCGCGTTTTCGCAGAAACGTGGCTGGCCTGGTTCACCACGCGGGAAACGGTCTGATAAGAGACACCGGCATACTCTGCGACATCGTATAACGTTACTGGTTTCACATTCACCACCCTGAATTGACTCTCTTCCGGGCGCTATCATGCCATACCGCGAAAGGTTTTGCGCCATTCGATGGTGTCCGGGATCTCGACGCTCTCCCTTATGCGACTCCTGCATTAGGAAGCAGCCCAGTAGTAGGTTGAGGCCGTTGAGCACCGCCGCCGCAAGGAATGGTGCATGCAAGGAGATGGCGCCCAACAGTCCCCCGGCCACGGGGCCTGCCACCATACCCACGCCGAAACAAGCGCTCATGAGCCCGAAGTGGCGAGCCCGATCTTCCCCATCGGTGATGTCGGCGATATAGGCGCCAGCAACCGCACCTGTGGCGCCGGTGATGCCGGCCACGATGCGTCCGGCGTAGAGGATCGAGATCTCGATCCCGCGAAATATGACTAAAAAAATTTCATTCATTATTAACGGCCAGGTTGAAATCTTTCCCGAAAGTGATGATTTAGTGCAATCCATTAATTTTGGTGATAATAGTGTTTACCTGCCAATATTGAATGACTCTCATGTAAAAAACATTATTGATTGTAATGGAAATAACGAATTACGGTTGCATAACATTGTCAATTTTCTCTATACGGTAGGGCAAAGATGGAAAAATGAAGAATACTCAAGACGCAGGACATACATTCGTGACTTAAAAAAATATATGGGATATTCAGAAGAAATGGCTAAGCTAGAGGCCAATTGGATATCTATGATTTTATGTTCTAAAGGCGGCCTTTATGATGTTGTAGAAAATGAACTTGGTTCTCGCCATATCATGGATGAATGGCTACCTCAGGATGAAAGTTATGTTCGGGCTTTTCCGAAAGGTAAATCTGTACATCTGTTGGCAGGTAATGTTCCATTATCTGGGATCATGTCTATATTACGCGCAATTTTAACTAAGAATCAGTGTATTATAAAAACATCGTCAACCGATCCTTTTACCGCTAATGCATTAGCGTTAAGTTTTATTGATGTAGACCCTAATCATCCGATAACGCGCTCTTTATCTGTTATATATTGGCCCCACCAAGGTGATACATCACTCGCAAAAGAAATTATGCGACATGCGGATGTTATTGTCGCTTGGGGAGGGCCAGATGCGATTAATTGGGCGGTAGAGCATGCGCCATCTTATGCTGATGTGATTAAATTTGGTTCTAAAAAGAGTCTTTGCATTATCGATAATCCTGTTGATTTGACGTCCGCAGCGACAGGTGCGGCTCATGATGTTTGTTTTTACGATCAGCGAGCTTGTTTTTCTGCCCAAAACATATATTACATGGGAAATCATTATGAGGAATTTAAGTTAGCGTTGATAGAAAAACTTAATCTATATGCGCATATATTACCGAATGCCAAAAAAGATTTTGATGAAAAGGCGGCCTATTCTTTAGTTCAAAAAGAAAGCTTGTTTGCTGGATTAAAAGTAGAGGTGGATATTCATCAACGTTGGATGATTATTGAGTCAAATGCAGGTGTGGAATTTAATCAACCACTTGGCAGATGTGTGTACCTTCATCACGTCGATAATATTGAGCAAATATTGCCTTATGTTCAAAAAAATAAGACGCAAACCATATCTATTTTTCCTTGGGAGTCATCATTTAAATATCGAGATGCGTTAGCATTAAAAGGTGCGGAAAGGATTGTAGAAGCAGGAATGAATAACATATTTCGAGTTGGTGGATCTCATGACGGAATGAGACCGTTGCAACGATTAGTGACATATATTTCTCATGAAAGGCCATCTAACTATACGGCTAAGGATGTTGCGGTTGAAATAGAACAGACTCGATTCCTGGAAGAAGATAAGTTCCTTGTATTTGTCCCATAATAGGTAAAAGTATGGAAAATGAATCAAAATATAAAACCATCGACCACGTTATTTGTGTTGAAGGAAATAAAAAAATTCATGTTTGGGAAACGCTGCCAGAAGAAAACAGCCCAAAGAGAAAGAATGCCATTATTATTGCGTCTGGTTTTGCCCGCAGGATGGATCATTTTGCTGGTCTGGCGGAATATTTATCGCGGAATGGATTTCATGTGATCCGCTATGATTCGCTTCACCACGTTGGATTGAGTTCAGGGACAATTGATGAATTTACAATGTCTATAGGAAAGCAGAGCTTGTTAGCAGTGGTTGATTGGTTAACTACACGAAAAATAAATAACTTCGGTATGTTGGCTTCAAGCTTATCTGCGCGGATAGCTTATGCAAGCCTATCTGAAATCAATGCTTCGTTTTTAATCACCGCAGTCGGTGTTGTTAACTTAAGATATTCTCTTGAAAGAGCTTTAGGGTTTGATTATCTCAGTCTACCCATTAATGAATTGCCGGATAATCTAGATTTTGAAGGCCATAAATTGGGTGCTGAAGTCTTTGCGAGAGATTGTCTTGATTTTGGTTGGGAAGATTTAGCTTCTACAATTAATAACATGATGTATCTTGATATACCGTTTATTGCTTTTACTGCAAATAACGATAATTGGGTCAAGCAAGATGAAGTTATCACATTGTTATCAAATATTCGTAGTAATCGATGCAAGATATATTCTTTGTTAGGAAGTTCGCATGACTTGAGTGAAAATTTAGTGGTCCTGCGCAATTTTTATCAATCGGTTACGAAAGCCGCTATCGCGATGGATAATGATCATCTGGATATTGATGTTGATATTACTGAACCGTCATTTGAACATTTAACTATTGCGACAGTCAATGAACGCCGAATGAGAATTGAGATTGAAAATCAAGCAATTTCTCTGTCTTAAAATCTATTGAGATATTCTATCACTCAAATAGCAATATAAGGACTCTCTATGAAATTTGGAAACTTTTTGCTTACATACCAACCTCCCCAATTTTCTCAAACAGAGGTAATGAAACGTTTGGTTAAATTAGGTCGCATCTCTGAGGAGTGTGGTTTTGATACCGTATGGTTACTGGAGCATCATTTCACGGAGTTTGGTTTGCTTGGTAACCCTTATGTCGCTGCTGCATATTTACTTGGCGCGACTAAAAAATTGAATGTAGGAACTGCCGCTATTGTTCTTCCCACAGCCCATCCAGTACGCCAACTTGAAGATGTGAATTTATTGGATCAAATGTCAAAAGGACGATTTCGGTTTGGTATTTGCCGAGGGCTTTACAACAAGGACTTTCGCGTATTCGGCACAGATATGAATAACAGTCGCGCCTTAGCGGAATGCTGGTACGGGCTGATAAAGAATGGCATGACAGAGGGATATATGGAAGCTGATAATGAACATATCAAGTTCCATAAGGTAAAAGTAAACCCCGCGGCGTATAGCAGAGGTGGCGCACCGGTTTATGTGGTGGCTGAATCAGCTTCGACGACTGAGTGGGCTGCTCAATTTGGCCTACCGATGATATTAAGTTGGATTATAAATACTAACGAAAAGAAAGCACAACTTGAGCTTTATAATGAAGTGGCTCAAGAATATGGGCACGATATTCATAATATCGACCATTGCTTATCATATATAACATCTGTAGATCATGACTCAATTAAAGCGAAAGAGATTTGCCGGAAATTTCTGGGGCATTGGTATGATTCTTATGTGAATGCTACGACTATTTTTGATGATTCAGACCAAACAAGAGGTTATGATTTCAATAAAGGGCAGTGGCGTGACTTTGTATTAAAAGGACATAAAGATACTAATCGCCGTATTGATTACAGTTACGAAATCAATCCCGTGGGAACGCCGCAGGAATGTATTGACATAATTCAAAAAGACATTGATGCTACAGGAATATCAAATATTTGTTGTGGATTTGAAGCTAATGGAACAGTAGACGAAATTATTGCTTCCATGAAGCTCTTCCAGTCTGATGTCATGCCATTTCTTAAAGAAAAACAACGTTCGCTATTATATTAGCTAAGGAGAAAGAAATGAAATTTGGATTGTTCTTCCTTAACTTCATCAATTCAACAACTGTTCAAGAACAAAGTATAGTTCGCATGCAGGAAATAACGGAGTATGTTGATAAGTTGAATTTTGAACAGATTTTAGTGTATGAAAATCATTTTTCAGATAATGGTGTTGTCGGCGCTCCTCTGACTGTTTCTGGTTTTCTGCTCGGTTTAACAGAGAAAATTAAAATTGGTTCATTAAATCACATCATTACAACTCATCATCCTGTCGCCATAGCGGAGGAAGCTTGCTTATTGGATCAGTTAAGTGAAGGGAGATTTATTTTAGGGTTTAGTGATTGCGAAAAAAAAGATGAAATGCATTTTTTTAATCGCCCGGTTGAATATCAACAGCAACTATTTGAAGAGTGTTATGAAATCATTAACGATGCTTTAACAACAGGCTATTGTAATCCAGATAACGATTTTTATAGCTTCCCTAAAATATCTGTAAATCCCCATGCTTATACGCCAGGCGGACCTCGGAAATATGTAACAGCAACCAGTCATCATATTGTTGAGTGGGCGGCCAAAAAAGGTATTCCTCTCATCTTTAAGTGGGATGATTCTAATGATGTTAGATATGAATATGCTGAAAGATATAAAGCCGTTGCGGATAAATATGACGTTGACCTATCAGAGATAGACCATCAGTTAATGATATTAGTTAACTATAACGAAGATAGTAATAAAGCTAAACAAGAGACGCGTGCATTTATTAGTGATTATGTTCTTGAAATGCACCCTAATGAAAATTTCGAAAATAAACTTGAAGAAATAATTGCAGAAAACGCTGTCGGAAATTATACGGAGTGTATAACTGCGGCTAAGTTGGCAATTGAAAAGTGTGGTGCGAAAAGTGTATTGCTGTCCTTTGAACCAATGAATGATTTGATGAGCCAAAAAAATGTAATCAATATTGTTGATGATAATATTAAGAAGTACCACATGGAATATACCTAATAGATTTCGAGTTGCAGCGAGGCGGCAAGTGAACGAATCCCCAGGAGCATAGATAACTATGTGACTGGGGTGAGTGAAAGCAGCCAACAAAGCAGCAGCTTGAAAGATGAAGGGTATAAAAGAGTATGACAGCAGTGCTGCCATACTTTCTAATATTATCTTGAGGAGTAAAACAGGTATGACTTCATATGTTGATAAACAAGAAATTACAGCAAGCTCAGAAATTGATGATTTGATTTTTTCGAGCGATCCATTAGTGTGGTCTTACGACGAGCAGGAAAAAATCAGAAAGAAACTTGTGCTTGATGCATTTCGTAATCATTATAAACATTGTCGAGAATATCGTCACTACTGTCAGGCACACAAAGTAGATGACAATATTACGGAAATTGATGACATACCTGTATTCCCAACATCGGTTTTTAAGTTTACTCGCTTATTAACTTCTCAGGAAAACGAGATTGAAAGTTGGTTTACCAGTAGCGGCACGAATGGTTTAAAAAGTCAGGTGGCGCGTGACAGATTAAGTATTGAGAGACTCTTAGGCTCTGTGAGTTATGGCATGAAATATGTTGGTAGTTGGTTTGATCATCAAATAGAATTAGTCAATTTGGGACCAGATAGATTTAATGCTCATAATATTTGGTTTAAATATGTTATGAGTTTGGTGGAATTGTTATATCCTACGACATTTACCGTAACAGAAGAACGAATAGATTTTGTTAAAACATTGAATAGTCTTGAACGAATAAAAAATCAAGGGAAAGATCTTTGTCTTATTGGTTCGCCATACTTTATTTATTTACTCTGCCATTATATGAAAGATAAAAAAATCTCATTTTCTGGAGATAAAAGCCTTTATATCATAACCGGAGGCGGCTGGAAAAGTTACGAAAAAGAATCTCTGAAACGTGATGATTTCAATCATCTTTTATTTGATACTTTCAATCTCAGTGATATTAGTCAGATCCGAGATATATTTAATCAAGTTGAACTCAACACTTGTTTCTTTGAGGATGAAATGCAGCGTAAACATGTTCCGCCGTGGGTATATGCGCGAGCGCTTGATCCTGAAACGTTGAAACCTGTACCTGATGGAACGCCGGGGTTGATGAGTTATATGGATGCGTCAGCAACCAGTTATCCAGCATTTATTGTTACCGATGATGTCGGGATAATTAGCAGAGAATATGGTAAGTATCCCGGCGTGCTCGTTGAAATTTTACGTCGCGTCAATACGAGGACGCAGAAAGGGTGTGCTTTAAGCTTAACCGAAGCGTTTGATAGTTGAGGCCGCACTCGAGCACCACCACCACCACCACTGAGATCCGGCTGCTAACAAAGCCCGAAAGGAAGCTGAGTTGGCTGCTGCCACCGCTGAGCAATAACTAGCATAACCCCTTGGGGCCTCTAAACGGGTCTTGAGGGGTTTTTTGCTGAAAGGAGGAACTATATCCGGATTGGCGAATGGGACGCGCCCTGTAGCGGCGCATTAAGCGCGGCGGGTGTGGTGGTTACGCGCAGCGTGACCGCTACACTTGCCAGCGCCCTAGCGCCCGCTCCTTTCGCTTTCTTCCCTTCCTTTCTCGCCACGTTCGCCGGCTTTCCCCGTCAAGCTCTAAATCGGGGGCTCCCTTTAGGGTTCCGATTTAGTGCTTTACGGCACCTCGACCCCAAAAAACTTGATTAGGGTGATGGTTCACGTAGTGGGCCATCGCCCTGATAGACGGTTTTTCGCCCTTTGACGTTGGAGTCCACGTTCTTTAATAGTGGACTCTTGTTCCAAACTGGAACAACACTCAACCCTATCTCGGTCTATTCTTTTGATTTATAAGGGATTTTGCCGATTTCGGCCTATTGGTTAAAAAATGAGCTGATTTAACAAAAATTTAACGCGAATTTTAACAAAATATTAACGTTTACAATTTCAGGTGGCACTTTTCGGGGAAATGTGCGCGGAACCCCTATTTGTTTATTTTTCTAAATACATTCAAATATGTATCCGCTCATGAATTAATTCTTAGAAAAACTCATCGAGCATCAAATGAAACTGCAATTTATTCATATCAGGATTATCAATACCATATTTTTGAAAAAGCCGTTTCTGTAATGAAGGAGAAAACTCACCGAGGCAGTTCCATAGGATGGCAAGATCCTGGTATCGGTCTGCGATTCCGACTCGTCCAACATCAATACAACCTATTAATTTCCCCTCGTCAAAAATAAGGTTATCAAGTGAGAAATCACCATGAGTGACGACTGAATCCGGTGAGAATGGCAAAAGTTTATGCATTTCTTTCCAGACTTGTTCAACAGGCCAGCCATTACGCTCGTCATCAAAATCACTCGCATCAACCAAACCGTTATTCATTCGTGATTGCGCCTGAGCGAGACGAAATACGCGATCGCTGTTAAAAGGACAATTACAAACAGGAATCGAATGCAACCGGCGCAGGAACACTGCCAGCGCATCAACAATATTTTCACCTGAATCAGGATATTCTTCTAATACCTGGAATGCTGTTTTCCCGGGGATCGCAGTGGTGAGTAACCATGCATCATCAGGAGTACGGATAAAATGCTTGATGGTCGGAAGAGGCATAAATTCCGTCAGCCAGTTTAGTCTGACCATCTCATCTGTAACATCATTGGCAACGCTACCTTTGCCATGTTTCAGAAACAACTCTGGCGCATCGGGCTTCCCATACAATCGATAGATTGTCGCACCTGATTGCCCGACATTATCGCGAGCCCATTTATACCCATATAAATCAGCATCCATGTTGGAATTTAATCGCGGCCTAGAGCAAGACGTTTCCCGTTGAATATGGCTCATAACACCCCTTGTATTACTGTTTATGTAAGCAGACAGTTTTATTGTTCATGACCAAAATCCCTTAACGTGAGTTTTCGTTCCACTGAGCGTCAGACCCCGTAGAAAAGATCAAAGGATCTTCTTGAGATCCTTTTTTTCTGCGCGTAATCTGCTGCTTGCAAACAAAAAAACCACCGCTACCAGCGGTGGTTTGTTTGCCGGATCAAGAGCTACCAACTCTTTTTCCGAAGGTAACTGGCTTCAGCAGAGCGCAGATACCAAATACTGTCCTTCTAGTGTAGCCGTAGTTAGGCCACCACTTCAAGAACTCTGTAGCACCGCCTACATACCTCGCTCTGCTAATCCTGTTACCAGTGGCTGCTGCCAGTGGCGATAAGTCGTGTCTTACCGGGTTGGACTCAAGACGATAGTTACCGGATAAGGCGCAGCGGTCGGGCTGAACGGGGGGTTCGTGCACACAGCCCAGCTTGGAGCGAACGACCTACACCGAACTGAGATACCTACAGCGTGAGCTATGAGAAAGCGCCACGCTTCCCGAAGGGAGAAAGGCGGACAGGTATCCGGTAAGCGGCAGGGTCGGAACAGGAGAGCGCACGAGGGAGCTTCCAGGGGGAAACGCCTGGTATCTTTATAGTCCTGTCGGGTTTCGCCACCTCTGACTTGAGCGTCGATTTTTGTGATGCTCGTCAGGGGGGCGGAGCCTATGGAAAAACGCCAGCAACGCGGCCTTTTTACGGTTCCTGGCCTTTTGCTGGCCTTTTGCTCACATGTTCTTTCCTGCGTTATCCCCTGATTCTGTGGATAACCGTATTACCGCCTTTGAGTGAGCTGATACCGCTCGCCGCAGCCGAACGACCGAGCGCAGCGAGTCAGTGAGCGAGGAAGCGGAAGAGCGCCTGATGCGGTATTTTCTCCTTACGCATCTGTGCGGTATTTCACACCGCATATATGGTGCACTCTCAGTACAATCTGCTCTGATGCCGCATAGTTAAGCCAGTATACACTCCGCTATCGCTACGTGACTGGGTCATG


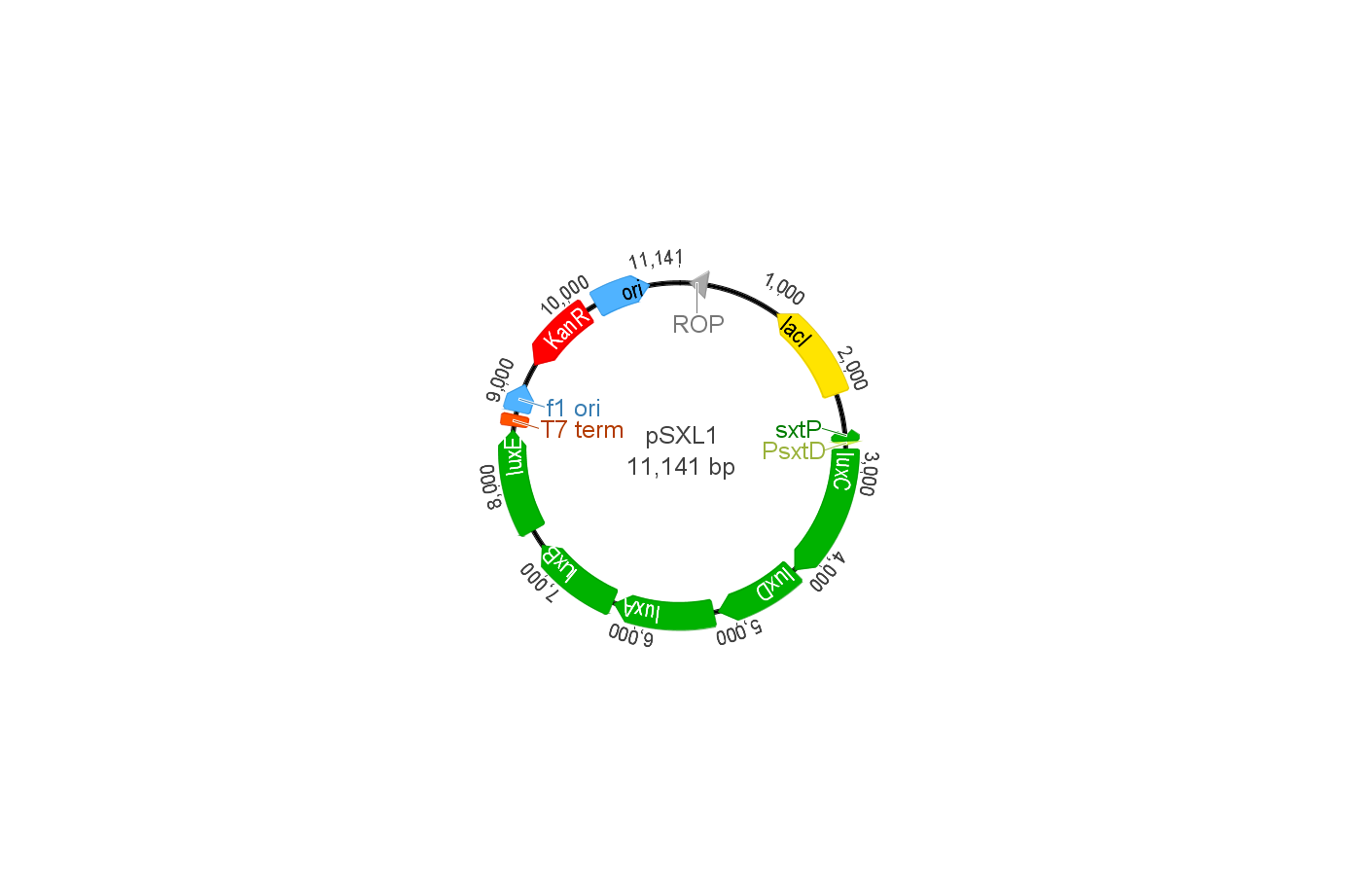


>pSXL1_nucl

CGCCCCGACACCCGCCAACACCCGCTGACGCGCCCTGACGGGCTTGTCTGCTCCCGGCATCCGCTTACAGACAAGCTGTGACCGTCTCCGGGAGCTGCATGTGTCAGAGGTTTTCACCGTCATCACCGAAACGCGCGAGGCAGCTGCGGTAAAGCTCATCAGCGTGGTCGTGAAGCGATTCACAGATGTCTGCCTGTTCATCCGCGTCCAGCTCGTTGAGTTTCTCCAGAAGCGTTAATGTCTGGCTTCTGATAAAGCGGGCCATGTTAAGGGCGGTTTTTTCCTGTTTGGTCACTGATGCCTCCGTGTAAGGGGGATTTCTGTTCATGGGGGTAATGATACCGATGAAACGAGAGAGGATGCTCACGATACGGGTTACTGATGATGAACATGCCCGGTTACTGGAACGTTGTGAGGGTAAACAACTGGCGGTATGGATGCGGCGGGACCAGAGAAAAATCACTCAGGGTCAATGCCAGCGCTTCGTTAATACAGATGTAGGTGTTCCACAGGGTAGCCAGCAGCATCCTGCGATGCAGATCCGGAACATAATGGTGCAGGGCGCTGACTTCCGCGTTTCCAGACTTTACGAAACACGGAAACCGAAGACCATTCATGTTGTTGCTCAGGTCGCAGACGTTTTGCAGCAGCAGTCGCTTCACGTTCGCTCGCGTATCGGTGATTCATTCTGCTAACCAGTAAGGCAACCCCGCCAGCCTAGCCGGGTCCTCAACGACAGGAGCACGATCATGCGCACCCGTGGGGCCGCCATGCCGGCGATAATGGCCTGCTTCTCGCCGAAACGTTTGGTGGCGGGACCAGTGACGAAGGCTTGAGCGAGGGCGTGCAAGATTCCGAATACCGCAAGCGACAGGCCGATCATCGTCGCGCTCCAGCGAAAGCGGTCCTCGCCGAAAATGACCCAGAGCGCTGCCGGCACCTGTCCTACGAGTTGCATGATAAAGAAGACAGTCATAAGTGCGGCGACGATAGTCATGCCCCGCGCCCACCGGAAGGAGCTGACTGGGTTGAAGGCTCTCAAGGGCATCGGTCGAGATCCCGGTGCCTAATGAGTGAGCTAACTTACATTAATTGCGTTGCGCTCACTGCCCGCTTTCCAGTCGGGAAACCTGTCGTGCCAGCTGCATTAATGAATCGGCCAACGCGCGGGGAGAGGCGGTTTGCGTATTGGGCGCCAGGGTGGTTTTTCTTTTCACCAGTGAGACGGGCAACAGCTGATTGCCCTTCACCGCCTGGCCCTGAGAGAGTTGCAGCAAGCGGTCCACGCTGGTTTGCCCCAGCAGGCGAAAATCCTGTTTGATGGTGGTTAACGGCGGGATATAACATGAGCTGTCTTCGGTATCGTCGTATCCCACTACCGAGATATCCGCACCAACGCGCAGCCCGGACTCGGTAATGGCGCGCATTGCGCCCAGCGCCATCTGATCGTTGGCAACCAGCATCGCAGTGGGAACGATGCCCTCATTCAGCATTTGCATGGTTTGTTGAAAACCGGACATGGCACTCCAGTCGCCTTCCCGTTCCGCTATCGGCTGAATTTGATTGCGAGTGAGATATTTATGCCAGCCAGCCAGACGCAGACGCGCCGAGACAGAACTTAATGGGCCCGCTAACAGCGCGATTTGCTGGTGACCCAATGCGACCAGATGCTCCACGCCCAGTCGCGTACCGTCTTCATGGGAGAAAATAATACTGTTGATGGGTGTCTGGTCAGAGACATCAAGAAATAACGCCGGAACATTAGTGCAGGCAGCTTCCACAGCAATGGCATCCTGGTCATCCAGCGGATAGTTAATGATCAGCCCACTGACGCGTTGCGCGAGAAGATTGTGCACCGCCGCTTTACAGGCTTCGACGCCGCTTCGTTCTACCATCGACACCACCACGCTGGCACCCAGTTGATCGGCGCGAGATTTAATCGCCGCGACAATTTGCGACGGCGCGTGCAGGGCCAGACTGGAGGTGGCAACGCCAATCAGCAACGACTGTTTGCCCGCCAGTTGTTGTGCCACGCGGTTGGGAATGTAATTCAGCTCCGCCATCGCCGCTTCCACTTTTTCCCGCGTTTTCGCAGAAACGTGGCTGGCCTGGTTCACCACGCGGGAAACGGTCTGATAAGAGACACCGGCATACTCTGCGACATCGTATAACGTTACTGGTTTCACATTCACCACCCTGAATTGACTCTCTTCCGGGCGCTATCATGCCATACCGCGAAAGGTTTTGCGCCATTCGATGGTGTCCGGGATCTCGACGCTCTCCCTTATGCGACTCCTGCATTAGGAAGCAGCCCAGTAGTAGGTTGAGGCCGTTGAGCACCGCCGCCGCAAGGAATGGTGCATGCAAGGAGATGGCGCCCAACAGTCCCCCGGCCACGGGGCCTGCCACCATACCCACGCCGAAACAAGCGCTCATGAGCCCGAAGTGGCGAGCCCGATCTTCCCCATCGGTGATGTCGGCGATATAGGCGCCAGCAACCGCACCTGTGGCGCCGGTGATGCCGGCCACGATGCGTCCGGCGTAGAGGATCGAGATCTCGATCCCGCGAAATCAGAAAGCTTTTCATTCTTCAGATAATCGTTAGTTGTATCTGCTAATGCAACAGTGGTTATACATAGGAATGTGCTGACAAACAAGAGTGCTGCTAGAACTAACGAACTGAGCCTTTTCATGAGTTGTCTTGTGGAAATTTGTTAATAAGAGTATACTTGACTAGTATACACTAAGGTTACCAATGATAGATACAAAATGACTAAAAAAATTTCATTCATTATTAACGGCCAGGTTGAAATCTTTCCCGAAAGTGATGATTTAGTGCAATCCATTAATTTTGGTGATAATAGTGTTTACCTGCCAATATTGAATGACTCTCATGTAAAAAACATTATTGATTGTAATGGAAATAACGAATTACGGTTGCATAACATTGTCAATTTTCTCTATACGGTAGGGCAAAGATGGAAAAATGAAGAATACTCAAGACGCAGGACATACATTCGTGACTTAAAAAAATATATGGGATATTCAGAAGAAATGGCTAAGCTAGAGGCCAATTGGATATCTATGATTTTATGTTCTAAAGGCGGCCTTTATGATGTTGTAGAAAATGAACTTGGTTCTCGCCATATCATGGATGAATGGCTACCTCAGGATGAAAGTTATGTTCGGGCTTTTCCGAAAGGTAAATCTGTACATCTGTTGGCAGGTAATGTTCCATTATCTGGGATCATGTCTATATTACGCGCAATTTTAACTAAGAATCAGTGTATTATAAAAACATCGTCAACCGATCCTTTTACCGCTAATGCATTAGCGTTAAGTTTTATTGATGTAGACCCTAATCATCCGATAACGCGCTCTTTATCTGTTATATATTGGCCCCACCAAGGTGATACATCACTCGCAAAAGAAATTATGCGACATGCGGATGTTATTGTCGCTTGGGGAGGGCCAGATGCGATTAATTGGGCGGTAGAGCATGCGCCATCTTATGCTGATGTGATTAAATTTGGTTCTAAAAAGAGTCTTTGCATTATCGATAATCCTGTTGATTTGACGTCCGCAGCGACAGGTGCGGCTCATGATGTTTGTTTTTACGATCAGCGAGCTTGTTTTTCTGCCCAAAACATATATTACATGGGAAATCATTATGAGGAATTTAAGTTAGCGTTGATAGAAAAACTTAATCTATATGCGCATATATTACCGAATGCCAAAAAAGATTTTGATGAAAAGGCGGCCTATTCTTTAGTTCAAAAAGAAAGCTTGTTTGCTGGATTAAAAGTAGAGGTGGATATTCATCAACGTTGGATGATTATTGAGTCAAATGCAGGTGTGGAATTTAATCAACCACTTGGCAGATGTGTGTACCTTCATCACGTCGATAATATTGAGCAAATATTGCCTTATGTTCAAAAAAATAAGACGCAAACCATATCTATTTTTCCTTGGGAGTCATCATTTAAATATCGAGATGCGTTAGCATTAAAAGGTGCGGAAAGGATTGTAGAAGCAGGAATGAATAACATATTTCGAGTTGGTGGATCTCATGACGGAATGAGACCGTTGCAACGATTAGTGACATATATTTCTCATGAAAGGCCATCTAACTATACGGCTAAGGATGTTGCGGTTGAAATAGAACAGACTCGATTCCTGGAAGAAGATAAGTTCCTTGTATTTGTCCCATAATAGGTAAAAGTATGGAAAATGAATCAAAATATAAAACCATCGACCACGTTATTTGTGTTGAAGGAAATAAAAAAATTCATGTTTGGGAAACGCTGCCAGAAGAAAACAGCCCAAAGAGAAAGAATGCCATTATTATTGCGTCTGGTTTTGCCCGCAGGATGGATCATTTTGCTGGTCTGGCGGAATATTTATCGCGGAATGGATTTCATGTGATCCGCTATGATTCGCTTCACCACGTTGGATTGAGTTCAGGGACAATTGATGAATTTACAATGTCTATAGGAAAGCAGAGCTTGTTAGCAGTGGTTGATTGGTTAACTACACGAAAAATAAATAACTTCGGTATGTTGGCTTCAAGCTTATCTGCGCGGATAGCTTATGCAAGCCTATCTGAAATCAATGCTTCGTTTTTAATCACCGCAGTCGGTGTTGTTAACTTAAGATATTCTCTTGAAAGAGCTTTAGGGTTTGATTATCTCAGTCTACCCATTAATGAATTGCCGGATAATCTAGATTTTGAAGGCCATAAATTGGGTGCTGAAGTCTTTGCGAGAGATTGTCTTGATTTTGGTTGGGAAGATTTAGCTTCTACAATTAATAACATGATGTATCTTGATATACCGTTTATTGCTTTTACTGCAAATAACGATAATTGGGTCAAGCAAGATGAAGTTATCACATTGTTATCAAATATTCGTAGTAATCGATGCAAGATATATTCTTTGTTAGGAAGTTCGCATGACTTGAGTGAAAATTTAGTGGTCCTGCGCAATTTTTATCAATCGGTTACGAAAGCCGCTATCGCGATGGATAATGATCATCTGGATATTGATGTTGATATTACTGAACCGTCATTTGAACATTTAACTATTGCGACAGTCAATGAACGCCGAATGAGAATTGAGATTGAAAATCAAGCAATTTCTCTGTCTTAAAATCTATTGAGATATTCTATCACTCAAATAGCAATATAAGGACTCTCTATGAAATTTGGAAACTTTTTGCTTACATACCAACCTCCCCAATTTTCTCAAACAGAGGTAATGAAACGTTTGGTTAAATTAGGTCGCATCTCTGAGGAGTGTGGTTTTGATACCGTATGGTTACTGGAGCATCATTTCACGGAGTTTGGTTTGCTTGGTAACCCTTATGTCGCTGCTGCATATTTACTTGGCGCGACTAAAAAATTGAATGTAGGAACTGCCGCTATTGTTCTTCCCACAGCCCATCCAGTACGCCAACTTGAAGATGTGAATTTATTGGATCAAATGTCAAAAGGACGATTTCGGTTTGGTATTTGCCGAGGGCTTTACAACAAGGACTTTCGCGTATTCGGCACAGATATGAATAACAGTCGCGCCTTAGCGGAATGCTGGTACGGGCTGATAAAGAATGGCATGACAGAGGGATATATGGAAGCTGATAATGAACATATCAAGTTCCATAAGGTAAAAGTAAACCCCGCGGCGTATAGCAGAGGTGGCGCACCGGTTTATGTGGTGGCTGAATCAGCTTCGACGACTGAGTGGGCTGCTCAATTTGGCCTACCGATGATATTAAGTTGGATTATAAATACTAACGAAAAGAAAGCACAACTTGAGCTTTATAATGAAGTGGCTCAAGAATATGGGCACGATATTCATAATATCGACCATTGCTTATCATATATAACATCTGTAGATCATGACTCAATTAAAGCGAAAGAGATTTGCCGGAAATTTCTGGGGCATTGGTATGATTCTTATGTGAATGCTACGACTATTTTTGATGATTCAGACCAAACAAGAGGTTATGATTTCAATAAAGGGCAGTGGCGTGACTTTGTATTAAAAGGACATAAAGATACTAATCGCCGTATTGATTACAGTTACGAAATCAATCCCGTGGGAACGCCGCAGGAATGTATTGACATAATTCAAAAAGACATTGATGCTACAGGAATATCAAATATTTGTTGTGGATTTGAAGCTAATGGAACAGTAGACGAAATTATTGCTTCCATGAAGCTCTTCCAGTCTGATGTCATGCCATTTCTTAAAGAAAAACAACGTTCGCTATTATATTAGCTAAGGAGAAAGAAATGAAATTTGGATTGTTCTTCCTTAACTTCATCAATTCAACAACTGTTCAAGAACAAAGTATAGTTCGCATGCAGGAAATAACGGAGTATGTTGATAAGTTGAATTTTGAACAGATTTTAGTGTATGAAAATCATTTTTCAGATAATGGTGTTGTCGGCGCTCCTCTGACTGTTTCTGGTTTTCTGCTCGGTTTAACAGAGAAAATTAAAATTGGTTCATTAAATCACATCATTACAACTCATCATCCTGTCGCCATAGCGGAGGAAGCTTGCTTATTGGATCAGTTAAGTGAAGGGAGATTTATTTTAGGGTTTAGTGATTGCGAAAAAAAAGATGAAATGCATTTTTTTAATCGCCCGGTTGAATATCAACAGCAACTATTTGAAGAGTGTTATGAAATCATTAACGATGCTTTAACAACAGGCTATTGTAATCCAGATAACGATTTTTATAGCTTCCCTAAAATATCTGTAAATCCCCATGCTTATACGCCAGGCGGACCTCGGAAATATGTAACAGCAACCAGTCATCATATTGTTGAGTGGGCGGCCAAAAAAGGTATTCCTCTCATCTTTAAGTGGGATGATTCTAATGATGTTAGATATGAATATGCTGAAAGATATAAAGCCGTTGCGGATAAATATGACGTTGACCTATCAGAGATAGACCATCAGTTAATGATATTAGTTAACTATAACGAAGATAGTAATAAAGCTAAACAAGAGACGCGTGCATTTATTAGTGATTATGTTCTTGAAATGCACCCTAATGAAAATTTCGAAAATAAACTTGAAGAAATAATTGCAGAAAACGCTGTCGGAAATTATACGGAGTGTATAACTGCGGCTAAGTTGGCAATTGAAAAGTGTGGTGCGAAAAGTGTATTGCTGTCCTTTGAACCAATGAATGATTTGATGAGCCAAAAAAATGTAATCAATATTGTTGATGATAATATTAAGAAGTACCACATGGAATATACCTAATAGATTTCGAGTTGCAGCGAGGCGGCAAGTGAACGAATCCCCAGGAGCATAGATAACTATGTGACTGGGGTGAGTGAAAGCAGCCAACAAAGCAGCAGCTTGAAAGATGAAGGGTATAAAAGAGTATGACAGCAGTGCTGCCATACTTTCTAATATTATCTTGAGGAGTAAAACAGGTATGACTTCATATGTTGATAAACAAGAAATTACAGCAAGCTCAGAAATTGATGATTTGATTTTTTCGAGCGATCCATTAGTGTGGTCTTACGACGAGCAGGAAAAAATCAGAAAGAAACTTGTGCTTGATGCATTTCGTAATCATTATAAACATTGTCGAGAATATCGTCACTACTGTCAGGCACACAAAGTAGATGACAATATTACGGAAATTGATGACATACCTGTATTCCCAACATCGGTTTTTAAGTTTACTCGCTTATTAACTTCTCAGGAAAACGAGATTGAAAGTTGGTTTACCAGTAGCGGCACGAATGGTTTAAAAAGTCAGGTGGCGCGTGACAGATTAAGTATTGAGAGACTCTTAGGCTCTGTGAGTTATGGCATGAAATATGTTGGTAGTTGGTTTGATCATCAAATAGAATTAGTCAATTTGGGACCAGATAGATTTAATGCTCATAATATTTGGTTTAAATATGTTATGAGTTTGGTGGAATTGTTATATCCTACGACATTTACCGTAACAGAAGAACGAATAGATTTTGTTAAAACATTGAATAGTCTTGAACGAATAAAAAATCAAGGGAAAGATCTTTGTCTTATTGGTTCGCCATACTTTATTTATTTACTCTGCCATTATATGAAAGATAAAAAAATCTCATTTTCTGGAGATAAAAGCCTTTATATCATAACCGGAGGCGGCTGGAAAAGTTACGAAAAAGAATCTCTGAAACGTGATGATTTCAATCATCTTTTATTTGATACTTTCAATCTCAGTGATATTAGTCAGATCCGAGATATATTTAATCAAGTTGAACTCAACACTTGTTTCTTTGAGGATGAAATGCAGCGTAAACATGTTCCGCCGTGGGTATATGCGCGAGCGCTTGATCCTGAAACGTTGAAACCTGTACCTGATGGAACGCCGGGGTTGATGAGTTATATGGATGCGTCAGCAACCAGTTATCCAGCATTTATTGTTACCGATGATGTCGGGATAATTAGCAGAGAATATGGTAAGTATCCCGGCGTGCTCGTTGAAATTTTACGTCGCGTCAATACGAGGACGCAGAAAGGGTGTGCTTTAAGCTTAACCGAAGCGTTTGATAGTTGACTCGAGCACCACCACCACCACCACTGAGATCCGGCTGCTAACAAAGCCCGAAAGGAAGCTGAGTTGGCTGCTGCCACCGCTGAGCAATAACTAGCATAACCCCTTGGGGCCTCTAAACGGGTCTTGAGGGGTTTTTTGCTGAAAGGAGGAACTATATCCGGATTGGCGAATGGGACGCGCCCTGTAGCGGCGCATTAAGCGCGGCGGGTGTGGTGGTTACGCGCAGCGTGACCGCTACACTTGCCAGCGCCCTAGCGCCCGCTCCTTTCGCTTTCTTCCCTTCCTTTCTCGCCACGTTCGCCGGCTTTCCCCGTCAAGCTCTAAATCGGGGGCTCCCTTTAGGGTTCCGATTTAGTGCTTTACGGCACCTCGACCCCAAAAAACTTGATTAGGGTGATGGTTCACGTAGTGGGCCATCGCCCTGATAGACGGTTTTTCGCCCTTTGACGTTGGAGTCCACGTTCTTTAATAGTGGACTCTTGTTCCAAACTGGAACAACACTCAACCCTATCTCGGTCTATTCTTTTGATTTATAAGGGATTTTGCCGATTTCGGCCTATTGGTTAAAAAATGAGCTGATTTAACAAAAATTTAACGCGAATTTTAACAAAATATTAACGTTTACAATTTCAGGTGGCACTTTTCGGGGAAATGTGCGCGGAACCCCTATTTGTTTATTTTTCTAAATACATTCAAATATGTATCCGCTCATGAATTAATTCTTAGAAAAACTCATCGAGCATCAAATGAAACTGCAATTTATTCATATCAGGATTATCAATACCATATTTTTGAAAAAGCCGTTTCTGTAATGAAGGAGAAAACTCACCGAGGCAGTTCCATAGGATGGCAAGATCCTGGTATCGGTCTGCGATTCCGACTCGTCCAACATCAATACAACCTATTAATTTCCCCTCGTCAAAAATAAGGTTATCAAGTGAGAAATCACCATGAGTGACGACTGAATCCGGTGAGAATGGCAAAAGTTTATGCATTTCTTTCCAGACTTGTTCAACAGGCCAGCCATTACGCTCGTCATCAAAATCACTCGCATCAACCAAACCGTTATTCATTCGTGATTGCGCCTGAGCGAGACGAAATACGCGATCGCTGTTAAAAGGACAATTACAAACAGGAATCGAATGCAACCGGCGCAGGAACACTGCCAGCGCATCAACAATATTTTCACCTGAATCAGGATATTCTTCTAATACCTGGAATGCTGTTTTCCCGGGGATCGCAGTGGTGAGTAACCATGCATCATCAGGAGTACGGATAAAATGCTTGATGGTCGGAAGAGGCATAAATTCCGTCAGCCAGTTTAGTCTGACCATCTCATCTGTAACATCATTGGCAACGCTACCTTTGCCATGTTTCAGAAACAACTCTGGCGCATCGGGCTTCCCATACAATCGATAGATTGTCGCACCTGATTGCCCGACATTATCGCGAGCCCATTTATACCCATATAAATCAGCATCCATGTTGGAATTTAATCGCGGCCTAGAGCAAGACGTTTCCCGTTGAATATGGCTCATAACACCCCTTGTATTACTGTTTATGTAAGCAGACAGTTTTATTGTTCATGACCAAAATCCCTTAACGTGAGTTTTCGTTCCACTGAGCGTCAGACCCCGTAGAAAAGATCAAAGGATCTTCTTGAGATCCTTTTTTTCTGCGCGTAATCTGCTGCTTGCAAACAAAAAAACCACCGCTACCAGCGGTGGTTTGTTTGCCGGATCAAGAGCTACCAACTCTTTTTCCGAAGGTAACTGGCTTCAGCAGAGCGCAGATACCAAATACTGTCCTTCTAGTGTAGCCGTAGTTAGGCCACCACTTCAAGAACTCTGTAGCACCGCCTACATACCTCGCTCTGCTAATCCTGTTACCAGTGGCTGCTGCCAGTGGCGATAAGTCGTGTCTTACCGGGTTGGACTCAAGACGATAGTTACCGGATAAGGCGCAGCGGTCGGGCTGAACGGGGGGTTCGTGCACACAGCCCAGCTTGGAGCGAACGACCTACACCGAACTGAGATACCTACAGCGTGAGCTATGAGAAAGCGCCACGCTTCCCGAAGGGAGAAAGGCGGACAGGTATCCGGTAAGCGGCAGGGTCGGAACAGGAGAGCGCACGAGGGAGCTTCCAGGGGGAAACGCCTGGTATCTTTATAGTCCTGTCGGGTTTCGCCACCTCTGACTTGAGCGTCGATTTTTGTGATGCTCGTCAGGGGGGCGGAGCCTATGGAAAAACGCCAGCAACGCGGCCTTTTTACGGTTCCTGGCCTTTTGCTGGCCTTTTGCTCACATGTTCTTTCCTGCGTTATCCCCTGATTCTGTGGATAACCGTATTACCGCCTTTGAGTGAGCTGATACCGCTCGCCGCAGCCGAACGACCGAGCGCAGCGAGTCAGTGAGCGAGGAAGCGGAAGAGCGCCTGATGCGGTATTTTCTCCTTACGCATCTGTGCGGTATTTCACACCGCATATATGGTGCACTCTCAGTACAATCTGCTCTGATGCCGCATAGTTAAGCCAGTATACACTCCGCTATCGCTACGTGACTGGGTCATGGCTG


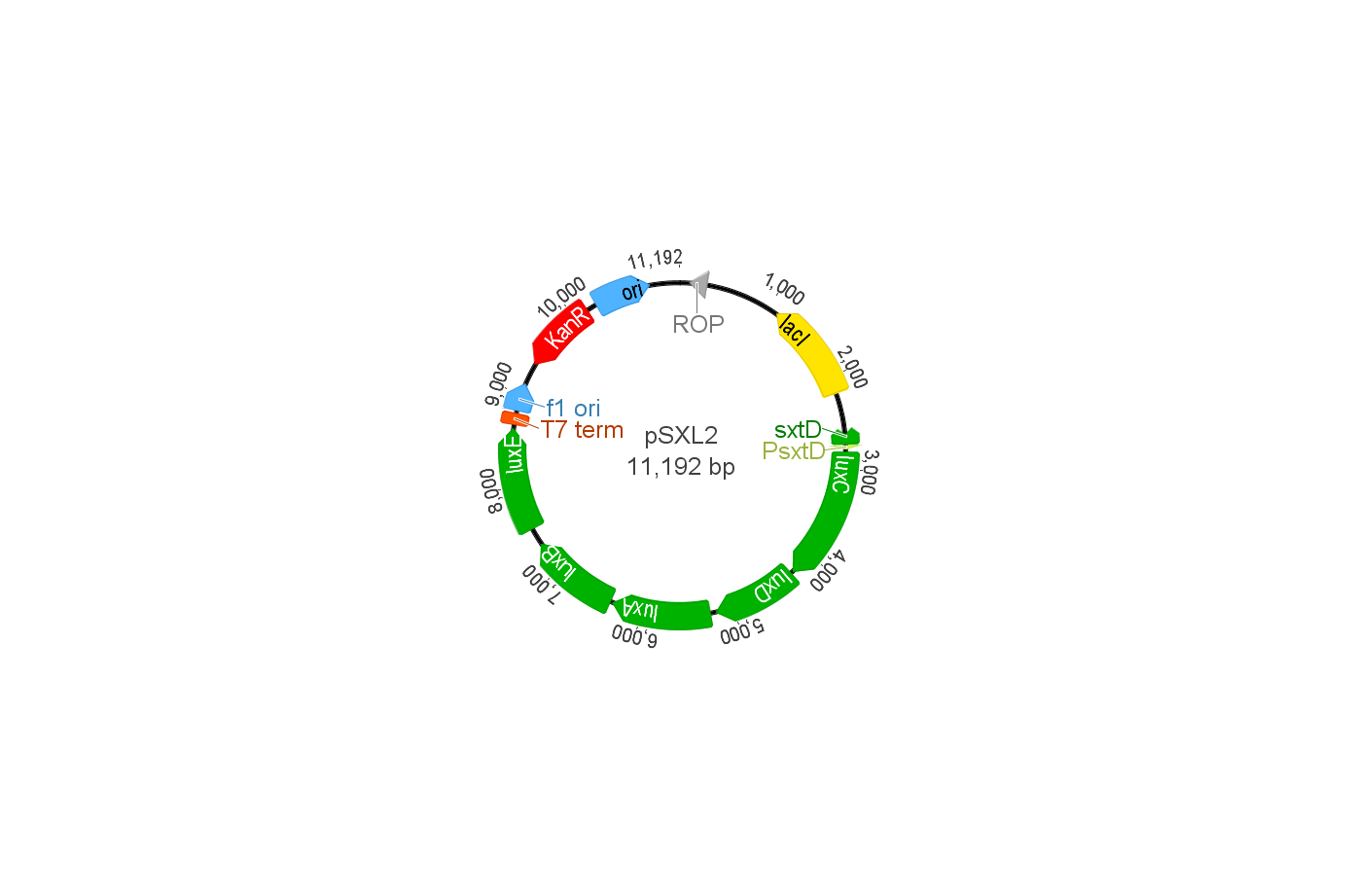


>pSXL2_nucl

CGCCCCGACACCCGCCAACACCCGCTGACGCGCCCTGACGGGCTTGTCTGCTCCCGGCATCCGCTTACAGACAAGCTGTGACCGTCTCCGGGAGCTGCATGTGTCAGAGGTTTTCACCGTCATCACCGAAACGCGCGAGGCAGCTGCGGTAAAGCTCATCAGCGTGGTCGTGAAGCGATTCACAGATGTCTGCCTGTTCATCCGCGTCCAGCTCGTTGAGTTTCTCCAGAAGCGTTAATGTCTGGCTTCTGATAAAGCGGGCCATGTTAAGGGCGGTTTTTTCCTGTTTGGTCACTGATGCCTCCGTGTAAGGGGGATTTCTGTTCATGGGGGTAATGATACCGATGAAACGAGAGAGGATGCTCACGATACGGGTTACTGATGATGAACATGCCCGGTTACTGGAACGTTGTGAGGGTAAACAACTGGCGGTATGGATGCGGCGGGACCAGAGAAAAATCACTCAGGGTCAATGCCAGCGCTTCGTTAATACAGATGTAGGTGTTCCACAGGGTAGCCAGCAGCATCCTGCGATGCAGATCCGGAACATAATGGTGCAGGGCGCTGACTTCCGCGTTTCCAGACTTTACGAAACACGGAAACCGAAGACCATTCATGTTGTTGCTCAGGTCGCAGACGTTTTGCAGCAGCAGTCGCTTCACGTTCGCTCGCGTATCGGTGATTCATTCTGCTAACCAGTAAGGCAACCCCGCCAGCCTAGCCGGGTCCTCAACGACAGGAGCACGATCATGCGCACCCGTGGGGCCGCCATGCCGGCGATAATGGCCTGCTTCTCGCCGAAACGTTTGGTGGCGGGACCAGTGACGAAGGCTTGAGCGAGGGCGTGCAAGATTCCGAATACCGCAAGCGACAGGCCGATCATCGTCGCGCTCCAGCGAAAGCGGTCCTCGCCGAAAATGACCCAGAGCGCTGCCGGCACCTGTCCTACGAGTTGCATGATAAAGAAGACAGTCATAAGTGCGGCGACGATAGTCATGCCCCGCGCCCACCGGAAGGAGCTGACTGGGTTGAAGGCTCTCAAGGGCATCGGTCGAGATCCCGGTGCCTAATGAGTGAGCTAACTTACATTAATTGCGTTGCGCTCACTGCCCGCTTTCCAGTCGGGAAACCTGTCGTGCCAGCTGCATTAATGAATCGGCCAACGCGCGGGGAGAGGCGGTTTGCGTATTGGGCGCCAGGGTGGTTTTTCTTTTCACCAGTGAGACGGGCAACAGCTGATTGCCCTTCACCGCCTGGCCCTGAGAGAGTTGCAGCAAGCGGTCCACGCTGGTTTGCCCCAGCAGGCGAAAATCCTGTTTGATGGTGGTTAACGGCGGGATATAACATGAGCTGTCTTCGGTATCGTCGTATCCCACTACCGAGATATCCGCACCAACGCGCAGCCCGGACTCGGTAATGGCGCGCATTGCGCCCAGCGCCATCTGATCGTTGGCAACCAGCATCGCAGTGGGAACGATGCCCTCATTCAGCATTTGCATGGTTTGTTGAAAACCGGACATGGCACTCCAGTCGCCTTCCCGTTCCGCTATCGGCTGAATTTGATTGCGAGTGAGATATTTATGCCAGCCAGCCAGACGCAGACGCGCCGAGACAGAACTTAATGGGCCCGCTAACAGCGCGATTTGCTGGTGACCCAATGCGACCAGATGCTCCACGCCCAGTCGCGTACCGTCTTCATGGGAGAAAATAATACTGTTGATGGGTGTCTGGTCAGAGACATCAAGAAATAACGCCGGAACATTAGTGCAGGCAGCTTCCACAGCAATGGCATCCTGGTCATCCAGCGGATAGTTAATGATCAGCCCACTGACGCGTTGCGCGAGAAGATTGTGCACCGCCGCTTTACAGGCTTCGACGCCGCTTCGTTCTACCATCGACACCACCACGCTGGCACCCAGTTGATCGGCGCGAGATTTAATCGCCGCGACAATTTGCGACGGCGCGTGCAGGGCCAGACTGGAGGTGGCAACGCCAATCAGCAACGACTGTTTGCCCGCCAGTTGTTGTGCCACGCGGTTGGGAATGTAATTCAGCTCCGCCATCGCCGCTTCCACTTTTTCCCGCGTTTTCGCAGAAACGTGGCTGGCCTGGTTCACCACGCGGGAAACGGTCTGATAAGAGACACCGGCATACTCTGCGACATCGTATAACGTTACTGGTTTCACATTCACCACCCTGAATTGACTCTCTTCCGGGCGCTATCATGCCATACCGCGAAAGGTTTTGCGCCATTCGATGGTGTCCGGGATCTCGACGCTCTCCCTTATGCGACTCCTGCATTAGGAAGCAGCCCAGTAGTAGGTTGAGGCCGTTGAGCACCGCCGCCGCAAGGAATGGTGCATGCAAGGAGATGGCGCCCAACAGTCCCCCGGCCACGGGGCCTGCCACCATACCCACGCCGAAACAAGCGCTCATGAGCCCGAAGTGGCGAGCCCGATCTTCCCCATCGGTGATGTCGGCGATATAGGCGCCAGCAACCGCACCTGTGGCGCCGGTGATGCCGGCCACGATGCGTCCGGCGTAGAGGATCGAGATCTCGATCCCGCGAAATCATAGATAATTCTAAAGACGAGTGATATTCCTGCCCATCCCAATTCCTTAAAGAAATTAGCTTTGTATTTAATCTCATGTAAAGGATAGCGAATTTCCCAAAGCCAGAATATCATACCCGTAATTAAAATTACAGTCCAATCTCTTAATAGGATTAATATTGTATCTATCATTTTGTATCTATCATTGGTAACCTTAGTGTATACTAGTCAAGTATACTCTTATTAACAAATTTCCACAAGACAACTCATGACTAAAAAAATTTCATTCATTATTAACGGCCAGGTTGAAATCTTTCCCGAAAGTGATGATTTAGTGCAATCCATTAATTTTGGTGATAATAGTGTTTACCTGCCAATATTGAATGACTCTCATGTAAAAAACATTATTGATTGTAATGGAAATAACGAATTACGGTTGCATAACATTGTCAATTTTCTCTATACGGTAGGGCAAAGATGGAAAAATGAAGAATACTCAAGACGCAGGACATACATTCGTGACTTAAAAAAATATATGGGATATTCAGAAGAAATGGCTAAGCTAGAGGCCAATTGGATATCTATGATTTTATGTTCTAAAGGCGGCCTTTATGATGTTGTAGAAAATGAACTTGGTTCTCGCCATATCATGGATGAATGGCTACCTCAGGATGAAAGTTATGTTCGGGCTTTTCCGAAAGGTAAATCTGTACATCTGTTGGCAGGTAATGTTCCATTATCTGGGATCATGTCTATATTACGCGCAATTTTAACTAAGAATCAGTGTATTATAAAAACATCGTCAACCGATCCTTTTACCGCTAATGCATTAGCGTTAAGTTTTATTGATGTAGACCCTAATCATCCGATAACGCGCTCTTTATCTGTTATATATTGGCCCCACCAAGGTGATACATCACTCGCAAAAGAAATTATGCGACATGCGGATGTTATTGTCGCTTGGGGAGGGCCAGATGCGATTAATTGGGCGGTAGAGCATGCGCCATCTTATGCTGATGTGATTAAATTTGGTTCTAAAAAGAGTCTTTGCATTATCGATAATCCTGTTGATTTGACGTCCGCAGCGACAGGTGCGGCTCATGATGTTTGTTTTTACGATCAGCGAGCTTGTTTTTCTGCCCAAAACATATATTACATGGGAAATCATTATGAGGAATTTAAGTTAGCGTTGATAGAAAAACTTAATCTATATGCGCATATATTACCGAATGCCAAAAAAGATTTTGATGAAAAGGCGGCCTATTCTTTAGTTCAAAAAGAAAGCTTGTTTGCTGGATTAAAAGTAGAGGTGGATATTCATCAACGTTGGATGATTATTGAGTCAAATGCAGGTGTGGAATTTAATCAACCACTTGGCAGATGTGTGTACCTTCATCACGTCGATAATATTGAGCAAATATTGCCTTATGTTCAAAAAAATAAGACGCAAACCATATCTATTTTTCCTTGGGAGTCATCATTTAAATATCGAGATGCGTTAGCATTAAAAGGTGCGGAAAGGATTGTAGAAGCAGGAATGAATAACATATTTCGAGTTGGTGGATCTCATGACGGAATGAGACCGTTGCAACGATTAGTGACATATATTTCTCATGAAAGGCCATCTAACTATACGGCTAAGGATGTTGCGGTTGAAATAGAACAGACTCGATTCCTGGAAGAAGATAAGTTCCTTGTATTTGTCCCATAATAGGTAAAAGTATGGAAAATGAATCAAAATATAAAACCATCGACCACGTTATTTGTGTTGAAGGAAATAAAAAAATTCATGTTTGGGAAACGCTGCCAGAAGAAAACAGCCCAAAGAGAAAGAATGCCATTATTATTGCGTCTGGTTTTGCCCGCAGGATGGATCATTTTGCTGGTCTGGCGGAATATTTATCGCGGAATGGATTTCATGTGATCCGCTATGATTCGCTTCACCACGTTGGATTGAGTTCAGGGACAATTGATGAATTTACAATGTCTATAGGAAAGCAGAGCTTGTTAGCAGTGGTTGATTGGTTAACTACACGAAAAATAAATAACTTCGGTATGTTGGCTTCAAGCTTATCTGCGCGGATAGCTTATGCAAGCCTATCTGAAATCAATGCTTCGTTTTTAATCACCGCAGTCGGTGTTGTTAACTTAAGATATTCTCTTGAAAGAGCTTTAGGGTTTGATTATCTCAGTCTACCCATTAATGAATTGCCGGATAATCTAGATTTTGAAGGCCATAAATTGGGTGCTGAAGTCTTTGCGAGAGATTGTCTTGATTTTGGTTGGGAAGATTTAGCTTCTACAATTAATAACATGATGTATCTTGATATACCGTTTATTGCTTTTACTGCAAATAACGATAATTGGGTCAAGCAAGATGAAGTTATCACATTGTTATCAAATATTCGTAGTAATCGATGCAAGATATATTCTTTGTTAGGAAGTTCGCATGACTTGAGTGAAAATTTAGTGGTCCTGCGCAATTTTTATCAATCGGTTACGAAAGCCGCTATCGCGATGGATAATGATCATCTGGATATTGATGTTGATATTACTGAACCGTCATTTGAACATTTAACTATTGCGACAGTCAATGAACGCCGAATGAGAATTGAGATTGAAAATCAAGCAATTTCTCTGTCTTAAAATCTATTGAGATATTCTATCACTCAAATAGCAATATAAGGACTCTCTATGAAATTTGGAAACTTTTTGCTTACATACCAACCTCCCCAATTTTCTCAAACAGAGGTAATGAAACGTTTGGTTAAATTAGGTCGCATCTCTGAGGAGTGTGGTTTTGATACCGTATGGTTACTGGAGCATCATTTCACGGAGTTTGGTTTGCTTGGTAACCCTTATGTCGCTGCTGCATATTTACTTGGCGCGACTAAAAAATTGAATGTAGGAACTGCCGCTATTGTTCTTCCCACAGCCCATCCAGTACGCCAACTTGAAGATGTGAATTTATTGGATCAAATGTCAAAAGGACGATTTCGGTTTGGTATTTGCCGAGGGCTTTACAACAAGGACTTTCGCGTATTCGGCACAGATATGAATAACAGTCGCGCCTTAGCGGAATGCTGGTACGGGCTGATAAAGAATGGCATGACAGAGGGATATATGGAAGCTGATAATGAACATATCAAGTTCCATAAGGTAAAAGTAAACCCCGCGGCGTATAGCAGAGGTGGCGCACCGGTTTATGTGGTGGCTGAATCAGCTTCGACGACTGAGTGGGCTGCTCAATTTGGCCTACCGATGATATTAAGTTGGATTATAAATACTAACGAAAAGAAAGCACAACTTGAGCTTTATAATGAAGTGGCTCAAGAATATGGGCACGATATTCATAATATCGACCATTGCTTATCATATATAACATCTGTAGATCATGACTCAATTAAAGCGAAAGAGATTTGCCGGAAATTTCTGGGGCATTGGTATGATTCTTATGTGAATGCTACGACTATTTTTGATGATTCAGACCAAACAAGAGGTTATGATTTCAATAAAGGGCAGTGGCGTGACTTTGTATTAAAAGGACATAAAGATACTAATCGCCGTATTGATTACAGTTACGAAATCAATCCCGTGGGAACGCCGCAGGAATGTATTGACATAATTCAAAAAGACATTGATGCTACAGGAATATCAAATATTTGTTGTGGATTTGAAGCTAATGGAACAGTAGACGAAATTATTGCTTCCATGAAGCTCTTCCAGTCTGATGTCATGCCATTTCTTAAAGAAAAACAACGTTCGCTATTATATTAGCTAAGGAGAAAGAAATGAAATTTGGATTGTTCTTCCTTAACTTCATCAATTCAACAACTGTTCAAGAACAAAGTATAGTTCGCATGCAGGAAATAACGGAGTATGTTGATAAGTTGAATTTTGAACAGATTTTAGTGTATGAAAATCATTTTTCAGATAATGGTGTTGTCGGCGCTCCTCTGACTGTTTCTGGTTTTCTGCTCGGTTTAACAGAGAAAATTAAAATTGGTTCATTAAATCACATCATTACAACTCATCATCCTGTCGCCATAGCGGAGGAAGCTTGCTTATTGGATCAGTTAAGTGAAGGGAGATTTATTTTAGGGTTTAGTGATTGCGAAAAAAAAGATGAAATGCATTTTTTTAATCGCCCGGTTGAATATCAACAGCAACTATTTGAAGAGTGTTATGAAATCATTAACGATGCTTTAACAACAGGCTATTGTAATCCAGATAACGATTTTTATAGCTTCCCTAAAATATCTGTAAATCCCCATGCTTATACGCCAGGCGGACCTCGGAAATATGTAACAGCAACCAGTCATCATATTGTTGAGTGGGCGGCCAAAAAAGGTATTCCTCTCATCTTTAAGTGGGATGATTCTAATGATGTTAGATATGAATATGCTGAAAGATATAAAGCCGTTGCGGATAAATATGACGTTGACCTATCAGAGATAGACCATCAGTTAATGATATTAGTTAACTATAACGAAGATAGTAATAAAGCTAAACAAGAGACGCGTGCATTTATTAGTGATTATGTTCTTGAAATGCACCCTAATGAAAATTTCGAAAATAAACTTGAAGAAATAATTGCAGAAAACGCTGTCGGAAATTATACGGAGTGTATAACTGCGGCTAAGTTGGCAATTGAAAAGTGTGGTGCGAAAAGTGTATTGCTGTCCTTTGAACCAATGAATGATTTGATGAGCCAAAAAAATGTAATCAATATTGTTGATGATAATATTAAGAAGTACCACATGGAATATACCTAATAGATTTCGAGTTGCAGCGAGGCGGCAAGTGAACGAATCCCCAGGAGCATAGATAACTATGTGACTGGGGTGAGTGAAAGCAGCCAACAAAGCAGCAGCTTGAAAGATGAAGGGTATAAAAGAGTATGACAGCAGTGCTGCCATACTTTCTAATATTATCTTGAGGAGTAAAACAGGTATGACTTCATATGTTGATAAACAAGAAATTACAGCAAGCTCAGAAATTGATGATTTGATTTTTTCGAGCGATCCATTAGTGTGGTCTTACGACGAGCAGGAAAAAATCAGAAAGAAACTTGTGCTTGATGCATTTCGTAATCATTATAAACATTGTCGAGAATATCGTCACTACTGTCAGGCACACAAAGTAGATGACAATATTACGGAAATTGATGACATACCTGTATTCCCAACATCGGTTTTTAAGTTTACTCGCTTATTAACTTCTCAGGAAAACGAGATTGAAAGTTGGTTTACCAGTAGCGGCACGAATGGTTTAAAAAGTCAGGTGGCGCGTGACAGATTAAGTATTGAGAGACTCTTAGGCTCTGTGAGTTATGGCATGAAATATGTTGGTAGTTGGTTTGATCATCAAATAGAATTAGTCAATTTGGGACCAGATAGATTTAATGCTCATAATATTTGGTTTAAATATGTTATGAGTTTGGTGGAATTGTTATATCCTACGACATTTACCGTAACAGAAGAACGAATAGATTTTGTTAAAACATTGAATAGTCTTGAACGAATAAAAAATCAAGGGAAAGATCTTTGTCTTATTGGTTCGCCATACTTTATTTATTTACTCTGCCATTATATGAAAGATAAAAAAATCTCATTTTCTGGAGATAAAAGCCTTTATATCATAACCGGAGGCGGCTGGAAAAGTTACGAAAAAGAATCTCTGAAACGTGATGATTTCAATCATCTTTTATTTGATACTTTCAATCTCAGTGATATTAGTCAGATCCGAGATATATTTAATCAAGTTGAACTCAACACTTGTTTCTTTGAGGATGAAATGCAGCGTAAACATGTTCCGCCGTGGGTATATGCGCGAGCGCTTGATCCTGAAACGTTGAAACCTGTACCTGATGGAACGCCGGGGTTGATGAGTTATATGGATGCGTCAGCAACCAGTTATCCAGCATTTATTGTTACCGATGATGTCGGGATAATTAGCAGAGAATATGGTAAGTATCCCGGCGTGCTCGTTGAAATTTTACGTCGCGTCAATACGAGGACGCAGAAAGGGTGTGCTTTAAGCTTAACCGAAGCGTTTGATAGTTGACTCGAGCACCACCACCACCACCACTGAGATCCGGCTGCTAACAAAGCCCGAAAGGAAGCTGAGTTGGCTGCTGCCACCGCTGAGCAATAACTAGCATAACCCCTTGGGGCCTCTAAACGGGTCTTGAGGGGTTTTTTGCTGAAAGGAGGAACTATATCCGGATTGGCGAATGGGACGCGCCCTGTAGCGGCGCATTAAGCGCGGCGGGTGTGGTGGTTACGCGCAGCGTGACCGCTACACTTGCCAGCGCCCTAGCGCCCGCTCCTTTCGCTTTCTTCCCTTCCTTTCTCGCCACGTTCGCCGGCTTTCCCCGTCAAGCTCTAAATCGGGGGCTCCCTTTAGGGTTCCGATTTAGTGCTTTACGGCACCTCGACCCCAAAAAACTTGATTAGGGTGATGGTTCACGTAGTGGGCCATCGCCCTGATAGACGGTTTTTCGCCCTTTGACGTTGGAGTCCACGTTCTTTAATAGTGGACTCTTGTTCCAAACTGGAACAACACTCAACCCTATCTCGGTCTATTCTTTTGATTTATAAGGGATTTTGCCGATTTCGGCCTATTGGTTAAAAAATGAGCTGATTTAACAAAAATTTAACGCGAATTTTAACAAAATATTAACGTTTACAATTTCAGGTGGCACTTTTCGGGGAAATGTGCGCGGAACCCCTATTTGTTTATTTTTCTAAATACATTCAAATATGTATCCGCTCATGAATTAATTCTTAGAAAAACTCATCGAGCATCAAATGAAACTGCAATTTATTCATATCAGGATTATCAATACCATATTTTTGAAAAAGCCGTTTCTGTAATGAAGGAGAAAACTCACCGAGGCAGTTCCATAGGATGGCAAGATCCTGGTATCGGTCTGCGATTCCGACTCGTCCAACATCAATACAACCTATTAATTTCCCCTCGTCAAAAATAAGGTTATCAAGTGAGAAATCACCATGAGTGACGACTGAATCCGGTGAGAATGGCAAAAGTTTATGCATTTCTTTCCAGACTTGTTCAACAGGCCAGCCATTACGCTCGTCATCAAAATCACTCGCATCAACCAAACCGTTATTCATTCGTGATTGCGCCTGAGCGAGACGAAATACGCGATCGCTGTTAAAAGGACAATTACAAACAGGAATCGAATGCAACCGGCGCAGGAACACTGCCAGCGCATCAACAATATTTTCACCTGAATCAGGATATTCTTCTAATACCTGGAATGCTGTTTTCCCGGGGATCGCAGTGGTGAGTAACCATGCATCATCAGGAGTACGGATAAAATGCTTGATGGTCGGAAGAGGCATAAATTCCGTCAGCCAGTTTAGTCTGACCATCTCATCTGTAACATCATTGGCAACGCTACCTTTGCCATGTTTCAGAAACAACTCTGGCGCATCGGGCTTCCCATACAATCGATAGATTGTCGCACCTGATTGCCCGACATTATCGCGAGCCCATTTATACCCATATAAATCAGCATCCATGTTGGAATTTAATCGCGGCCTAGAGCAAGACGTTTCCCGTTGAATATGGCTCATAACACCCCTTGTATTACTGTTTATGTAAGCAGACAGTTTTATTGTTCATGACCAAAATCCCTTAACGTGAGTTTTCGTTCCACTGAGCGTCAGACCCCGTAGAAAAGATCAAAGGATCTTCTTGAGATCCTTTTTTTCTGCGCGTAATCTGCTGCTTGCAAACAAAAAAACCACCGCTACCAGCGGTGGTTTGTTTGCCGGATCAAGAGCTACCAACTCTTTTTCCGAAGGTAACTGGCTTCAGCAGAGCGCAGATACCAAATACTGTCCTTCTAGTGTAGCCGTAGTTAGGCCACCACTTCAAGAACTCTGTAGCACCGCCTACATACCTCGCTCTGCTAATCCTGTTACCAGTGGCTGCTGCCAGTGGCGATAAGTCGTGTCTTACCGGGTTGGACTCAAGACGATAGTTACCGGATAAGGCGCAGCGGTCGGGCTGAACGGGGGGTTCGTGCACACAGCCCAGCTTGGAGCGAACGACCTACACCGAACTGAGATACCTACAGCGTGAGCTATGAGAAAGCGCCACGCTTCCCGAAGGGAGAAAGGCGGACAGGTATCCGGTAAGCGGCAGGGTCGGAACAGGAGAGCGCACGAGGGAGCTTCCAGGGGGAAACGCCTGGTATCTTTATAGTCCTGTCGGGTTTCGCCACCTCTGACTTGAGCGTCGATTTTTGTGATGCTCGTCAGGGGGGCGGAGCCTATGGAAAAACGCCAGCAACGCGGCCTTTTTACGGTTCCTGGCCTTTTGCTGGCCTTTTGCTCACATGTTCTTTCCTGCGTTATCCCCTGATTCTGTGGATAACCGTATTACCGCCTTTGAGTGAGCTGATACCGCTCGCCGCAGCCGAACGACCGAGCGCAGCGAGTCAGTGAGCGAGGAAGCGGAAGAGCGCCTGATGCGGTATTTTCTCCTTACGCATCTGTGCGGTATTTCACACCGCATATATGGTGCACTCTCAGTACAATCTGCTCTGATGCCGCATAGTTAAGCCAGTATACACTCCGCTATCGCTACGTGACTGGGTCATGGCTG


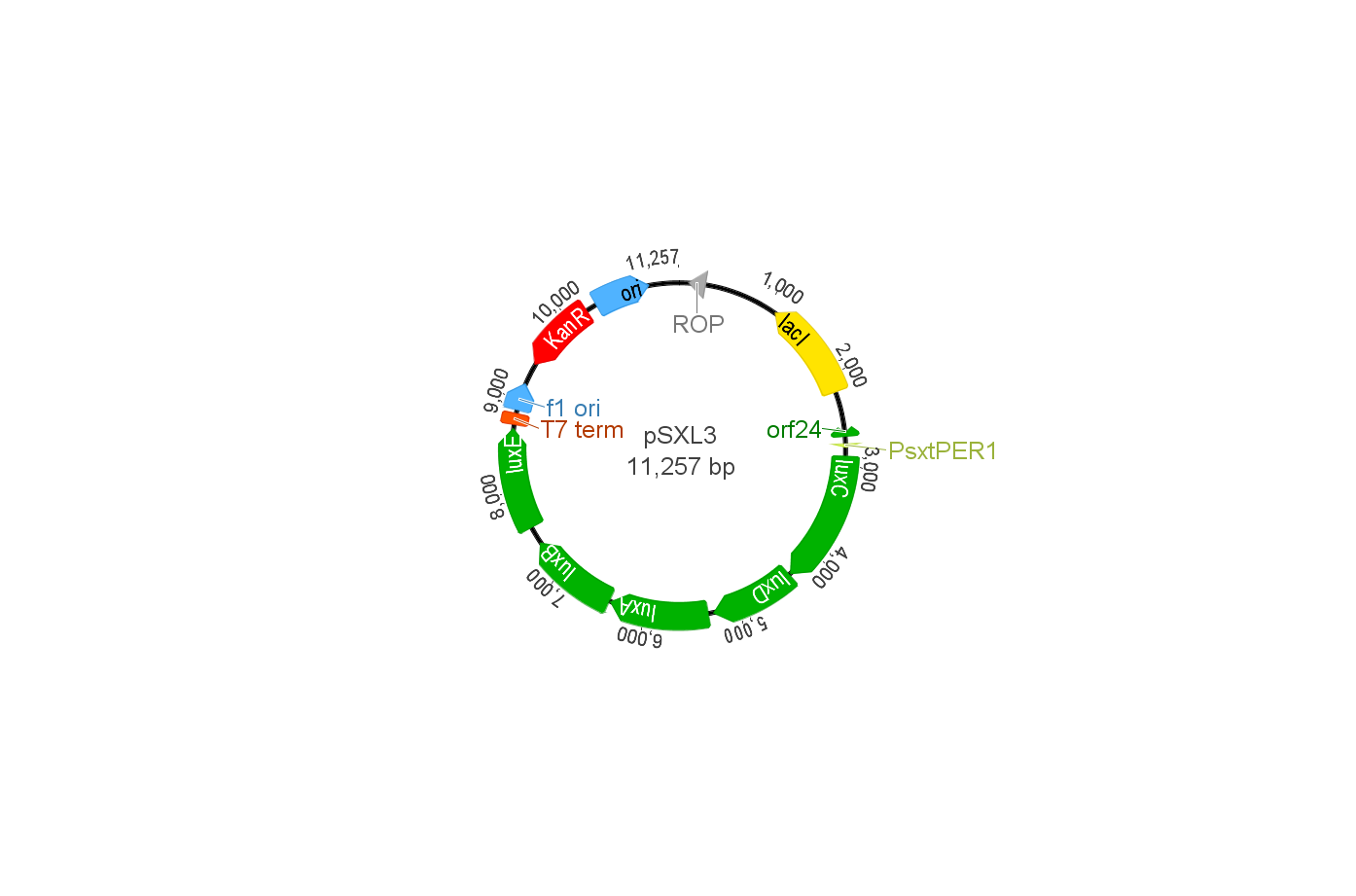


>pSXL3_nucl

CGCCCCGACACCCGCCAACACCCGCTGACGCGCCCTGACGGGCTTGTCTGCTCCCGGCATCCGCTTACAGACAAGCTGTGACCGTCTCCGGGAGCTGCATGTGTCAGAGGTTTTCACCGTCATCACCGAAACGCGCGAGGCAGCTGCGGTAAAGCTCATCAGCGTGGTCGTGAAGCGATTCACAGATGTCTGCCTGTTCATCCGCGTCCAGCTCGTTGAGTTTCTCCAGAAGCGTTAATGTCTGGCTTCTGATAAAGCGGGCCATGTTAAGGGCGGTTTTTTCCTGTTTGGTCACTGATGCCTCCGTGTAAGGGGGATTTCTGTTCATGGGGGTAATGATACCGATGAAACGAGAGAGGATGCTCACGATACGGGTTACTGATGATGAACATGCCCGGTTACTGGAACGTTGTGAGGGTAAACAACTGGCGGTATGGATGCGGCGGGACCAGAGAAAAATCACTCAGGGTCAATGCCAGCGCTTCGTTAATACAGATGTAGGTGTTCCACAGGGTAGCCAGCAGCATCCTGCGATGCAGATCCGGAACATAATGGTGCAGGGCGCTGACTTCCGCGTTTCCAGACTTTACGAAACACGGAAACCGAAGACCATTCATGTTGTTGCTCAGGTCGCAGACGTTTTGCAGCAGCAGTCGCTTCACGTTCGCTCGCGTATCGGTGATTCATTCTGCTAACCAGTAAGGCAACCCCGCCAGCCTAGCCGGGTCCTCAACGACAGGAGCACGATCATGCGCACCCGTGGGGCCGCCATGCCGGCGATAATGGCCTGCTTCTCGCCGAAACGTTTGGTGGCGGGACCAGTGACGAAGGCTTGAGCGAGGGCGTGCAAGATTCCGAATACCGCAAGCGACAGGCCGATCATCGTCGCGCTCCAGCGAAAGCGGTCCTCGCCGAAAATGACCCAGAGCGCTGCCGGCACCTGTCCTACGAGTTGCATGATAAAGAAGACAGTCATAAGTGCGGCGACGATAGTCATGCCCCGCGCCCACCGGAAGGAGCTGACTGGGTTGAAGGCTCTCAAGGGCATCGGTCGAGATCCCGGTGCCTAATGAGTGAGCTAACTTACATTAATTGCGTTGCGCTCACTGCCCGCTTTCCAGTCGGGAAACCTGTCGTGCCAGCTGCATTAATGAATCGGCCAACGCGCGGGGAGAGGCGGTTTGCGTATTGGGCGCCAGGGTGGTTTTTCTTTTCACCAGTGAGACGGGCAACAGCTGATTGCCCTTCACCGCCTGGCCCTGAGAGAGTTGCAGCAAGCGGTCCACGCTGGTTTGCCCCAGCAGGCGAAAATCCTGTTTGATGGTGGTTAACGGCGGGATATAACATGAGCTGTCTTCGGTATCGTCGTATCCCACTACCGAGATATCCGCACCAACGCGCAGCCCGGACTCGGTAATGGCGCGCATTGCGCCCAGCGCCATCTGATCGTTGGCAACCAGCATCGCAGTGGGAACGATGCCCTCATTCAGCATTTGCATGGTTTGTTGAAAACCGGACATGGCACTCCAGTCGCCTTCCCGTTCCGCTATCGGCTGAATTTGATTGCGAGTGAGATATTTATGCCAGCCAGCCAGACGCAGACGCGCCGAGACAGAACTTAATGGGCCCGCTAACAGCGCGATTTGCTGGTGACCCAATGCGACCAGATGCTCCACGCCCAGTCGCGTACCGTCTTCATGGGAGAAAATAATACTGTTGATGGGTGTCTGGTCAGAGACATCAAGAAATAACGCCGGAACATTAGTGCAGGCAGCTTCCACAGCAATGGCATCCTGGTCATCCAGCGGATAGTTAATGATCAGCCCACTGACGCGTTGCGCGAGAAGATTGTGCACCGCCGCTTTACAGGCTTCGACGCCGCTTCGTTCTACCATCGACACCACCACGCTGGCACCCAGTTGATCGGCGCGAGATTTAATCGCCGCGACAATTTGCGACGGCGCGTGCAGGGCCAGACTGGAGGTGGCAACGCCAATCAGCAACGACTGTTTGCCCGCCAGTTGTTGTGCCACGCGGTTGGGAATGTAATTCAGCTCCGCCATCGCCGCTTCCACTTTTTCCCGCGTTTTCGCAGAAACGTGGCTGGCCTGGTTCACCACGCGGGAAACGGTCTGATAAGAGACACCGGCATACTCTGCGACATCGTATAACGTTACTGGTTTCACATTCACCACCCTGAATTGACTCTCTTCCGGGCGCTATCATGCCATACCGCGAAAGGTTTTGCGCCATTCGATGGTGTCCGGGATCTCGACGCTCTCCCTTATGCGACTCCTGCATTAGGAAGCAGCCCAGTAGTAGGTTGAGGCCGTTGAGCACCGCCGCCGCAAGGAATGGTGCATGCAAGGAGATGGCGCCCAACAGTCCCCCGGCCACGGGGCCTGCCACCATACCCACGCCGAAACAAGCGCTCATGAGCCCGAAGTGGCGAGCCCGATCTTCCCCATCGGTGATGTCGGCGATATAGGCGCCAGCAACCGCACCTGTGGCGCCGGTGATGCCGGCCACGATGCGTCCGGCGTAGAGGATCGAGATCTCGATCCCGCGAAATGCTGACAACATTGATAATCCAATCCTCCATCTCTTGGCGAAATTGTTCGAGATTTATCATAATATTTGATTTTTCCTCGCTTGTAGTAATTGGTAGTTGAAGCATTGGCAGCTGCGGTGTGCTATGTAATTTCTGTATAAAACATTGCTTGGTTTTTATTCAGATGTAAGCATCTGTTTTATTTCCTTGCAATTCAAGGGATTTATGAGTTACAATTACATGACTCACATTATTCGCGTTATTAGCGATATACCGTACAGCCAAAGATTTCATTATAGCACTACGCCCAGACCTAAGGAAATTTTAAGTTGAGATGACTAAAAAAATTTCATTCATTATTAACGGCCAGGTTGAAATCTTTCCCGAAAGTGATGATTTAGTGCAATCCATTAATTTTGGTGATAATAGTGTTTACCTGCCAATATTGAATGACTCTCATGTAAAAAACATTATTGATTGTAATGGAAATAACGAATTACGGTTGCATAACATTGTCAATTTTCTCTATACGGTAGGGCAAAGATGGAAAAATGAAGAATACTCAAGACGCAGGACATACATTCGTGACTTAAAAAAATATATGGGATATTCAGAAGAAATGGCTAAGCTAGAGGCCAATTGGATATCTATGATTTTATGTTCTAAAGGCGGCCTTTATGATGTTGTAGAAAATGAACTTGGTTCTCGCCATATCATGGATGAATGGCTACCTCAGGATGAAAGTTATGTTCGGGCTTTTCCGAAAGGTAAATCTGTACATCTGTTGGCAGGTAATGTTCCATTATCTGGGATCATGTCTATATTACGCGCAATTTTAACTAAGAATCAGTGTATTATAAAAACATCGTCAACCGATCCTTTTACCGCTAATGCATTAGCGTTAAGTTTTATTGATGTAGACCCTAATCATCCGATAACGCGCTCTTTATCTGTTATATATTGGCCCCACCAAGGTGATACATCACTCGCAAAAGAAATTATGCGACATGCGGATGTTATTGTCGCTTGGGGAGGGCCAGATGCGATTAATTGGGCGGTAGAGCATGCGCCATCTTATGCTGATGTGATTAAATTTGGTTCTAAAAAGAGTCTTTGCATTATCGATAATCCTGTTGATTTGACGTCCGCAGCGACAGGTGCGGCTCATGATGTTTGTTTTTACGATCAGCGAGCTTGTTTTTCTGCCCAAAACATATATTACATGGGAAATCATTATGAGGAATTTAAGTTAGCGTTGATAGAAAAACTTAATCTATATGCGCATATATTACCGAATGCCAAAAAAGATTTTGATGAAAAGGCGGCCTATTCTTTAGTTCAAAAAGAAAGCTTGTTTGCTGGATTAAAAGTAGAGGTGGATATTCATCAACGTTGGATGATTATTGAGTCAAATGCAGGTGTGGAATTTAATCAACCACTTGGCAGATGTGTGTACCTTCATCACGTCGATAATATTGAGCAAATATTGCCTTATGTTCAAAAAAATAAGACGCAAACCATATCTATTTTTCCTTGGGAGTCATCATTTAAATATCGAGATGCGTTAGCATTAAAAGGTGCGGAAAGGATTGTAGAAGCAGGAATGAATAACATATTTCGAGTTGGTGGATCTCATGACGGAATGAGACCGTTGCAACGATTAGTGACATATATTTCTCATGAAAGGCCATCTAACTATACGGCTAAGGATGTTGCGGTTGAAATAGAACAGACTCGATTCCTGGAAGAAGATAAGTTCCTTGTATTTGTCCCATAATAGGTAAAAGTATGGAAAATGAATCAAAATATAAAACCATCGACCACGTTATTTGTGTTGAAGGAAATAAAAAAATTCATGTTTGGGAAACGCTGCCAGAAGAAAACAGCCCAAAGAGAAAGAATGCCATTATTATTGCGTCTGGTTTTGCCCGCAGGATGGATCATTTTGCTGGTCTGGCGGAATATTTATCGCGGAATGGATTTCATGTGATCCGCTATGATTCGCTTCACCACGTTGGATTGAGTTCAGGGACAATTGATGAATTTACAATGTCTATAGGAAAGCAGAGCTTGTTAGCAGTGGTTGATTGGTTAACTACACGAAAAATAAATAACTTCGGTATGTTGGCTTCAAGCTTATCTGCGCGGATAGCTTATGCAAGCCTATCTGAAATCAATGCTTCGTTTTTAATCACCGCAGTCGGTGTTGTTAACTTAAGATATTCTCTTGAAAGAGCTTTAGGGTTTGATTATCTCAGTCTACCCATTAATGAATTGCCGGATAATCTAGATTTTGAAGGCCATAAATTGGGTGCTGAAGTCTTTGCGAGAGATTGTCTTGATTTTGGTTGGGAAGATTTAGCTTCTACAATTAATAACATGATGTATCTTGATATACCGTTTATTGCTTTTACTGCAAATAACGATAATTGGGTCAAGCAAGATGAAGTTATCACATTGTTATCAAATATTCGTAGTAATCGATGCAAGATATATTCTTTGTTAGGAAGTTCGCATGACTTGAGTGAAAATTTAGTGGTCCTGCGCAATTTTTATCAATCGGTTACGAAAGCCGCTATCGCGATGGATAATGATCATCTGGATATTGATGTTGATATTACTGAACCGTCATTTGAACATTTAACTATTGCGACAGTCAATGAACGCCGAATGAGAATTGAGATTGAAAATCAAGCAATTTCTCTGTCTTAAAATCTATTGAGATATTCTATCACTCAAATAGCAATATAAGGACTCTCTATGAAATTTGGAAACTTTTTGCTTACATACCAACCTCCCCAATTTTCTCAAACAGAGGTAATGAAACGTTTGGTTAAATTAGGTCGCATCTCTGAGGAGTGTGGTTTTGATACCGTATGGTTACTGGAGCATCATTTCACGGAGTTTGGTTTGCTTGGTAACCCTTATGTCGCTGCTGCATATTTACTTGGCGCGACTAAAAAATTGAATGTAGGAACTGCCGCTATTGTTCTTCCCACAGCCCATCCAGTACGCCAACTTGAAGATGTGAATTTATTGGATCAAATGTCAAAAGGACGATTTCGGTTTGGTATTTGCCGAGGGCTTTACAACAAGGACTTTCGCGTATTCGGCACAGATATGAATAACAGTCGCGCCTTAGCGGAATGCTGGTACGGGCTGATAAAGAATGGCATGACAGAGGGATATATGGAAGCTGATAATGAACATATCAAGTTCCATAAGGTAAAAGTAAACCCCGCGGCGTATAGCAGAGGTGGCGCACCGGTTTATGTGGTGGCTGAATCAGCTTCGACGACTGAGTGGGCTGCTCAATTTGGCCTACCGATGATATTAAGTTGGATTATAAATACTAACGAAAAGAAAGCACAACTTGAGCTTTATAATGAAGTGGCTCAAGAATATGGGCACGATATTCATAATATCGACCATTGCTTATCATATATAACATCTGTAGATCATGACTCAATTAAAGCGAAAGAGATTTGCCGGAAATTTCTGGGGCATTGGTATGATTCTTATGTGAATGCTACGACTATTTTTGATGATTCAGACCAAACAAGAGGTTATGATTTCAATAAAGGGCAGTGGCGTGACTTTGTATTAAAAGGACATAAAGATACTAATCGCCGTATTGATTACAGTTACGAAATCAATCCCGTGGGAACGCCGCAGGAATGTATTGACATAATTCAAAAAGACATTGATGCTACAGGAATATCAAATATTTGTTGTGGATTTGAAGCTAATGGAACAGTAGACGAAATTATTGCTTCCATGAAGCTCTTCCAGTCTGATGTCATGCCATTTCTTAAAGAAAAACAACGTTCGCTATTATATTAGCTAAGGAGAAAGAAATGAAATTTGGATTGTTCTTCCTTAACTTCATCAATTCAACAACTGTTCAAGAACAAAGTATAGTTCGCATGCAGGAAATAACGGAGTATGTTGATAAGTTGAATTTTGAACAGATTTTAGTGTATGAAAATCATTTTTCAGATAATGGTGTTGTCGGCGCTCCTCTGACTGTTTCTGGTTTTCTGCTCGGTTTAACAGAGAAAATTAAAATTGGTTCATTAAATCACATCATTACAACTCATCATCCTGTCGCCATAGCGGAGGAAGCTTGCTTATTGGATCAGTTAAGTGAAGGGAGATTTATTTTAGGGTTTAGTGATTGCGAAAAAAAAGATGAAATGCATTTTTTTAATCGCCCGGTTGAATATCAACAGCAACTATTTGAAGAGTGTTATGAAATCATTAACGATGCTTTAACAACAGGCTATTGTAATCCAGATAACGATTTTTATAGCTTCCCTAAAATATCTGTAAATCCCCATGCTTATACGCCAGGCGGACCTCGGAAATATGTAACAGCAACCAGTCATCATATTGTTGAGTGGGCGGCCAAAAAAGGTATTCCTCTCATCTTTAAGTGGGATGATTCTAATGATGTTAGATATGAATATGCTGAAAGATATAAAGCCGTTGCGGATAAATATGACGTTGACCTATCAGAGATAGACCATCAGTTAATGATATTAGTTAACTATAACGAAGATAGTAATAAAGCTAAACAAGAGACGCGTGCATTTATTAGTGATTATGTTCTTGAAATGCACCCTAATGAAAATTTCGAAAATAAACTTGAAGAAATAATTGCAGAAAACGCTGTCGGAAATTATACGGAGTGTATAACTGCGGCTAAGTTGGCAATTGAAAAGTGTGGTGCGAAAAGTGTATTGCTGTCCTTTGAACCAATGAATGATTTGATGAGCCAAAAAAATGTAATCAATATTGTTGATGATAATATTAAGAAGTACCACATGGAATATACCTAATAGATTTCGAGTTGCAGCGAGGCGGCAAGTGAACGAATCCCCAGGAGCATAGATAACTATGTGACTGGGGTGAGTGAAAGCAGCCAACAAAGCAGCAGCTTGAAAGATGAAGGGTATAAAAGAGTATGACAGCAGTGCTGCCATACTTTCTAATATTATCTTGAGGAGTAAAACAGGTATGACTTCATATGTTGATAAACAAGAAATTACAGCAAGCTCAGAAATTGATGATTTGATTTTTTCGAGCGATCCATTAGTGTGGTCTTACGACGAGCAGGAAAAAATCAGAAAGAAACTTGTGCTTGATGCATTTCGTAATCATTATAAACATTGTCGAGAATATCGTCACTACTGTCAGGCACACAAAGTAGATGACAATATTACGGAAATTGATGACATACCTGTATTCCCAACATCGGTTTTTAAGTTTACTCGCTTATTAACTTCTCAGGAAAACGAGATTGAAAGTTGGTTTACCAGTAGCGGCACGAATGGTTTAAAAAGTCAGGTGGCGCGTGACAGATTAAGTATTGAGAGACTCTTAGGCTCTGTGAGTTATGGCATGAAATATGTTGGTAGTTGGTTTGATCATCAAATAGAATTAGTCAATTTGGGACCAGATAGATTTAATGCTCATAATATTTGGTTTAAATATGTTATGAGTTTGGTGGAATTGTTATATCCTACGACATTTACCGTAACAGAAGAACGAATAGATTTTGTTAAAACATTGAATAGTCTTGAACGAATAAAAAATCAAGGGAAAGATCTTTGTCTTATTGGTTCGCCATACTTTATTTATTTACTCTGCCATTATATGAAAGATAAAAAAATCTCATTTTCTGGAGATAAAAGCCTTTATATCATAACCGGAGGCGGCTGGAAAAGTTACGAAAAAGAATCTCTGAAACGTGATGATTTCAATCATCTTTTATTTGATACTTTCAATCTCAGTGATATTAGTCAGATCCGAGATATATTTAATCAAGTTGAACTCAACACTTGTTTCTTTGAGGATGAAATGCAGCGTAAACATGTTCCGCCGTGGGTATATGCGCGAGCGCTTGATCCTGAAACGTTGAAACCTGTACCTGATGGAACGCCGGGGTTGATGAGTTATATGGATGCGTCAGCAACCAGTTATCCAGCATTTATTGTTACCGATGATGTCGGGATAATTAGCAGAGAATATGGTAAGTATCCCGGCGTGCTCGTTGAAATTTTACGTCGCGTCAATACGAGGACGCAGAAAGGGTGTGCTTTAAGCTTAACCGAAGCGTTTGATAGTTGACTCGAGCACCACCACCACCACCACTGAGATCCGGCTGCTAACAAAGCCCGAAAGGAAGCTGAGTTGGCTGCTGCCACCGCTGAGCAATAACTAGCATAACCCCTTGGGGCCTCTAAACGGGTCTTGAGGGGTTTTTTGCTGAAAGGAGGAACTATATCCGGATTGGCGAATGGGACGCGCCCTGTAGCGGCGCATTAAGCGCGGCGGGTGTGGTGGTTACGCGCAGCGTGACCGCTACACTTGCCAGCGCCCTAGCGCCCGCTCCTTTCGCTTTCTTCCCTTCCTTTCTCGCCACGTTCGCCGGCTTTCCCCGTCAAGCTCTAAATCGGGGGCTCCCTTTAGGGTTCCGATTTAGTGCTTTACGGCACCTCGACCCCAAAAAACTTGATTAGGGTGATGGTTCACGTAGTGGGCCATCGCCCTGATAGACGGTTTTTCGCCCTTTGACGTTGGAGTCCACGTTCTTTAATAGTGGACTCTTGTTCCAAACTGGAACAACACTCAACCCTATCTCGGTCTATTCTTTTGATTTATAAGGGATTTTGCCGATTTCGGCCTATTGGTTAAAAAATGAGCTGATTTAACAAAAATTTAACGCGAATTTTAACAAAATATTAACGTTTACAATTTCAGGTGGCACTTTTCGGGGAAATGTGCGCGGAACCCCTATTTGTTTATTTTTCTAAATACATTCAAATATGTATCCGCTCATGAATTAATTCTTAGAAAAACTCATCGAGCATCAAATGAAACTGCAATTTATTCATATCAGGATTATCAATACCATATTTTTGAAAAAGCCGTTTCTGTAATGAAGGAGAAAACTCACCGAGGCAGTTCCATAGGATGGCAAGATCCTGGTATCGGTCTGCGATTCCGACTCGTCCAACATCAATACAACCTATTAATTTCCCCTCGTCAAAAATAAGGTTATCAAGTGAGAAATCACCATGAGTGACGACTGAATCCGGTGAGAATGGCAAAAGTTTATGCATTTCTTTCCAGACTTGTTCAACAGGCCAGCCATTACGCTCGTCATCAAAATCACTCGCATCAACCAAACCGTTATTCATTCGTGATTGCGCCTGAGCGAGACGAAATACGCGATCGCTGTTAAAAGGACAATTACAAACAGGAATCGAATGCAACCGGCGCAGGAACACTGCCAGCGCATCAACAATATTTTCACCTGAATCAGGATATTCTTCTAATACCTGGAATGCTGTTTTCCCGGGGATCGCAGTGGTGAGTAACCATGCATCATCAGGAGTACGGATAAAATGCTTGATGGTCGGAAGAGGCATAAATTCCGTCAGCCAGTTTAGTCTGACCATCTCATCTGTAACATCATTGGCAACGCTACCTTTGCCATGTTTCAGAAACAACTCTGGCGCATCGGGCTTCCCATACAATCGATAGATTGTCGCACCTGATTGCCCGACATTATCGCGAGCCCATTTATACCCATATAAATCAGCATCCATGTTGGAATTTAATCGCGGCCTAGAGCAAGACGTTTCCCGTTGAATATGGCTCATAACACCCCTTGTATTACTGTTTATGTAAGCAGACAGTTTTATTGTTCATGACCAAAATCCCTTAACGTGAGTTTTCGTTCCACTGAGCGTCAGACCCCGTAGAAAAGATCAAAGGATCTTCTTGAGATCCTTTTTTTCTGCGCGTAATCTGCTGCTTGCAAACAAAAAAACCACCGCTACCAGCGGTGGTTTGTTTGCCGGATCAAGAGCTACCAACTCTTTTTCCGAAGGTAACTGGCTTCAGCAGAGCGCAGATACCAAATACTGTCCTTCTAGTGTAGCCGTAGTTAGGCCACCACTTCAAGAACTCTGTAGCACCGCCTACATACCTCGCTCTGCTAATCCTGTTACCAGTGGCTGCTGCCAGTGGCGATAAGTCGTGTCTTACCGGGTTGGACTCAAGACGATAGTTACCGGATAAGGCGCAGCGGTCGGGCTGAACGGGGGGTTCGTGCACACAGCCCAGCTTGGAGCGAACGACCTACACCGAACTGAGATACCTACAGCGTGAGCTATGAGAAAGCGCCACGCTTCCCGAAGGGAGAAAGGCGGACAGGTATCCGGTAAGCGGCAGGGTCGGAACAGGAGAGCGCACGAGGGAGCTTCCAGGGGGAAACGCCTGGTATCTTTATAGTCCTGTCGGGTTTCGCCACCTCTGACTTGAGCGTCGATTTTTGTGATGCTCGTCAGGGGGGCGGAGCCTATGGAAAAACGCCAGCAACGCGGCCTTTTTACGGTTCCTGGCCTTTTGCTGGCCTTTTGCTCACATGTTCTTTCCTGCGTTATCCCCTGATTCTGTGGATAACCGTATTACCGCCTTTGAGTGAGCTGATACCGCTCGCCGCAGCCGAACGACCGAGCGCAGCGAGTCAGTGAGCGAGGAAGCGGAAGAGCGCCTGATGCGGTATTTTCTCCTTACGCATCTGTGCGGTATTTCACACCGCATATATGGTGCACTCTCAGTACAATCTGCTCTGATGCCGCATAGTTAAGCCAGTATACACTCCGCTATCGCTACGTGACTGGGTCATGGCTG


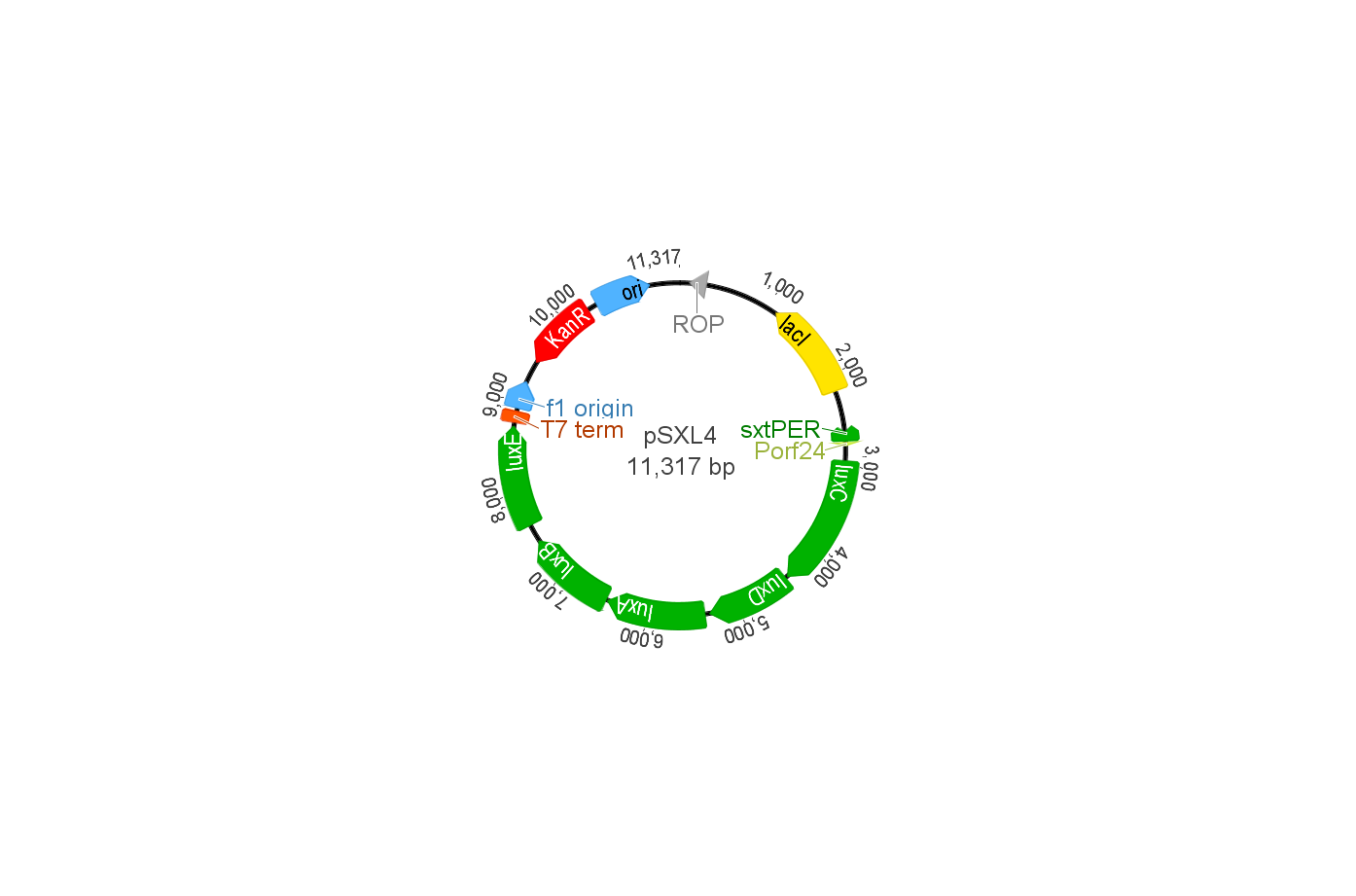


>pSXL4_nucl

CGCCCCGACACCCGCCAACACCCGCTGACGCGCCCTGACGGGCTTGTCTGCTCCCGGCATCCGCTTACAGACAAGCTGTGACCGTCTCCGGGAGCTGCATGTGTCAGAGGTTTTCACCGTCATCACCGAAACGCGCGAGGCAGCTGCGGTAAAGCTCATCAGCGTGGTCGTGAAGCGATTCACAGATGTCTGCCTGTTCATCCGCGTCCAGCTCGTTGAGTTTCTCCAGAAGCGTTAATGTCTGGCTTCTGATAAAGCGGGCCATGTTAAGGGCGGTTTTTTCCTGTTTGGTCACTGATGCCTCCGTGTAAGGGGGATTTCTGTTCATGGGGGTAATGATACCGATGAAACGAGAGAGGATGCTCACGATACGGGTTACTGATGATGAACATGCCCGGTTACTGGAACGTTGTGAGGGTAAACAACTGGCGGTATGGATGCGGCGGGACCAGAGAAAAATCACTCAGGGTCAATGCCAGCGCTTCGTTAATACAGATGTAGGTGTTCCACAGGGTAGCCAGCAGCATCCTGCGATGCAGATCCGGAACATAATGGTGCAGGGCGCTGACTTCCGCGTTTCCAGACTTTACGAAACACGGAAACCGAAGACCATTCATGTTGTTGCTCAGGTCGCAGACGTTTTGCAGCAGCAGTCGCTTCACGTTCGCTCGCGTATCGGTGATTCATTCTGCTAACCAGTAAGGCAACCCCGCCAGCCTAGCCGGGTCCTCAACGACAGGAGCACGATCATGCGCACCCGTGGGGCCGCCATGCCGGCGATAATGGCCTGCTTCTCGCCGAAACGTTTGGTGGCGGGACCAGTGACGAAGGCTTGAGCGAGGGCGTGCAAGATTCCGAATACCGCAAGCGACAGGCCGATCATCGTCGCGCTCCAGCGAAAGCGGTCCTCGCCGAAAATGACCCAGAGCGCTGCCGGCACCTGTCCTACGAGTTGCATGATAAAGAAGACAGTCATAAGTGCGGCGACGATAGTCATGCCCCGCGCCCACCGGAAGGAGCTGACTGGGTTGAAGGCTCTCAAGGGCATCGGTCGAGATCCCGGTGCCTAATGAGTGAGCTAACTTACATTAATTGCGTTGCGCTCACTGCCCGCTTTCCAGTCGGGAAACCTGTCGTGCCAGCTGCATTAATGAATCGGCCAACGCGCGGGGAGAGGCGGTTTGCGTATTGGGCGCCAGGGTGGTTTTTCTTTTCACCAGTGAGACGGGCAACAGCTGATTGCCCTTCACCGCCTGGCCCTGAGAGAGTTGCAGCAAGCGGTCCACGCTGGTTTGCCCCAGCAGGCGAAAATCCTGTTTGATGGTGGTTAACGGCGGGATATAACATGAGCTGTCTTCGGTATCGTCGTATCCCACTACCGAGATATCCGCACCAACGCGCAGCCCGGACTCGGTAATGGCGCGCATTGCGCCCAGCGCCATCTGATCGTTGGCAACCAGCATCGCAGTGGGAACGATGCCCTCATTCAGCATTTGCATGGTTTGTTGAAAACCGGACATGGCACTCCAGTCGCCTTCCCGTTCCGCTATCGGCTGAATTTGATTGCGAGTGAGATATTTATGCCAGCCAGCCAGACGCAGACGCGCCGAGACAGAACTTAATGGGCCCGCTAACAGCGCGATTTGCTGGTGACCCAATGCGACCAGATGCTCCACGCCCAGTCGCGTACCGTCTTCATGGGAGAAAATAATACTGTTGATGGGTGTCTGGTCAGAGACATCAAGAAATAACGCCGGAACATTAGTGCAGGCAGCTTCCACAGCAATGGCATCCTGGTCATCCAGCGGATAGTTAATGATCAGCCCACTGACGCGTTGCGCGAGAAGATTGTGCACCGCCGCTTTACAGGCTTCGACGCCGCTTCGTTCTACCATCGACACCACCACGCTGGCACCCAGTTGATCGGCGCGAGATTTAATCGCCGCGACAATTTGCGACGGCGCGTGCAGGGCCAGACTGGAGGTGGCAACGCCAATCAGCAACGACTGTTTGCCCGCCAGTTGTTGTGCCACGCGGTTGGGAATGTAATTCAGCTCCGCCATCGCCGCTTCCACTTTTTCCCGCGTTTTCGCAGAAACGTGGCTGGCCTGGTTCACCACGCGGGAAACGGTCTGATAAGAGACACCGGCATACTCTGCGACATCGTATAACGTTACTGGTTTCACATTCACCACCCTGAATTGACTCTCTTCCGGGCGCTATCATGCCATACCGCGAAAGGTTTTGCGCCATTCGATGGTGTCCGGGATCTCGACGCTCTCCCTTATGCGACTCCTGCATTAGGAAGCAGCCCAGTAGTAGGTTGAGGCCGTTGAGCACCGCCGCCGCAAGGAATGGTGCATGCAAGGAGATGGCGCCCAACAGTCCCCCGGCCACGGGGCCTGCCACCATACCCACGCCGAAACAAGCGCTCATGAGCCCGAAGTGGCGAGCCCGATCTTCCCCATCGGTGATGTCGGCGATATAGGCGCCAGCAACCGCACCTGTGGCGCCGGTGATGCCGGCCACGATGCGTCCGGCGTAGAGGATCGAGATCTCGATCCCGCGAAATGTTAGTGATTAAACCTTTAGTAATTGGCAAGATTGAGCTATAGACTAGAGTTGCCAGAATAATCAACAGCACACCCAAAATATATCGGTTATTTTCTGGGGCAGATGTTGTCATCTCAACCTTATCCAAGCTATTTTGGAGTTCTGTTAAATCTTGGTTTACCATCTCAACTTAAAATTTCCTTAGGTCTGGGCGTAGTGCTATAATGAAATCTTTGGCTGTACGGTATATCGCTAATAACGCGAATAATGTGAGTCATGTAATTGTAACTCATAAATCCCTTGAATTGCAAGGAAATAAAACAGATGCTTACATCTGAATAAAAACCAAGCAATGTTTTATACAGAAATTACATAGCACACCGCAGCTGCCAATGACTAAAAAAATTTCATTCATTATTAACGGCCAGGTTGAAATCTTTCCCGAAAGTGATGATTTAGTGCAATCCATTAATTTTGGTGATAATAGTGTTTACCTGCCAATATTGAATGACTCTCATGTAAAAAACATTATTGATTGTAATGGAAATAACGAATTACGGTTGCATAACATTGTCAATTTTCTCTATACGGTAGGGCAAAGATGGAAAAATGAAGAATACTCAAGACGCAGGACATACATTCGTGACTTAAAAAAATATATGGGATATTCAGAAGAAATGGCTAAGCTAGAGGCCAATTGGATATCTATGATTTTATGTTCTAAAGGCGGCCTTTATGATGTTGTAGAAAATGAACTTGGTTCTCGCCATATCATGGATGAATGGCTACCTCAGGATGAAAGTTATGTTCGGGCTTTTCCGAAAGGTAAATCTGTACATCTGTTGGCAGGTAATGTTCCATTATCTGGGATCATGTCTATATTACGCGCAATTTTAACTAAGAATCAGTGTATTATAAAAACATCGTCAACCGATCCTTTTACCGCTAATGCATTAGCGTTAAGTTTTATTGATGTAGACCCTAATCATCCGATAACGCGCTCTTTATCTGTTATATATTGGCCCCACCAAGGTGATACATCACTCGCAAAAGAAATTATGCGACATGCGGATGTTATTGTCGCTTGGGGAGGGCCAGATGCGATTAATTGGGCGGTAGAGCATGCGCCATCTTATGCTGATGTGATTAAATTTGGTTCTAAAAAGAGTCTTTGCATTATCGATAATCCTGTTGATTTGACGTCCGCAGCGACAGGTGCGGCTCATGATGTTTGTTTTTACGATCAGCGAGCTTGTTTTTCTGCCCAAAACATATATTACATGGGAAATCATTATGAGGAATTTAAGTTAGCGTTGATAGAAAAACTTAATCTATATGCGCATATATTACCGAATGCCAAAAAAGATTTTGATGAAAAGGCGGCCTATTCTTTAGTTCAAAAAGAAAGCTTGTTTGCTGGATTAAAAGTAGAGGTGGATATTCATCAACGTTGGATGATTATTGAGTCAAATGCAGGTGTGGAATTTAATCAACCACTTGGCAGATGTGTGTACCTTCATCACGTCGATAATATTGAGCAAATATTGCCTTATGTTCAAAAAAATAAGACGCAAACCATATCTATTTTTCCTTGGGAGTCATCATTTAAATATCGAGATGCGTTAGCATTAAAAGGTGCGGAAAGGATTGTAGAAGCAGGAATGAATAACATATTTCGAGTTGGTGGATCTCATGACGGAATGAGACCGTTGCAACGATTAGTGACATATATTTCTCATGAAAGGCCATCTAACTATACGGCTAAGGATGTTGCGGTTGAAATAGAACAGACTCGATTCCTGGAAGAAGATAAGTTCCTTGTATTTGTCCCATAATAGGTAAAAGTATGGAAAATGAATCAAAATATAAAACCATCGACCACGTTATTTGTGTTGAAGGAAATAAAAAAATTCATGTTTGGGAAACGCTGCCAGAAGAAAACAGCCCAAAGAGAAAGAATGCCATTATTATTGCGTCTGGTTTTGCCCGCAGGATGGATCATTTTGCTGGTCTGGCGGAATATTTATCGCGGAATGGATTTCATGTGATCCGCTATGATTCGCTTCACCACGTTGGATTGAGTTCAGGGACAATTGATGAATTTACAATGTCTATAGGAAAGCAGAGCTTGTTAGCAGTGGTTGATTGGTTAACTACACGAAAAATAAATAACTTCGGTATGTTGGCTTCAAGCTTATCTGCGCGGATAGCTTATGCAAGCCTATCTGAAATCAATGCTTCGTTTTTAATCACCGCAGTCGGTGTTGTTAACTTAAGATATTCTCTTGAAAGAGCTTTAGGGTTTGATTATCTCAGTCTACCCATTAATGAATTGCCGGATAATCTAGATTTTGAAGGCCATAAATTGGGTGCTGAAGTCTTTGCGAGAGATTGTCTTGATTTTGGTTGGGAAGATTTAGCTTCTACAATTAATAACATGATGTATCTTGATATACCGTTTATTGCTTTTACTGCAAATAACGATAATTGGGTCAAGCAAGATGAAGTTATCACATTGTTATCAAATATTCGTAGTAATCGATGCAAGATATATTCTTTGTTAGGAAGTTCGCATGACTTGAGTGAAAATTTAGTGGTCCTGCGCAATTTTTATCAATCGGTTACGAAAGCCGCTATCGCGATGGATAATGATCATCTGGATATTGATGTTGATATTACTGAACCGTCATTTGAACATTTAACTATTGCGACAGTCAATGAACGCCGAATGAGAATTGAGATTGAAAATCAAGCAATTTCTCTGTCTTAAAATCTATTGAGATATTCTATCACTCAAATAGCAATATAAGGACTCTCTATGAAATTTGGAAACTTTTTGCTTACATACCAACCTCCCCAATTTTCTCAAACAGAGGTAATGAAACGTTTGGTTAAATTAGGTCGCATCTCTGAGGAGTGTGGTTTTGATACCGTATGGTTACTGGAGCATCATTTCACGGAGTTTGGTTTGCTTGGTAACCCTTATGTCGCTGCTGCATATTTACTTGGCGCGACTAAAAAATTGAATGTAGGAACTGCCGCTATTGTTCTTCCCACAGCCCATCCAGTACGCCAACTTGAAGATGTGAATTTATTGGATCAAATGTCAAAAGGACGATTTCGGTTTGGTATTTGCCGAGGGCTTTACAACAAGGACTTTCGCGTATTCGGCACAGATATGAATAACAGTCGCGCCTTAGCGGAATGCTGGTACGGGCTGATAAAGAATGGCATGACAGAGGGATATATGGAAGCTGATAATGAACATATCAAGTTCCATAAGGTAAAAGTAAACCCCGCGGCGTATAGCAGAGGTGGCGCACCGGTTTATGTGGTGGCTGAATCAGCTTCGACGACTGAGTGGGCTGCTCAATTTGGCCTACCGATGATATTAAGTTGGATTATAAATACTAACGAAAAGAAAGCACAACTTGAGCTTTATAATGAAGTGGCTCAAGAATATGGGCACGATATTCATAATATCGACCATTGCTTATCATATATAACATCTGTAGATCATGACTCAATTAAAGCGAAAGAGATTTGCCGGAAATTTCTGGGGCATTGGTATGATTCTTATGTGAATGCTACGACTATTTTTGATGATTCAGACCAAACAAGAGGTTATGATTTCAATAAAGGGCAGTGGCGTGACTTTGTATTAAAAGGACATAAAGATACTAATCGCCGTATTGATTACAGTTACGAAATCAATCCCGTGGGAACGCCGCAGGAATGTATTGACATAATTCAAAAAGACATTGATGCTACAGGAATATCAAATATTTGTTGTGGATTTGAAGCTAATGGAACAGTAGACGAAATTATTGCTTCCATGAAGCTCTTCCAGTCTGATGTCATGCCATTTCTTAAAGAAAAACAACGTTCGCTATTATATTAGCTAAGGAGAAAGAAATGAAATTTGGATTGTTCTTCCTTAACTTCATCAATTCAACAACTGTTCAAGAACAAAGTATAGTTCGCATGCAGGAAATAACGGAGTATGTTGATAAGTTGAATTTTGAACAGATTTTAGTGTATGAAAATCATTTTTCAGATAATGGTGTTGTCGGCGCTCCTCTGACTGTTTCTGGTTTTCTGCTCGGTTTAACAGAGAAAATTAAAATTGGTTCATTAAATCACATCATTACAACTCATCATCCTGTCGCCATAGCGGAGGAAGCTTGCTTATTGGATCAGTTAAGTGAAGGGAGATTTATTTTAGGGTTTAGTGATTGCGAAAAAAAAGATGAAATGCATTTTTTTAATCGCCCGGTTGAATATCAACAGCAACTATTTGAAGAGTGTTATGAAATCATTAACGATGCTTTAACAACAGGCTATTGTAATCCAGATAACGATTTTTATAGCTTCCCTAAAATATCTGTAAATCCCCATGCTTATACGCCAGGCGGACCTCGGAAATATGTAACAGCAACCAGTCATCATATTGTTGAGTGGGCGGCCAAAAAAGGTATTCCTCTCATCTTTAAGTGGGATGATTCTAATGATGTTAGATATGAATATGCTGAAAGATATAAAGCCGTTGCGGATAAATATGACGTTGACCTATCAGAGATAGACCATCAGTTAATGATATTAGTTAACTATAACGAAGATAGTAATAAAGCTAAACAAGAGACGCGTGCATTTATTAGTGATTATGTTCTTGAAATGCACCCTAATGAAAATTTCGAAAATAAACTTGAAGAAATAATTGCAGAAAACGCTGTCGGAAATTATACGGAGTGTATAACTGCGGCTAAGTTGGCAATTGAAAAGTGTGGTGCGAAAAGTGTATTGCTGTCCTTTGAACCAATGAATGATTTGATGAGCCAAAAAAATGTAATCAATATTGTTGATGATAATATTAAGAAGTACCACATGGAATATACCTAATAGATTTCGAGTTGCAGCGAGGCGGCAAGTGAACGAATCCCCAGGAGCATAGATAACTATGTGACTGGGGTGAGTGAAAGCAGCCAACAAAGCAGCAGCTTGAAAGATGAAGGGTATAAAAGAGTATGACAGCAGTGCTGCCATACTTTCTAATATTATCTTGAGGAGTAAAACAGGTATGACTTCATATGTTGATAAACAAGAAATTACAGCAAGCTCAGAAATTGATGATTTGATTTTTTCGAGCGATCCATTAGTGTGGTCTTACGACGAGCAGGAAAAAATCAGAAAGAAACTTGTGCTTGATGCATTTCGTAATCATTATAAACATTGTCGAGAATATCGTCACTACTGTCAGGCACACAAAGTAGATGACAATATTACGGAAATTGATGACATACCTGTATTCCCAACATCGGTTTTTAAGTTTACTCGCTTATTAACTTCTCAGGAAAACGAGATTGAAAGTTGGTTTACCAGTAGCGGCACGAATGGTTTAAAAAGTCAGGTGGCGCGTGACAGATTAAGTATTGAGAGACTCTTAGGCTCTGTGAGTTATGGCATGAAATATGTTGGTAGTTGGTTTGATCATCAAATAGAATTAGTCAATTTGGGACCAGATAGATTTAATGCTCATAATATTTGGTTTAAATATGTTATGAGTTTGGTGGAATTGTTATATCCTACGACATTTACCGTAACAGAAGAACGAATAGATTTTGTTAAAACATTGAATAGTCTTGAACGAATAAAAAATCAAGGGAAAGATCTTTGTCTTATTGGTTCGCCATACTTTATTTATTTACTCTGCCATTATATGAAAGATAAAAAAATCTCATTTTCTGGAGATAAAAGCCTTTATATCATAACCGGAGGCGGCTGGAAAAGTTACGAAAAAGAATCTCTGAAACGTGATGATTTCAATCATCTTTTATTTGATACTTTCAATCTCAGTGATATTAGTCAGATCCGAGATATATTTAATCAAGTTGAACTCAACACTTGTTTCTTTGAGGATGAAATGCAGCGTAAACATGTTCCGCCGTGGGTATATGCGCGAGCGCTTGATCCTGAAACGTTGAAACCTGTACCTGATGGAACGCCGGGGTTGATGAGTTATATGGATGCGTCAGCAACCAGTTATCCAGCATTTATTGTTACCGATGATGTCGGGATAATTAGCAGAGAATATGGTAAGTATCCCGGCGTGCTCGTTGAAATTTTACGTCGCGTCAATACGAGGACGCAGAAAGGGTGTGCTTTAAGCTTAACCGAAGCGTTTGATAGTTGACTCGAGCACCACCACCACCACCACTGAGATCCGGCTGCTAACAAAGCCCGAAAGGAAGCTGAGTTGGCTGCTGCCACCGCTGAGCAATAACTAGCATAACCCCTTGGGGCCTCTAAACGGGTCTTGAGGGGTTTTTTGCTGAAAGGAGGAACTATATCCGGATTGGCGAATGGGACGCGCCCTGTAGCGGCGCATTAAGCGCGGCGGGTGTGGTGGTTACGCGCAGCGTGACCGCTACACTTGCCAGCGCCCTAGCGCCCGCTCCTTTCGCTTTCTTCCCTTCCTTTCTCGCCACGTTCGCCGGCTTTCCCCGTCAAGCTCTAAATCGGGGGCTCCCTTTAGGGTTCCGATTTAGTGCTTTACGGCACCTCGACCCCAAAAAACTTGATTAGGGTGATGGTTCACGTAGTGGGCCATCGCCCTGATAGACGGTTTTTCGCCCTTTGACGTTGGAGTCCACGTTCTTTAATAGTGGACTCTTGTTCCAAACTGGAACAACACTCAACCCTATCTCGGTCTATTCTTTTGATTTATAAGGGATTTTGCCGATTTCGGCCTATTGGTTAAAAAATGAGCTGATTTAACAAAAATTTAACGCGAATTTTAACAAAATATTAACGTTTACAATTTCAGGTGGCACTTTTCGGGGAAATGTGCGCGGAACCCCTATTTGTTTATTTTTCTAAATACATTCAAATATGTATCCGCTCATGAATTAATTCTTAGAAAAACTCATCGAGCATCAAATGAAACTGCAATTTATTCATATCAGGATTATCAATACCATATTTTTGAAAAAGCCGTTTCTGTAATGAAGGAGAAAACTCACCGAGGCAGTTCCATAGGATGGCAAGATCCTGGTATCGGTCTGCGATTCCGACTCGTCCAACATCAATACAACCTATTAATTTCCCCTCGTCAAAAATAAGGTTATCAAGTGAGAAATCACCATGAGTGACGACTGAATCCGGTGAGAATGGCAAAAGTTTATGCATTTCTTTCCAGACTTGTTCAACAGGCCAGCCATTACGCTCGTCATCAAAATCACTCGCATCAACCAAACCGTTATTCATTCGTGATTGCGCCTGAGCGAGACGAAATACGCGATCGCTGTTAAAAGGACAATTACAAACAGGAATCGAATGCAACCGGCGCAGGAACACTGCCAGCGCATCAACAATATTTTCACCTGAATCAGGATATTCTTCTAATACCTGGAATGCTGTTTTCCCGGGGATCGCAGTGGTGAGTAACCATGCATCATCAGGAGTACGGATAAAATGCTTGATGGTCGGAAGAGGCATAAATTCCGTCAGCCAGTTTAGTCTGACCATCTCATCTGTAACATCATTGGCAACGCTACCTTTGCCATGTTTCAGAAACAACTCTGGCGCATCGGGCTTCCCATACAATCGATAGATTGTCGCACCTGATTGCCCGACATTATCGCGAGCCCATTTATACCCATATAAATCAGCATCCATGTTGGAATTTAATCGCGGCCTAGAGCAAGACGTTTCCCGTTGAATATGGCTCATAACACCCCTTGTATTACTGTTTATGTAAGCAGACAGTTTTATTGTTCATGACCAAAATCCCTTAACGTGAGTTTTCGTTCCACTGAGCGTCAGACCCCGTAGAAAAGATCAAAGGATCTTCTTGAGATCCTTTTTTTCTGCGCGTAATCTGCTGCTTGCAAACAAAAAAACCACCGCTACCAGCGGTGGTTTGTTTGCCGGATCAAGAGCTACCAACTCTTTTTCCGAAGGTAACTGGCTTCAGCAGAGCGCAGATACCAAATACTGTCCTTCTAGTGTAGCCGTAGTTAGGCCACCACTTCAAGAACTCTGTAGCACCGCCTACATACCTCGCTCTGCTAATCCTGTTACCAGTGGCTGCTGCCAGTGGCGATAAGTCGTGTCTTACCGGGTTGGACTCAAGACGATAGTTACCGGATAAGGCGCAGCGGTCGGGCTGAACGGGGGGTTCGTGCACACAGCCCAGCTTGGAGCGAACGACCTACACCGAACTGAGATACCTACAGCGTGAGCTATGAGAAAGCGCCACGCTTCCCGAAGGGAGAAAGGCGGACAGGTATCCGGTAAGCGGCAGGGTCGGAACAGGAGAGCGCACGAGGGAGCTTCCAGGGGGAAACGCCTGGTATCTTTATAGTCCTGTCGGGTTTCGCCACCTCTGACTTGAGCGTCGATTTTTGTGATGCTCGTCAGGGGGGCGGAGCCTATGGAAAAACGCCAGCAACGCGGCCTTTTTACGGTTCCTGGCCTTTTGCTGGCCTTTTGCTCACATGTTCTTTCCTGCGTTATCCCCTGATTCTGTGGATAACCGTATTACCGCCTTTGAGTGAGCTGATACCGCTCGCCGCAGCCGAACGACCGAGCGCAGCGAGTCAGTGAGCGAGGAAGCGGAAGAGCGCCTGATGCGGTATTTTCTCCTTACGCATCTGTGCGGTATTTCACACCGCATATATGGTGCACTCTCAGTACAATCTGCTCTGATGCCGCATAGTTAAGCCAGTATACACTCCGCTATCGCTACGTGACTGGGTCATGGCTG


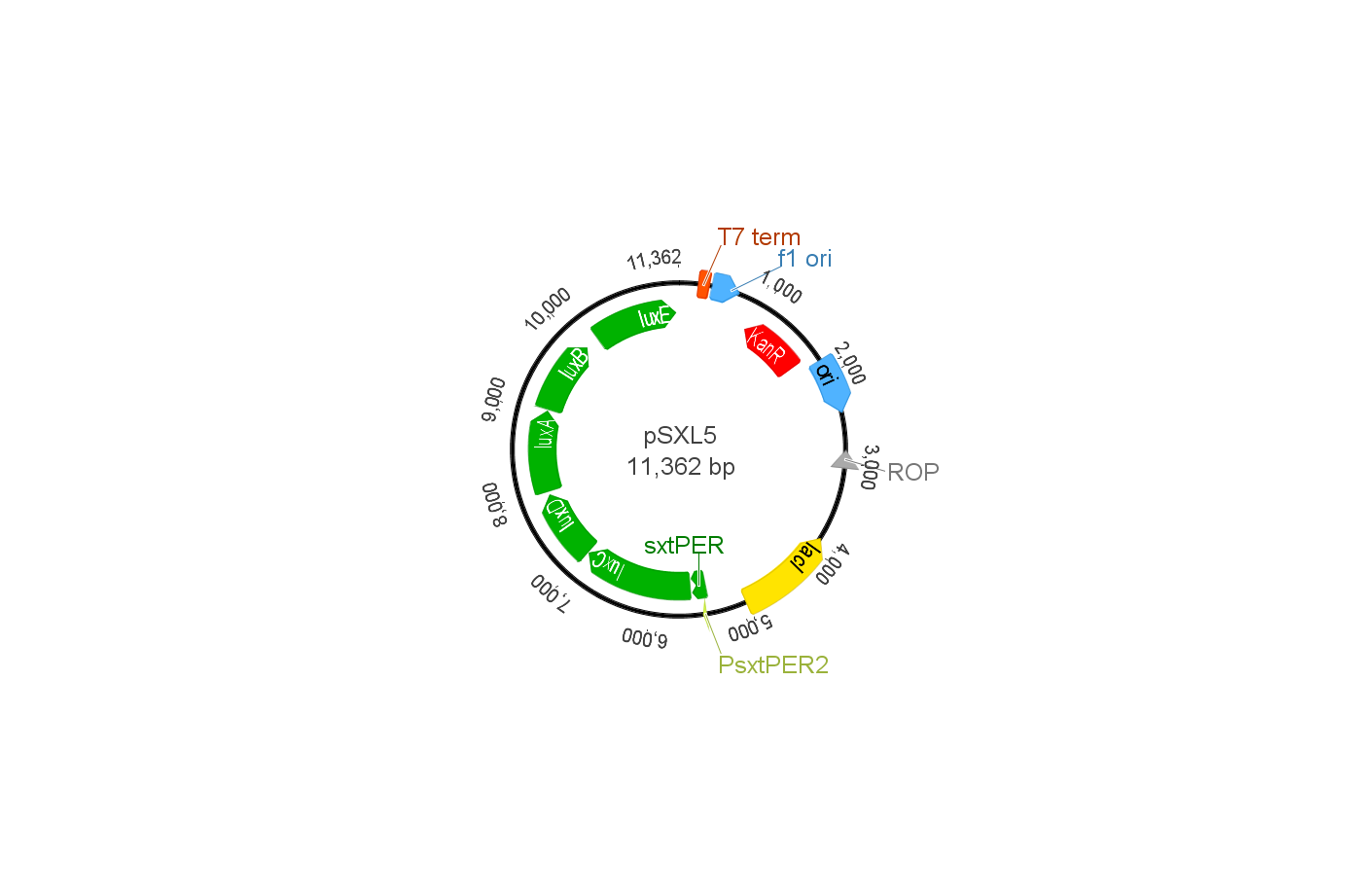


>pSXL5_nucl

TCTAGAAATAATTTTGTTTAACTTTAAGAAGGAGATATACCATGGGCAGCAGCCATCATCATCATCATCACAGCAGCGGCCTGGTGCCGCGCGGCAGCCATATGGCTAGCATGACTGGTGGACAGCAAATGGGTCGCGGATCCGAATTCGAGCTCCGTCGACAAGCTTGCGGCCGCACTCGAGCACCACCACCACCACCACTGAGATCCGGCTGCTAACAAAGCCCGAAAGGAAGCTGAGTTGGCTGCTGCCACCGCTGAGCAATAACTAGCATAACCCCTTGGGGCCTCTAAACGGGTCTTGAGGGGTTTTTTGCTGAAAGGAGGAACTATATCCGGATTGGCGAATGGGACGCGCCCTGTAGCGGCGCATTAAGCGCGGCGGGTGTGGTGGTTACGCGCAGCGTGACCGCTACACTTGCCAGCGCCCTAGCGCCCGCTCCTTTCGCTTTCTTCCCTTCCTTTCTCGCCACGTTCGCCGGCTTTCCCCGTCAAGCTCTAAATCGGGGGCTCCCTTTAGGGTTCCGATTTAGTGCTTTACGGCACCTCGACCCCAAAAAACTTGATTAGGGTGATGGTTCACGTAGTGGGCCATCGCCCTGATAGACGGTTTTTCGCCCTTTGACGTTGGAGTCCACGTTCTTTAATAGTGGACTCTTGTTCCAAACTGGAACAACACTCAACCCTATCTCGGTCTATTCTTTTGATTTATAAGGGATTTTGCCGATTTCGGCCTATTGGTTAAAAAATGAGCTGATTTAACAAAAATTTAACGCGAATTTTAACAAAATATTAACGTTTACAATTTCAGGTGGCACTTTTCGGGGAAATGTGCGCGGAACCCCTATTTGTTTATTTTTCTAAATACATTCAAATATGTATCCGCTCATGAATTAATTCTTAGAAAAACTCATCGAGCATCAAATGAAACTGCAATTTATTCATATCAGGATTATCAATACCATATTTTTGAAAAAGCCGTTTCTGTAATGAAGGAGAAAACTCACCGAGGCAGTTCCATAGGATGGCAAGATCCTGGTATCGGTCTGCGATTCCGACTCGTCCAACATCAATACAACCTATTAATTTCCCCTCGTCAAAAATAAGGTTATCAAGTGAGAAATCACCATGAGTGACGACTGAATCCGGTGAGAATGGCAAAAGTTTATGCATTTCTTTCCAGACTTGTTCAACAGGCCAGCCATTACGCTCGTCATCAAAATCACTCGCATCAACCAAACCGTTATTCATTCGTGATTGCGCCTGAGCGAGACGAAATACGCGATCGCTGTTAAAAGGACAATTACAAACAGGAATCGAATGCAACCGGCGCAGGAACACTGCCAGCGCATCAACAATATTTTCACCTGAATCAGGATATTCTTCTAATACCTGGAATGCTGTTTTCCCGGGGATCGCAGTGGTGAGTAACCATGCATCATCAGGAGTACGGATAAAATGCTTGATGGTCGGAAGAGGCATAAATTCCGTCAGCCAGTTTAGTCTGACCATCTCATCTGTAACATCATTGGCAACGCTACCTTTGCCATGTTTCAGAAACAACTCTGGCGCATCGGGCTTCCCATACAATCGATAGATTGTCGCACCTGATTGCCCGACATTATCGCGAGCCCATTTATACCCATATAAATCAGCATCCATGTTGGAATTTAATCGCGGCCTAGAGCAAGACGTTTCCCGTTGAATATGGCTCATAACACCCCTTGTATTACTGTTTATGTAAGCAGACAGTTTTATTGTTCATGACCAAAATCCCTTAACGTGAGTTTTCGTTCCACTGAGCGTCAGACCCCGTAGAAAAGATCAAAGGATCTTCTTGAGATCCTTTTTTTCTGCGCGTAATCTGCTGCTTGCAAACAAAAAAACCACCGCTACCAGCGGTGGTTTGTTTGCCGGATCAAGAGCTACCAACTCTTTTTCCGAAGGTAACTGGCTTCAGCAGAGCGCAGATACCAAATACTGTCCTTCTAGTGTAGCCGTAGTTAGGCCACCACTTCAAGAACTCTGTAGCACCGCCTACATACCTCGCTCTGCTAATCCTGTTACCAGTGGCTGCTGCCAGTGGCGATAAGTCGTGTCTTACCGGGTTGGACTCAAGACGATAGTTACCGGATAAGGCGCAGCGGTCGGGCTGAACGGGGGGTTCGTGCACACAGCCCAGCTTGGAGCGAACGACCTACACCGAACTGAGATACCTACAGCGTGAGCTATGAGAAAGCGCCACGCTTCCCGAAGGGAGAAAGGCGGACAGGTATCCGGTAAGCGGCAGGGTCGGAACAGGAGAGCGCACGAGGGAGCTTCCAGGGGGAAACGCCTGGTATCTTTATAGTCCTGTCGGGTTTCGCCACCTCTGACTTGAGCGTCGATTTTTGTGATGCTCGTCAGGGGGGCGGAGCCTATGGAAAAACGCCAGCAACGCGGCCTTTTTACGGTTCCTGGCCTTTTGCTGGCCTTTTGCTCACATGTTCTTTCCTGCGTTATCCCCTGATTCTGTGGATAACCGTATTACCGCCTTTGAGTGAGCTGATACCGCTCGCCGCAGCCGAACGACCGAGCGCAGCGAGTCAGTGAGCGAGGAAGCGGAAGAGCGCCTGATGCGGTATTTTCTCCTTACGCATCTGTGCGGTATTTCACACCGCATATATGGTGCACTCTCAGTACAATCTGCTCTGATGCCGCATAGTTAAGCCAGTATACACTCCGCTATCGCTACGTGACTGGGTCATGGCTGCGCCCCGACACCCGCCAACACCCGCTGACGCGCCCTGACGGGCTTGTCTGCTCCCGGCATCCGCTTACAGACAAGCTGTGACCGTCTCCGGGAGCTGCATGTGTCAGAGGTTTTCACCGTCATCACCGAAACGCGCGAGGCAGCTGCGGTAAAGCTCATCAGCGTGGTCGTGAAGCGATTCACAGATGTCTGCCTGTTCATCCGCGTCCAGCTCGTTGAGTTTCTCCAGAAGCGTTAATGTCTGGCTTCTGATAAAGCGGGCCATGTTAAGGGCGGTTTTTTCCTGTTTGGTCACTGATGCCTCCGTGTAAGGGGGATTTCTGTTCATGGGGGTAATGATACCGATGAAACGAGAGAGGATGCTCACGATACGGGTTACTGATGATGAACATGCCCGGTTACTGGAACGTTGTGAGGGTAAACAACTGGCGGTATGGATGCGGCGGGACCAGAGAAAAATCACTCAGGGTCAATGCCAGCGCTTCGTTAATACAGATGTAGGTGTTCCACAGGGTAGCCAGCAGCATCCTGCGATGCAGATCCGGAACATAATGGTGCAGGGCGCTGACTTCCGCGTTTCCAGACTTTACGAAACACGGAAACCGAAGACCATTCATGTTGTTGCTCAGGTCGCAGACGTTTTGCAGCAGCAGTCGCTTCACGTTCGCTCGCGTATCGGTGATTCATTCTGCTAACCAGTAAGGCAACCCCGCCAGCCTAGCCGGGTCCTCAACGACAGGAGCACGATCATGCGCACCCGTGGGGCCGCCATGCCGGCGATAATGGCCTGCTTCTCGCCGAAACGTTTGGTGGCGGGACCAGTGACGAAGGCTTGAGCGAGGGCGTGCAAGATTCCGAATACCGCAAGCGACAGGCCGATCATCGTCGCGCTCCAGCGAAAGCGGTCCTCGCCGAAAATGACCCAGAGCGCTGCCGGCACCTGTCCTACGAGTTGCATGATAAAGAAGACAGTCATAAGTGCGGCGACGATAGTCATGCCCCGCGCCCACCGGAAGGAGCTGACTGGGTTGAAGGCTCTCAAGGGCATCGGTCGAGATCCCGGTGCCTAATGAGTGAGCTAACTTACATTAATTGCGTTGCGCTCACTGCCCGCTTTCCAGTCGGGAAACCTGTCGTGCCAGCTGCATTAATGAATCGGCCAACGCGCGGGGAGAGGCGGTTTGCGTATTGGGCGCCAGGGTGGTTTTTCTTTTCACCAGTGAGACGGGCAACAGCTGATTGCCCTTCACCGCCTGGCCCTGAGAGAGTTGCAGCAAGCGGTCCACGCTGGTTTGCCCCAGCAGGCGAAAATCCTGTTTGATGGTGGTTAACGGCGGGATATAACATGAGCTGTCTTCGGTATCGTCGTATCCCACTACCGAGATATCCGCACCAACGCGCAGCCCGGACTCGGTAATGGCGCGCATTGCGCCCAGCGCCATCTGATCGTTGGCAACCAGCATCGCAGTGGGAACGATGCCCTCATTCAGCATTTGCATGGTTTGTTGAAAACCGGACATGGCACTCCAGTCGCCTTCCCGTTCCGCTATCGGCTGAATTTGATTGCGAGTGAGATATTTATGCCAGCCAGCCAGACGCAGACGCGCCGAGACAGAACTTAATGGGCCCGCTAACAGCGCGATTTGCTGGTGACCCAATGCGACCAGATGCTCCACGCCCAGTCGCGTACCGTCTTCATGGGAGAAAATAATACTGTTGATGGGTGTCTGGTCAGAGACATCAAGAAATAACGCCGGAACATTAGTGCAGGCAGCTTCCACAGCAATGGCATCCTGGTCATCCAGCGGATAGTTAATGATCAGCCCACTGACGCGTTGCGCGAGAAGATTGTGCACCGCCGCTTTACAGGCTTCGACGCCGCTTCGTTCTACCATCGACACCACCACGCTGGCACCCAGTTGATCGGCGCGAGATTTAATCGCCGCGACAATTTGCGACGGCGCGTGCAGGGCCAGACTGGAGGTGGCAACGCCAATCAGCAACGACTGTTTGCCCGCCAGTTGTTGTGCCACGCGGTTGGGAATGTAATTCAGCTCCGCCATCGCCGCTTCCACTTTTTCCCGCGTTTTCGCAGAAACGTGGCTGGCCTGGTTCACCACGCGGGAAACGGTCTGATAAGAGACACCGGCATACTCTGCGACATCGTATAACGTTACTGGTTTCACATTCACCACCCTGAATTGACTCTCTTCCGGGCGCTATCATGCCATACCGCGAAAGGTTTTGCGCCATTCGATGGTGTCCGGGATCTCGACGCTCTCCCTTATGCGACTCCTGCATTAGGAAGCAGCCCAGTAGTAGGTTGAGGCCGTTGAGCACCGCCGCCGCAAGGAATGGTGCATGCAAGGAGATGGCGCCCAACAGTCCCCCGGCCACGGGGCCTGCCACCATACCCACGCCGAAACAAGCGCTCATGAGCCCGAAGTGGCGAGCCCGATCTTCCCCATCGGTGATGTCGGCGATATAGGCGCCAGCAACCGCACCTGTGGCGCCGGTGATGCCGGCCACGATGCGTCCGGCGTAGAGGATCGAGATCTCGATCCCGCGAAATTTTAAGTTGAGATGGTAAACCAAGATTTAACAGAACTCCAAAATAGCTTGGATAAGGTTGAGATGACAACATCTGCCCCAGAAAATAACCGATATATTTTGGGTGTGCTGTTGATTATTCTGGCAACTCTAGTCTATAGCTCAATCTTGCCAATTACTAAAGGTTTAATCACTAACATCTCTAAAGAAGTGCTAATGACTAAAAAAAGAAGTGCTAATGACTAAAAAAATTTCATTCATTATTAACGGCCAGGTTGAAATCTTTCCCGAAAGTGATGATTTAGTGCAATCCATTAATTTTGGTGATAATAGTGTTTACCTGCCAATATTGAATGACTCTCATGTAAAAAACATTATTGATTGTAATGGAAATAACGAATTACGGTTGCATAACATTGTCAATTTTCTCTATACGGTAGGGCAAAGATGGAAAAATGAAGAATACTCAAGACGCAGGACATACATTCGTGACTTAAAAAAATATATGGGATATTCAGAAGAAATGGCTAAGCTAGAGGCCAATTGGATATCTATGATTTTATGTTCTAAAGGCGGCCTTTATGATGTTGTAGAAAATGAACTTGGTTCTCGCCATATCATGGATGAATGGCTACCTCAGGATGAAAGTTATGTTCGGGCTTTTCCGAAAGGTAAATCTGTACATCTGTTGGCAGGTAATGTTCCATTATCTGGGATCATGTCTATATTACGCGCAATTTTAACTAAGAATCAGTGTATTATAAAAACATCGTCAACCGATCCTTTTACCGCTAATGCATTAGCGTTAAGTTTTATTGATGTAGACCCTAATCATCCGATAACGCGCTCTTTATCTGTTATATATTGGCCCCACCAAGGTGATACATCACTCGCAAAAGAAATTATGCGACATGCGGATGTTATTGTCGCTTGGGGAGGGCCAGATGCGATTAATTGGGCGGTAGAGCATGCGCCATCTTATGCTGATGTGATTAAATTTGGTTCTAAAAAGAGTCTTTGCATTATCGATAATCCTGTTGATTTGACGTCCGCAGCGACAGGTGCGGCTCATGATGTTTGTTTTTACGATCAGCGAGCTTGTTTTTCTGCCCAAAACATATATTACATGGGAAATCATTATGAGGAATTTAAGTTAGCGTTGATAGAAAAACTTAATCTATATGCGCATATATTACCGAATGCCAAAAAAGATTTTGATGAAAAGGCGGCCTATTCTTTAGTTCAAAAAGAAAGCTTGTTTGCTGGATTAAAAGTAGAGGTGGATATTCATCAACGTTGGATGATTATTGAGTCAAATGCAGGTGTGGAATTTAATCAACCACTTGGCAGATGTGTGTACCTTCATCACGTCGATAATATTGAGCAAATATTGCCTTATGTTCAAAAAAATAAGACGCAAACCATATCTATTTTTCCTTGGGAGTCATCATTTAAATATCGAGATGCGTTAGCATTAAAAGGTGCGGAAAGGATTGTAGAAGCAGGAATGAATAACATATTTCGAGTTGGTGGATCTCATGACGGAATGAGACCGTTGCAACGATTAGTGACATATATTTCTCATGAAAGGCCATCTAACTATACGGCTAAGGATGTTGCGGTTGAAATAGAACAGACTCGATTCCTGGAAGAAGATAAGTTCCTTGTATTTGTCCCATAATAGGTAAAAGTATGGAAAATGAATCAAAATATAAAACCATCGACCACGTTATTTGTGTTGAAGGAAATAAAAAAATTCATGTTTGGGAAACGCTGCCAGAAGAAAACAGCCCAAAGAGAAAGAATGCCATTATTATTGCGTCTGGTTTTGCCCGCAGGATGGATCATTTTGCTGGTCTGGCGGAATATTTATCGCGGAATGGATTTCATGTGATCCGCTATGATTCGCTTCACCACGTTGGATTGAGTTCAGGGACAATTGATGAATTTACAATGTCTATAGGAAAGCAGAGCTTGTTAGCAGTGGTTGATTGGTTAACTACACGAAAAATAAATAACTTCGGTATGTTGGCTTCAAGCTTATCTGCGCGGATAGCTTATGCAAGCCTATCTGAAATCAATGCTTCGTTTTTAATCACCGCAGTCGGTGTTGTTAACTTAAGATATTCTCTTGAAAGAGCTTTAGGGTTTGATTATCTCAGTCTACCCATTAATGAATTGCCGGATAATCTAGATTTTGAAGGCCATAAATTGGGTGCTGAAGTCTTTGCGAGAGATTGTCTTGATTTTGGTTGGGAAGATTTAGCTTCTACAATTAATAACATGATGTATCTTGATATACCGTTTATTGCTTTTACTGCAAATAACGATAATTGGGTCAAGCAAGATGAAGTTATCACATTGTTATCAAATATTCGTAGTAATCGATGCAAGATATATTCTTTGTTAGGAAGTTCGCATGACTTGAGTGAAAATTTAGTGGTCCTGCGCAATTTTTATCAATCGGTTACGAAAGCCGCTATCGCGATGGATAATGATCATCTGGATATTGATGTTGATATTACTGAACCGTCATTTGAACATTTAACTATTGCGACAGTCAATGAACGCCGAATGAGAATTGAGATTGAAAATCAAGCAATTTCTCTGTCTTAAAATCTATTGAGATATTCTATCACTCAAATAGCAATATAAGGACTCTCTATGAAATTTGGAAACTTTTTGCTTACATACCAACCTCCCCAATTTTCTCAAACAGAGGTAATGAAACGTTTGGTTAAATTAGGTCGCATCTCTGAGGAGTGTGGTTTTGATACCGTATGGTTACTGGAGCATCATTTCACGGAGTTTGGTTTGCTTGGTAACCCTTATGTCGCTGCTGCATATTTACTTGGCGCGACTAAAAAATTGAATGTAGGAACTGCCGCTATTGTTCTTCCCACAGCCCATCCAGTACGCCAACTTGAAGATGTGAATTTATTGGATCAAATGTCAAAAGGACGATTTCGGTTTGGTATTTGCCGAGGGCTTTACAACAAGGACTTTCGCGTATTCGGCACAGATATGAATAACAGTCGCGCCTTAGCGGAATGCTGGTACGGGCTGATAAAGAATGGCATGACAGAGGGATATATGGAAGCTGATAATGAACATATCAAGTTCCATAAGGTAAAAGTAAACCCCGCGGCGTATAGCAGAGGTGGCGCACCGGTTTATGTGGTGGCTGAATCAGCTTCGACGACTGAGTGGGCTGCTCAATTTGGCCTACCGATGATATTAAGTTGGATTATAAATACTAACGAAAAGAAAGCACAACTTGAGCTTTATAATGAAGTGGCTCAAGAATATGGGCACGATATTCATAATATCGACCATTGCTTATCATATATAACATCTGTAGATCATGACTCAATTAAAGCGAAAGAGATTTGCCGGAAATTTCTGGGGCATTGGTATGATTCTTATGTGAATGCTACGACTATTTTTGATGATTCAGACCAAACAAGAGGTTATGATTTCAATAAAGGGCAGTGGCGTGACTTTGTATTAAAAGGACATAAAGATACTAATCGCCGTATTGATTACAGTTACGAAATCAATCCCGTGGGAACGCCGCAGGAATGTATTGACATAATTCAAAAAGACATTGATGCTACAGGAATATCAAATATTTGTTGTGGATTTGAAGCTAATGGAACAGTAGACGAAATTATTGCTTCCATGAAGCTCTTCCAGTCTGATGTCATGCCATTTCTTAAAGAAAAACAACGTTCGCTATTATATTAGCTAAGGAGAAAGAAATGAAATTTGGATTGTTCTTCCTTAACTTCATCAATTCAACAACTGTTCAAGAACAAAGTATAGTTCGCATGCAGGAAATAACGGAGTATGTTGATAAGTTGAATTTTGAACAGATTTTAGTGTATGAAAATCATTTTTCAGATAATGGTGTTGTCGGCGCTCCTCTGACTGTTTCTGGTTTTCTGCTCGGTTTAACAGAGAAAATTAAAATTGGTTCATTAAATCACATCATTACAACTCATCATCCTGTCGCCATAGCGGAGGAAGCTTGCTTATTGGATCAGTTAAGTGAAGGGAGATTTATTTTAGGGTTTAGTGATTGCGAAAAAAAAGATGAAATGCATTTTTTTAATCGCCCGGTTGAATATCAACAGCAACTATTTGAAGAGTGTTATGAAATCATTAACGATGCTTTAACAACAGGCTATTGTAATCCAGATAACGATTTTTATAGCTTCCCTAAAATATCTGTAAATCCCCATGCTTATACGCCAGGCGGACCTCGGAAATATGTAACAGCAACCAGTCATCATATTGTTGAGTGGGCGGCCAAAAAAGGTATTCCTCTCATCTTTAAGTGGGATGATTCTAATGATGTTAGATATGAATATGCTGAAAGATATAAAGCCGTTGCGGATAAATATGACGTTGACCTATCAGAGATAGACCATCAGTTAATGATATTAGTTAACTATAACGAAGATAGTAATAAAGCTAAACAAGAGACGCGTGCATTTATTAGTGATTATGTTCTTGAAATGCACCCTAATGAAAATTTCGAAAATAAACTTGAAGAAATAATTGCAGAAAACGCTGTCGGAAATTATACGGAGTGTATAACTGCGGCTAAGTTGGCAATTGAAAAGTGTGGTGCGAAAAGTGTATTGCTGTCCTTTGAACCAATGAATGATTTGATGAGCCAAAAAAATGTAATCAATATTGTTGATGATAATATTAAGAAGTACCACATGGAATATACCTAATAGATTTCGAGTTGCAGCGAGGCGGCAAGTGAACGAATCCCCAGGAGCATAGATAACTATGTGACTGGGGTGAGTGAAAGCAGCCAACAAAGCAGCAGCTTGAAAGATGAAGGGTATAAAAGAGTATGACAGCAGTGCTGCCATACTTTCTAATATTATCTTGAGGAGTAAAACAGGTATGACTTCATATGTTGATAAACAAGAAATTACAGCAAGCTCAGAAATTGATGATTTGATTTTTTCGAGCGATCCATTAGTGTGGTCTTACGACGAGCAGGAAAAAATCAGAAAGAAACTTGTGCTTGATGCATTTCGTAATCATTATAAACATTGTCGAGAATATCGTCACTACTGTCAGGCACACAAAGTAGATGACAATATTACGGAAATTGATGACATACCTGTATTCCCAACATCGGTTTTTAAGTTTACTCGCTTATTAACTTCTCAGGAAAACGAGATTGAAAGTTGGTTTACCAGTAGCGGCACGAATGGTTTAAAAAGTCAGGTGGCGCGTGACAGATTAAGTATTGAGAGACTCTTAGGCTCTGTGAGTTATGGCATGAAATATGTTGGTAGTTGGTTTGATCATCAAATAGAATTAGTCAATTTGGGACCAGATAGATTTAATGCTCATAATATTTGGTTTAAATATGTTATGAGTTTGGTGGAATTGTTATATCCTACGACATTTACCGTAACAGAAGAACGAATAGATTTTGTTAAAACATTGAATAGTCTTGAACGAATAAAAAATCAAGGGAAAGATCTTTGTCTTATTGGTTCGCCATACTTTATTTATTTACTCTGCCATTATATGAAAGATAAAAAAATCTCATTTTCTGGAGATAAAAGCCTTTATATCATAACCGGAGGCGGCTGGAAAAGTTACGAAAAAGAATCTCTGAAACGTGATGATTTCAATCATCTTTTATTTGATACTTTCAATCTCAGTGATATTAGTCAGATCCGAGATATATTTAATCAAGTTGAACTCAACACTTGTTTCTTTGAGGATGAAATGCAGCGTAAACATGTTCCGCCGTGGGTATATGCGCGAGCGCTTGATCCTGAAACGTTGAAACCTGTACCTGATGGAACGCCGGGGTTGATGAGTTATATGGATGCGTCAGCAACCAGTTATCCAGCATTTATTGTTACCGATGATGTCGGGATAATTAGCAGAGAATATGGTAAGTATCCCGGCGTGCTCGTTGAAATTTTACGTCGCGTCAATACGAGGACGCAGAAAGGGTGTGCTTTAAGCTTAACCGAAGCGTTTGATAGTTGACTCGAGCACCACCACCACCACCACT


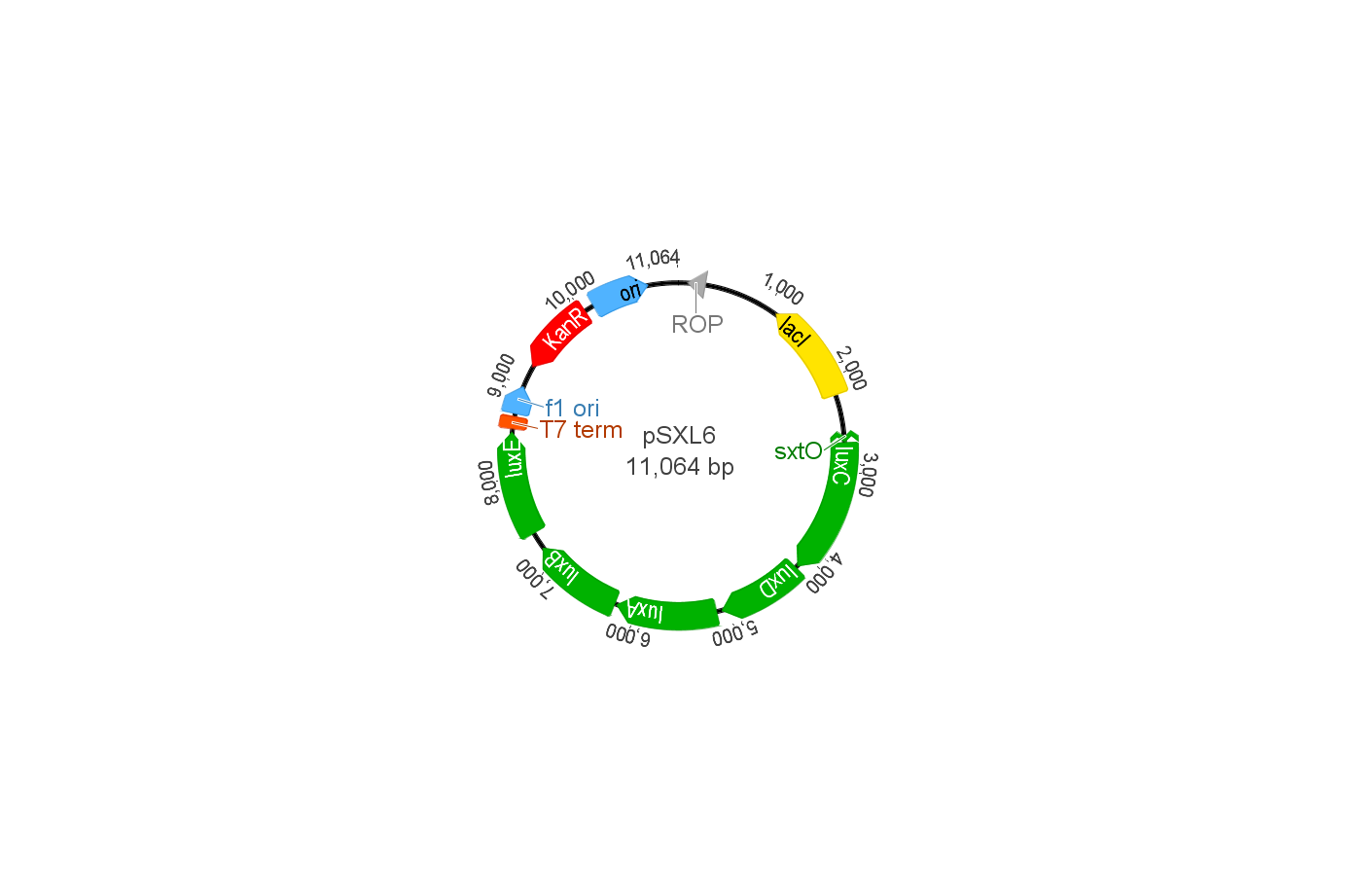


>pSXL6_nucl

TGCGCCCCGACACCCGCCAACACCCGCTGACGCGCCCTGACGGGCTTGTCTGCTCCCGGCATCCGCTTACAGACAAGCTGTGACCGTCTCCGGGAGCTGCATGTGTCAGAGGTTTTCACCGTCATCACCGAAACGCGCGAGGCAGCTGCGGTAAAGCTCATCAGCGTGGTCGTGAAGCGATTCACAGATGTCTGCCTGTTCATCCGCGTCCAGCTCGTTGAGTTTCTCCAGAAGCGTTAATGTCTGGCTTCTGATAAAGCGGGCCATGTTAAGGGCGGTTTTTTCCTGTTTGGTCACTGATGCCTCCGTGTAAGGGGGATTTCTGTTCATGGGGGTAATGATACCGATGAAACGAGAGAGGATGCTCACGATACGGGTTACTGATGATGAACATGCCCGGTTACTGGAACGTTGTGAGGGTAAACAACTGGCGGTATGGATGCGGCGGGACCAGAGAAAAATCACTCAGGGTCAATGCCAGCGCTTCGTTAATACAGATGTAGGTGTTCCACAGGGTAGCCAGCAGCATCCTGCGATGCAGATCCGGAACATAATGGTGCAGGGCGCTGACTTCCGCGTTTCCAGACTTTACGAAACACGGAAACCGAAGACCATTCATGTTGTTGCTCAGGTCGCAGACGTTTTGCAGCAGCAGTCGCTTCACGTTCGCTCGCGTATCGGTGATTCATTCTGCTAACCAGTAAGGCAACCCCGCCAGCCTAGCCGGGTCCTCAACGACAGGAGCACGATCATGCGCACCCGTGGGGCCGCCATGCCGGCGATAATGGCCTGCTTCTCGCCGAAACGTTTGGTGGCGGGACCAGTGACGAAGGCTTGAGCGAGGGCGTGCAAGATTCCGAATACCGCAAGCGACAGGCCGATCATCGTCGCGCTCCAGCGAAAGCGGTCCTCGCCGAAAATGACCCAGAGCGCTGCCGGCACCTGTCCTACGAGTTGCATGATAAAGAAGACAGTCATAAGTGCGGCGACGATAGTCATGCCCCGCGCCCACCGGAAGGAGCTGACTGGGTTGAAGGCTCTCAAGGGCATCGGTCGAGATCCCGGTGCCTAATGAGTGAGCTAACTTACATTAATTGCGTTGCGCTCACTGCCCGCTTTCCAGTCGGGAAACCTGTCGTGCCAGCTGCATTAATGAATCGGCCAACGCGCGGGGAGAGGCGGTTTGCGTATTGGGCGCCAGGGTGGTTTTTCTTTTCACCAGTGAGACGGGCAACAGCTGATTGCCCTTCACCGCCTGGCCCTGAGAGAGTTGCAGCAAGCGGTCCACGCTGGTTTGCCCCAGCAGGCGAAAATCCTGTTTGATGGTGGTTAACGGCGGGATATAACATGAGCTGTCTTCGGTATCGTCGTATCCCACTACCGAGATATCCGCACCAACGCGCAGCCCGGACTCGGTAATGGCGCGCATTGCGCCCAGCGCCATCTGATCGTTGGCAACCAGCATCGCAGTGGGAACGATGCCCTCATTCAGCATTTGCATGGTTTGTTGAAAACCGGACATGGCACTCCAGTCGCCTTCCCGTTCCGCTATCGGCTGAATTTGATTGCGAGTGAGATATTTATGCCAGCCAGCCAGACGCAGACGCGCCGAGACAGAACTTAATGGGCCCGCTAACAGCGCGATTTGCTGGTGACCCAATGCGACCAGATGCTCCACGCCCAGTCGCGTACCGTCTTCATGGGAGAAAATAATACTGTTGATGGGTGTCTGGTCAGAGACATCAAGAAATAACGCCGGAACATTAGTGCAGGCAGCTTCCACAGCAATGGCATCCTGGTCATCCAGCGGATAGTTAATGATCAGCCCACTGACGCGTTGCGCGAGAAGATTGTGCACCGCCGCTTTACAGGCTTCGACGCCGCTTCGTTCTACCATCGACACCACCACGCTGGCACCCAGTTGATCGGCGCGAGATTTAATCGCCGCGACAATTTGCGACGGCGCGTGCAGGGCCAGACTGGAGGTGGCAACGCCAATCAGCAACGACTGTTTGCCCGCCAGTTGTTGTGCCACGCGGTTGGGAATGTAATTCAGCTCCGCCATCGCCGCTTCCACTTTTTCCCGCGTTTTCGCAGAAACGTGGCTGGCCTGGTTCACCACGCGGGAAACGGTCTGATAAGAGACACCGGCATACTCTGCGACATCGTATAACGTTACTGGTTTCACATTCACCACCCTGAATTGACTCTCTTCCGGGCGCTATCATGCCATACCGCGAAAGGTTTTGCGCCATTCGATGGTGTCCGGGATCTCGACGCTCTCCCTTATGCGACTCCTGCATTAGGAAGCAGCCCAGTAGTAGGTTGAGGCCGTTGAGCACCGCCGCCGCAAGGAATGGTGCATGCAAGGAGATGGCGCCCAACAGTCCCCCGGCCACGGGGCCTGCCACCATACCCACGCCGAAACAAGCGCTCATGAGCCCGAAGTGGCGAGCCCGATCTTCCCCATCGGTGATGTCGGCGATATAGGCGCCAGCAACCGCACCTGTGGCGCCGGTGATGCCGGCCACGATGCGTCCGGCGTAGAGGATCGAGATCTCGATCCCGCGAAATGCTATTGTTCTTCCTCCAGCTTTCAATGTTGCTGAATGACTTTCGGTGTCTATTCCCTCGGCCATACCAGAAACAACGGTAAAACCATTTTTTGCTAATGCGGTACTAATTTGACGAGTCATGACTAAAAAAATTTCATTCATTATTAACGGCCAGGTTGAAATCTTTCCCGAAAGTGATGATTTAGTGCAATCCATTAATTTTGGTGATAATAGTGTTTACCTGCCAATATTGAATGACTCTCATGTAAAAAACATTATTGATTGTAATGGAAATAACGAATTACGGTTGCATAACATTGTCAATTTTCTCTATACGGTAGGGCAAAGATGGAAAAATGAAGAATACTCAAGACGCAGGACATACATTCGTGACTTAAAAAAATATATGGGATATTCAGAAGAAATGGCTAAGCTAGAGGCCAATTGGATATCTATGATTTTATGTTCTAAAGGCGGCCTTTATGATGTTGTAGAAAATGAACTTGGTTCTCGCCATATCATGGATGAATGGCTACCTCAGGATGAAAGTTATGTTCGGGCTTTTCCGAAAGGTAAATCTGTACATCTGTTGGCAGGTAATGTTCCATTATCTGGGATCATGTCTATATTACGCGCAATTTTAACTAAGAATCAGTGTATTATAAAAACATCGTCAACCGATCCTTTTACCGCTAATGCATTAGCGTTAAGTTTTATTGATGTAGACCCTAATCATCCGATAACGCGCTCTTTATCTGTTATATATTGGCCCCACCAAGGTGATACATCACTCGCAAAAGAAATTATGCGACATGCGGATGTTATTGTCGCTTGGGGAGGGCCAGATGCGATTAATTGGGCGGTAGAGCATGCGCCATCTTATGCTGATGTGATTAAATTTGGTTCTAAAAAGAGTCTTTGCATTATCGATAATCCTGTTGATTTGACGTCCGCAGCGACAGGTGCGGCTCATGATGTTTGTTTTTACGATCAGCGAGCTTGTTTTTCTGCCCAAAACATATATTACATGGGAAATCATTATGAGGAATTTAAGTTAGCGTTGATAGAAAAACTTAATCTATATGCGCATATATTACCGAATGCCAAAAAAGATTTTGATGAAAAGGCGGCCTATTCTTTAGTTCAAAAAGAAAGCTTGTTTGCTGGATTAAAAGTAGAGGTGGATATTCATCAACGTTGGATGATTATTGAGTCAAATGCAGGTGTGGAATTTAATCAACCACTTGGCAGATGTGTGTACCTTCATCACGTCGATAATATTGAGCAAATATTGCCTTATGTTCAAAAAAATAAGACGCAAACCATATCTATTTTTCCTTGGGAGTCATCATTTAAATATCGAGATGCGTTAGCATTAAAAGGTGCGGAAAGGATTGTAGAAGCAGGAATGAATAACATATTTCGAGTTGGTGGATCTCATGACGGAATGAGACCGTTGCAACGATTAGTGACATATATTTCTCATGAAAGGCCATCTAACTATACGGCTAAGGATGTTGCGGTTGAAATAGAACAGACTCGATTCCTGGAAGAAGATAAGTTCCTTGTATTTGTCCCATAATAGGTAAAAGTATGGAAAATGAATCAAAATATAAAACCATCGACCACGTTATTTGTGTTGAAGGAAATAAAAAAATTCATGTTTGGGAAACGCTGCCAGAAGAAAACAGCCCAAAGAGAAAGAATGCCATTATTATTGCGTCTGGTTTTGCCCGCAGGATGGATCATTTTGCTGGTCTGGCGGAATATTTATCGCGGAATGGATTTCATGTGATCCGCTATGATTCGCTTCACCACGTTGGATTGAGTTCAGGGACAATTGATGAATTTACAATGTCTATAGGAAAGCAGAGCTTGTTAGCAGTGGTTGATTGGTTAACTACACGAAAAATAAATAACTTCGGTATGTTGGCTTCAAGCTTATCTGCGCGGATAGCTTATGCAAGCCTATCTGAAATCAATGCTTCGTTTTTAATCACCGCAGTCGGTGTTGTTAACTTAAGATATTCTCTTGAAAGAGCTTTAGGGTTTGATTATCTCAGTCTACCCATTAATGAATTGCCGGATAATCTAGATTTTGAAGGCCATAAATTGGGTGCTGAAGTCTTTGCGAGAGATTGTCTTGATTTTGGTTGGGAAGATTTAGCTTCTACAATTAATAACATGATGTATCTTGATATACCGTTTATTGCTTTTACTGCAAATAACGATAATTGGGTCAAGCAAGATGAAGTTATCACATTGTTATCAAATATTCGTAGTAATCGATGCAAGATATATTCTTTGTTAGGAAGTTCGCATGACTTGAGTGAAAATTTAGTGGTCCTGCGCAATTTTTATCAATCGGTTACGAAAGCCGCTATCGCGATGGATAATGATCATCTGGATATTGATGTTGATATTACTGAACCGTCATTTGAACATTTAACTATTGCGACAGTCAATGAACGCCGAATGAGAATTGAGATTGAAAATCAAGCAATTTCTCTGTCTTAAAATCTATTGAGATATTCTATCACTCAAATAGCAATATAAGGACTCTCTATGAAATTTGGAAACTTTTTGCTTACATACCAACCTCCCCAATTTTCTCAAACAGAGGTAATGAAACGTTTGGTTAAATTAGGTCGCATCTCTGAGGAGTGTGGTTTTGATACCGTATGGTTACTGGAGCATCATTTCACGGAGTTTGGTTTGCTTGGTAACCCTTATGTCGCTGCTGCATATTTACTTGGCGCGACTAAAAAATTGAATGTAGGAACTGCCGCTATTGTTCTTCCCACAGCCCATCCAGTACGCCAACTTGAAGATGTGAATTTATTGGATCAAATGTCAAAAGGACGATTTCGGTTTGGTATTTGCCGAGGGCTTTACAACAAGGACTTTCGCGTATTCGGCACAGATATGAATAACAGTCGCGCCTTAGCGGAATGCTGGTACGGGCTGATAAAGAATGGCATGACAGAGGGATATATGGAAGCTGATAATGAACATATCAAGTTCCATAAGGTAAAAGTAAACCCCGCGGCGTATAGCAGAGGTGGCGCACCGGTTTATGTGGTGGCTGAATCAGCTTCGACGACTGAGTGGGCTGCTCAATTTGGCCTACCGATGATATTAAGTTGGATTATAAATACTAACGAAAAGAAAGCACAACTTGAGCTTTATAATGAAGTGGCTCAAGAATATGGGCACGATATTCATAATATCGACCATTGCTTATCATATATAACATCTGTAGATCATGACTCAATTAAAGCGAAAGAGATTTGCCGGAAATTTCTGGGGCATTGGTATGATTCTTATGTGAATGCTACGACTATTTTTGATGATTCAGACCAAACAAGAGGTTATGATTTCAATAAAGGGCAGTGGCGTGACTTTGTATTAAAAGGACATAAAGATACTAATCGCCGTATTGATTACAGTTACGAAATCAATCCCGTGGGAACGCCGCAGGAATGTATTGACATAATTCAAAAAGACATTGATGCTACAGGAATATCAAATATTTGTTGTGGATTTGAAGCTAATGGAACAGTAGACGAAATTATTGCTTCCATGAAGCTCTTCCAGTCTGATGTCATGCCATTTCTTAAAGAAAAACAACGTTCGCTATTATATTAGCTAAGGAGAAAGAAATGAAATTTGGATTGTTCTTCCTTAACTTCATCAATTCAACAACTGTTCAAGAACAAAGTATAGTTCGCATGCAGGAAATAACGGAGTATGTTGATAAGTTGAATTTTGAACAGATTTTAGTGTATGAAAATCATTTTTCAGATAATGGTGTTGTCGGCGCTCCTCTGACTGTTTCTGGTTTTCTGCTCGGTTTAACAGAGAAAATTAAAATTGGTTCATTAAATCACATCATTACAACTCATCATCCTGTCGCCATAGCGGAGGAAGCTTGCTTATTGGATCAGTTAAGTGAAGGGAGATTTATTTTAGGGTTTAGTGATTGCGAAAAAAAAGATGAAATGCATTTTTTTAATCGCCCGGTTGAATATCAACAGCAACTATTTGAAGAGTGTTATGAAATCATTAACGATGCTTTAACAACAGGCTATTGTAATCCAGATAACGATTTTTATAGCTTCCCTAAAATATCTGTAAATCCCCATGCTTATACGCCAGGCGGACCTCGGAAATATGTAACAGCAACCAGTCATCATATTGTTGAGTGGGCGGCCAAAAAAGGTATTCCTCTCATCTTTAAGTGGGATGATTCTAATGATGTTAGATATGAATATGCTGAAAGATATAAAGCCGTTGCGGATAAATATGACGTTGACCTATCAGAGATAGACCATCAGTTAATGATATTAGTTAACTATAACGAAGATAGTAATAAAGCTAAACAAGAGACGCGTGCATTTATTAGTGATTATGTTCTTGAAATGCACCCTAATGAAAATTTCGAAAATAAACTTGAAGAAATAATTGCAGAAAACGCTGTCGGAAATTATACGGAGTGTATAACTGCGGCTAAGTTGGCAATTGAAAAGTGTGGTGCGAAAAGTGTATTGCTGTCCTTTGAACCAATGAATGATTTGATGAGCCAAAAAAATGTAATCAATATTGTTGATGATAATATTAAGAAGTACCACATGGAATATACCTAATAGATTTCGAGTTGCAGCGAGGCGGCAAGTGAACGAATCCCCAGGAGCATAGATAACTATGTGACTGGGGTGAGTGAAAGCAGCCAACAAAGCAGCAGCTTGAAAGATGAAGGGTATAAAAGAGTATGACAGCAGTGCTGCCATACTTTCTAATATTATCTTGAGGAGTAAAACAGGTATGACTTCATATGTTGATAAACAAGAAATTACAGCAAGCTCAGAAATTGATGATTTGATTTTTTCGAGCGATCCATTAGTGTGGTCTTACGACGAGCAGGAAAAAATCAGAAAGAAACTTGTGCTTGATGCATTTCGTAATCATTATAAACATTGTCGAGAATATCGTCACTACTGTCAGGCACACAAAGTAGATGACAATATTACGGAAATTGATGACATACCTGTATTCCCAACATCGGTTTTTAAGTTTACTCGCTTATTAACTTCTCAGGAAAACGAGATTGAAAGTTGGTTTACCAGTAGCGGCACGAATGGTTTAAAAAGTCAGGTGGCGCGTGACAGATTAAGTATTGAGAGACTCTTAGGCTCTGTGAGTTATGGCATGAAATATGTTGGTAGTTGGTTTGATCATCAAATAGAATTAGTCAATTTGGGACCAGATAGATTTAATGCTCATAATATTTGGTTTAAATATGTTATGAGTTTGGTGGAATTGTTATATCCTACGACATTTACCGTAACAGAAGAACGAATAGATTTTGTTAAAACATTGAATAGTCTTGAACGAATAAAAAATCAAGGGAAAGATCTTTGTCTTATTGGTTCGCCATACTTTATTTATTTACTCTGCCATTATATGAAAGATAAAAAAATCTCATTTTCTGGAGATAAAAGCCTTTATATCATAACCGGAGGCGGCTGGAAAAGTTACGAAAAAGAATCTCTGAAACGTGATGATTTCAATCATCTTTTATTTGATACTTTCAATCTCAGTGATATTAGTCAGATCCGAGATATATTTAATCAAGTTGAACTCAACACTTGTTTCTTTGAGGATGAAATGCAGCGTAAACATGTTCCGCCGTGGGTATATGCGCGAGCGCTTGATCCTGAAACGTTGAAACCTGTACCTGATGGAACGCCGGGGTTGATGAGTTATATGGATGCGTCAGCAACCAGTTATCCAGCATTTATTGTTACCGATGATGTCGGGATAATTAGCAGAGAATATGGTAAGTATCCCGGCGTGCTCGTTGAAATTTTACGTCGCGTCAATACGAGGACGCAGAAAGGGTGTGCTTTAAGCTTAACCGAAGCGTTTGATAGTTGACTCGAGCACCACCACCACCACCACTGAGATCCGGCTGCTAACAAAGCCCGAAAGGAAGCTGAGTTGGCTGCTGCCACCGCTGAGCAATAACTAGCATAACCCCTTGGGGCCTCTAAACGGGTCTTGAGGGGTTTTTTGCTGAAAGGAGGAACTATATCCGGATTGGCGAATGGGACGCGCCCTGTAGCGGCGCATTAAGCGCGGCGGGTGTGGTGGTTACGCGCAGCGTGACCGCTACACTTGCCAGCGCCCTAGCGCCCGCTCCTTTCGCTTTCTTCCCTTCCTTTCTCGCCACGTTCGCCGGCTTTCCCCGTCAAGCTCTAAATCGGGGGCTCCCTTTAGGGTTCCGATTTAGTGCTTTACGGCACCTCGACCCCAAAAAACTTGATTAGGGTGATGGTTCACGTAGTGGGCCATCGCCCTGATAGACGGTTTTTCGCCCTTTGACGTTGGAGTCCACGTTCTTTAATAGTGGACTCTTGTTCCAAACTGGAACAACACTCAACCCTATCTCGGTCTATTCTTTTGATTTATAAGGGATTTTGCCGATTTCGGCCTATTGGTTAAAAAATGAGCTGATTTAACAAAAATTTAACGCGAATTTTAACAAAATATTAACGTTTACAATTTCAGGTGGCACTTTTCGGGGAAATGTGCGCGGAACCCCTATTTGTTTATTTTTCTAAATACATTCAAATATGTATCCGCTCATGAATTAATTCTTAGAAAAACTCATCGAGCATCAAATGAAACTGCAATTTATTCATATCAGGATTATCAATACCATATTTTTGAAAAAGCCGTTTCTGTAATGAAGGAGAAAACTCACCGAGGCAGTTCCATAGGATGGCAAGATCCTGGTATCGGTCTGCGATTCCGACTCGTCCAACATCAATACAACCTATTAATTTCCCCTCGTCAAAAATAAGGTTATCAAGTGAGAAATCACCATGAGTGACGACTGAATCCGGTGAGAATGGCAAAAGTTTATGCATTTCTTTCCAGACTTGTTCAACAGGCCAGCCATTACGCTCGTCATCAAAATCACTCGCATCAACCAAACCGTTATTCATTCGTGATTGCGCCTGAGCGAGACGAAATACGCGATCGCTGTTAAAAGGACAATTACAAACAGGAATCGAATGCAACCGGCGCAGGAACACTGCCAGCGCATCAACAATATTTTCACCTGAATCAGGATATTCTTCTAATACCTGGAATGCTGTTTTCCCGGGGATCGCAGTGGTGAGTAACCATGCATCATCAGGAGTACGGATAAAATGCTTGATGGTCGGAAGAGGCATAAATTCCGTCAGCCAGTTTAGTCTGACCATCTCATCTGTAACATCATTGGCAACGCTACCTTTGCCATGTTTCAGAAACAACTCTGGCGCATCGGGCTTCCCATACAATCGATAGATTGTCGCACCTGATTGCCCGACATTATCGCGAGCCCATTTATACCCATATAAATCAGCATCCATGTTGGAATTTAATCGCGGCCTAGAGCAAGACGTTTCCCGTTGAATATGGCTCATAACACCCCTTGTATTACTGTTTATGTAAGCAGACAGTTTTATTGTTCATGACCAAAATCCCTTAACGTGAGTTTTCGTTCCACTGAGCGTCAGACCCCGTAGAAAAGATCAAAGGATCTTCTTGAGATCCTTTTTTTCTGCGCGTAATCTGCTGCTTGCAAACAAAAAAACCACCGCTACCAGCGGTGGTTTGTTTGCCGGATCAAGAGCTACCAACTCTTTTTCCGAAGGTAACTGGCTTCAGCAGAGCGCAGATACCAAATACTGTCCTTCTAGTGTAGCCGTAGTTAGGCCACCACTTCAAGAACTCTGTAGCACCGCCTACATACCTCGCTCTGCTAATCCTGTTACCAGTGGCTGCTGCCAGTGGCGATAAGTCGTGTCTTACCGGGTTGGACTCAAGACGATAGTTACCGGATAAGGCGCAGCGGTCGGGCTGAACGGGGGGTTCGTGCACACAGCCCAGCTTGGAGCGAACGACCTACACCGAACTGAGATACCTACAGCGTGAGCTATGAGAAAGCGCCACGCTTCCCGAAGGGAGAAAGGCGGACAGGTATCCGGTAAGCGGCAGGGTCGGAACAGGAGAGCGCACGAGGGAGCTTCCAGGGGGAAACGCCTGGTATCTTTATAGTCCTGTCGGGTTTCGCCACCTCTGACTTGAGCGTCGATTTTTGTGATGCTCGTCAGGGGGGCGGAGCCTATGGAAAAACGCCAGCAACGCGGCCTTTTTACGGTTCCTGGCCTTTTGCTGGCCTTTTGCTCACATGTTCTTTCCTGCGTTATCCCCTGATTCTGTGGATAACCGTATTACCGCCTTTGAGTGAGCTGATACCGCTCGCCGCAGCCGAACGACCGAGCGCAGCGAGTCAGTGAGCGAGGAAGCGGAAGAGCGCCTGATGCGGTATTTTCTCCTTACGCATCTGTGCGGTATTTCACACCGCATATATGGTGCACTCTCAGTACAATCTGCTCTGATGCCGCATAGTTAAGCCAGTATACACTCCGCTATCGCTACGTGACTGGGTCATGGC


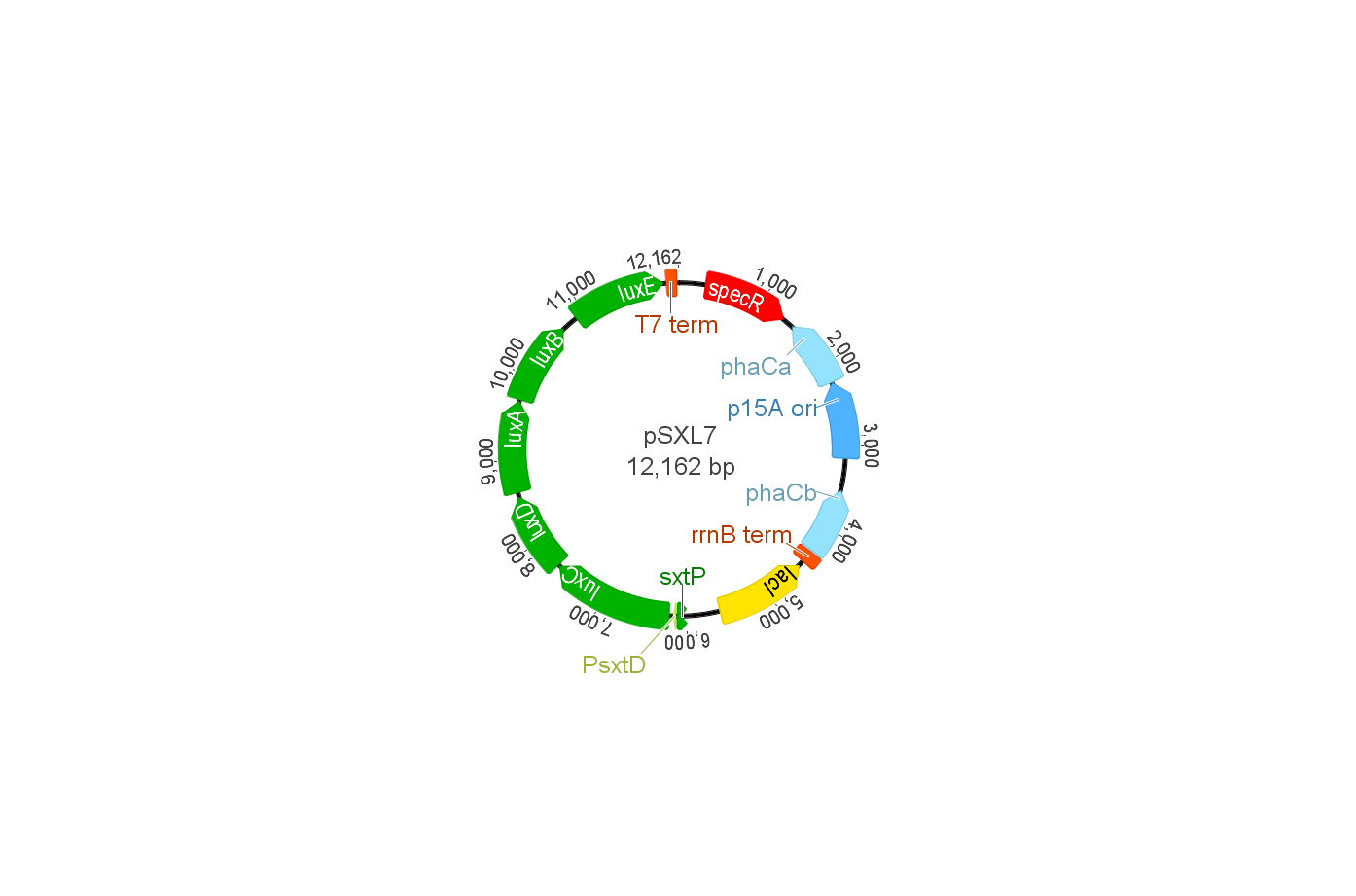


>pSXL7_nucl

GCGGAGATTAACTGTCAAAAAGGTGTAAAAAACCCTTAATTTTTAGCTAAGTAGACACTATTTTTATGTAACGAAAACTTTGTGAATTTATGTAATGTTTAGAGCGATCGCCCAAGGCTACAGTGCTCAACGGAGCCAAAACTACTGTCTACACTGGATTAGAAGACGCTAAATAGCCAGCCAGGACAGAAATGCCTCGACTTCGCTGCTACCCAAGGTTGCCGGGTGACGCACACCGTGGAAACGGATGAAGGCACGAACCCAGTGGACATAAGCCTGTTCGGTTCGTAAGCTGTAATGCAAGTAGCGTATGCGCTCACGCAACTGGTCCAGAACCTTGACCGAACGCAGCGGTGGTAACGGCGCAGTGGCGGTTTTCATGGCTTGTTATGACTGTTTTTTTGGGGTACAGTCTATGCCTCGGGCATCCAAGCAGCAAGCGCGTTACGCCGTGGGTCGATGTTTGATGTTATGGAGCAGCAACGATGTTACGCAGCAGGGCAGTCGCCCTAAAACAAAGTTAAACATTATGAGGGAAGCGGTGATCGCCGAAGTATCGACTCAACTATCAGAGGTAGTTGGCGTCATCGAGCGCCATCTCGAACCGACGTTGCTGGCCGTACATTTGTACGGCTCCGCAGTGGATGGCGGCCTGAAGCCACACAGTGATATTGATTTGCTGGTTACGGTGACCGTAAGGCTTGATGAAACAACGCGGCGAGCTTTGATCAACGACCTTTTGGAAACTTCGGCTTCCCCTGGAGAGAGCGAGATTCTCCGCGCTGTAGAAGTCACCATTGTTGTGCACGACGACATCATTCCGTGGCGTTATCCAGCTAAGCGCGAACTGCAATTTGGAGAATGGCAGCGCAATGACATTCTTGCTGGTATCTTCGAGCCAGCCACGATCGACATTGATCTGGCTATCTTGCTGACAAAAGCAAGAGAACATAGCGTTGCCTTGGTAGGTCCAGCGGCGGAGGAACTCTTTGATCCGGTTCCTGAACAGGATCTATTTGAGGCGCTAAATGAAACCTTAACGCTATGGAACTCGCCGCCCGACTGGGCTGGCGATGAGCGAAATGTAGTGCTTACGTTGTCCCGCATTTGGTACAGCGCAGTAACCGGCAAAATCGCGCCGAAGGATGTCGCTGCCGACTGGGCAATGGAGCGCCTGCCGGCCCAGTATCAGCCCGTCATACTTGAAGCTAGACAGGCTTATCTTGGACAAGAAGAAGATCGCTTGGCCTCGCGCGCAGATCAGTTGGAAGAATTTGTCCACTACGTGAAAGGCGAGATCACCAAGGTAGTCGGCAAATAACCTAATTGCCGGAATTACAGCAGTCTGAATAATCAAAAAGCGCCTCGAATGGGGCGCTTTTTGATGCTTGGATATTGATTGTAGACAACCTTTGGACTTGCGATCGCTAGGGATTATCTAGATTAGCCTGGGTTTGCTTCTGTTTCTCCCAATCGTTTTTTCAAACTTTTAACTTCTTTCCGCAACTGATAAATGGTTTGATGAATTTCATCCACCTCTGAGCGGGTAGGGAGGTTCAGCATTTTTAACCATGCTTCTAGAATCTCCTGTTGCTGAATGCGATAACGATTCAAGGCATTGATGAATTTGCCCCGTACTTTCAGATTTTTTTCCTCACAAAAAGCTTCTTCAAACACTTGGTCAGCGGCGATCGCCCACCGTTGTTGAAATTCCTTCCAGGTTTTAACGGGTTTATCTTCCTTAGCCCGGGCCAGTAAATCTTCCATCAAAGCAGCAAAGCCCCGGTATTGAATATCTGCTTCCAGTAATTGATAGTCTGCCATGGCCTGGGATAACTTAACCCAATCGTCAAAGGCCCGCAATAATTTGCCATTCATTTCCCGACTGGGCCCCAGCAAAGGCGATCGCATAAAACTGCTCAGGTTTTTGTTGTAGAGGGCATCGCCGTAGAGATTATTTAAATCTAACCAAGCATGGATGTCCCCGGTGGGTAATTTGCCCAGGGGGGCGACGCTACTCTGCCAGGTGGAGAGCCAGAGTTGGGAAAATCTTTGTACTTCCTTGATGTAACACTGCCATAAACCATCCATGTCCCCTTGGAGAGCTTGGGTGGTGGCGGTATATTGCTCAATTTGGGTTTGTAGCTGTTTTAGGTAGCCCTGCACTGCCCCTGGCGCCGAACCATTATCCAATTTAGGCCACAGGCTTTGCCAAGCTTCAAAAGAAAGCTTAAGTAACTCTGCTAGCGGAGTGTATACTGGCTTACTATGTTGGCACTGATGAGGGTGTCAGTGAAGTGCTTCATGTGGCAGGAGAAAAAAGGCTGCACCGGTGCGTCAGCAGAATATGTGATACAGGATATATTCCGCTTCCTCGCTCACTGACTCGCTACGCTCGGTCGTTCGACTGCGGCGAGCGGAAATGGCTTACGAACGGGGCGGAGATTTCCTGGAAGATGCCAGGAAGATACTTAACAGGGAAGTGAGAGGGCCGCGGCAAAGCCGTTTTTCCATAGGCTCCGCCCCCCTGACAAGCATCACGAAATCTGACGCTCAAATCAGTGGTGGCGAAACCCGACAGGACTATAAAGATACCAGGCGTTTCCCCCTGGCGGCTCCCTCGTGCGCTCTCCTGTTCCTGCCTTTCGGTTTACCGGTGTCATTCCGCTGTTATGGCCGCGTTTGTCTCATTCCACGCCTGACACTCAGTTCCGGGTAGGCAGTTCGCTCCAAGCTGGACTGTATGCACGAACCCCCCGTTCAGTCCGACCGCTGCGCCTTATCCGGTAACTATCGTCTTGAGTCCAACCCGGAAAGACATGCAAAAGCACCACTGGCAGCAGCCACTGGTAATTGATTTAGAGGAGTTAGTCTTGAAGTCATGCGCCGGTTAAGGCTAAACTGAAAGGACAAGTTTTGGTGACTGCGCTCCTCCAAGCCAGTTACCTCGGTTCAAAGAGTTGGTAGCTCAGAGAACCTTCGAAAAACCGCCCTGCAAGGCGGTTTTTTCGTTTTCAGAGCAAGAGATTACGCGCAGACCAAAACGATCTCAAGAAGATCATCTTATTAATCAGATAAAATATTTCTAGATTTCAGTGCAATTTATCTCTTCAAATGTAGCACCTGAAGTCAGCCCCATACGATATAAGTTGTGGGGTCTGACGCTCAGTGGAACGAAAACTCACGTTAAGGGATTTTGGTCATGAGATTATCGATCTTCCGCTGCATAACCCTGCTTCGGGGTCATTATAGCGATTTTTTCGGTATATCCATCCTTTTTCGCACGATATACAGGATTTTGCCAAAGGGTTCGTGTAGACTTTCCTTGGTGTATCCAACGGCGTCAGCCGGGCAGGATAGGTGAAGTAGGCCCACCCGCGAGCGGGTGTTCCTTCTTCACTGTCCCTTATTCGCACCTGGCGGTGCTCAACGGGAATCCTGCTCTGCGAGGCTGGCCGGCTACCGCCGGCGTAACAGATGAGGGCAAGCGGATGGCTGATGAAACCAAGCCAACCAGCGGCTACCATTACCACCCTGGTACAAAATATTCCCCAATTGGCTCGGATTTTAACCTACCACGTGGTTGCCGGGAAGTTTACCCAAGCTGATTTATGTCGCCTATCAACTGTTGATTCAGTAGAGGGCTCCCCGATCGCCATCGACTGTACGGAGGGGTTTGAAGTGAAAAATGCCACGGTAATCATCCCCGATATCGAAGCGGACAACGGCATTATCCATGTGATTGACAACGTGATTTTAATGGGATAGGCATGGTGTGCGACCTGTTCAAAACCTTTAGCCCAGGGGAGGATACCTTGGTATTCCAACTGCGGAGCTAATGGACTAGGATGGAAAATAGTGATCCCAGCGGTAATGCTGTTGGTAGGAGCCATCTGTGAACAACTTTTCCCCCAAATTTGTTACCGTCAACCAATCAGGCCTGGGCTGTGGCTTGACTGTCCTTGCCGTGGCCATGCTCCTCAGTGCCATTGGTCTGAAATGGGTTGTCAATGGCATTGCTCTGTTGATTTTGCTATTGTTTCTGGCACCAACGGTGGGCTTTTTGGTATTCCGCTGGTGGCTGAAGAGAAAATTGGTGGATGGGGAGTGTCCCGTTTGTCAGTATCACTTCACTGGTTTTGACGGTCAGCCATGCCGATGCCCCAGTTGCGGGGAAGAGTTGGAAGTGAGCAATGGACAATTTACCCGCCCCACTCCCCCTGGAACTATTGACGTACAAGCGGTGGATGTATCAGTTAAAGCACTAGATGAGTCCTGAGTGTTGCCCTAATGTATTCCAGCAAAATTTAAGGCCCAGTCTTTCGACTGAGCCTTTCGTTTTATTTGATGCCTGGCAGTTCCCTACTCTCGCATGGGGAGACCCCACACTACCATCGGCGCTACGGCGTTTCACTTCTGAGTTCGGCATGGGGTCAGGTGGGACCACCGCGCTACTGCCGCCAGGCACTCGATGCATGGTACCCGGTGCCTAATGAGTGAGCTAACTTACATTAATTGCGTTGCGCTCACTGCCCGCTTTCCAGTCGGGAAACCTGTCGTGCCAGCTGCATTAATGAATCGGCCAACGCGCGGGGAGAGGCGGTTTGCGTATTGGGCGCCAGGGTGGTTTTTCTTTTCACCAGTGAGACGGGCAACAGCTGATTGCCCTTCACCGCCTGGCCCTGAGAGAGTTGCAGCAAGCGGTCCACGCTGGTTTGCCCCAGCAGGCGAAAATCCTGTTTGATGGTGGTTAACGGCGGGATATAACATGAGCTGTCTTCGGTATCGTCGTATCCCACTACCGAGATATCCGCACCAACGCGCAGCCCGGACTCGGTAATGGCGCGCATTGCGCCCAGCGCCATCTGATCGTTGGCAACCAGCATCGCAGTGGGAACGATGCCCTCATTCAGCATTTGCATGGTTTGTTGAAAACCGGACATGGCACTCCAGTCGCCTTCCCGTTCCGCTATCGGCTGAATTTGATTGCGAGTGAGATATTTATGCCAGCCAGCCAGACGCAGACGCGCCGAGACAGAACTTAATGGGCCCGCTAACAGCGCGATTTGCTGGTGACCCAATGCGACCAGATGCTCCACGCCCAGTCGCGTACCGTCTTCATGGGAGAAAATAATACTGTTGATGGGTGTCTGGTCAGAGACATCAAGAAATAACGCCGGAACATTAGTGCAGGCAGCTTCCACAGCAATGGCATCCTGGTCATCCAGCGGATAGTTAATGATCAGCCCACTGACGCGTTGCGCGAGAAGATTGTGCACCGCCGCTTTACAGGCTTCGACGCCGCTTCGTTCTACCATCGACACCACCACGCTGGCACCCAGTTGATCGGCGCGAGATTTAATCGCCGCGACAATTTGCGACGGCGCGTGCAGGGCCAGACTGGAGGTGGCAACGCCAATCAGCAACGACTGTTTGCCCGCCAGTTGTTGTGCCACGCGGTTGGGAATGTAATTCAGCTCCGCCATCGCCGCTTCCACTTTTTCCCGCGTTTTCGCAGAAACGTGGCTGGCCTGGTTCACCACGCGGGAAACGGTCTGATAAGAGACACCGGCATACTCTGCGACATCGTATAACGTTACTGGTTTCACATTCACCACCCTGAATTGACTCTCTTCCGGGCGCTATCATGCCATACCGCGAAAGGTTTTGCGCCATTCGATGGTGTCCGGGATCTCGACGCTCTCCCTTATGCGACTCCTGCATTAGGAAGCAGCCCAGTAGTAGGTTGAGGCCGTTGAGCACCGCCGCCGCAAGGAATGGTGCATGCAAGGAGATGGCGCCCAACAGTCCCCCGGCCACGGGGCCTGCCACCATACCCACGCCGAAACAAGCGCTCATGAGCCCGAAGTGGCGAGCCCGATCTTCCCCATCGGTGATGTCGGCGATATAGGCGCCAGCAACCGCACCTGTGGCGCCGGTGATGCCGGCCACGATGCGTCCGGCGTAGAGGATCGAGATCTCGATCCCGCGAAATCAGAAAGCTTTTCATTCTTCAGATAATCGTTAGTTGTATCTGCTAATGCAACAGTGGTTATACATAGGAATGTGCTGACAAACAAGAGTGCTGCTAGAACTAACGAACTGAGCCTTTTCATGAGTTGTCTTGTGGAAATTTGTTAATAAGAGTATACTTGACTAGTATACACTAAGGTTACCAATGATAGATACAAAATGACTAAAAAAATTTCATTCATTATTAACGGCCAGGTTGAAATCTTTCCCGAAAGTGATGATTTAGTGCAATCCATTAATTTTGGTGATAATAGTGTTTACCTGCCAATATTGAATGACTCTCATGTAAAAAACATTATTGATTGTAATGGAAATAACGAATTACGGTTGCATAACATTGTCAATTTTCTCTATACGGTAGGGCAAAGATGGAAAAATGAAGAATACTCAAGACGCAGGACATACATTCGTGACTTAAAAAAATATATGGGATATTCAGAAGAAATGGCTAAGCTAGAGGCCAATTGGATATCTATGATTTTATGTTCTAAAGGCGGCCTTTATGATGTTGTAGAAAATGAACTTGGTTCTCGCCATATCATGGATGAATGGCTACCTCAGGATGAAAGTTATGTTCGGGCTTTTCCGAAAGGTAAATCTGTACATCTGTTGGCAGGTAATGTTCCATTATCTGGGATCATGTCTATATTACGCGCAATTTTAACTAAGAATCAGTGTATTATAAAAACATCGTCAACCGATCCTTTTACCGCTAATGCATTAGCGTTAAGTTTTATTGATGTAGACCCTAATCATCCGATAACGCGCTCTTTATCTGTTATATATTGGCCCCACCAAGGTGATACATCACTCGCAAAAGAAATTATGCGACATGCGGATGTTATTGTCGCTTGGGGAGGGCCAGATGCGATTAATTGGGCGGTAGAGCATGCGCCATCTTATGCTGATGTGATTAAATTTGGTTCTAAAAAGAGTCTTTGCATTATCGATAATCCTGTTGATTTGACGTCCGCAGCGACAGGTGCGGCTCATGATGTTTGTTTTTACGATCAGCGAGCTTGTTTTTCTGCCCAAAACATATATTACATGGGAAATCATTATGAGGAATTTAAGTTAGCGTTGATAGAAAAACTTAATCTATATGCGCATATATTACCGAATGCCAAAAAAGATTTTGATGAAAAGGCGGCCTATTCTTTAGTTCAAAAAGAAAGCTTGTTTGCTGGATTAAAAGTAGAGGTGGATATTCATCAACGTTGGATGATTATTGAGTCAAATGCAGGTGTGGAATTTAATCAACCACTTGGCAGATGTGTGTACCTTCATCACGTCGATAATATTGAGCAAATATTGCCTTATGTTCAAAAAAATAAGACGCAAACCATATCTATTTTTCCTTGGGAGTCATCATTTAAATATCGAGATGCGTTAGCATTAAAAGGTGCGGAAAGGATTGTAGAAGCAGGAATGAATAACATATTTCGAGTTGGTGGATCTCATGACGGAATGAGACCGTTGCAACGATTAGTGACATATATTTCTCATGAAAGGCCATCTAACTATACGGCTAAGGATGTTGCGGTTGAAATAGAACAGACTCGATTCCTGGAAGAAGATAAGTTCCTTGTATTTGTCCCATAATAGGTAAAAGTATGGAAAATGAATCAAAATATAAAACCATCGACCACGTTATTTGTGTTGAAGGAAATAAAAAAATTCATGTTTGGGAAACGCTGCCAGAAGAAAACAGCCCAAAGAGAAAGAATGCCATTATTATTGCGTCTGGTTTTGCCCGCAGGATGGATCATTTTGCTGGTCTGGCGGAATATTTATCGCGGAATGGATTTCATGTGATCCGCTATGATTCGCTTCACCACGTTGGATTGAGTTCAGGGACAATTGATGAATTTACAATGTCTATAGGAAAGCAGAGCTTGTTAGCAGTGGTTGATTGGTTAACTACACGAAAAATAAATAACTTCGGTATGTTGGCTTCAAGCTTATCTGCGCGGATAGCTTATGCAAGCCTATCTGAAATCAATGCTTCGTTTTTAATCACCGCAGTCGGTGTTGTTAACTTAAGATATTCTCTTGAAAGAGCTTTAGGGTTTGATTATCTCAGTCTACCCATTAATGAATTGCCGGATAATCTAGATTTTGAAGGCCATAAATTGGGTGCTGAAGTCTTTGCGAGAGATTGTCTTGATTTTGGTTGGGAAGATTTAGCTTCTACAATTAATAACATGATGTATCTTGATATACCGTTTATTGCTTTTACTGCAAATAACGATAATTGGGTCAAGCAAGATGAAGTTATCACATTGTTATCAAATATTCGTAGTAATCGATGCAAGATATATTCTTTGTTAGGAAGTTCGCATGACTTGAGTGAAAATTTAGTGGTCCTGCGCAATTTTTATCAATCGGTTACGAAAGCCGCTATCGCGATGGATAATGATCATCTGGATATTGATGTTGATATTACTGAACCGTCATTTGAACATTTAACTATTGCGACAGTCAATGAACGCCGAATGAGAATTGAGATTGAAAATCAAGCAATTTCTCTGTCTTAAAATCTATTGAGATATTCTATCACTCAAATAGCAATATAAGGACTCTCTATGAAATTTGGAAACTTTTTGCTTACATACCAACCTCCCCAATTTTCTCAAACAGAGGTAATGAAACGTTTGGTTAAATTAGGTCGCATCTCTGAGGAGTGTGGTTTTGATACCGTATGGTTACTGGAGCATCATTTCACGGAGTTTGGTTTGCTTGGTAACCCTTATGTCGCTGCTGCATATTTACTTGGCGCGACTAAAAAATTGAATGTAGGAACTGCCGCTATTGTTCTTCCCACAGCCCATCCAGTACGCCAACTTGAAGATGTGAATTTATTGGATCAAATGTCAAAAGGACGATTTCGGTTTGGTATTTGCCGAGGGCTTTACAACAAGGACTTTCGCGTATTCGGCACAGATATGAATAACAGTCGCGCCTTAGCGGAATGCTGGTACGGGCTGATAAAGAATGGCATGACAGAGGGATATATGGAAGCTGATAATGAACATATCAAGTTCCATAAGGTAAAAGTAAACCCCGCGGCGTATAGCAGAGGTGGCGCACCGGTTTATGTGGTGGCTGAATCAGCTTCGACGACTGAGTGGGCTGCTCAATTTGGCCTACCGATGATATTAAGTTGGATTATAAATACTAACGAAAAGAAAGCACAACTTGAGCTTTATAATGAAGTGGCTCAAGAATATGGGCACGATATTCATAATATCGACCATTGCTTATCATATATAACATCTGTAGATCATGACTCAATTAAAGCGAAAGAGATTTGCCGGAAATTTCTGGGGCATTGGTATGATTCTTATGTGAATGCTACGACTATTTTTGATGATTCAGACCAAACAAGAGGTTATGATTTCAATAAAGGGCAGTGGCGTGACTTTGTATTAAAAGGACATAAAGATACTAATCGCCGTATTGATTACAGTTACGAAATCAATCCCGTGGGAACGCCGCAGGAATGTATTGACATAATTCAAAAAGACATTGATGCTACAGGAATATCAAATATTTGTTGTGGATTTGAAGCTAATGGAACAGTAGACGAAATTATTGCTTCCATGAAGCTCTTCCAGTCTGATGTCATGCCATTTCTTAAAGAAAAACAACGTTCGCTATTATATTAGCTAAGGAGAAAGAAATGAAATTTGGATTGTTCTTCCTTAACTTCATCAATTCAACAACTGTTCAAGAACAAAGTATAGTTCGCATGCAGGAAATAACGGAGTATGTTGATAAGTTGAATTTTGAACAGATTTTAGTGTATGAAAATCATTTTTCAGATAATGGTGTTGTCGGCGCTCCTCTGACTGTTTCTGGTTTTCTGCTCGGTTTAACAGAGAAAATTAAAATTGGTTCATTAAATCACATCATTACAACTCATCATCCTGTCGCCATAGCGGAGGAAGCTTGCTTATTGGATCAGTTAAGTGAAGGGAGATTTATTTTAGGGTTTAGTGATTGCGAAAAAAAAGATGAAATGCATTTTTTTAATCGCCCGGTTGAATATCAACAGCAACTATTTGAAGAGTGTTATGAAATCATTAACGATGCTTTAACAACAGGCTATTGTAATCCAGATAACGATTTTTATAGCTTCCCTAAAATATCTGTAAATCCCCATGCTTATACGCCAGGCGGACCTCGGAAATATGTAACAGCAACCAGTCATCATATTGTTGAGTGGGCGGCCAAAAAAGGTATTCCTCTCATCTTTAAGTGGGATGATTCTAATGATGTTAGATATGAATATGCTGAAAGATATAAAGCCGTTGCGGATAAATATGACGTTGACCTATCAGAGATAGACCATCAGTTAATGATATTAGTTAACTATAACGAAGATAGTAATAAAGCTAAACAAGAGACGCGTGCATTTATTAGTGATTATGTTCTTGAAATGCACCCTAATGAAAATTTCGAAAATAAACTTGAAGAAATAATTGCAGAAAACGCTGTCGGAAATTATACGGAGTGTATAACTGCGGCTAAGTTGGCAATTGAAAAGTGTGGTGCGAAAAGTGTATTGCTGTCCTTTGAACCAATGAATGATTTGATGAGCCAAAAAAATGTAATCAATATTGTTGATGATAATATTAAGAAGTACCACATGGAATATACCTAATAGATTTCGAGTTGCAGCGAGGCGGCAAGTGAACGAATCCCCAGGAGCATAGATAACTATGTGACTGGGGTGAGTGAAAGCAGCCAACAAAGCAGCAGCTTGAAAGATGAAGGGTATAAAAGAGTATGACAGCAGTGCTGCCATACTTTCTAATATTATCTTGAGGAGTAAAACAGGTATGACTTCATATGTTGATAAACAAGAAATTACAGCAAGCTCAGAAATTGATGATTTGATTTTTTCGAGCGATCCATTAGTGTGGTCTTACGACGAGCAGGAAAAAATCAGAAAGAAACTTGTGCTTGATGCATTTCGTAATCATTATAAACATTGTCGAGAATATCGTCACTACTGTCAGGCACACAAAGTAGATGACAATATTACGGAAATTGATGACATACCTGTATTCCCAACATCGGTTTTTAAGTTTACTCGCTTATTAACTTCTCAGGAAAACGAGATTGAAAGTTGGTTTACCAGTAGCGGCACGAATGGTTTAAAAAGTCAGGTGGCGCGTGACAGATTAAGTATTGAGAGACTCTTAGGCTCTGTGAGTTATGGCATGAAATATGTTGGTAGTTGGTTTGATCATCAAATAGAATTAGTCAATTTGGGACCAGATAGATTTAATGCTCATAATATTTGGTTTAAATATGTTATGAGTTTGGTGGAATTGTTATATCCTACGACATTTACCGTAACAGAAGAACGAATAGATTTTGTTAAAACATTGAATAGTCTTGAACGAATAAAAAATCAAGGGAAAGATCTTTGTCTTATTGGTTCGCCATACTTTATTTATTTACTCTGCCATTATATGAAAGATAAAAAAATCTCATTTTCTGGAGATAAAAGCCTTTATATCATAACCGGAGGCGGCTGGAAAAGTTACGAAAAAGAATCTCTGAAACGTGATGATTTCAATCATCTTTTATTTGATACTTTCAATCTCAGTGATATTAGTCAGATCCGAGATATATTTAATCAAGTTGAACTCAACACTTGTTTCTTTGAGGATGAAATGCAGCGTAAACATGTTCCGCCGTGGGTATATGCGCGAGCGCTTGATCCTGAAACGTTGAAACCTGTACCTGATGGAACGCCGGGGTTGATGAGTTATATGGATGCGTCAGCAACCAGTTATCCAGCATTTATTGTTACCGATGATGTCGGGATAATTAGCAGAGAATATGGTAAGTATCCCGGCGTGCTCGTTGAAATTTTACGTCGCGTCAATACGAGGACGCAGAAAGGGTGTGCTTTAAGCTTAACCGAAGCGTTTGATAGTTGACTCGAGCACCACCACCACCACCACTGAGATCCGGCTGCTAACAAAGCCCGAAAGGAAGCTGAGTTGGCTGCTGCCACCGCTGAGCAATAACTAGCATAACCCCTTGGGGCCTCTAAACGGGTCTTGAGGGGTTTTTTGCTGAAAGGAGGAACTATATCCGGATTGGGCGGCC


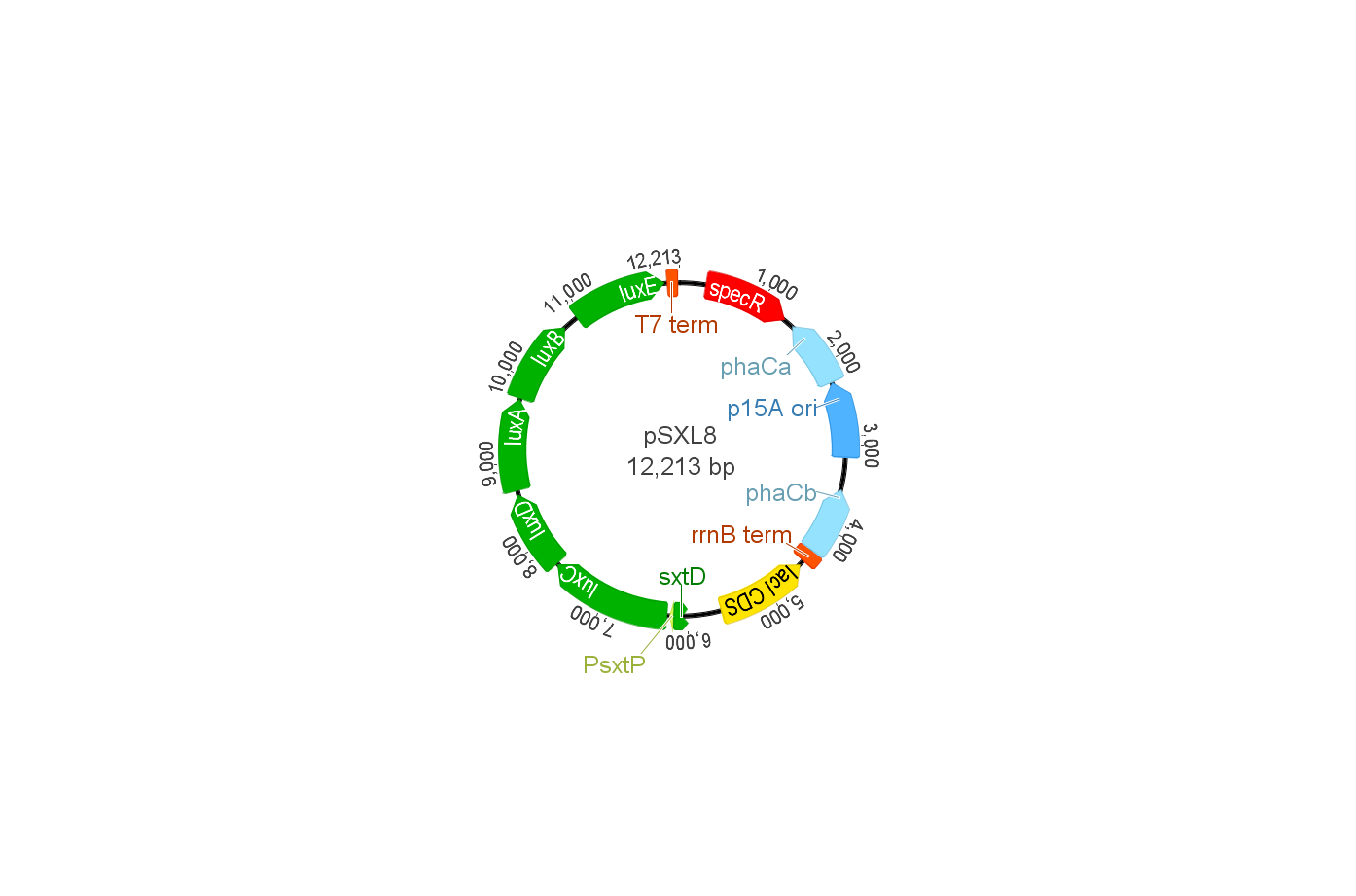


>pSXL8_nucl

GCGGAGATTAACTGTCAAAAAGGTGTAAAAAACCCTTAATTTTTAGCTAAGTAGACACTATTTTTATGTAACGAAAACTTTGTGAATTTATGTAATGTTTAGAGCGATCGCCCAAGGCTACAGTGCTCAACGGAGCCAAAACTACTGTCTACACTGGATTAGAAGACGCTAAATAGCCAGCCAGGACAGAAATGCCTCGACTTCGCTGCTACCCAAGGTTGCCGGGTGACGCACACCGTGGAAACGGATGAAGGCACGAACCCAGTGGACATAAGCCTGTTCGGTTCGTAAGCTGTAATGCAAGTAGCGTATGCGCTCACGCAACTGGTCCAGAACCTTGACCGAACGCAGCGGTGGTAACGGCGCAGTGGCGGTTTTCATGGCTTGTTATGACTGTTTTTTTGGGGTACAGTCTATGCCTCGGGCATCCAAGCAGCAAGCGCGTTACGCCGTGGGTCGATGTTTGATGTTATGGAGCAGCAACGATGTTACGCAGCAGGGCAGTCGCCCTAAAACAAAGTTAAACATTATGAGGGAAGCGGTGATCGCCGAAGTATCGACTCAACTATCAGAGGTAGTTGGCGTCATCGAGCGCCATCTCGAACCGACGTTGCTGGCCGTACATTTGTACGGCTCCGCAGTGGATGGCGGCCTGAAGCCACACAGTGATATTGATTTGCTGGTTACGGTGACCGTAAGGCTTGATGAAACAACGCGGCGAGCTTTGATCAACGACCTTTTGGAAACTTCGGCTTCCCCTGGAGAGAGCGAGATTCTCCGCGCTGTAGAAGTCACCATTGTTGTGCACGACGACATCATTCCGTGGCGTTATCCAGCTAAGCGCGAACTGCAATTTGGAGAATGGCAGCGCAATGACATTCTTGCTGGTATCTTCGAGCCAGCCACGATCGACATTGATCTGGCTATCTTGCTGACAAAAGCAAGAGAACATAGCGTTGCCTTGGTAGGTCCAGCGGCGGAGGAACTCTTTGATCCGGTTCCTGAACAGGATCTATTTGAGGCGCTAAATGAAACCTTAACGCTATGGAACTCGCCGCCCGACTGGGCTGGCGATGAGCGAAATGTAGTGCTTACGTTGTCCCGCATTTGGTACAGCGCAGTAACCGGCAAAATCGCGCCGAAGGATGTCGCTGCCGACTGGGCAATGGAGCGCCTGCCGGCCCAGTATCAGCCCGTCATACTTGAAGCTAGACAGGCTTATCTTGGACAAGAAGAAGATCGCTTGGCCTCGCGCGCAGATCAGTTGGAAGAATTTGTCCACTACGTGAAAGGCGAGATCACCAAGGTAGTCGGCAAATAACCTAATTGCCGGAATTACAGCAGTCTGAATAATCAAAAAGCGCCTCGAATGGGGCGCTTTTTGATGCTTGGATATTGATTGTAGACAACCTTTGGACTTGCGATCGCTAGGGATTATCTAGATTAGCCTGGGTTTGCTTCTGTTTCTCCCAATCGTTTTTTCAAACTTTTAACTTCTTTCCGCAACTGATAAATGGTTTGATGAATTTCATCCACCTCTGAGCGGGTAGGGAGGTTCAGCATTTTTAACCATGCTTCTAGAATCTCCTGTTGCTGAATGCGATAACGATTCAAGGCATTGATGAATTTGCCCCGTACTTTCAGATTTTTTTCCTCACAAAAAGCTTCTTCAAACACTTGGTCAGCGGCGATCGCCCACCGTTGTTGAAATTCCTTCCAGGTTTTAACGGGTTTATCTTCCTTAGCCCGGGCCAGTAAATCTTCCATCAAAGCAGCAAAGCCCCGGTATTGAATATCTGCTTCCAGTAATTGATAGTCTGCCATGGCCTGGGATAACTTAACCCAATCGTCAAAGGCCCGCAATAATTTGCCATTCATTTCCCGACTGGGCCCCAGCAAAGGCGATCGCATAAAACTGCTCAGGTTTTTGTTGTAGAGGGCATCGCCGTAGAGATTATTTAAATCTAACCAAGCATGGATGTCCCCGGTGGGTAATTTGCCCAGGGGGGCGACGCTACTCTGCCAGGTGGAGAGCCAGAGTTGGGAAAATCTTTGTACTTCCTTGATGTAACACTGCCATAAACCATCCATGTCCCCTTGGAGAGCTTGGGTGGTGGCGGTATATTGCTCAATTTGGGTTTGTAGCTGTTTTAGGTAGCCCTGCACTGCCCCTGGCGCCGAACCATTATCCAATTTAGGCCACAGGCTTTGCCAAGCTTCAAAAGAAAGCTTAAGTAACTCTGCTAGCGGAGTGTATACTGGCTTACTATGTTGGCACTGATGAGGGTGTCAGTGAAGTGCTTCATGTGGCAGGAGAAAAAAGGCTGCACCGGTGCGTCAGCAGAATATGTGATACAGGATATATTCCGCTTCCTCGCTCACTGACTCGCTACGCTCGGTCGTTCGACTGCGGCGAGCGGAAATGGCTTACGAACGGGGCGGAGATTTCCTGGAAGATGCCAGGAAGATACTTAACAGGGAAGTGAGAGGGCCGCGGCAAAGCCGTTTTTCCATAGGCTCCGCCCCCCTGACAAGCATCACGAAATCTGACGCTCAAATCAGTGGTGGCGAAACCCGACAGGACTATAAAGATACCAGGCGTTTCCCCCTGGCGGCTCCCTCGTGCGCTCTCCTGTTCCTGCCTTTCGGTTTACCGGTGTCATTCCGCTGTTATGGCCGCGTTTGTCTCATTCCACGCCTGACACTCAGTTCCGGGTAGGCAGTTCGCTCCAAGCTGGACTGTATGCACGAACCCCCCGTTCAGTCCGACCGCTGCGCCTTATCCGGTAACTATCGTCTTGAGTCCAACCCGGAAAGACATGCAAAAGCACCACTGGCAGCAGCCACTGGTAATTGATTTAGAGGAGTTAGTCTTGAAGTCATGCGCCGGTTAAGGCTAAACTGAAAGGACAAGTTTTGGTGACTGCGCTCCTCCAAGCCAGTTACCTCGGTTCAAAGAGTTGGTAGCTCAGAGAACCTTCGAAAAACCGCCCTGCAAGGCGGTTTTTTCGTTTTCAGAGCAAGAGATTACGCGCAGACCAAAACGATCTCAAGAAGATCATCTTATTAATCAGATAAAATATTTCTAGATTTCAGTGCAATTTATCTCTTCAAATGTAGCACCTGAAGTCAGCCCCATACGATATAAGTTGTGGGGTCTGACGCTCAGTGGAACGAAAACTCACGTTAAGGGATTTTGGTCATGAGATTATCGATCTTCCGCTGCATAACCCTGCTTCGGGGTCATTATAGCGATTTTTTCGGTATATCCATCCTTTTTCGCACGATATACAGGATTTTGCCAAAGGGTTCGTGTAGACTTTCCTTGGTGTATCCAACGGCGTCAGCCGGGCAGGATAGGTGAAGTAGGCCCACCCGCGAGCGGGTGTTCCTTCTTCACTGTCCCTTATTCGCACCTGGCGGTGCTCAACGGGAATCCTGCTCTGCGAGGCTGGCCGGCTACCGCCGGCGTAACAGATGAGGGCAAGCGGATGGCTGATGAAACCAAGCCAACCAGCGGCTACCATTACCACCCTGGTACAAAATATTCCCCAATTGGCTCGGATTTTAACCTACCACGTGGTTGCCGGGAAGTTTACCCAAGCTGATTTATGTCGCCTATCAACTGTTGATTCAGTAGAGGGCTCCCCGATCGCCATCGACTGTACGGAGGGGTTTGAAGTGAAAAATGCCACGGTAATCATCCCCGATATCGAAGCGGACAACGGCATTATCCATGTGATTGACAACGTGATTTTAATGGGATAGGCATGGTGTGCGACCTGTTCAAAACCTTTAGCCCAGGGGAGGATACCTTGGTATTCCAACTGCGGAGCTAATGGACTAGGATGGAAAATAGTGATCCCAGCGGTAATGCTGTTGGTAGGAGCCATCTGTGAACAACTTTTCCCCCAAATTTGTTACCGTCAACCAATCAGGCCTGGGCTGTGGCTTGACTGTCCTTGCCGTGGCCATGCTCCTCAGTGCCATTGGTCTGAAATGGGTTGTCAATGGCATTGCTCTGTTGATTTTGCTATTGTTTCTGGCACCAACGGTGGGCTTTTTGGTATTCCGCTGGTGGCTGAAGAGAAAATTGGTGGATGGGGAGTGTCCCGTTTGTCAGTATCACTTCACTGGTTTTGACGGTCAGCCATGCCGATGCCCCAGTTGCGGGGAAGAGTTGGAAGTGAGCAATGGACAATTTACCCGCCCCACTCCCCCTGGAACTATTGACGTACAAGCGGTGGATGTATCAGTTAAAGCACTAGATGAGTCCTGAGTGTTGCCCTAATGTATTCCAGCAAAATTTAAGGCCCAGTCTTTCGACTGAGCCTTTCGTTTTATTTGATGCCTGGCAGTTCCCTACTCTCGCATGGGGAGACCCCACACTACCATCGGCGCTACGGCGTTTCACTTCTGAGTTCGGCATGGGGTCAGGTGGGACCACCGCGCTACTGCCGCCAGGCACTCGATGCATGGTACCCGGTGCCTAATGAGTGAGCTAACTTACATTAATTGCGTTGCGCTCACTGCCCGCTTTCCAGTCGGGAAACCTGTCGTGCCAGCTGCATTAATGAATCGGCCAACGCGCGGGGAGAGGCGGTTTGCGTATTGGGCGCCAGGGTGGTTTTTCTTTTCACCAGTGAGACGGGCAACAGCTGATTGCCCTTCACCGCCTGGCCCTGAGAGAGTTGCAGCAAGCGGTCCACGCTGGTTTGCCCCAGCAGGCGAAAATCCTGTTTGATGGTGGTTAACGGCGGGATATAACATGAGCTGTCTTCGGTATCGTCGTATCCCACTACCGAGATATCCGCACCAACGCGCAGCCCGGACTCGGTAATGGCGCGCATTGCGCCCAGCGCCATCTGATCGTTGGCAACCAGCATCGCAGTGGGAACGATGCCCTCATTCAGCATTTGCATGGTTTGTTGAAAACCGGACATGGCACTCCAGTCGCCTTCCCGTTCCGCTATCGGCTGAATTTGATTGCGAGTGAGATATTTATGCCAGCCAGCCAGACGCAGACGCGCCGAGACAGAACTTAATGGGCCCGCTAACAGCGCGATTTGCTGGTGACCCAATGCGACCAGATGCTCCACGCCCAGTCGCGTACCGTCTTCATGGGAGAAAATAATACTGTTGATGGGTGTCTGGTCAGAGACATCAAGAAATAACGCCGGAACATTAGTGCAGGCAGCTTCCACAGCAATGGCATCCTGGTCATCCAGCGGATAGTTAATGATCAGCCCACTGACGCGTTGCGCGAGAAGATTGTGCACCGCCGCTTTACAGGCTTCGACGCCGCTTCGTTCTACCATCGACACCACCACGCTGGCACCCAGTTGATCGGCGCGAGATTTAATCGCCGCGACAATTTGCGACGGCGCGTGCAGGGCCAGACTGGAGGTGGCAACGCCAATCAGCAACGACTGTTTGCCCGCCAGTTGTTGTGCCACGCGGTTGGGAATGTAATTCAGCTCCGCCATCGCCGCTTCCACTTTTTCCCGCGTTTTCGCAGAAACGTGGCTGGCCTGGTTCACCACGCGGGAAACGGTCTGATAAGAGACACCGGCATACTCTGCGACATCGTATAACGTTACTGGTTTCACATTCACCACCCTGAATTGACTCTCTTCCGGGCGCTATCATGCCATACCGCGAAAGGTTTTGCGCCATTCGATGGTGTCCGGGATCTCGACGCTCTCCCTTATGCGACTCCTGCATTAGGAAGCAGCCCAGTAGTAGGTTGAGGCCGTTGAGCACCGCCGCCGCAAGGAATGGTGCATGCAAGGAGATGGCGCCCAACAGTCCCCCGGCCACGGGGCCTGCCACCATACCCACGCCGAAACAAGCGCTCATGAGCCCGAAGTGGCGAGCCCGATCTTCCCCATCGGTGATGTCGGCGATATAGGCGCCAGCAACCGCACCTGTGGCGCCGGTGATGCCGGCCACGATGCGTCCGGCGTAGAGGATCGAGATCTCGATCCCGCGAAATCATAGATAATTCTAAAGACGAGTGATATTCCTGCCCATCCCAATTCCTTAAAGAAATTAGCTTTGTATTTAATCTCATGTAAAGGATAGCGAATTTCCCAAAGCCAGAATATCATACCCGTAATTAAAATTACAGTCCAATCTCTTAATAGGATTAATATTGTATCTATCATTTTGTATCTATCATTGGTAACCTTAGTGTATACTAGTCAAGTATACTCTTATTAACAAATTTCCACAAGACAACTCATGACTAAAAAAATTTCATTCATTATTAACGGCCAGGTTGAAATCTTTCCCGAAAGTGATGATTTAGTGCAATCCATTAATTTTGGTGATAATAGTGTTTACCTGCCAATATTGAATGACTCTCATGTAAAAAACATTATTGATTGTAATGGAAATAACGAATTACGGTTGCATAACATTGTCAATTTTCTCTATACGGTAGGGCAAAGATGGAAAAATGAAGAATACTCAAGACGCAGGACATACATTCGTGACTTAAAAAAATATATGGGATATTCAGAAGAAATGGCTAAGCTAGAGGCCAATTGGATATCTATGATTTTATGTTCTAAAGGCGGCCTTTATGATGTTGTAGAAAATGAACTTGGTTCTCGCCATATCATGGATGAATGGCTACCTCAGGATGAAAGTTATGTTCGGGCTTTTCCGAAAGGTAAATCTGTACATCTGTTGGCAGGTAATGTTCCATTATCTGGGATCATGTCTATATTACGCGCAATTTTAACTAAGAATCAGTGTATTATAAAAACATCGTCAACCGATCCTTTTACCGCTAATGCATTAGCGTTAAGTTTTATTGATGTAGACCCTAATCATCCGATAACGCGCTCTTTATCTGTTATATATTGGCCCCACCAAGGTGATACATCACTCGCAAAAGAAATTATGCGACATGCGGATGTTATTGTCGCTTGGGGAGGGCCAGATGCGATTAATTGGGCGGTAGAGCATGCGCCATCTTATGCTGATGTGATTAAATTTGGTTCTAAAAAGAGTCTTTGCATTATCGATAATCCTGTTGATTTGACGTCCGCAGCGACAGGTGCGGCTCATGATGTTTGTTTTTACGATCAGCGAGCTTGTTTTTCTGCCCAAAACATATATTACATGGGAAATCATTATGAGGAATTTAAGTTAGCGTTGATAGAAAAACTTAATCTATATGCGCATATATTACCGAATGCCAAAAAAGATTTTGATGAAAAGGCGGCCTATTCTTTAGTTCAAAAAGAAAGCTTGTTTGCTGGATTAAAAGTAGAGGTGGATATTCATCAACGTTGGATGATTATTGAGTCAAATGCAGGTGTGGAATTTAATCAACCACTTGGCAGATGTGTGTACCTTCATCACGTCGATAATATTGAGCAAATATTGCCTTATGTTCAAAAAAATAAGACGCAAACCATATCTATTTTTCCTTGGGAGTCATCATTTAAATATCGAGATGCGTTAGCATTAAAAGGTGCGGAAAGGATTGTAGAAGCAGGAATGAATAACATATTTCGAGTTGGTGGATCTCATGACGGAATGAGACCGTTGCAACGATTAGTGACATATATTTCTCATGAAAGGCCATCTAACTATACGGCTAAGGATGTTGCGGTTGAAATAGAACAGACTCGATTCCTGGAAGAAGATAAGTTCCTTGTATTTGTCCCATAATAGGTAAAAGTATGGAAAATGAATCAAAATATAAAACCATCGACCACGTTATTTGTGTTGAAGGAAATAAAAAAATTCATGTTTGGGAAACGCTGCCAGAAGAAAACAGCCCAAAGAGAAAGAATGCCATTATTATTGCGTCTGGTTTTGCCCGCAGGATGGATCATTTTGCTGGTCTGGCGGAATATTTATCGCGGAATGGATTTCATGTGATCCGCTATGATTCGCTTCACCACGTTGGATTGAGTTCAGGGACAATTGATGAATTTACAATGTCTATAGGAAAGCAGAGCTTGTTAGCAGTGGTTGATTGGTTAACTACACGAAAAATAAATAACTTCGGTATGTTGGCTTCAAGCTTATCTGCGCGGATAGCTTATGCAAGCCTATCTGAAATCAATGCTTCGTTTTTAATCACCGCAGTCGGTGTTGTTAACTTAAGATATTCTCTTGAAAGAGCTTTAGGGTTTGATTATCTCAGTCTACCCATTAATGAATTGCCGGATAATCTAGATTTTGAAGGCCATAAATTGGGTGCTGAAGTCTTTGCGAGAGATTGTCTTGATTTTGGTTGGGAAGATTTAGCTTCTACAATTAATAACATGATGTATCTTGATATACCGTTTATTGCTTTTACTGCAAATAACGATAATTGGGTCAAGCAAGATGAAGTTATCACATTGTTATCAAATATTCGTAGTAATCGATGCAAGATATATTCTTTGTTAGGAAGTTCGCATGACTTGAGTGAAAATTTAGTGGTCCTGCGCAATTTTTATCAATCGGTTACGAAAGCCGCTATCGCGATGGATAATGATCATCTGGATATTGATGTTGATATTACTGAACCGTCATTTGAACATTTAACTATTGCGACAGTCAATGAACGCCGAATGAGAATTGAGATTGAAAATCAAGCAATTTCTCTGTCTTAAAATCTATTGAGATATTCTATCACTCAAATAGCAATATAAGGACTCTCTATGAAATTTGGAAACTTTTTGCTTACATACCAACCTCCCCAATTTTCTCAAACAGAGGTAATGAAACGTTTGGTTAAATTAGGTCGCATCTCTGAGGAGTGTGGTTTTGATACCGTATGGTTACTGGAGCATCATTTCACGGAGTTTGGTTTGCTTGGTAACCCTTATGTCGCTGCTGCATATTTACTTGGCGCGACTAAAAAATTGAATGTAGGAACTGCCGCTATTGTTCTTCCCACAGCCCATCCAGTACGCCAACTTGAAGATGTGAATTTATTGGATCAAATGTCAAAAGGACGATTTCGGTTTGGTATTTGCCGAGGGCTTTACAACAAGGACTTTCGCGTATTCGGCACAGATATGAATAACAGTCGCGCCTTAGCGGAATGCTGGTACGGGCTGATAAAGAATGGCATGACAGAGGGATATATGGAAGCTGATAATGAACATATCAAGTTCCATAAGGTAAAAGTAAACCCCGCGGCGTATAGCAGAGGTGGCGCACCGGTTTATGTGGTGGCTGAATCAGCTTCGACGACTGAGTGGGCTGCTCAATTTGGCCTACCGATGATATTAAGTTGGATTATAAATACTAACGAAAAGAAAGCACAACTTGAGCTTTATAATGAAGTGGCTCAAGAATATGGGCACGATATTCATAATATCGACCATTGCTTATCATATATAACATCTGTAGATCATGACTCAATTAAAGCGAAAGAGATTTGCCGGAAATTTCTGGGGCATTGGTATGATTCTTATGTGAATGCTACGACTATTTTTGATGATTCAGACCAAACAAGAGGTTATGATTTCAATAAAGGGCAGTGGCGTGACTTTGTATTAAAAGGACATAAAGATACTAATCGCCGTATTGATTACAGTTACGAAATCAATCCCGTGGGAACGCCGCAGGAATGTATTGACATAATTCAAAAAGACATTGATGCTACAGGAATATCAAATATTTGTTGTGGATTTGAAGCTAATGGAACAGTAGACGAAATTATTGCTTCCATGAAGCTCTTCCAGTCTGATGTCATGCCATTTCTTAAAGAAAAACAACGTTCGCTATTATATTAGCTAAGGAGAAAGAAATGAAATTTGGATTGTTCTTCCTTAACTTCATCAATTCAACAACTGTTCAAGAACAAAGTATAGTTCGCATGCAGGAAATAACGGAGTATGTTGATAAGTTGAATTTTGAACAGATTTTAGTGTATGAAAATCATTTTTCAGATAATGGTGTTGTCGGCGCTCCTCTGACTGTTTCTGGTTTTCTGCTCGGTTTAACAGAGAAAATTAAAATTGGTTCATTAAATCACATCATTACAACTCATCATCCTGTCGCCATAGCGGAGGAAGCTTGCTTATTGGATCAGTTAAGTGAAGGGAGATTTATTTTAGGGTTTAGTGATTGCGAAAAAAAAGATGAAATGCATTTTTTTAATCGCCCGGTTGAATATCAACAGCAACTATTTGAAGAGTGTTATGAAATCATTAACGATGCTTTAACAACAGGCTATTGTAATCCAGATAACGATTTTTATAGCTTCCCTAAAATATCTGTAAATCCCCATGCTTATACGCCAGGCGGACCTCGGAAATATGTAACAGCAACCAGTCATCATATTGTTGAGTGGGCGGCCAAAAAAGGTATTCCTCTCATCTTTAAGTGGGATGATTCTAATGATGTTAGATATGAATATGCTGAAAGATATAAAGCCGTTGCGGATAAATATGACGTTGACCTATCAGAGATAGACCATCAGTTAATGATATTAGTTAACTATAACGAAGATAGTAATAAAGCTAAACAAGAGACGCGTGCATTTATTAGTGATTATGTTCTTGAAATGCACCCTAATGAAAATTTCGAAAATAAACTTGAAGAAATAATTGCAGAAAACGCTGTCGGAAATTATACGGAGTGTATAACTGCGGCTAAGTTGGCAATTGAAAAGTGTGGTGCGAAAAGTGTATTGCTGTCCTTTGAACCAATGAATGATTTGATGAGCCAAAAAAATGTAATCAATATTGTTGATGATAATATTAAGAAGTACCACATGGAATATACCTAATAGATTTCGAGTTGCAGCGAGGCGGCAAGTGAACGAATCCCCAGGAGCATAGATAACTATGTGACTGGGGTGAGTGAAAGCAGCCAACAAAGCAGCAGCTTGAAAGATGAAGGGTATAAAAGAGTATGACAGCAGTGCTGCCATACTTTCTAATATTATCTTGAGGAGTAAAACAGGTATGACTTCATATGTTGATAAACAAGAAATTACAGCAAGCTCAGAAATTGATGATTTGATTTTTTCGAGCGATCCATTAGTGTGGTCTTACGACGAGCAGGAAAAAATCAGAAAGAAACTTGTGCTTGATGCATTTCGTAATCATTATAAACATTGTCGAGAATATCGTCACTACTGTCAGGCACACAAAGTAGATGACAATATTACGGAAATTGATGACATACCTGTATTCCCAACATCGGTTTTTAAGTTTACTCGCTTATTAACTTCTCAGGAAAACGAGATTGAAAGTTGGTTTACCAGTAGCGGCACGAATGGTTTAAAAAGTCAGGTGGCGCGTGACAGATTAAGTATTGAGAGACTCTTAGGCTCTGTGAGTTATGGCATGAAATATGTTGGTAGTTGGTTTGATCATCAAATAGAATTAGTCAATTTGGGACCAGATAGATTTAATGCTCATAATATTTGGTTTAAATATGTTATGAGTTTGGTGGAATTGTTATATCCTACGACATTTACCGTAACAGAAGAACGAATAGATTTTGTTAAAACATTGAATAGTCTTGAACGAATAAAAAATCAAGGGAAAGATCTTTGTCTTATTGGTTCGCCATACTTTATTTATTTACTCTGCCATTATATGAAAGATAAAAAAATCTCATTTTCTGGAGATAAAAGCCTTTATATCATAACCGGAGGCGGCTGGAAAAGTTACGAAAAAGAATCTCTGAAACGTGATGATTTCAATCATCTTTTATTTGATACTTTCAATCTCAGTGATATTAGTCAGATCCGAGATATATTTAATCAAGTTGAACTCAACACTTGTTTCTTTGAGGATGAAATGCAGCGTAAACATGTTCCGCCGTGGGTATATGCGCGAGCGCTTGATCCTGAAACGTTGAAACCTGTACCTGATGGAACGCCGGGGTTGATGAGTTATATGGATGCGTCAGCAACCAGTTATCCAGCATTTATTGTTACCGATGATGTCGGGATAATTAGCAGAGAATATGGTAAGTATCCCGGCGTGCTCGTTGAAATTTTACGTCGCGTCAATACGAGGACGCAGAAAGGGTGTGCTTTAAGCTTAACCGAAGCGTTTGATAGTTGACTCGAGCACCACCACCACCACCACTGAGATCCGGCTGCTAACAAAGCCCGAAAGGAAGCTGAGTTGGCTGCTGCCACCGCTGAGCAATAACTAGCATAACCCCTTGGGGCCTCTAAACGGGTCTTGAGGGGTTTTTTGCTGAAAGGAGGAACTATATCCGGATTGGGCGGCC


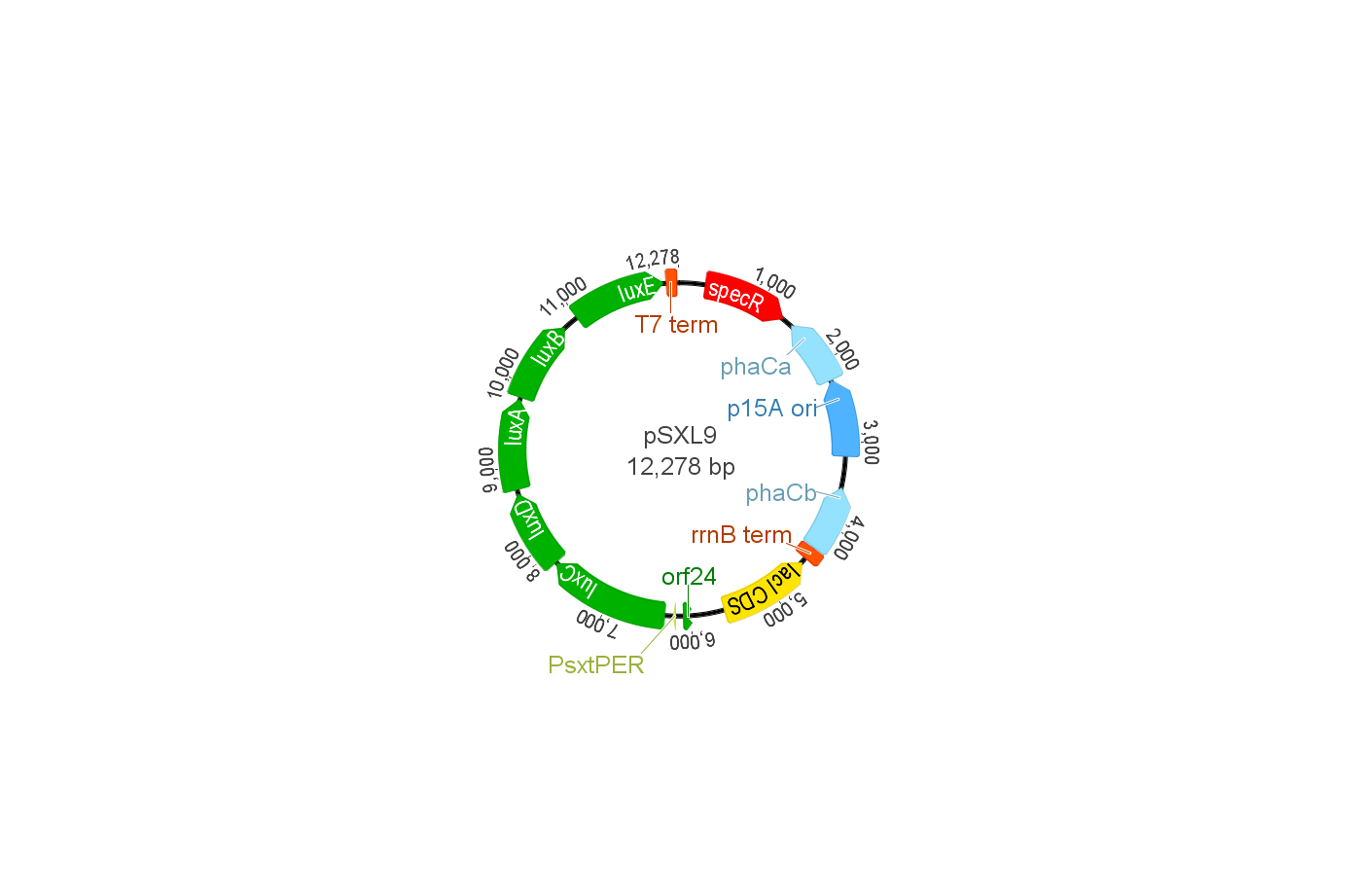


>pSXL9_nucl

GCGGAGATTAACTGTCAAAAAGGTGTAAAAAACCCTTAATTTTTAGCTAAGTAGACACTATTTTTATGTAACGAAAACTTTGTGAATTTATGTAATGTTTAGAGCGATCGCCCAAGGCTACAGTGCTCAACGGAGCCAAAACTACTGTCTACACTGGATTAGAAGACGCTAAATAGCCAGCCAGGACAGAAATGCCTCGACTTCGCTGCTACCCAAGGTTGCCGGGTGACGCACACCGTGGAAACGGATGAAGGCACGAACCCAGTGGACATAAGCCTGTTCGGTTCGTAAGCTGTAATGCAAGTAGCGTATGCGCTCACGCAACTGGTCCAGAACCTTGACCGAACGCAGCGGTGGTAACGGCGCAGTGGCGGTTTTCATGGCTTGTTATGACTGTTTTTTTGGGGTACAGTCTATGCCTCGGGCATCCAAGCAGCAAGCGCGTTACGCCGTGGGTCGATGTTTGATGTTATGGAGCAGCAACGATGTTACGCAGCAGGGCAGTCGCCCTAAAACAAAGTTAAACATTATGAGGGAAGCGGTGATCGCCGAAGTATCGACTCAACTATCAGAGGTAGTTGGCGTCATCGAGCGCCATCTCGAACCGACGTTGCTGGCCGTACATTTGTACGGCTCCGCAGTGGATGGCGGCCTGAAGCCACACAGTGATATTGATTTGCTGGTTACGGTGACCGTAAGGCTTGATGAAACAACGCGGCGAGCTTTGATCAACGACCTTTTGGAAACTTCGGCTTCCCCTGGAGAGAGCGAGATTCTCCGCGCTGTAGAAGTCACCATTGTTGTGCACGACGACATCATTCCGTGGCGTTATCCAGCTAAGCGCGAACTGCAATTTGGAGAATGGCAGCGCAATGACATTCTTGCTGGTATCTTCGAGCCAGCCACGATCGACATTGATCTGGCTATCTTGCTGACAAAAGCAAGAGAACATAGCGTTGCCTTGGTAGGTCCAGCGGCGGAGGAACTCTTTGATCCGGTTCCTGAACAGGATCTATTTGAGGCGCTAAATGAAACCTTAACGCTATGGAACTCGCCGCCCGACTGGGCTGGCGATGAGCGAAATGTAGTGCTTACGTTGTCCCGCATTTGGTACAGCGCAGTAACCGGCAAAATCGCGCCGAAGGATGTCGCTGCCGACTGGGCAATGGAGCGCCTGCCGGCCCAGTATCAGCCCGTCATACTTGAAGCTAGACAGGCTTATCTTGGACAAGAAGAAGATCGCTTGGCCTCGCGCGCAGATCAGTTGGAAGAATTTGTCCACTACGTGAAAGGCGAGATCACCAAGGTAGTCGGCAAATAACCTAATTGCCGGAATTACAGCAGTCTGAATAATCAAAAAGCGCCTCGAATGGGGCGCTTTTTGATGCTTGGATATTGATTGTAGACAACCTTTGGACTTGCGATCGCTAGGGATTATCTAGATTAGCCTGGGTTTGCTTCTGTTTCTCCCAATCGTTTTTTCAAACTTTTAACTTCTTTCCGCAACTGATAAATGGTTTGATGAATTTCATCCACCTCTGAGCGGGTAGGGAGGTTCAGCATTTTTAACCATGCTTCTAGAATCTCCTGTTGCTGAATGCGATAACGATTCAAGGCATTGATGAATTTGCCCCGTACTTTCAGATTTTTTTCCTCACAAAAAGCTTCTTCAAACACTTGGTCAGCGGCGATCGCCCACCGTTGTTGAAATTCCTTCCAGGTTTTAACGGGTTTATCTTCCTTAGCCCGGGCCAGTAAATCTTCCATCAAAGCAGCAAAGCCCCGGTATTGAATATCTGCTTCCAGTAATTGATAGTCTGCCATGGCCTGGGATAACTTAACCCAATCGTCAAAGGCCCGCAATAATTTGCCATTCATTTCCCGACTGGGCCCCAGCAAAGGCGATCGCATAAAACTGCTCAGGTTTTTGTTGTAGAGGGCATCGCCGTAGAGATTATTTAAATCTAACCAAGCATGGATGTCCCCGGTGGGTAATTTGCCCAGGGGGGCGACGCTACTCTGCCAGGTGGAGAGCCAGAGTTGGGAAAATCTTTGTACTTCCTTGATGTAACACTGCCATAAACCATCCATGTCCCCTTGGAGAGCTTGGGTGGTGGCGGTATATTGCTCAATTTGGGTTTGTAGCTGTTTTAGGTAGCCCTGCACTGCCCCTGGCGCCGAACCATTATCCAATTTAGGCCACAGGCTTTGCCAAGCTTCAAAAGAAAGCTTAAGTAACTCTGCTAGCGGAGTGTATACTGGCTTACTATGTTGGCACTGATGAGGGTGTCAGTGAAGTGCTTCATGTGGCAGGAGAAAAAAGGCTGCACCGGTGCGTCAGCAGAATATGTGATACAGGATATATTCCGCTTCCTCGCTCACTGACTCGCTACGCTCGGTCGTTCGACTGCGGCGAGCGGAAATGGCTTACGAACGGGGCGGAGATTTCCTGGAAGATGCCAGGAAGATACTTAACAGGGAAGTGAGAGGGCCGCGGCAAAGCCGTTTTTCCATAGGCTCCGCCCCCCTGACAAGCATCACGAAATCTGACGCTCAAATCAGTGGTGGCGAAACCCGACAGGACTATAAAGATACCAGGCGTTTCCCCCTGGCGGCTCCCTCGTGCGCTCTCCTGTTCCTGCCTTTCGGTTTACCGGTGTCATTCCGCTGTTATGGCCGCGTTTGTCTCATTCCACGCCTGACACTCAGTTCCGGGTAGGCAGTTCGCTCCAAGCTGGACTGTATGCACGAACCCCCCGTTCAGTCCGACCGCTGCGCCTTATCCGGTAACTATCGTCTTGAGTCCAACCCGGAAAGACATGCAAAAGCACCACTGGCAGCAGCCACTGGTAATTGATTTAGAGGAGTTAGTCTTGAAGTCATGCGCCGGTTAAGGCTAAACTGAAAGGACAAGTTTTGGTGACTGCGCTCCTCCAAGCCAGTTACCTCGGTTCAAAGAGTTGGTAGCTCAGAGAACCTTCGAAAAACCGCCCTGCAAGGCGGTTTTTTCGTTTTCAGAGCAAGAGATTACGCGCAGACCAAAACGATCTCAAGAAGATCATCTTATTAATCAGATAAAATATTTCTAGATTTCAGTGCAATTTATCTCTTCAAATGTAGCACCTGAAGTCAGCCCCATACGATATAAGTTGTGGGGTCTGACGCTCAGTGGAACGAAAACTCACGTTAAGGGATTTTGGTCATGAGATTATCGATCTTCCGCTGCATAACCCTGCTTCGGGGTCATTATAGCGATTTTTTCGGTATATCCATCCTTTTTCGCACGATATACAGGATTTTGCCAAAGGGTTCGTGTAGACTTTCCTTGGTGTATCCAACGGCGTCAGCCGGGCAGGATAGGTGAAGTAGGCCCACCCGCGAGCGGGTGTTCCTTCTTCACTGTCCCTTATTCGCACCTGGCGGTGCTCAACGGGAATCCTGCTCTGCGAGGCTGGCCGGCTACCGCCGGCGTAACAGATGAGGGCAAGCGGATGGCTGATGAAACCAAGCCAACCAGCGGCTACCATTACCACCCTGGTACAAAATATTCCCCAATTGGCTCGGATTTTAACCTACCACGTGGTTGCCGGGAAGTTTACCCAAGCTGATTTATGTCGCCTATCAACTGTTGATTCAGTAGAGGGCTCCCCGATCGCCATCGACTGTACGGAGGGGTTTGAAGTGAAAAATGCCACGGTAATCATCCCCGATATCGAAGCGGACAACGGCATTATCCATGTGATTGACAACGTGATTTTAATGGGATAGGCATGGTGTGCGACCTGTTCAAAACCTTTAGCCCAGGGGAGGATACCTTGGTATTCCAACTGCGGAGCTAATGGACTAGGATGGAAAATAGTGATCCCAGCGGTAATGCTGTTGGTAGGAGCCATCTGTGAACAACTTTTCCCCCAAATTTGTTACCGTCAACCAATCAGGCCTGGGCTGTGGCTTGACTGTCCTTGCCGTGGCCATGCTCCTCAGTGCCATTGGTCTGAAATGGGTTGTCAATGGCATTGCTCTGTTGATTTTGCTATTGTTTCTGGCACCAACGGTGGGCTTTTTGGTATTCCGCTGGTGGCTGAAGAGAAAATTGGTGGATGGGGAGTGTCCCGTTTGTCAGTATCACTTCACTGGTTTTGACGGTCAGCCATGCCGATGCCCCAGTTGCGGGGAAGAGTTGGAAGTGAGCAATGGACAATTTACCCGCCCCACTCCCCCTGGAACTATTGACGTACAAGCGGTGGATGTATCAGTTAAAGCACTAGATGAGTCCTGAGTGTTGCCCTAATGTATTCCAGCAAAATTTAAGGCCCAGTCTTTCGACTGAGCCTTTCGTTTTATTTGATGCCTGGCAGTTCCCTACTCTCGCATGGGGAGACCCCACACTACCATCGGCGCTACGGCGTTTCACTTCTGAGTTCGGCATGGGGTCAGGTGGGACCACCGCGCTACTGCCGCCAGGCACTCGATGCATGGTACCCGGTGCCTAATGAGTGAGCTAACTTACATTAATTGCGTTGCGCTCACTGCCCGCTTTCCAGTCGGGAAACCTGTCGTGCCAGCTGCATTAATGAATCGGCCAACGCGCGGGGAGAGGCGGTTTGCGTATTGGGCGCCAGGGTGGTTTTTCTTTTCACCAGTGAGACGGGCAACAGCTGATTGCCCTTCACCGCCTGGCCCTGAGAGAGTTGCAGCAAGCGGTCCACGCTGGTTTGCCCCAGCAGGCGAAAATCCTGTTTGATGGTGGTTAACGGCGGGATATAACATGAGCTGTCTTCGGTATCGTCGTATCCCACTACCGAGATATCCGCACCAACGCGCAGCCCGGACTCGGTAATGGCGCGCATTGCGCCCAGCGCCATCTGATCGTTGGCAACCAGCATCGCAGTGGGAACGATGCCCTCATTCAGCATTTGCATGGTTTGTTGAAAACCGGACATGGCACTCCAGTCGCCTTCCCGTTCCGCTATCGGCTGAATTTGATTGCGAGTGAGATATTTATGCCAGCCAGCCAGACGCAGACGCGCCGAGACAGAACTTAATGGGCCCGCTAACAGCGCGATTTGCTGGTGACCCAATGCGACCAGATGCTCCACGCCCAGTCGCGTACCGTCTTCATGGGAGAAAATAATACTGTTGATGGGTGTCTGGTCAGAGACATCAAGAAATAACGCCGGAACATTAGTGCAGGCAGCTTCCACAGCAATGGCATCCTGGTCATCCAGCGGATAGTTAATGATCAGCCCACTGACGCGTTGCGCGAGAAGATTGTGCACCGCCGCTTTACAGGCTTCGACGCCGCTTCGTTCTACCATCGACACCACCACGCTGGCACCCAGTTGATCGGCGCGAGATTTAATCGCCGCGACAATTTGCGACGGCGCGTGCAGGGCCAGACTGGAGGTGGCAACGCCAATCAGCAACGACTGTTTGCCCGCCAGTTGTTGTGCCACGCGGTTGGGAATGTAATTCAGCTCCGCCATCGCCGCTTCCACTTTTTCCCGCGTTTTCGCAGAAACGTGGCTGGCCTGGTTCACCACGCGGGAAACGGTCTGATAAGAGACACCGGCATACTCTGCGACATCGTATAACGTTACTGGTTTCACATTCACCACCCTGAATTGACTCTCTTCCGGGCGCTATCATGCCATACCGCGAAAGGTTTTGCGCCATTCGATGGTGTCCGGGATCTCGACGCTCTCCCTTATGCGACTCCTGCATTAGGAAGCAGCCCAGTAGTAGGTTGAGGCCGTTGAGCACCGCCGCCGCAAGGAATGGTGCATGCAAGGAGATGGCGCCCAACAGTCCCCCGGCCACGGGGCCTGCCACCATACCCACGCCGAAACAAGCGCTCATGAGCCCGAAGTGGCGAGCCCGATCTTCCCCATCGGTGATGTCGGCGATATAGGCGCCAGCAACCGCACCTGTGGCGCCGGTGATGCCGGCCACGATGCGTCCGGCGTAGAGGATCGAGATCTCGATCCCGCGAAATGCTGACAACATTGATAATCCAATCCTCCATCTCTTGGCGAAATTGTTCGAGATTTATCATAATATTTGATTTTTCCTCGCTTGTAGTAATTGGTAGTTGAAGCATTGGCAGCTGCGGTGTGCTATGTAATTTCTGTATAAAACATTGCTTGGTTTTTATTCAGATGTAAGCATCTGTTTTATTTCCTTGCAATTCAAGGGATTTATGAGTTACAATTACATGACTCACATTATTCGCGTTATTAGCGATATACCGTACAGCCAAAGATTTCATTATAGCACTACGCCCAGACCTAAGGAAATTTTAAGTTGAGATGACTAAAAAAATTTCATTCATTATTAACGGCCAGGTTGAAATCTTTCCCGAAAGTGATGATTTAGTGCAATCCATTAATTTTGGTGATAATAGTGTTTACCTGCCAATATTGAATGACTCTCATGTAAAAAACATTATTGATTGTAATGGAAATAACGAATTACGGTTGCATAACATTGTCAATTTTCTCTATACGGTAGGGCAAAGATGGAAAAATGAAGAATACTCAAGACGCAGGACATACATTCGTGACTTAAAAAAATATATGGGATATTCAGAAGAAATGGCTAAGCTAGAGGCCAATTGGATATCTATGATTTTATGTTCTAAAGGCGGCCTTTATGATGTTGTAGAAAATGAACTTGGTTCTCGCCATATCATGGATGAATGGCTACCTCAGGATGAAAGTTATGTTCGGGCTTTTCCGAAAGGTAAATCTGTACATCTGTTGGCAGGTAATGTTCCATTATCTGGGATCATGTCTATATTACGCGCAATTTTAACTAAGAATCAGTGTATTATAAAAACATCGTCAACCGATCCTTTTACCGCTAATGCATTAGCGTTAAGTTTTATTGATGTAGACCCTAATCATCCGATAACGCGCTCTTTATCTGTTATATATTGGCCCCACCAAGGTGATACATCACTCGCAAAAGAAATTATGCGACATGCGGATGTTATTGTCGCTTGGGGAGGGCCAGATGCGATTAATTGGGCGGTAGAGCATGCGCCATCTTATGCTGATGTGATTAAATTTGGTTCTAAAAAGAGTCTTTGCATTATCGATAATCCTGTTGATTTGACGTCCGCAGCGACAGGTGCGGCTCATGATGTTTGTTTTTACGATCAGCGAGCTTGTTTTTCTGCCCAAAACATATATTACATGGGAAATCATTATGAGGAATTTAAGTTAGCGTTGATAGAAAAACTTAATCTATATGCGCATATATTACCGAATGCCAAAAAAGATTTTGATGAAAAGGCGGCCTATTCTTTAGTTCAAAAAGAAAGCTTGTTTGCTGGATTAAAAGTAGAGGTGGATATTCATCAACGTTGGATGATTATTGAGTCAAATGCAGGTGTGGAATTTAATCAACCACTTGGCAGATGTGTGTACCTTCATCACGTCGATAATATTGAGCAAATATTGCCTTATGTTCAAAAAAATAAGACGCAAACCATATCTATTTTTCCTTGGGAGTCATCATTTAAATATCGAGATGCGTTAGCATTAAAAGGTGCGGAAAGGATTGTAGAAGCAGGAATGAATAACATATTTCGAGTTGGTGGATCTCATGACGGAATGAGACCGTTGCAACGATTAGTGACATATATTTCTCATGAAAGGCCATCTAACTATACGGCTAAGGATGTTGCGGTTGAAATAGAACAGACTCGATTCCTGGAAGAAGATAAGTTCCTTGTATTTGTCCCATAATAGGTAAAAGTATGGAAAATGAATCAAAATATAAAACCATCGACCACGTTATTTGTGTTGAAGGAAATAAAAAAATTCATGTTTGGGAAACGCTGCCAGAAGAAAACAGCCCAAAGAGAAAGAATGCCATTATTATTGCGTCTGGTTTTGCCCGCAGGATGGATCATTTTGCTGGTCTGGCGGAATATTTATCGCGGAATGGATTTCATGTGATCCGCTATGATTCGCTTCACCACGTTGGATTGAGTTCAGGGACAATTGATGAATTTACAATGTCTATAGGAAAGCAGAGCTTGTTAGCAGTGGTTGATTGGTTAACTACACGAAAAATAAATAACTTCGGTATGTTGGCTTCAAGCTTATCTGCGCGGATAGCTTATGCAAGCCTATCTGAAATCAATGCTTCGTTTTTAATCACCGCAGTCGGTGTTGTTAACTTAAGATATTCTCTTGAAAGAGCTTTAGGGTTTGATTATCTCAGTCTACCCATTAATGAATTGCCGGATAATCTAGATTTTGAAGGCCATAAATTGGGTGCTGAAGTCTTTGCGAGAGATTGTCTTGATTTTGGTTGGGAAGATTTAGCTTCTACAATTAATAACATGATGTATCTTGATATACCGTTTATTGCTTTTACTGCAAATAACGATAATTGGGTCAAGCAAGATGAAGTTATCACATTGTTATCAAATATTCGTAGTAATCGATGCAAGATATATTCTTTGTTAGGAAGTTCGCATGACTTGAGTGAAAATTTAGTGGTCCTGCGCAATTTTTATCAATCGGTTACGAAAGCCGCTATCGCGATGGATAATGATCATCTGGATATTGATGTTGATATTACTGAACCGTCATTTGAACATTTAACTATTGCGACAGTCAATGAACGCCGAATGAGAATTGAGATTGAAAATCAAGCAATTTCTCTGTCTTAAAATCTATTGAGATATTCTATCACTCAAATAGCAATATAAGGACTCTCTATGAAATTTGGAAACTTTTTGCTTACATACCAACCTCCCCAATTTTCTCAAACAGAGGTAATGAAACGTTTGGTTAAATTAGGTCGCATCTCTGAGGAGTGTGGTTTTGATACCGTATGGTTACTGGAGCATCATTTCACGGAGTTTGGTTTGCTTGGTAACCCTTATGTCGCTGCTGCATATTTACTTGGCGCGACTAAAAAATTGAATGTAGGAACTGCCGCTATTGTTCTTCCCACAGCCCATCCAGTACGCCAACTTGAAGATGTGAATTTATTGGATCAAATGTCAAAAGGACGATTTCGGTTTGGTATTTGCCGAGGGCTTTACAACAAGGACTTTCGCGTATTCGGCACAGATATGAATAACAGTCGCGCCTTAGCGGAATGCTGGTACGGGCTGATAAAGAATGGCATGACAGAGGGATATATGGAAGCTGATAATGAACATATCAAGTTCCATAAGGTAAAAGTAAACCCCGCGGCGTATAGCAGAGGTGGCGCACCGGTTTATGTGGTGGCTGAATCAGCTTCGACGACTGAGTGGGCTGCTCAATTTGGCCTACCGATGATATTAAGTTGGATTATAAATACTAACGAAAAGAAAGCACAACTTGAGCTTTATAATGAAGTGGCTCAAGAATATGGGCACGATATTCATAATATCGACCATTGCTTATCATATATAACATCTGTAGATCATGACTCAATTAAAGCGAAAGAGATTTGCCGGAAATTTCTGGGGCATTGGTATGATTCTTATGTGAATGCTACGACTATTTTTGATGATTCAGACCAAACAAGAGGTTATGATTTCAATAAAGGGCAGTGGCGTGACTTTGTATTAAAAGGACATAAAGATACTAATCGCCGTATTGATTACAGTTACGAAATCAATCCCGTGGGAACGCCGCAGGAATGTATTGACATAATTCAAAAAGACATTGATGCTACAGGAATATCAAATATTTGTTGTGGATTTGAAGCTAATGGAACAGTAGACGAAATTATTGCTTCCATGAAGCTCTTCCAGTCTGATGTCATGCCATTTCTTAAAGAAAAACAACGTTCGCTATTATATTAGCTAAGGAGAAAGAAATGAAATTTGGATTGTTCTTCCTTAACTTCATCAATTCAACAACTGTTCAAGAACAAAGTATAGTTCGCATGCAGGAAATAACGGAGTATGTTGATAAGTTGAATTTTGAACAGATTTTAGTGTATGAAAATCATTTTTCAGATAATGGTGTTGTCGGCGCTCCTCTGACTGTTTCTGGTTTTCTGCTCGGTTTAACAGAGAAAATTAAAATTGGTTCATTAAATCACATCATTACAACTCATCATCCTGTCGCCATAGCGGAGGAAGCTTGCTTATTGGATCAGTTAAGTGAAGGGAGATTTATTTTAGGGTTTAGTGATTGCGAAAAAAAAGATGAAATGCATTTTTTTAATCGCCCGGTTGAATATCAACAGCAACTATTTGAAGAGTGTTATGAAATCATTAACGATGCTTTAACAACAGGCTATTGTAATCCAGATAACGATTTTTATAGCTTCCCTAAAATATCTGTAAATCCCCATGCTTATACGCCAGGCGGACCTCGGAAATATGTAACAGCAACCAGTCATCATATTGTTGAGTGGGCGGCCAAAAAAGGTATTCCTCTCATCTTTAAGTGGGATGATTCTAATGATGTTAGATATGAATATGCTGAAAGATATAAAGCCGTTGCGGATAAATATGACGTTGACCTATCAGAGATAGACCATCAGTTAATGATATTAGTTAACTATAACGAAGATAGTAATAAAGCTAAACAAGAGACGCGTGCATTTATTAGTGATTATGTTCTTGAAATGCACCCTAATGAAAATTTCGAAAATAAACTTGAAGAAATAATTGCAGAAAACGCTGTCGGAAATTATACGGAGTGTATAACTGCGGCTAAGTTGGCAATTGAAAAGTGTGGTGCGAAAAGTGTATTGCTGTCCTTTGAACCAATGAATGATTTGATGAGCCAAAAAAATGTAATCAATATTGTTGATGATAATATTAAGAAGTACCACATGGAATATACCTAATAGATTTCGAGTTGCAGCGAGGCGGCAAGTGAACGAATCCCCAGGAGCATAGATAACTATGTGACTGGGGTGAGTGAAAGCAGCCAACAAAGCAGCAGCTTGAAAGATGAAGGGTATAAAAGAGTATGACAGCAGTGCTGCCATACTTTCTAATATTATCTTGAGGAGTAAAACAGGTATGACTTCATATGTTGATAAACAAGAAATTACAGCAAGCTCAGAAATTGATGATTTGATTTTTTCGAGCGATCCATTAGTGTGGTCTTACGACGAGCAGGAAAAAATCAGAAAGAAACTTGTGCTTGATGCATTTCGTAATCATTATAAACATTGTCGAGAATATCGTCACTACTGTCAGGCACACAAAGTAGATGACAATATTACGGAAATTGATGACATACCTGTATTCCCAACATCGGTTTTTAAGTTTACTCGCTTATTAACTTCTCAGGAAAACGAGATTGAAAGTTGGTTTACCAGTAGCGGCACGAATGGTTTAAAAAGTCAGGTGGCGCGTGACAGATTAAGTATTGAGAGACTCTTAGGCTCTGTGAGTTATGGCATGAAATATGTTGGTAGTTGGTTTGATCATCAAATAGAATTAGTCAATTTGGGACCAGATAGATTTAATGCTCATAATATTTGGTTTAAATATGTTATGAGTTTGGTGGAATTGTTATATCCTACGACATTTACCGTAACAGAAGAACGAATAGATTTTGTTAAAACATTGAATAGTCTTGAACGAATAAAAAATCAAGGGAAAGATCTTTGTCTTATTGGTTCGCCATACTTTATTTATTTACTCTGCCATTATATGAAAGATAAAAAAATCTCATTTTCTGGAGATAAAAGCCTTTATATCATAACCGGAGGCGGCTGGAAAAGTTACGAAAAAGAATCTCTGAAACGTGATGATTTCAATCATCTTTTATTTGATACTTTCAATCTCAGTGATATTAGTCAGATCCGAGATATATTTAATCAAGTTGAACTCAACACTTGTTTCTTTGAGGATGAAATGCAGCGTAAACATGTTCCGCCGTGGGTATATGCGCGAGCGCTTGATCCTGAAACGTTGAAACCTGTACCTGATGGAACGCCGGGGTTGATGAGTTATATGGATGCGTCAGCAACCAGTTATCCAGCATTTATTGTTACCGATGATGTCGGGATAATTAGCAGAGAATATGGTAAGTATCCCGGCGTGCTCGTTGAAATTTTACGTCGCGTCAATACGAGGACGCAGAAAGGGTGTGCTTTAAGCTTAACCGAAGCGTTTGATAGTTGACTCGAGCACCACCACCACCACCACTGAGATCCGGCTGCTAACAAAGCCCGAAAGGAAGCTGAGTTGGCTGCTGCCACCGCTGAGCAATAACTAGCATAACCCCTTGGGGCCTCTAAACGGGTCTTGAGGGGTTTTTTGCTGAAAGGAGGAACTATATCCGGATTGGGCGGCC


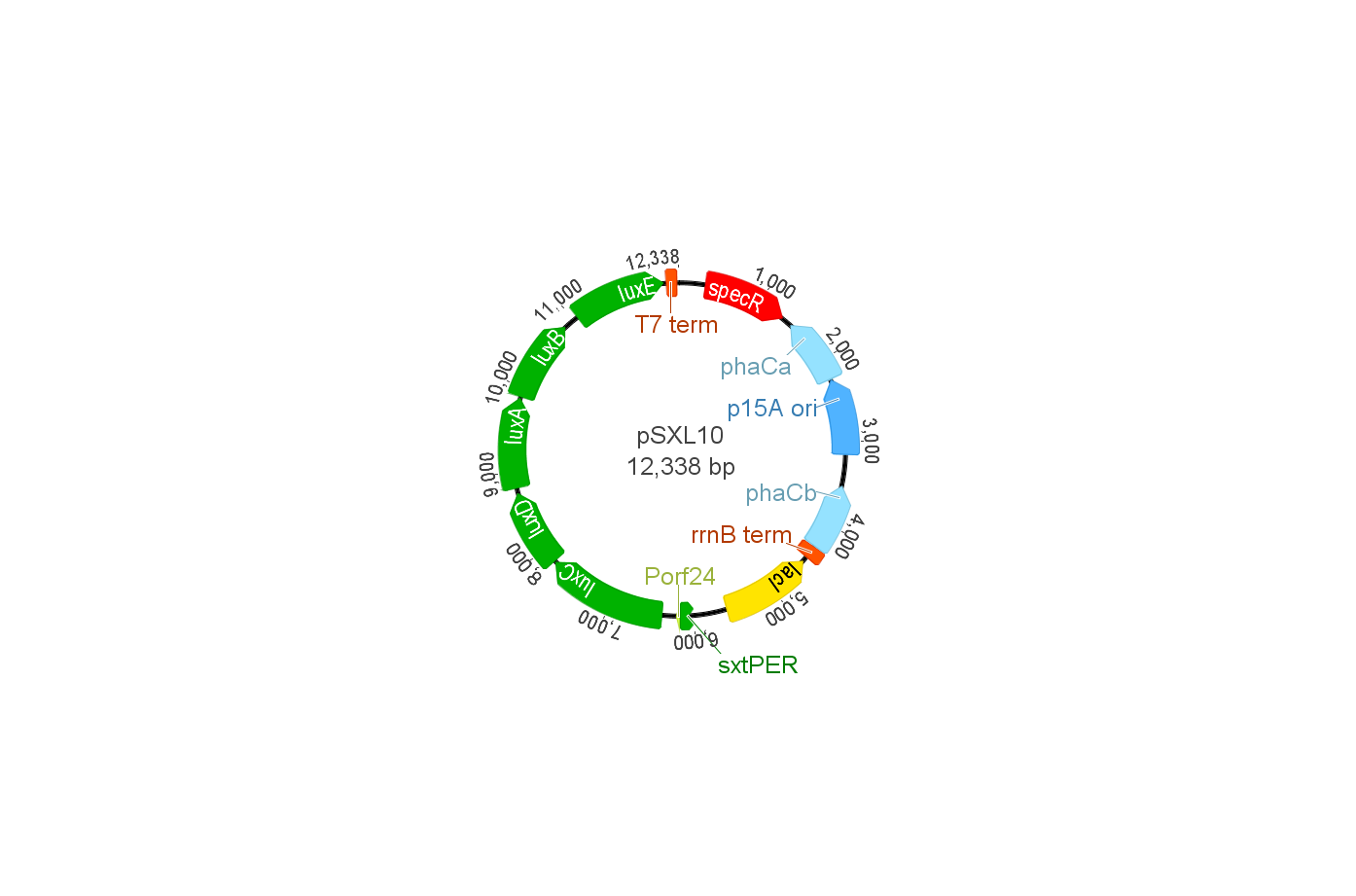


>pSXL10_nucl

GCGGAGATTAACTGTCAAAAAGGTGTAAAAAACCCTTAATTTTTAGCTAAGTAGACACTATTTTTATGTAACGAAAACTTTGTGAATTTATGTAATGTTTAGAGCGATCGCCCAAGGCTACAGTGCTCAACGGAGCCAAAACTACTGTCTACACTGGATTAGAAGACGCTAAATAGCCAGCCAGGACAGAAATGCCTCGACTTCGCTGCTACCCAAGGTTGCCGGGTGACGCACACCGTGGAAACGGATGAAGGCACGAACCCAGTGGACATAAGCCTGTTCGGTTCGTAAGCTGTAATGCAAGTAGCGTATGCGCTCACGCAACTGGTCCAGAACCTTGACCGAACGCAGCGGTGGTAACGGCGCAGTGGCGGTTTTCATGGCTTGTTATGACTGTTTTTTTGGGGTACAGTCTATGCCTCGGGCATCCAAGCAGCAAGCGCGTTACGCCGTGGGTCGATGTTTGATGTTATGGAGCAGCAACGATGTTACGCAGCAGGGCAGTCGCCCTAAAACAAAGTTAAACATTATGAGGGAAGCGGTGATCGCCGAAGTATCGACTCAACTATCAGAGGTAGTTGGCGTCATCGAGCGCCATCTCGAACCGACGTTGCTGGCCGTACATTTGTACGGCTCCGCAGTGGATGGCGGCCTGAAGCCACACAGTGATATTGATTTGCTGGTTACGGTGACCGTAAGGCTTGATGAAACAACGCGGCGAGCTTTGATCAACGACCTTTTGGAAACTTCGGCTTCCCCTGGAGAGAGCGAGATTCTCCGCGCTGTAGAAGTCACCATTGTTGTGCACGACGACATCATTCCGTGGCGTTATCCAGCTAAGCGCGAACTGCAATTTGGAGAATGGCAGCGCAATGACATTCTTGCTGGTATCTTCGAGCCAGCCACGATCGACATTGATCTGGCTATCTTGCTGACAAAAGCAAGAGAACATAGCGTTGCCTTGGTAGGTCCAGCGGCGGAGGAACTCTTTGATCCGGTTCCTGAACAGGATCTATTTGAGGCGCTAAATGAAACCTTAACGCTATGGAACTCGCCGCCCGACTGGGCTGGCGATGAGCGAAATGTAGTGCTTACGTTGTCCCGCATTTGGTACAGCGCAGTAACCGGCAAAATCGCGCCGAAGGATGTCGCTGCCGACTGGGCAATGGAGCGCCTGCCGGCCCAGTATCAGCCCGTCATACTTGAAGCTAGACAGGCTTATCTTGGACAAGAAGAAGATCGCTTGGCCTCGCGCGCAGATCAGTTGGAAGAATTTGTCCACTACGTGAAAGGCGAGATCACCAAGGTAGTCGGCAAATAACCTAATTGCCGGAATTACAGCAGTCTGAATAATCAAAAAGCGCCTCGAATGGGGCGCTTTTTGATGCTTGGATATTGATTGTAGACAACCTTTGGACTTGCGATCGCTAGGGATTATCTAGATTAGCCTGGGTTTGCTTCTGTTTCTCCCAATCGTTTTTTCAAACTTTTAACTTCTTTCCGCAACTGATAAATGGTTTGATGAATTTCATCCACCTCTGAGCGGGTAGGGAGGTTCAGCATTTTTAACCATGCTTCTAGAATCTCCTGTTGCTGAATGCGATAACGATTCAAGGCATTGATGAATTTGCCCCGTACTTTCAGATTTTTTTCCTCACAAAAAGCTTCTTCAAACACTTGGTCAGCGGCGATCGCCCACCGTTGTTGAAATTCCTTCCAGGTTTTAACGGGTTTATCTTCCTTAGCCCGGGCCAGTAAATCTTCCATCAAAGCAGCAAAGCCCCGGTATTGAATATCTGCTTCCAGTAATTGATAGTCTGCCATGGCCTGGGATAACTTAACCCAATCGTCAAAGGCCCGCAATAATTTGCCATTCATTTCCCGACTGGGCCCCAGCAAAGGCGATCGCATAAAACTGCTCAGGTTTTTGTTGTAGAGGGCATCGCCGTAGAGATTATTTAAATCTAACCAAGCATGGATGTCCCCGGTGGGTAATTTGCCCAGGGGGGCGACGCTACTCTGCCAGGTGGAGAGCCAGAGTTGGGAAAATCTTTGTACTTCCTTGATGTAACACTGCCATAAACCATCCATGTCCCCTTGGAGAGCTTGGGTGGTGGCGGTATATTGCTCAATTTGGGTTTGTAGCTGTTTTAGGTAGCCCTGCACTGCCCCTGGCGCCGAACCATTATCCAATTTAGGCCACAGGCTTTGCCAAGCTTCAAAAGAAAGCTTAAGTAACTCTGCTAGCGGAGTGTATACTGGCTTACTATGTTGGCACTGATGAGGGTGTCAGTGAAGTGCTTCATGTGGCAGGAGAAAAAAGGCTGCACCGGTGCGTCAGCAGAATATGTGATACAGGATATATTCCGCTTCCTCGCTCACTGACTCGCTACGCTCGGTCGTTCGACTGCGGCGAGCGGAAATGGCTTACGAACGGGGCGGAGATTTCCTGGAAGATGCCAGGAAGATACTTAACAGGGAAGTGAGAGGGCCGCGGCAAAGCCGTTTTTCCATAGGCTCCGCCCCCCTGACAAGCATCACGAAATCTGACGCTCAAATCAGTGGTGGCGAAACCCGACAGGACTATAAAGATACCAGGCGTTTCCCCCTGGCGGCTCCCTCGTGCGCTCTCCTGTTCCTGCCTTTCGGTTTACCGGTGTCATTCCGCTGTTATGGCCGCGTTTGTCTCATTCCACGCCTGACACTCAGTTCCGGGTAGGCAGTTCGCTCCAAGCTGGACTGTATGCACGAACCCCCCGTTCAGTCCGACCGCTGCGCCTTATCCGGTAACTATCGTCTTGAGTCCAACCCGGAAAGACATGCAAAAGCACCACTGGCAGCAGCCACTGGTAATTGATTTAGAGGAGTTAGTCTTGAAGTCATGCGCCGGTTAAGGCTAAACTGAAAGGACAAGTTTTGGTGACTGCGCTCCTCCAAGCCAGTTACCTCGGTTCAAAGAGTTGGTAGCTCAGAGAACCTTCGAAAAACCGCCCTGCAAGGCGGTTTTTTCGTTTTCAGAGCAAGAGATTACGCGCAGACCAAAACGATCTCAAGAAGATCATCTTATTAATCAGATAAAATATTTCTAGATTTCAGTGCAATTTATCTCTTCAAATGTAGCACCTGAAGTCAGCCCCATACGATATAAGTTGTGGGGTCTGACGCTCAGTGGAACGAAAACTCACGTTAAGGGATTTTGGTCATGAGATTATCGATCTTCCGCTGCATAACCCTGCTTCGGGGTCATTATAGCGATTTTTTCGGTATATCCATCCTTTTTCGCACGATATACAGGATTTTGCCAAAGGGTTCGTGTAGACTTTCCTTGGTGTATCCAACGGCGTCAGCCGGGCAGGATAGGTGAAGTAGGCCCACCCGCGAGCGGGTGTTCCTTCTTCACTGTCCCTTATTCGCACCTGGCGGTGCTCAACGGGAATCCTGCTCTGCGAGGCTGGCCGGCTACCGCCGGCGTAACAGATGAGGGCAAGCGGATGGCTGATGAAACCAAGCCAACCAGCGGCTACCATTACCACCCTGGTACAAAATATTCCCCAATTGGCTCGGATTTTAACCTACCACGTGGTTGCCGGGAAGTTTACCCAAGCTGATTTATGTCGCCTATCAACTGTTGATTCAGTAGAGGGCTCCCCGATCGCCATCGACTGTACGGAGGGGTTTGAAGTGAAAAATGCCACGGTAATCATCCCCGATATCGAAGCGGACAACGGCATTATCCATGTGATTGACAACGTGATTTTAATGGGATAGGCATGGTGTGCGACCTGTTCAAAACCTTTAGCCCAGGGGAGGATACCTTGGTATTCCAACTGCGGAGCTAATGGACTAGGATGGAAAATAGTGATCCCAGCGGTAATGCTGTTGGTAGGAGCCATCTGTGAACAACTTTTCCCCCAAATTTGTTACCGTCAACCAATCAGGCCTGGGCTGTGGCTTGACTGTCCTTGCCGTGGCCATGCTCCTCAGTGCCATTGGTCTGAAATGGGTTGTCAATGGCATTGCTCTGTTGATTTTGCTATTGTTTCTGGCACCAACGGTGGGCTTTTTGGTATTCCGCTGGTGGCTGAAGAGAAAATTGGTGGATGGGGAGTGTCCCGTTTGTCAGTATCACTTCACTGGTTTTGACGGTCAGCCATGCCGATGCCCCAGTTGCGGGGAAGAGTTGGAAGTGAGCAATGGACAATTTACCCGCCCCACTCCCCCTGGAACTATTGACGTACAAGCGGTGGATGTATCAGTTAAAGCACTAGATGAGTCCTGAGTGTTGCCCTAATGTATTCCAGCAAAATTTAAGGCCCAGTCTTTCGACTGAGCCTTTCGTTTTATTTGATGCCTGGCAGTTCCCTACTCTCGCATGGGGAGACCCCACACTACCATCGGCGCTACGGCGTTTCACTTCTGAGTTCGGCATGGGGTCAGGTGGGACCACCGCGCTACTGCCGCCAGGCACTCGATGCATGGTACCCGGTGCCTAATGAGTGAGCTAACTTACATTAATTGCGTTGCGCTCACTGCCCGCTTTCCAGTCGGGAAACCTGTCGTGCCAGCTGCATTAATGAATCGGCCAACGCGCGGGGAGAGGCGGTTTGCGTATTGGGCGCCAGGGTGGTTTTTCTTTTCACCAGTGAGACGGGCAACAGCTGATTGCCCTTCACCGCCTGGCCCTGAGAGAGTTGCAGCAAGCGGTCCACGCTGGTTTGCCCCAGCAGGCGAAAATCCTGTTTGATGGTGGTTAACGGCGGGATATAACATGAGCTGTCTTCGGTATCGTCGTATCCCACTACCGAGATATCCGCACCAACGCGCAGCCCGGACTCGGTAATGGCGCGCATTGCGCCCAGCGCCATCTGATCGTTGGCAACCAGCATCGCAGTGGGAACGATGCCCTCATTCAGCATTTGCATGGTTTGTTGAAAACCGGACATGGCACTCCAGTCGCCTTCCCGTTCCGCTATCGGCTGAATTTGATTGCGAGTGAGATATTTATGCCAGCCAGCCAGACGCAGACGCGCCGAGACAGAACTTAATGGGCCCGCTAACAGCGCGATTTGCTGGTGACCCAATGCGACCAGATGCTCCACGCCCAGTCGCGTACCGTCTTCATGGGAGAAAATAATACTGTTGATGGGTGTCTGGTCAGAGACATCAAGAAATAACGCCGGAACATTAGTGCAGGCAGCTTCCACAGCAATGGCATCCTGGTCATCCAGCGGATAGTTAATGATCAGCCCACTGACGCGTTGCGCGAGAAGATTGTGCACCGCCGCTTTACAGGCTTCGACGCCGCTTCGTTCTACCATCGACACCACCACGCTGGCACCCAGTTGATCGGCGCGAGATTTAATCGCCGCGACAATTTGCGACGGCGCGTGCAGGGCCAGACTGGAGGTGGCAACGCCAATCAGCAACGACTGTTTGCCCGCCAGTTGTTGTGCCACGCGGTTGGGAATGTAATTCAGCTCCGCCATCGCCGCTTCCACTTTTTCCCGCGTTTTCGCAGAAACGTGGCTGGCCTGGTTCACCACGCGGGAAACGGTCTGATAAGAGACACCGGCATACTCTGCGACATCGTATAACGTTACTGGTTTCACATTCACCACCCTGAATTGACTCTCTTCCGGGCGCTATCATGCCATACCGCGAAAGGTTTTGCGCCATTCGATGGTGTCCGGGATCTCGACGCTCTCCCTTATGCGACTCCTGCATTAGGAAGCAGCCCAGTAGTAGGTTGAGGCCGTTGAGCACCGCCGCCGCAAGGAATGGTGCATGCAAGGAGATGGCGCCCAACAGTCCCCCGGCCACGGGGCCTGCCACCATACCCACGCCGAAACAAGCGCTCATGAGCCCGAAGTGGCGAGCCCGATCTTCCCCATCGGTGATGTCGGCGATATAGGCGCCAGCAACCGCACCTGTGGCGCCGGTGATGCCGGCCACGATGCGTCCGGCGTAGAGGATCGAGATCTCGATCCCGCGAAATGTTAGTGATTAAACCTTTAGTAATTGGCAAGATTGAGCTATAGACTAGAGTTGCCAGAATAATCAACAGCACACCCAAAATATATCGGTTATTTTCTGGGGCAGATGTTGTCATCTCAACCTTATCCAAGCTATTTTGGAGTTCTGTTAAATCTTGGTTTACCATCTCAACTTAAAATTTCCTTAGGTCTGGGCGTAGTGCTATAATGAAATCTTTGGCTGTACGGTATATCGCTAATAACGCGAATAATGTGAGTCATGTAATTGTAACTCATAAATCCCTTGAATTGCAAGGAAATAAAACAGATGCTTACATCTGAATAAAAACCAAGCAATGTTTTATACAGAAATTACATAGCACACCGCAGCTGCCAATGACTAAAAAAATTTCATTCATTATTAACGGCCAGGTTGAAATCTTTCCCGAAAGTGATGATTTAGTGCAATCCATTAATTTTGGTGATAATAGTGTTTACCTGCCAATATTGAATGACTCTCATGTAAAAAACATTATTGATTGTAATGGAAATAACGAATTACGGTTGCATAACATTGTCAATTTTCTCTATACGGTAGGGCAAAGATGGAAAAATGAAGAATACTCAAGACGCAGGACATACATTCGTGACTTAAAAAAATATATGGGATATTCAGAAGAAATGGCTAAGCTAGAGGCCAATTGGATATCTATGATTTTATGTTCTAAAGGCGGCCTTTATGATGTTGTAGAAAATGAACTTGGTTCTCGCCATATCATGGATGAATGGCTACCTCAGGATGAAAGTTATGTTCGGGCTTTTCCGAAAGGTAAATCTGTACATCTGTTGGCAGGTAATGTTCCATTATCTGGGATCATGTCTATATTACGCGCAATTTTAACTAAGAATCAGTGTATTATAAAAACATCGTCAACCGATCCTTTTACCGCTAATGCATTAGCGTTAAGTTTTATTGATGTAGACCCTAATCATCCGATAACGCGCTCTTTATCTGTTATATATTGGCCCCACCAAGGTGATACATCACTCGCAAAAGAAATTATGCGACATGCGGATGTTATTGTCGCTTGGGGAGGGCCAGATGCGATTAATTGGGCGGTAGAGCATGCGCCATCTTATGCTGATGTGATTAAATTTGGTTCTAAAAAGAGTCTTTGCATTATCGATAATCCTGTTGATTTGACGTCCGCAGCGACAGGTGCGGCTCATGATGTTTGTTTTTACGATCAGCGAGCTTGTTTTTCTGCCCAAAACATATATTACATGGGAAATCATTATGAGGAATTTAAGTTAGCGTTGATAGAAAAACTTAATCTATATGCGCATATATTACCGAATGCCAAAAAAGATTTTGATGAAAAGGCGGCCTATTCTTTAGTTCAAAAAGAAAGCTTGTTTGCTGGATTAAAAGTAGAGGTGGATATTCATCAACGTTGGATGATTATTGAGTCAAATGCAGGTGTGGAATTTAATCAACCACTTGGCAGATGTGTGTACCTTCATCACGTCGATAATATTGAGCAAATATTGCCTTATGTTCAAAAAAATAAGACGCAAACCATATCTATTTTTCCTTGGGAGTCATCATTTAAATATCGAGATGCGTTAGCATTAAAAGGTGCGGAAAGGATTGTAGAAGCAGGAATGAATAACATATTTCGAGTTGGTGGATCTCATGACGGAATGAGACCGTTGCAACGATTAGTGACATATATTTCTCATGAAAGGCCATCTAACTATACGGCTAAGGATGTTGCGGTTGAAATAGAACAGACTCGATTCCTGGAAGAAGATAAGTTCCTTGTATTTGTCCCATAATAGGTAAAAGTATGGAAAATGAATCAAAATATAAAACCATCGACCACGTTATTTGTGTTGAAGGAAATAAAAAAATTCATGTTTGGGAAACGCTGCCAGAAGAAAACAGCCCAAAGAGAAAGAATGCCATTATTATTGCGTCTGGTTTTGCCCGCAGGATGGATCATTTTGCTGGTCTGGCGGAATATTTATCGCGGAATGGATTTCATGTGATCCGCTATGATTCGCTTCACCACGTTGGATTGAGTTCAGGGACAATTGATGAATTTACAATGTCTATAGGAAAGCAGAGCTTGTTAGCAGTGGTTGATTGGTTAACTACACGAAAAATAAATAACTTCGGTATGTTGGCTTCAAGCTTATCTGCGCGGATAGCTTATGCAAGCCTATCTGAAATCAATGCTTCGTTTTTAATCACCGCAGTCGGTGTTGTTAACTTAAGATATTCTCTTGAAAGAGCTTTAGGGTTTGATTATCTCAGTCTACCCATTAATGAATTGCCGGATAATCTAGATTTTGAAGGCCATAAATTGGGTGCTGAAGTCTTTGCGAGAGATTGTCTTGATTTTGGTTGGGAAGATTTAGCTTCTACAATTAATAACATGATGTATCTTGATATACCGTTTATTGCTTTTACTGCAAATAACGATAATTGGGTCAAGCAAGATGAAGTTATCACATTGTTATCAAATATTCGTAGTAATCGATGCAAGATATATTCTTTGTTAGGAAGTTCGCATGACTTGAGTGAAAATTTAGTGGTCCTGCGCAATTTTTATCAATCGGTTACGAAAGCCGCTATCGCGATGGATAATGATCATCTGGATATTGATGTTGATATTACTGAACCGTCATTTGAACATTTAACTATTGCGACAGTCAATGAACGCCGAATGAGAATTGAGATTGAAAATCAAGCAATTTCTCTGTCTTAAAATCTATTGAGATATTCTATCACTCAAATAGCAATATAAGGACTCTCTATGAAATTTGGAAACTTTTTGCTTACATACCAACCTCCCCAATTTTCTCAAACAGAGGTAATGAAACGTTTGGTTAAATTAGGTCGCATCTCTGAGGAGTGTGGTTTTGATACCGTATGGTTACTGGAGCATCATTTCACGGAGTTTGGTTTGCTTGGTAACCCTTATGTCGCTGCTGCATATTTACTTGGCGCGACTAAAAAATTGAATGTAGGAACTGCCGCTATTGTTCTTCCCACAGCCCATCCAGTACGCCAACTTGAAGATGTGAATTTATTGGATCAAATGTCAAAAGGACGATTTCGGTTTGGTATTTGCCGAGGGCTTTACAACAAGGACTTTCGCGTATTCGGCACAGATATGAATAACAGTCGCGCCTTAGCGGAATGCTGGTACGGGCTGATAAAGAATGGCATGACAGAGGGATATATGGAAGCTGATAATGAACATATCAAGTTCCATAAGGTAAAAGTAAACCCCGCGGCGTATAGCAGAGGTGGCGCACCGGTTTATGTGGTGGCTGAATCAGCTTCGACGACTGAGTGGGCTGCTCAATTTGGCCTACCGATGATATTAAGTTGGATTATAAATACTAACGAAAAGAAAGCACAACTTGAGCTTTATAATGAAGTGGCTCAAGAATATGGGCACGATATTCATAATATCGACCATTGCTTATCATATATAACATCTGTAGATCATGACTCAATTAAAGCGAAAGAGATTTGCCGGAAATTTCTGGGGCATTGGTATGATTCTTATGTGAATGCTACGACTATTTTTGATGATTCAGACCAAACAAGAGGTTATGATTTCAATAAAGGGCAGTGGCGTGACTTTGTATTAAAAGGACATAAAGATACTAATCGCCGTATTGATTACAGTTACGAAATCAATCCCGTGGGAACGCCGCAGGAATGTATTGACATAATTCAAAAAGACATTGATGCTACAGGAATATCAAATATTTGTTGTGGATTTGAAGCTAATGGAACAGTAGACGAAATTATTGCTTCCATGAAGCTCTTCCAGTCTGATGTCATGCCATTTCTTAAAGAAAAACAACGTTCGCTATTATATTAGCTAAGGAGAAAGAAATGAAATTTGGATTGTTCTTCCTTAACTTCATCAATTCAACAACTGTTCAAGAACAAAGTATAGTTCGCATGCAGGAAATAACGGAGTATGTTGATAAGTTGAATTTTGAACAGATTTTAGTGTATGAAAATCATTTTTCAGATAATGGTGTTGTCGGCGCTCCTCTGACTGTTTCTGGTTTTCTGCTCGGTTTAACAGAGAAAATTAAAATTGGTTCATTAAATCACATCATTACAACTCATCATCCTGTCGCCATAGCGGAGGAAGCTTGCTTATTGGATCAGTTAAGTGAAGGGAGATTTATTTTAGGGTTTAGTGATTGCGAAAAAAAAGATGAAATGCATTTTTTTAATCGCCCGGTTGAATATCAACAGCAACTATTTGAAGAGTGTTATGAAATCATTAACGATGCTTTAACAACAGGCTATTGTAATCCAGATAACGATTTTTATAGCTTCCCTAAAATATCTGTAAATCCCCATGCTTATACGCCAGGCGGACCTCGGAAATATGTAACAGCAACCAGTCATCATATTGTTGAGTGGGCGGCCAAAAAAGGTATTCCTCTCATCTTTAAGTGGGATGATTCTAATGATGTTAGATATGAATATGCTGAAAGATATAAAGCCGTTGCGGATAAATATGACGTTGACCTATCAGAGATAGACCATCAGTTAATGATATTAGTTAACTATAACGAAGATAGTAATAAAGCTAAACAAGAGACGCGTGCATTTATTAGTGATTATGTTCTTGAAATGCACCCTAATGAAAATTTCGAAAATAAACTTGAAGAAATAATTGCAGAAAACGCTGTCGGAAATTATACGGAGTGTATAACTGCGGCTAAGTTGGCAATTGAAAAGTGTGGTGCGAAAAGTGTATTGCTGTCCTTTGAACCAATGAATGATTTGATGAGCCAAAAAAATGTAATCAATATTGTTGATGATAATATTAAGAAGTACCACATGGAATATACCTAATAGATTTCGAGTTGCAGCGAGGCGGCAAGTGAACGAATCCCCAGGAGCATAGATAACTATGTGACTGGGGTGAGTGAAAGCAGCCAACAAAGCAGCAGCTTGAAAGATGAAGGGTATAAAAGAGTATGACAGCAGTGCTGCCATACTTTCTAATATTATCTTGAGGAGTAAAACAGGTATGACTTCATATGTTGATAAACAAGAAATTACAGCAAGCTCAGAAATTGATGATTTGATTTTTTCGAGCGATCCATTAGTGTGGTCTTACGACGAGCAGGAAAAAATCAGAAAGAAACTTGTGCTTGATGCATTTCGTAATCATTATAAACATTGTCGAGAATATCGTCACTACTGTCAGGCACACAAAGTAGATGACAATATTACGGAAATTGATGACATACCTGTATTCCCAACATCGGTTTTTAAGTTTACTCGCTTATTAACTTCTCAGGAAAACGAGATTGAAAGTTGGTTTACCAGTAGCGGCACGAATGGTTTAAAAAGTCAGGTGGCGCGTGACAGATTAAGTATTGAGAGACTCTTAGGCTCTGTGAGTTATGGCATGAAATATGTTGGTAGTTGGTTTGATCATCAAATAGAATTAGTCAATTTGGGACCAGATAGATTTAATGCTCATAATATTTGGTTTAAATATGTTATGAGTTTGGTGGAATTGTTATATCCTACGACATTTACCGTAACAGAAGAACGAATAGATTTTGTTAAAACATTGAATAGTCTTGAACGAATAAAAAATCAAGGGAAAGATCTTTGTCTTATTGGTTCGCCATACTTTATTTATTTACTCTGCCATTATATGAAAGATAAAAAAATCTCATTTTCTGGAGATAAAAGCCTTTATATCATAACCGGAGGCGGCTGGAAAAGTTACGAAAAAGAATCTCTGAAACGTGATGATTTCAATCATCTTTTATTTGATACTTTCAATCTCAGTGATATTAGTCAGATCCGAGATATATTTAATCAAGTTGAACTCAACACTTGTTTCTTTGAGGATGAAATGCAGCGTAAACATGTTCCGCCGTGGGTATATGCGCGAGCGCTTGATCCTGAAACGTTGAAACCTGTACCTGATGGAACGCCGGGGTTGATGAGTTATATGGATGCGTCAGCAACCAGTTATCCAGCATTTATTGTTACCGATGATGTCGGGATAATTAGCAGAGAATATGGTAAGTATCCCGGCGTGCTCGTTGAAATTTTACGTCGCGTCAATACGAGGACGCAGAAAGGGTGTGCTTTAAGCTTAACCGAAGCGTTTGATAGTTGACTCGAGCACCACCACCACCACCACTGAGATCCGGCTGCTAACAAAGCCCGAAAGGAAGCTGAGTTGGCTGCTGCCACCGCTGAGCAATAACTAGCATAACCCCTTGGGGCCTCTAAACGGGTCTTGAGGGGTTTTTTGCTGAAAGGAGGAACTATATCCGGATTGGGCGGCC


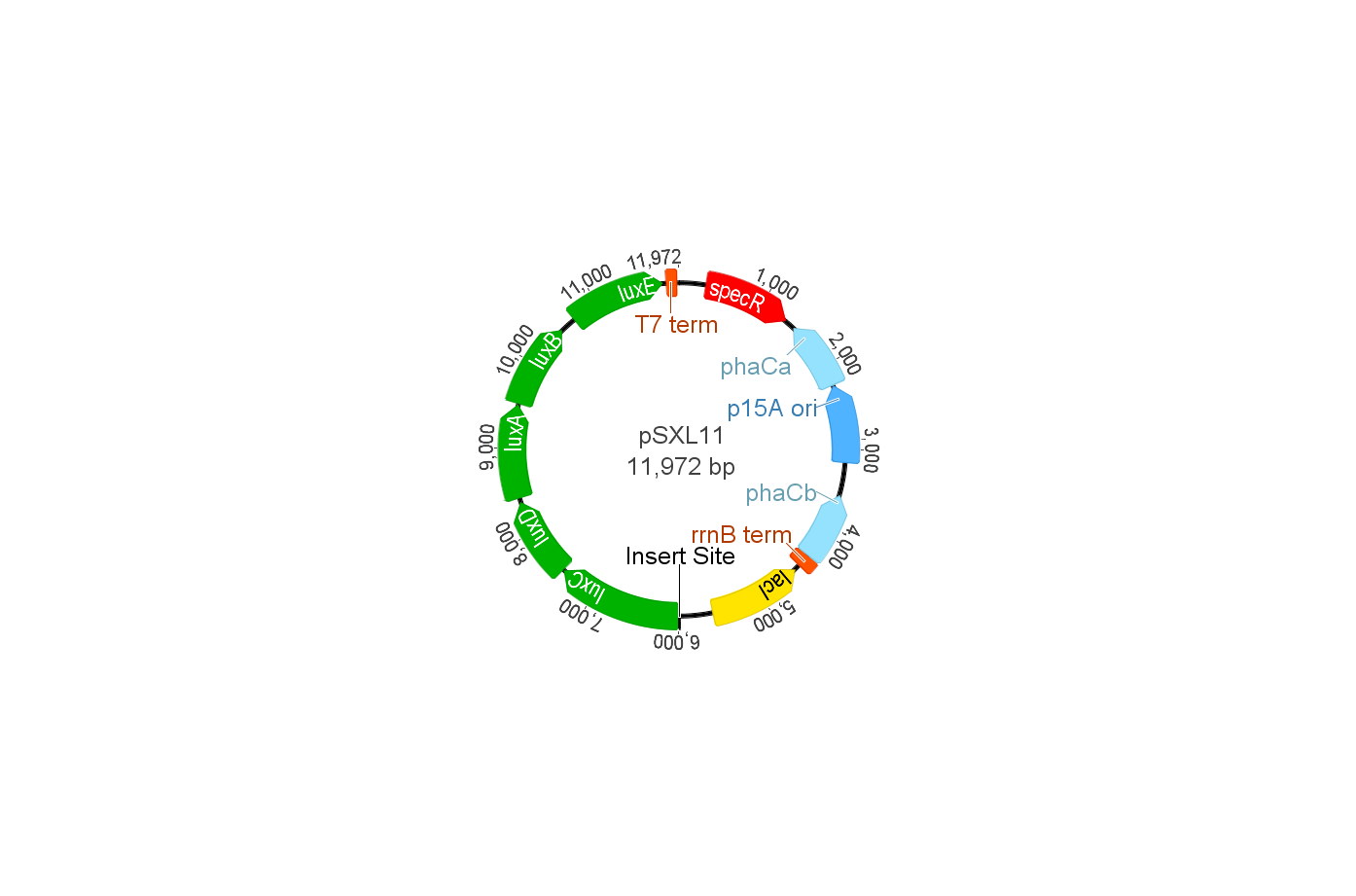


>pSXL11_nucl

GCGGAGATTAACTGTCAAAAAGGTGTAAAAAACCCTTAATTTTTAGCTAAGTAGACACTATTTTTATGTAACGAAAACTTTGTGAATTTATGTAATGTTTAGAGCGATCGCCCAAGGCTACAGTGCTCAACGGAGCCAAAACTACTGTCTACACTGGATTAGAAGACGCTAAATAGCCAGCCAGGACAGAAATGCCTCGACTTCGCTGCTACCCAAGGTTGCCGGGTGACGCACACCGTGGAAACGGATGAAGGCACGAACCCAGTGGACATAAGCCTGTTCGGTTCGTAAGCTGTAATGCAAGTAGCGTATGCGCTCACGCAACTGGTCCAGAACCTTGACCGAACGCAGCGGTGGTAACGGCGCAGTGGCGGTTTTCATGGCTTGTTATGACTGTTTTTTTGGGGTACAGTCTATGCCTCGGGCATCCAAGCAGCAAGCGCGTTACGCCGTGGGTCGATGTTTGATGTTATGGAGCAGCAACGATGTTACGCAGCAGGGCAGTCGCCCTAAAACAAAGTTAAACATTATGAGGGAAGCGGTGATCGCCGAAGTATCGACTCAACTATCAGAGGTAGTTGGCGTCATCGAGCGCCATCTCGAACCGACGTTGCTGGCCGTACATTTGTACGGCTCCGCAGTGGATGGCGGCCTGAAGCCACACAGTGATATTGATTTGCTGGTTACGGTGACCGTAAGGCTTGATGAAACAACGCGGCGAGCTTTGATCAACGACCTTTTGGAAACTTCGGCTTCCCCTGGAGAGAGCGAGATTCTCCGCGCTGTAGAAGTCACCATTGTTGTGCACGACGACATCATTCCGTGGCGTTATCCAGCTAAGCGCGAACTGCAATTTGGAGAATGGCAGCGCAATGACATTCTTGCTGGTATCTTCGAGCCAGCCACGATCGACATTGATCTGGCTATCTTGCTGACAAAAGCAAGAGAACATAGCGTTGCCTTGGTAGGTCCAGCGGCGGAGGAACTCTTTGATCCGGTTCCTGAACAGGATCTATTTGAGGCGCTAAATGAAACCTTAACGCTATGGAACTCGCCGCCCGACTGGGCTGGCGATGAGCGAAATGTAGTGCTTACGTTGTCCCGCATTTGGTACAGCGCAGTAACCGGCAAAATCGCGCCGAAGGATGTCGCTGCCGACTGGGCAATGGAGCGCCTGCCGGCCCAGTATCAGCCCGTCATACTTGAAGCTAGACAGGCTTATCTTGGACAAGAAGAAGATCGCTTGGCCTCGCGCGCAGATCAGTTGGAAGAATTTGTCCACTACGTGAAAGGCGAGATCACCAAGGTAGTCGGCAAATAACCTAATTGCCGGAATTACAGCAGTCTGAATAATCAAAAAGCGCCTCGAATGGGGCGCTTTTTGATGCTTGGATATTGATTGTAGACAACCTTTGGACTTGCGATCGCTAGGGATTATCTAGATTAGCCTGGGTTTGCTTCTGTTTCTCCCAATCGTTTTTTCAAACTTTTAACTTCTTTCCGCAACTGATAAATGGTTTGATGAATTTCATCCACCTCTGAGCGGGTAGGGAGGTTCAGCATTTTTAACCATGCTTCTAGAATCTCCTGTTGCTGAATGCGATAACGATTCAAGGCATTGATGAATTTGCCCCGTACTTTCAGATTTTTTTCCTCACAAAAAGCTTCTTCAAACACTTGGTCAGCGGCGATCGCCCACCGTTGTTGAAATTCCTTCCAGGTTTTAACGGGTTTATCTTCCTTAGCCCGGGCCAGTAAATCTTCCATCAAAGCAGCAAAGCCCCGGTATTGAATATCTGCTTCCAGTAATTGATAGTCTGCCATGGCCTGGGATAACTTAACCCAATCGTCAAAGGCCCGCAATAATTTGCCATTCATTTCCCGACTGGGCCCCAGCAAAGGCGATCGCATAAAACTGCTCAGGTTTTTGTTGTAGAGGGCATCGCCGTAGAGATTATTTAAATCTAACCAAGCATGGATGTCCCCGGTGGGTAATTTGCCCAGGGGGGCGACGCTACTCTGCCAGGTGGAGAGCCAGAGTTGGGAAAATCTTTGTACTTCCTTGATGTAACACTGCCATAAACCATCCATGTCCCCTTGGAGAGCTTGGGTGGTGGCGGTATATTGCTCAATTTGGGTTTGTAGCTGTTTTAGGTAGCCCTGCACTGCCCCTGGCGCCGAACCATTATCCAATTTAGGCCACAGGCTTTGCCAAGCTTCAAAAGAAAGCTTAAGTAACTCTGCTAGCGGAGTGTATACTGGCTTACTATGTTGGCACTGATGAGGGTGTCAGTGAAGTGCTTCATGTGGCAGGAGAAAAAAGGCTGCACCGGTGCGTCAGCAGAATATGTGATACAGGATATATTCCGCTTCCTCGCTCACTGACTCGCTACGCTCGGTCGTTCGACTGCGGCGAGCGGAAATGGCTTACGAACGGGGCGGAGATTTCCTGGAAGATGCCAGGAAGATACTTAACAGGGAAGTGAGAGGGCCGCGGCAAAGCCGTTTTTCCATAGGCTCCGCCCCCCTGACAAGCATCACGAAATCTGACGCTCAAATCAGTGGTGGCGAAACCCGACAGGACTATAAAGATACCAGGCGTTTCCCCCTGGCGGCTCCCTCGTGCGCTCTCCTGTTCCTGCCTTTCGGTTTACCGGTGTCATTCCGCTGTTATGGCCGCGTTTGTCTCATTCCACGCCTGACACTCAGTTCCGGGTAGGCAGTTCGCTCCAAGCTGGACTGTATGCACGAACCCCCCGTTCAGTCCGACCGCTGCGCCTTATCCGGTAACTATCGTCTTGAGTCCAACCCGGAAAGACATGCAAAAGCACCACTGGCAGCAGCCACTGGTAATTGATTTAGAGGAGTTAGTCTTGAAGTCATGCGCCGGTTAAGGCTAAACTGAAAGGACAAGTTTTGGTGACTGCGCTCCTCCAAGCCAGTTACCTCGGTTCAAAGAGTTGGTAGCTCAGAGAACCTTCGAAAAACCGCCCTGCAAGGCGGTTTTTTCGTTTTCAGAGCAAGAGATTACGCGCAGACCAAAACGATCTCAAGAAGATCATCTTATTAATCAGATAAAATATTTCTAGATTTCAGTGCAATTTATCTCTTCAAATGTAGCACCTGAAGTCAGCCCCATACGATATAAGTTGTGGGGTCTGACGCTCAGTGGAACGAAAACTCACGTTAAGGGATTTTGGTCATGAGATTATCGATCTTCCGCTGCATAACCCTGCTTCGGGGTCATTATAGCGATTTTTTCGGTATATCCATCCTTTTTCGCACGATATACAGGATTTTGCCAAAGGGTTCGTGTAGACTTTCCTTGGTGTATCCAACGGCGTCAGCCGGGCAGGATAGGTGAAGTAGGCCCACCCGCGAGCGGGTGTTCCTTCTTCACTGTCCCTTATTCGCACCTGGCGGTGCTCAACGGGAATCCTGCTCTGCGAGGCTGGCCGGCTACCGCCGGCGTAACAGATGAGGGCAAGCGGATGGCTGATGAAACCAAGCCAACCAGCGGCTACCATTACCACCCTGGTACAAAATATTCCCCAATTGGCTCGGATTTTAACCTACCACGTGGTTGCCGGGAAGTTTACCCAAGCTGATTTATGTCGCCTATCAACTGTTGATTCAGTAGAGGGCTCCCCGATCGCCATCGACTGTACGGAGGGGTTTGAAGTGAAAAATGCCACGGTAATCATCCCCGATATCGAAGCGGACAACGGCATTATCCATGTGATTGACAACGTGATTTTAATGGGATAGGCATGGTGTGCGACCTGTTCAAAACCTTTAGCCCAGGGGAGGATACCTTGGTATTCCAACTGCGGAGCTAATGGACTAGGATGGAAAATAGTGATCCCAGCGGTAATGCTGTTGGTAGGAGCCATCTGTGAACAACTTTTCCCCCAAATTTGTTACCGTCAACCAATCAGGCCTGGGCTGTGGCTTGACTGTCCTTGCCGTGGCCATGCTCCTCAGTGCCATTGGTCTGAAATGGGTTGTCAATGGCATTGCTCTGTTGATTTTGCTATTGTTTCTGGCACCAACGGTGGGCTTTTTGGTATTCCGCTGGTGGCTGAAGAGAAAATTGGTGGATGGGGAGTGTCCCGTTTGTCAGTATCACTTCACTGGTTTTGACGGTCAGCCATGCCGATGCCCCAGTTGCGGGGAAGAGTTGGAAGTGAGCAATGGACAATTTACCCGCCCCACTCCCCCTGGAACTATTGACGTACAAGCGGTGGATGTATCAGTTAAAGCACTAGATGAGTCCTGAGTGTTGCCCTAATGTATTCCAGCAAAATTTAAGGCCCAGTCTTTCGACTGAGCCTTTCGTTTTATTTGATGCCTGGCAGTTCCCTACTCTCGCATGGGGAGACCCCACACTACCATCGGCGCTACGGCGTTTCACTTCTGAGTTCGGCATGGGGTCAGGTGGGACCACCGCGCTACTGCCGCCAGGCACTCGATGCATGGTACCCGGTGCCTAATGAGTGAGCTAACTTACATTAATTGCGTTGCGCTCACTGCCCGCTTTCCAGTCGGGAAACCTGTCGTGCCAGCTGCATTAATGAATCGGCCAACGCGCGGGGAGAGGCGGTTTGCGTATTGGGCGCCAGGGTGGTTTTTCTTTTCACCAGTGAGACGGGCAACAGCTGATTGCCCTTCACCGCCTGGCCCTGAGAGAGTTGCAGCAAGCGGTCCACGCTGGTTTGCCCCAGCAGGCGAAAATCCTGTTTGATGGTGGTTAACGGCGGGATATAACATGAGCTGTCTTCGGTATCGTCGTATCCCACTACCGAGATATCCGCACCAACGCGCAGCCCGGACTCGGTAATGGCGCGCATTGCGCCCAGCGCCATCTGATCGTTGGCAACCAGCATCGCAGTGGGAACGATGCCCTCATTCAGCATTTGCATGGTTTGTTGAAAACCGGACATGGCACTCCAGTCGCCTTCCCGTTCCGCTATCGGCTGAATTTGATTGCGAGTGAGATATTTATGCCAGCCAGCCAGACGCAGACGCGCCGAGACAGAACTTAATGGGCCCGCTAACAGCGCGATTTGCTGGTGACCCAATGCGACCAGATGCTCCACGCCCAGTCGCGTACCGTCTTCATGGGAGAAAATAATACTGTTGATGGGTGTCTGGTCAGAGACATCAAGAAATAACGCCGGAACATTAGTGCAGGCAGCTTCCACAGCAATGGCATCCTGGTCATCCAGCGGATAGTTAATGATCAGCCCACTGACGCGTTGCGCGAGAAGATTGTGCACCGCCGCTTTACAGGCTTCGACGCCGCTTCGTTCTACCATCGACACCACCACGCTGGCACCCAGTTGATCGGCGCGAGATTTAATCGCCGCGACAATTTGCGACGGCGCGTGCAGGGCCAGACTGGAGGTGGCAACGCCAATCAGCAACGACTGTTTGCCCGCCAGTTGTTGTGCCACGCGGTTGGGAATGTAATTCAGCTCCGCCATCGCCGCTTCCACTTTTTCCCGCGTTTTCGCAGAAACGTGGCTGGCCTGGTTCACCACGCGGGAAACGGTCTGATAAGAGACACCGGCATACTCTGCGACATCGTATAACGTTACTGGTTTCACATTCACCACCCTGAATTGACTCTCTTCCGGGCGCTATCATGCCATACCGCGAAAGGTTTTGCGCCATTCGATGGTGTCCGGGATCTCGACGCTCTCCCTTATGCGACTCCTGCATTAGGAAGCAGCCCAGTAGTAGGTTGAGGCCGTTGAGCACCGCCGCCGCAAGGAATGGTGCATGCAAGGAGATGGCGCCCAACAGTCCCCCGGCCACGGGGCCTGCCACCATACCCACGCCGAAACAAGCGCTCATGAGCCCGAAGTGGCGAGCCCGATCTTCCCCATCGGTGATGTCGGCGATATAGGCGCCAGCAACCGCACCTGTGGCGCCGGTGATGCCGGCCACGATGCGTCCGGCGTAGAGGATCGAGATCTCGATCCCGCGAAATATGACTAAAAAAATTTCATTCATTATTAACGGCCAGGTTGAAATCTTTCCCGAAAGTGATGATTTAGTGCAATCCATTAATTTTGGTGATAATAGTGTTTACCTGCCAATATTGAATGACTCTCATGTAAAAAACATTATTGATTGTAATGGAAATAACGAATTACGGTTGCATAACATTGTCAATTTTCTCTATACGGTAGGGCAAAGATGGAAAAATGAAGAATACTCAAGACGCAGGACATACATTCGTGACTTAAAAAAATATATGGGATATTCAGAAGAAATGGCTAAGCTAGAGGCCAATTGGATATCTATGATTTTATGTTCTAAAGGCGGCCTTTATGATGTTGTAGAAAATGAACTTGGTTCTCGCCATATCATGGATGAATGGCTACCTCAGGATGAAAGTTATGTTCGGGCTTTTCCGAAAGGTAAATCTGTACATCTGTTGGCAGGTAATGTTCCATTATCTGGGATCATGTCTATATTACGCGCAATTTTAACTAAGAATCAGTGTATTATAAAAACATCGTCAACCGATCCTTTTACCGCTAATGCATTAGCGTTAAGTTTTATTGATGTAGACCCTAATCATCCGATAACGCGCTCTTTATCTGTTATATATTGGCCCCACCAAGGTGATACATCACTCGCAAAAGAAATTATGCGACATGCGGATGTTATTGTCGCTTGGGGAGGGCCAGATGCGATTAATTGGGCGGTAGAGCATGCGCCATCTTATGCTGATGTGATTAAATTTGGTTCTAAAAAGAGTCTTTGCATTATCGATAATCCTGTTGATTTGACGTCCGCAGCGACAGGTGCGGCTCATGATGTTTGTTTTTACGATCAGCGAGCTTGTTTTTCTGCCCAAAACATATATTACATGGGAAATCATTATGAGGAATTTAAGTTAGCGTTGATAGAAAAACTTAATCTATATGCGCATATATTACCGAATGCCAAAAAAGATTTTGATGAAAAGGCGGCCTATTCTTTAGTTCAAAAAGAAAGCTTGTTTGCTGGATTAAAAGTAGAGGTGGATATTCATCAACGTTGGATGATTATTGAGTCAAATGCAGGTGTGGAATTTAATCAACCACTTGGCAGATGTGTGTACCTTCATCACGTCGATAATATTGAGCAAATATTGCCTTATGTTCAAAAAAATAAGACGCAAACCATATCTATTTTTCCTTGGGAGTCATCATTTAAATATCGAGATGCGTTAGCATTAAAAGGTGCGGAAAGGATTGTAGAAGCAGGAATGAATAACATATTTCGAGTTGGTGGATCTCATGACGGAATGAGACCGTTGCAACGATTAGTGACATATATTTCTCATGAAAGGCCATCTAACTATACGGCTAAGGATGTTGCGGTTGAAATAGAACAGACTCGATTCCTGGAAGAAGATAAGTTCCTTGTATTTGTCCCATAATAGGTAAAAGTATGGAAAATGAATCAAAATATAAAACCATCGACCACGTTATTTGTGTTGAAGGAAATAAAAAAATTCATGTTTGGGAAACGCTGCCAGAAGAAAACAGCCCAAAGAGAAAGAATGCCATTATTATTGCGTCTGGTTTTGCCCGCAGGATGGATCATTTTGCTGGTCTGGCGGAATATTTATCGCGGAATGGATTTCATGTGATCCGCTATGATTCGCTTCACCACGTTGGATTGAGTTCAGGGACAATTGATGAATTTACAATGTCTATAGGAAAGCAGAGCTTGTTAGCAGTGGTTGATTGGTTAACTACACGAAAAATAAATAACTTCGGTATGTTGGCTTCAAGCTTATCTGCGCGGATAGCTTATGCAAGCCTATCTGAAATCAATGCTTCGTTTTTAATCACCGCAGTCGGTGTTGTTAACTTAAGATATTCTCTTGAAAGAGCTTTAGGGTTTGATTATCTCAGTCTACCCATTAATGAATTGCCGGATAATCTAGATTTTGAAGGCCATAAATTGGGTGCTGAAGTCTTTGCGAGAGATTGTCTTGATTTTGGTTGGGAAGATTTAGCTTCTACAATTAATAACATGATGTATCTTGATATACCGTTTATTGCTTTTACTGCAAATAACGATAATTGGGTCAAGCAAGATGAAGTTATCACATTGTTATCAAATATTCGTAGTAATCGATGCAAGATATATTCTTTGTTAGGAAGTTCGCATGACTTGAGTGAAAATTTAGTGGTCCTGCGCAATTTTTATCAATCGGTTACGAAAGCCGCTATCGCGATGGATAATGATCATCTGGATATTGATGTTGATATTACTGAACCGTCATTTGAACATTTAACTATTGCGACAGTCAATGAACGCCGAATGAGAATTGAGATTGAAAATCAAGCAATTTCTCTGTCTTAAAATCTATTGAGATATTCTATCACTCAAATAGCAATATAAGGACTCTCTATGAAATTTGGAAACTTTTTGCTTACATACCAACCTCCCCAATTTTCTCAAACAGAGGTAATGAAACGTTTGGTTAAATTAGGTCGCATCTCTGAGGAGTGTGGTTTTGATACCGTATGGTTACTGGAGCATCATTTCACGGAGTTTGGTTTGCTTGGTAACCCTTATGTCGCTGCTGCATATTTACTTGGCGCGACTAAAAAATTGAATGTAGGAACTGCCGCTATTGTTCTTCCCACAGCCCATCCAGTACGCCAACTTGAAGATGTGAATTTATTGGATCAAATGTCAAAAGGACGATTTCGGTTTGGTATTTGCCGAGGGCTTTACAACAAGGACTTTCGCGTATTCGGCACAGATATGAATAACAGTCGCGCCTTAGCGGAATGCTGGTACGGGCTGATAAAGAATGGCATGACAGAGGGATATATGGAAGCTGATAATGAACATATCAAGTTCCATAAGGTAAAAGTAAACCCCGCGGCGTATAGCAGAGGTGGCGCACCGGTTTATGTGGTGGCTGAATCAGCTTCGACGACTGAGTGGGCTGCTCAATTTGGCCTACCGATGATATTAAGTTGGATTATAAATACTAACGAAAAGAAAGCACAACTTGAGCTTTATAATGAAGTGGCTCAAGAATATGGGCACGATATTCATAATATCGACCATTGCTTATCATATATAACATCTGTAGATCATGACTCAATTAAAGCGAAAGAGATTTGCCGGAAATTTCTGGGGCATTGGTATGATTCTTATGTGAATGCTACGACTATTTTTGATGATTCAGACCAAACAAGAGGTTATGATTTCAATAAAGGGCAGTGGCGTGACTTTGTATTAAAAGGACATAAAGATACTAATCGCCGTATTGATTACAGTTACGAAATCAATCCCGTGGGAACGCCGCAGGAATGTATTGACATAATTCAAAAAGACATTGATGCTACAGGAATATCAAATATTTGTTGTGGATTTGAAGCTAATGGAACAGTAGACGAAATTATTGCTTCCATGAAGCTCTTCCAGTCTGATGTCATGCCATTTCTTAAAGAAAAACAACGTTCGCTATTATATTAGCTAAGGAGAAAGAAATGAAATTTGGATTGTTCTTCCTTAACTTCATCAATTCAACAACTGTTCAAGAACAAAGTATAGTTCGCATGCAGGAAATAACGGAGTATGTTGATAAGTTGAATTTTGAACAGATTTTAGTGTATGAAAATCATTTTTCAGATAATGGTGTTGTCGGCGCTCCTCTGACTGTTTCTGGTTTTCTGCTCGGTTTAACAGAGAAAATTAAAATTGGTTCATTAAATCACATCATTACAACTCATCATCCTGTCGCCATAGCGGAGGAAGCTTGCTTATTGGATCAGTTAAGTGAAGGGAGATTTATTTTAGGGTTTAGTGATTGCGAAAAAAAAGATGAAATGCATTTTTTTAATCGCCCGGTTGAATATCAACAGCAACTATTTGAAGAGTGTTATGAAATCATTAACGATGCTTTAACAACAGGCTATTGTAATCCAGATAACGATTTTTATAGCTTCCCTAAAATATCTGTAAATCCCCATGCTTATACGCCAGGCGGACCTCGGAAATATGTAACAGCAACCAGTCATCATATTGTTGAGTGGGCGGCCAAAAAAGGTATTCCTCTCATCTTTAAGTGGGATGATTCTAATGATGTTAGATATGAATATGCTGAAAGATATAAAGCCGTTGCGGATAAATATGACGTTGACCTATCAGAGATAGACCATCAGTTAATGATATTAGTTAACTATAACGAAGATAGTAATAAAGCTAAACAAGAGACGCGTGCATTTATTAGTGATTATGTTCTTGAAATGCACCCTAATGAAAATTTCGAAAATAAACTTGAAGAAATAATTGCAGAAAACGCTGTCGGAAATTATACGGAGTGTATAACTGCGGCTAAGTTGGCAATTGAAAAGTGTGGTGCGAAAAGTGTATTGCTGTCCTTTGAACCAATGAATGATTTGATGAGCCAAAAAAATGTAATCAATATTGTTGATGATAATATTAAGAAGTACCACATGGAATATACCTAATAGATTTCGAGTTGCAGCGAGGCGGCAAGTGAACGAATCCCCAGGAGCATAGATAACTATGTGACTGGGGTGAGTGAAAGCAGCCAACAAAGCAGCAGCTTGAAAGATGAAGGGTATAAAAGAGTATGACAGCAGTGCTGCCATACTTTCTAATATTATCTTGAGGAGTAAAACAGGTATGACTTCATATGTTGATAAACAAGAAATTACAGCAAGCTCAGAAATTGATGATTTGATTTTTTCGAGCGATCCATTAGTGTGGTCTTACGACGAGCAGGAAAAAATCAGAAAGAAACTTGTGCTTGATGCATTTCGTAATCATTATAAACATTGTCGAGAATATCGTCACTACTGTCAGGCACACAAAGTAGATGACAATATTACGGAAATTGATGACATACCTGTATTCCCAACATCGGTTTTTAAGTTTACTCGCTTATTAACTTCTCAGGAAAACGAGATTGAAAGTTGGTTTACCAGTAGCGGCACGAATGGTTTAAAAAGTCAGGTGGCGCGTGACAGATTAAGTATTGAGAGACTCTTAGGCTCTGTGAGTTATGGCATGAAATATGTTGGTAGTTGGTTTGATCATCAAATAGAATTAGTCAATTTGGGACCAGATAGATTTAATGCTCATAATATTTGGTTTAAATATGTTATGAGTTTGGTGGAATTGTTATATCCTACGACATTTACCGTAACAGAAGAACGAATAGATTTTGTTAAAACATTGAATAGTCTTGAACGAATAAAAAATCAAGGGAAAGATCTTTGTCTTATTGGTTCGCCATACTTTATTTATTTACTCTGCCATTATATGAAAGATAAAAAAATCTCATTTTCTGGAGATAAAAGCCTTTATATCATAACCGGAGGCGGCTGGAAAAGTTACGAAAAAGAATCTCTGAAACGTGATGATTTCAATCATCTTTTATTTGATACTTTCAATCTCAGTGATATTAGTCAGATCCGAGATATATTTAATCAAGTTGAACTCAACACTTGTTTCTTTGAGGATGAAATGCAGCGTAAACATGTTCCGCCGTGGGTATATGCGCGAGCGCTTGATCCTGAAACGTTGAAACCTGTACCTGATGGAACGCCGGGGTTGATGAGTTATATGGATGCGTCAGCAACCAGTTATCCAGCATTTATTGTTACCGATGATGTCGGGATAATTAGCAGAGAATATGGTAAGTATCCCGGCGTGCTCGTTGAAATTTTACGTCGCGTCAATACGAGGACGCAGAAAGGGTGTGCTTTAAGCTTAACCGAAGCGTTTGATAGTTGAGGCCGCACTCGAGCACCACCACCACCACCACTGAGATCCGGCTGCTAACAAAGCCCGAAAGGAAGCTGAGTTGGCTGCTGCCACCGCTGAGCAATAACTAGCATAACCCCTTGGGGCCTCTAAACGGGTCTTGAGGGGTTTTTTGCTGAAAGGAGGAACTATATCCGGATTGGGCGGCC
